# A unified global genotyping framework of dengue virus serotype-1 for a stratified coordinated surveillance strategy of dengue epidemics

**DOI:** 10.1186/s40249-022-01024-5

**Published:** 2022-10-13

**Authors:** Liqiang Li, Xiang Guo, Xiaoqing Zhang, Lingzhai Zhao, Li Li, Yuji Wang, Tian Xie, Qingqing Yin, Qinlong Jing, Tian Hu, Ziyao Li, Rangke Wu, Wei Zhao, Sherman Xuegang Xin, Benyun Shi, Jiming Liu, Shang Xia, Zhiqiang Peng, Zhicong Yang, Fuchun Zhang, Xiao-Guang Chen, Xiaohong Zhou

**Affiliations:** 1 Institute of Tropical Medicine, Southern Medical University, Guangzhou, 510515 China; 2 Key Laboratory of Prevention and Control for Emerging Infectious Diseases of Guangdong Higher Institutes, Guangdong Provincial Key Laboratory of Tropical Disease Research, Department of Pathogen Biology, School of Public Health, Southern Medical University, Guangzhou, 510515 China; 3 Institute of Infectious Diseases, Guangzhou Eighth People’s Hospital, Guangzhou Medical University, Guangzhou, 510060 Guangdong China; 4 State Key Laboratory of Organ Failure Research, Guangdong Provincial Key Laboratory of Tropical Disease Research, Department of Biostatistics, School of Public Health, Southern Medical University, Guangzhou, 510515 China; 5 Guangzhou Center for Disease Control and Prevention, Guangzhou, 510440 China; 6 School of Foreign Studies, Southern Medical University, Guangzhou, 510515 China; 7 BSL-3 Laboratory (Guangdong), School of Public Health, Southern Medical University, Guangzhou, 510515 China; 8 Laboratory of Biophysics, School of Medicine, South China University of Technology, Guangzhou, 510006 China; 9 School of Computer Science and Technology, Nanjing Tech University, Nanjing, 211816 China; 10 Department of Computer Science, Hong Kong Baptist University, Kowloon, Hong Kong, 999077 China; 11 National Institute of Parasitic Diseases at Chinese Center for Disease Control and Prevention (Chinese Center for Tropical Diseases Research), NHC Key Laboratory of Parasite and Vector Biology, WHO Collaborating Centre for Tropical Diseases, Shanghai, People’s Republic of China; 12 School of Global Health, Chinese Center for Tropical Diseases Research, Shanghai Jiao Tong University School of Medicine, Shanghai, 200025 China; 13 Guangdong Provincial Center for Disease Control and Prevention, Guangzhou, 511430 China

**Keywords:** Dengue virus serotype-1 (DENV-1), Molecular epidemiology, Population structure, Phylogeography, Global genotyping framework, Molecular surveillance

## Abstract

**Background:**

Dengue is the fastest spreading arboviral disease, posing great challenges on global public health. A reproduceable and comparable global genotyping framework for contextualizing spatiotemporal epidemiological data of dengue virus (DENV) is essential for research studies and collaborative surveillance.

**Methods:**

Targeting DENV-1 spreading prominently in recent decades, by reconciling all qualified complete E gene sequences of 5003 DENV-1 strains with epidemiological information from 78 epidemic countries/areas ranging from 1944 to 2018, we established and characterized a unified global high-resolution genotyping framework using phylogenetics, population genetics, phylogeography, and phylodynamics.

**Results:**

The defined framework was discriminated with three hierarchical layers of genotype, subgenotype and clade with respective mean pairwise distances 2–6%, 0.8–2%, and ≤ 0.8%. The global epidemic patterns of DENV-1 showed strong geographic constraints representing stratified spatial-genetic epidemic pairs of Continent-Genotype, Region-Subgenotype and Nation-Clade, thereby identifying 12 epidemic regions which prospectively facilitates the region-based coordination. The increasing cross-transmission trends were also demonstrated. The traditional endemic countries such as Thailand, Vietnam and Indonesia displayed as persisting dominant source centers, while the emerging epidemic countries such as China, Australia, and the USA, where dengue outbreaks were frequently triggered by importation, showed a growing trend of DENV-1 diffusion. The probably hidden epidemics were found especially in Africa and India. Then, our framework can be utilized in an accurate stratified coordinated surveillance based on the defined viral population compositions. Thereby it is prospectively valuable for further hampering the ongoing transition process of epidemic to endemic, addressing the issue of inadequate monitoring, and warning us to be concerned about the cross-national, cross-regional, and cross-continental diffusions of dengue, which can potentially trigger large epidemics.

**Conclusions:**

The framework and its utilization in quantitatively assessing DENV-1 epidemics has laid a foundation and re-unveiled the urgency for establishing a stratified coordinated surveillance platform for blocking global spreading of dengue. This framework is also expected to bridge classical DENV-1 genotyping with genomic epidemiology and risk modeling. We will promote it to the public and update it periodically.

**Graphical Abstract:**

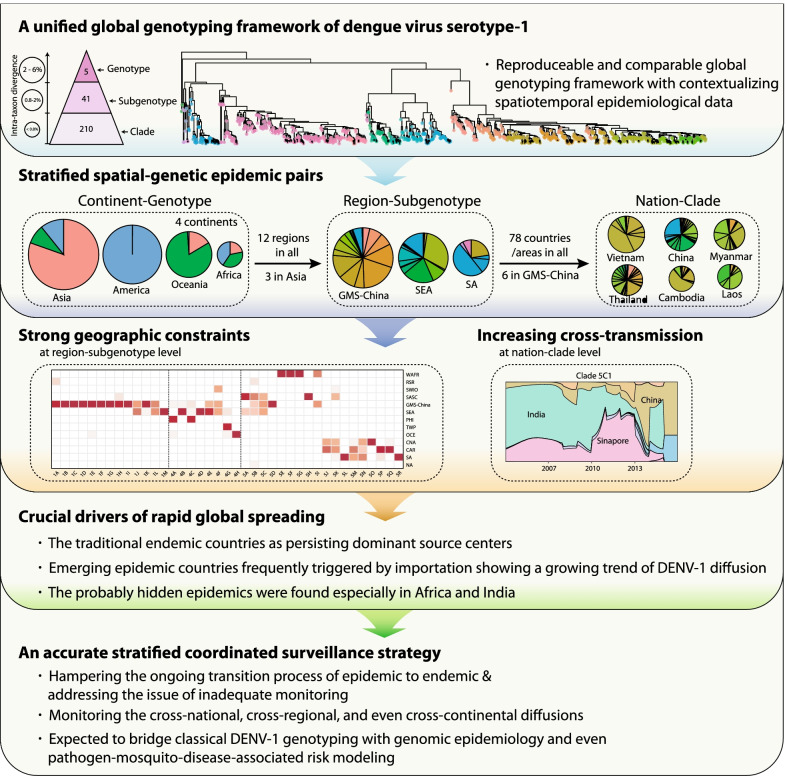

**Supplementary Information:**

The online version contains supplementary material available at 10.1186/s40249-022-01024-5.

## Background

Dengue, caused by dengue virus (DENV) infection [1] and mainly transmitted by *Aedes* mosquitoes, is now considered the most prevalent arboviral disease in human, and it circulates predominantly in tropical and subtropical regions, with more than half the world’s population at its risk [2, 3]. In the last twenty years, the global disease burden of dengue has risen about twofold [4]. Meanwhile, the spread of the *Aedes* vectors to previously unaffected areas is still ongoing, driven by human/cargo movement and continued global warming [5]. Furthermore, DENV has become more threatening and vaccine development is more difficult due to antibody-dependent enhancement, in view of alternate infection of multi-serotypes or even multi-genotypes of the virus [6, 7]. In 2019 dengue was classified by the World Health Organization as “the world’s most important mosquito-borne viral disease” [8]. To reverse this trend, myriad of means like outbreak prediction and coordinated epidemiological and entomological surveillance [8] are imperative for the containment of its morbidity. This measures can be combined with vector control interventions, diagnostics, triage prognostic systems, evidence-based clinical interventions and candidate vaccine development.

Urbanization, climate change, and globalization including rising global tourism are re-shaping the phylogeographic structure of virus populations, possibly contributing to a succession of severe viral pandemics as an unprecedented challenge to global public health [9]. To investigate viral dispersion mechanisms in phylogeography and phylodynamics, various analytical methods [10–12] and general genotyping methods have been developed for more accurate pathogen identification beyond viral species. However, few high-resolution genotyping scheme and standards in global scale which can contextualize local or regional epidemics are developed except the Avian influenza H5N1 [13]. Recently, the high-resolution genotyping scheme for surveillance manifested itself to be powerful in rapidly tracking the active lineages and transmission chains of SARS-CoV-2 [14]. It is still urgent to establish schemes and the matched epidemiological information for accurate identification of circulating viral populations in ordinary or even real-time surveillance.

The genetic diversity of DENV results from the variation and immune evasion of the viral genetic pools both in mosquito and human hosts [15, 16]. Antigenically and genetically, DENV has traditionally been classified into four serotypes [7]. To date, tremendous epidemiological investigations of dengue locally and regionally have been performed periodically. Then, along with the accumulation of DENV genetic information, its global spreading history and patterns, and even along with its origin and diversification have been gradually demonstrated [16–20]. The intra-genotype lineages of DENV have been recognized circulating in the same spatiotemporal context, which possibly arouses intra-genotype competition and consequently impacts genetic DENV diversity [19]. With strongly restricted genetic recombination [16], DENV is postulated to be clonal and structured by clonal population in genetics [21]. Moreover, DENV is found to present significant different population structures geographically discriminated by genotypes [22, 23], and its epidemics are concluded to be limited by geographical distance especially at the continental level [24].

At this point, it is crucial to characterize the frequent lineage replacements [25–27], identify the emergence and introduce new DENV populations for the studies on its evolution and transmission dynamics as well as its future surveillance [7, 28, 29]. Even though disease burden assessment and prediction using mosquito ecological data and dengue epidemic data [1] have been investigated globally, there is still lack of a systematic scheme for understanding the DENV population structure, dynamics and mechanisms of intercountry or regional transmission in a global profile [29, 30]. Several efforts have been tried to establish its global uniformed schemes of DENV beyond serotype by genotype using genomes or gene sequences based on different genetic resources [31–33]. However, none of these schemes has progressed into the prospect of utilization in its surveillance in reality, because of less characterization of epidemiological information and lack of reproduceable and comparable nomenclature system.

Dengue virus serotype 1 (DENV-1) is one of the first two serotypes identified [34], and has spread worldwide together with the other three [35]. DENV-1 is frequently symptomatic during primary and secondary infections and can lead to severe disease outcomes [36]. This serotype is predominantly epidemic in Asia, America and Oceania. In recent years, it has rapidly diffused, leading to a series of invasive outbreaks in temperate regions such as Madeira Island [37], Italy [38], and northern China [39]. Since five distinct genotypes (I, II, III, IV, and V) of DENV-1 have been identified based on complete E gene [17], E/NS1 gene junction [40], or genomic sequences [27], we limited our attention to DENV-1 and tried in this study to further define a unified global population framework of DENV-1 with higher spatiotemporal resolution, based on phylogenetics and population genetics, and characterize the transmission dynamics and geographical distribution of each viral population at stratified genotyping and geographical levels. Our study also provides insights into spreading, shifting and replacement of DENV-1, and contributes to developing an effective strategic implementation platform for coordinating global DENV-1 phylogenetic surveillance and public health interventions.

## Methods

The design of present study is summarized in Additional file 1: Fig. S Pre1. Additional file 1 including supplementary tables and figures is thereby presented in seven sections A to G.

### Virus sequences data selection

Aim at a comprehensive epidemiological coverage on whole spatiotemporal scale and in consideration of restriction by the amounts and quality of whole genome assemblies, a total of complete E gene sequences of 5409 DENV-1 isolates ranging from 1944 to 2018 were retrieved from the ViPR (Virus Pathogen Resource) up to April 9, 2018 (ViPR Workbench: 915064291709) [41], and of 44 DENV-1 isolates in Guangzhou from 2013 to 2017 sequenced and assembled in this study, together with the contextual epidemiological information including locations and isolating time of strains which confirmed by literature search, were collected (Additional file 2: Appendix data S A1). Only 5017 complete E gene sequences of DENV-1 recorded in 82 countries/areas were included after screening out the repetitive strains, the modified sequences by point mutation in the laboratories and those containing the unidentified segments, and then verified and augmented with the epidemiological information obtained through reviewing the literature and checking in GenBank (www.ncbi.nlm.nih.gov). Recombination events among the selected sequences were then assessed by Recombination detection program (RDP v.4.97, [42]) incorporated 7 algorithms [42] so that 14 sequences potentially associated with recombination events supported by at least one algorithm were removed (Additional file 1: Table S A1). The sequences from the non-epidemic countries, e.g., Germany, Ireland, Russia, and The Republic of Korea were included in the exporting countries visited by the returning travelers from the four countries for the further epidemic analysis. Ultimately, the complete E gene sequences of 5003 DENV-1 strains and 78 epidemic countries/areas were eligible for inclusion in the present systematic analyses (Additional file 1: Table S A2).

### Phylogenetic analysis

The phylogenetic tree of the 5003 DENV-1 strains based on alignments of complete E gene sequence were inferred by IQ-TREE v.1.6.8 [43] using maximum-likelihood (ML) method under the best-fitting substitution model evaluated and selected by ModelFinder [44], and the robustness was assessed by 5000 ultrafast bootstraps (UFBoot) [45]. After the GTR+F+R6, JC, and GTR+F+R5 substitution models were inferred by Akaike information criterion (AIC), corrected Akaike information criterion (AICc), and Bayesian information criterion (BIC), respectively, GTR+F+R5 was selected as the best-fitting substitution model. Consequently, 2952, 5, 3, 560 and 1485 isolates in the respective five genotypes I, II, III, IV, and V of DENV-1 were clearly recognized and verified, which is consistent with the previous genotype classification of DENV-1 based on the sequences of complete E gene [17, 23], E/NS1 gene junction [40], and genome of DENV-1 [27], but different from another genome-based classification of DENV-1 as the three genotypes I, II, and III in several previous studies (to avoid confusion, herein we rename the later genome-based genotypes as gI, gII, and gIII, with a probable explanation that gII and gIII are equivalent to respective genotype III and V of DENV-1 described in this study, due to phylogenetic recognition) [46]. Then the stratification inferences of ML trees of genotypes I, IV, and V sequences separately under their respective best-fitting substitutions GTR+R4+F, TN+R3+F, and GTR+R4+F, were performed. Due to inadequate strains, the genotypes II and III of DENV-1 were excluded in our further stratified phylogenetic, phylogeographical and population genetic analyses.

### Population structure characterization

A phylogeny-free population genetics approach implemented in R package “rhierBAPS” (Hierarchical Bayesian Analysis of Population Structure) v.1.1.0 [47, 48] was used for stratification analyses of population structure of DENV-1. The optimal number of cluster was evaluated until the algorithm converged to a local optimum by rhierBAPS, with the prior upper bound of “max.depth” of 12 and “n.pops” of 30, indicating the levels of different hierarchical clustering and the initial cluster number respectively. The genetic mixture of population cluster was assessed using the log marginal likelihoods (logML) at each BAPS depth, representing a parameter for quality improving of inferences, as the lower logML means the higher genetic mixture. The geographical diversity at the country/area level was assessed through Simpson index (SI) and Shannon–Wiener index (SWI) using R package ‘vegan’ by treating one country/area as an individual community unit. SI represents 0 to 1 probability belonging to the same species when two sampling from one community [49], with 0 representing no diversity and 1 infinite diversity. Meanwhile, SWI of diversity quantifies the uncertainty in the strings of text, with higher SWI meaning higher diversity [50].

Because of their heterogenicity, the genotypes I, IV, and V of DENV-1 were separately inferred. Estimated by BAPS, the logML of each clustering level increased within the fifth level and then reached a plateau, while the number of clusters increased within the sixth level and then reached a platform. The geographical diversities shown both by SI and SWI decreased to the fifth clustering level and then reached a bottom platform. Numbers of population cluster for genotypes I, IV, and V mounted to a platform, with the number of 247 clusters at depth 8 and 248 clusters at depth 9 for genotype I, 100 clusters at depth 8 for genotype IV, and 204 clusters at depth 8 for genotype V. The mean values of genetic mixture index logML and the geographical diversity indexes SI and SWI, kept steady accordingly since depth 8, showing − 452.4136, 0.1103 and 0.1907 respectively for genotype I; − 150.1962, 0.1145 and 0.1836 for genotype IV; and − 254.4943, 0.1125 and 0.1847 for genotype V. These were theoretically maximum numbers of viral population, with deeper clustering providing no greater resolution on genetic diversity and ecological diversity. Obvious intersections appearing around the levels 3–4 between the curves of number of initial clusters and the genetic and geographical diversity indexes, logML and SWI, SI, were observed (Additional file 1: Fig. S-B1), suggesting the balancing levels of clustering for viral population delamination. Thus, we selected raw BAPS clustering levels from 1 to 4 as following subgenotype and clade assignments, and level 4 as the extremity referenced for clade.

### Subgenotype, clade assignments and robustness assessment

Since DENV-1 displays star-like radical but drift hierarchic phylogeny, it is reasonable that its lineages are classified and subgenotyped at approximately equal or comparable genetic distances. Based on above population structure analysis, a detailed genotyping scheme was developed through assigning subgenotypes and clades hierarchically. Meanwhile, the genetic diversity parameter, MPD (mean pairwise distance), was adopted for determination of subgenotypes and clades for the three reasons: Firstly the MPDs of genotype I were the lowest, 0.0228 ± 0.0014 as compared with those of genotype IV and V (0.0432 ± 0.0027, 0.0359 ± 0.0026, respectively) when calculated by MEGA v.7.0.26 (Sudhir Kumar, Koichiro Tamura, Masatoshi Nei, and The Pennsylvania State University, PA, USA) [51], secondly the MPDs value of 0.02 was determined as the MPDs upper limit for subgenotype delimitation as the MPDs of 90% BAPS clusters generated from depth level 1 were below 0.02, and finally the MPDs of BAPS clusters generated from depth levels 2 to 4 were all below 0.01 and meanwhile the majority of BAPS clusters (90%) at depth 4 were covered when the threshold value was set at 0.008, which was selected as the upper limit of clade delimitation as well as the lower limit of subgenotypes of DENV-1 (Additional file 1: Fig. S B2).

Concretely, the subgenotypes of DENV-1 genotypes I, IV and V were assigned up to the following multiple criteria: (a) Referred to the BAPS clusters generated from depth levels 1 to 3, the subgenotypes were preliminarily designated based on the topology of the branches on the phylogenetic trees with assessed UFBoot values mainly at least higher than 70% represented unbiased support, preferably more than 90% indicated strong support; (b) If its MPDs was higher than 0.02, the assigned subgenotype was then split and optimized according to the topology of phylogenetic tree, with the corresponding UFBoot support values adjusted until the MPDs reached between 0.008 and 0.02 (Additional file 1: Tables S-B1, 3). Similarly, the clades were assigned as follows: (a) Referred to the BAPS clusters generated from depth levels 2 to 4, the clades of each subgenoytpe were preliminarily assessed based on the topology of the branches on the phylogenetic trees with assessed UFBoot values mainly at least > 70%, preferably > 90%; (b) If its MPDs was higher than 0.008, the assigned clade was then split and optimized according to the topology of phylogenetic tree, with the corresponding UFBoot support value adjusted until MPDs fell below 0.008 (Additional file 1: Tables S B2, C2). Finally, both the subgenotype and clade assignments, together with the spatiotemporal epidemiological information, SI, and SWI, were taken into further optimization of defining, especially when the UFBoot value of the lineage was less than 70% and its geo-position restrained from a single countries/areas (Additional file 1: Tables S B1–B2, C1–C2).

At last, for nomenclature, the genotyping framework of DENV-1 at three hierarchical levels of genotypes, subgenotypes, and clades were discriminated and labeled with [x], [y] and [z]. For [x], consecutive Arabic letters were used to name the genotypes, for [y] consecutive English letters to name the subgenotypes, and for [z] consecutive Arabic letters to name the clades. For example, genotype I was subdivided into subgenotypes 1A, 1B and so on, and subgenotype 1A was further subdivided into clades 1A1, 1A2 and so on. Simply, Clades 1A1 and 1A2 were sisters subordinate to subgenotype 1A in the secondary level and then to genotype I (1) of DENV-1 at the top level.

### Coalescent analysis, population dynamics

For ancestral state reconstruction and molecular clock phylogeny of the designated subgenotypes and clades, the complete E gene sequences of 910 out of 5003 strains from the 78 involving countries/areas, containing all the designated 41 subgenotypes and 210 clades with a full spatiotemporal range, were selected for subsequent evaluation and time-resolved phylogenetic analyses. First, a maximum-likelihood (ML) phylogenetic tree was inferred by IQ-TREE v.1.6.8 [43] under the GTR+I+Γ nucleotide substitution model and assessed by 5000 ultrafast bootstraps as above. Then, the correlation coefficient between root-to-tip genetic divergence and the sampling dates of strains of DENV-1 were estimated by TempEst v1.5.1 [52], supporting ancestral state reconstruction and molecular clock phylogenetic analyses.

Time-resolved phylogenetic trees were inferred using BEAST v1.10.4 [52] with incorporated the GTR+I+ nucleotide substitution model and the relax molecular clock across branches because of imprecise sampling dates (only by year) [53–55]. Bayesian Skyline model was chosen for coalescent analysis. All the other priors used were left at their default values (BEAST XML files are available from https://github.com/GuoXiang9399/D1Frame). For the following posterior distribution exploration, the independent Markov Chain Monte Carlo (MCMC) was used with at least 200 million generations in each analysis, after utilizing the BEAGLE library to accelerate computation [56]. All the parameters were checking and diagnosed using Tracer v1.7.1 until the effective sample sized (ESS) over 200 to ensure that the parameters were convergent [57]. Maximum clade credibility (MCC) trees were summarized using TreeAnnotator v1.10.4 after discarding 10% as burn-in. The most recent common ancestor (tMRCA) values, age(root) and the substitution rate values, meanRate, were retrieved from the log file using Trace Tracer v1.7.1 [57]. Population size and dynamics were assessed from sampled MCC trees using Bayesian Skyline plot method implemented in BEAST package without dependence on a prespecified parametric model of demographic history [55].

For in-depth exploration of characteristics of DENV-1 global epidemics, 31 subgenotypes and 47 clades in genotype I, IV, and V (including 8 singletons of subgenotypes which contained only one clade) with more than 20 strains and the rigorous root to tip correlation coefficient were selected for ancestral evaluation and substitution rates assessment following the above coalescent analysis method (BEAST XML files, https://github.com/GuoXiang9399/D1Frame). The substitution rates, tMRCA estimates, and their distributions were extracted using Tracer (Additional file 1: Tables S C1–C2). The stratified time-scale phylogenetic trees of 31 subgenotypes and 47 clades in DENV-1 were established by BEAST v1.10.4 [52]. After adjusting for strain numbers, MPDs, and SI, 47 clades as above mentioned were further included to examine the correlation between the substitution rates and tMRCAs using a linear regression model.

### Geographical clustering

To reduce the sampling bias possibly leading to imprecise epidemic demography among the 78 included countries/areas, we integrated detailed epidemiological backgrounds with geographical clustering. In this way, 32 countries/areas in Asian, American, and Oceanian continents recorded with more than 10 strains of DENV-1 were selected for preliminary geographical hierarchical clustering based on composition and frequencies of the defined genotype and subgenotypes using partitioning around medoid (PAM) algorithm, which was implemented with the factoextra package in R package “vegan” (Additional file 1: Fig. S E4). The optimal number of clusters was estimated using Silhouette method. Because the amount of reported data is not enough for hierarchical clustering by PAM, the other 46 countries or areas with strain number less than 10, including all of the African countries involved were manually assigned mainly based on their epidemic subgenotype composition and geographical proximity (Fig. 2a, b). Consequently, 12 epidemic regions corresponding to 12 clusters were assigned (Additional file 1: Table S E1). Then, geographical circulating distributions of DENV-1 at the genotype, subgenotype and clade levels were respectively demonstrated at global, regional and national scales.

### Phylogeographic analyses

Only those subgenotypes of DENV-1 with more than 20 strains were eligible for phylogeographic pattern analysis and thereby only 29 subgenotypes of DENV-1 involving 67 epidemic countries or areas were included in the analyses. The phylogeographical inferences were estimated by Bayesian stochastic search variable selection (BSSVS) procedure under discrete symmetric trait substitution model, coupling a standard continuous-time Markov chain (CTMC) computation [58] and using GTR+I+Γ nucleotide model of nucleotide substitution [59], with uncorrelated relaxed clock [53] and a Bayesian Skyline coalescent process prior settled [54, 55] in BEAST (v.1.10.4; BEAST XML files are available at https://github.com/GuoXiang9399/D1Frame).

The binary indicator (*I*) and Bayesian factor (BF) value were calculated using SpreaD3 [60] and then used to explore the potential of cross transmission [58]. The country pairs estimated with *I* > 0.50 and the BF > 6 were denoted by a migration pathway, with 6 ≤ BF < 10, 10 ≤ BF < 30, 30 ≤ BF < 100, 100 ≤ BF < 1000, and BF > 1000 indicating support, substantial support, strong support, very strong support, and decisive statistical support, respectively [61]. The transmission rates of transmission events per lineage per year were retrieved from the output files of CTMC computation using Tracer [57].

### Phylodynamics analyses

Forty-seven clades with strains number > 20 circulating in the 67 countries/areas were subject to demonstrating spatiotemporal dynamics and its relative global diffusion patterns. The phylogeographic inferences were first estimated for these clades, yielding posterior trees. Epidemic dynamics were then reconstructed using posterior analysis of coalescent trees (PACT) v0.9.5 [62] by 0.1 year step ward from these posterior trees clade by clade. Location of the trunk of each clade was inferred through time (Additional file 1: Fig. S G1) [63]. High trunk probability indicated a potential epidemic status, thus, for a clade, the countries or areas with a trunk probability > 50% were considered as the epidemic locations; while that with a trunk probability > 50% lasting at least 12 consecutive months, were considered as the dominant epidemic locations. These statistics were summarized and subjected to epidemic analysis. The viral transmission from the trunk location was assessed by the coalescent estimates of migration rates and direction, calculated by Migrate v3.0.8 in PACT from above yielded posterior trees by BEAST. Independent analyses of 100 resampling replicates were performed to balance differences between the overall sampling resolution and temporal sampling pattern [62].

### Data visualization

R 3.5.3 (R Foundation for Statistical Computing, Vienna, Austria) with package “ggplot2”, “ggtree”, “pheatmap”, “scatterpie”, “ggridges”, and “cowplot” were applied to achieve data visualization [64]. The stratification maps of geographic distribution of DENV-1 were conducted using R 3.5.3 (R Foundation for Statistical Computing, Vienna, Austria) with package “maps” and “scatterpie”. All of the data visualization was conducted using R 3.5.3 (R Foundation for Statistical Computing, Vienna, Austria).

## Results

### Establishment of a unified high-resolution global genotyping framework of DENV-1

**Fig. 1:**
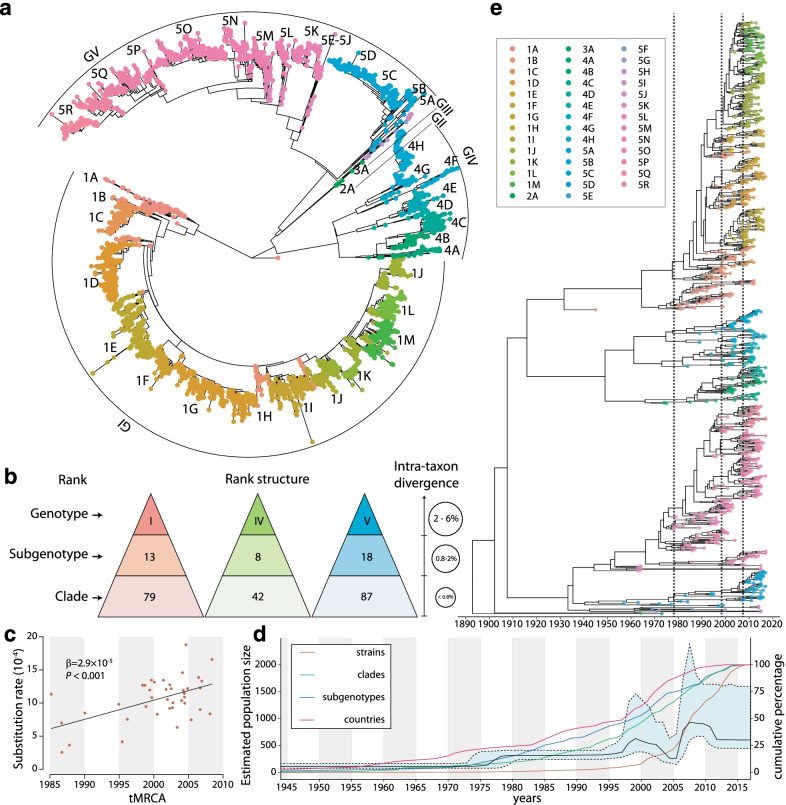
Global genotyping framework of DENV-1 established based on its population structure, phylogeny, and epidemiology. **a**, **e** The dots at the tip of each strains are marked with different colour representing 41 subgenotypes identified. **a** The unified global framework of DENV-1 subgenotypes is labeled in the phylogenetic tree of the complete E gene sequences of 5003 DENV-1 strains worldwide. Due to inadequate strains recorded, only 5 and 3 strains of genotype II and III were defined in a single subgenotype (2A and 3A) and clade (2A1 and 3A1), respectively. **b** The defined genotyping framework of DENV-1 represents an increasing intra-taxon divergence of the mean pairwise distances (MPDs). **c** The correlation between the substitution rate and time to the most common ancestor (tMRCA) was assessed using a linear regression model, after adjusting the inclusive number of strains, MPDs, and Simpson Index. **d** The dynamics description of the estimated viral population size of DENV-1 with the cumulative percentages of inclusive strains, clades, subgenotypes of DENV-1, and the involving epidemic countries or areas. **e** The temporal phylogeny of DENV-1 using 910 strains selected among the total 5003 ones worldwide which cover the full spatiotemporal ranges and represent all subgenotypes and clades in genotype I, IV, and V of DENV-1. The features of the selected 910 E complete sequences are presented in Additional file 3: Appendix-Data S-C1. The linear regression of root-to-tip genetic divergences versus strain sampling dates is displayed in Additional file 1: Fig. S C1. The gray vertical dotted lines show three sharp increases in the estimated viral population size of DENV-1 during the three periods of 1976–1977, 1996–1998, and 2005–2006

Incorporated with the population structure, phylogenetic features and epidemiological backgrounds, especially the balances between geographical distribution and genetic diversities, the designated global genotyping scheme of DENV-1 was proposed with a three-layer hierarchical structure-genotype, subgenotype and clade, with MPDs ≤ 0.8%, 0.8–2%, and 2–6%, respectively. Based on the five DENV-1 genotypes, with this hierarchical scheme, we identified 41 subgenotypes and 210 clades with phylogenetical and geographical information (Fig. 1a, b; Additional file 1: Figs. S B1–B3; Tables S A1–A2). Among them, 2952, 560, and 1485 strains of the genotype I, IV and V were sorted out into 13, 8, and 18 subgenotypes, and then 79, 42, and 87 clades, respectively (Additional file 1: Tables S B1–B2, C1–C2). For the genotypes I, IV and V, the respective averaged substitution rate in the subgenotypes were 10.37 × 10^–4^, 9.73 × 10^–4^ and 6.95 × 10^–4^, while in clades were 11.57 × 10^–4^, 9.76 × 10^–4^ and 8.67 × 10^–4^, compatible with previous assessed range at the genotype level [16]. The averaged substitution rates for the latter emerging clades displayed an increasing trend along time, but showed no correlation with strain counts, MPDs and SI (linear regression model, β = 2.9 × 10^–5^, *P* < 0.001, Additional file 1: Table S–D1; Fig. 1c; Additional file 1: Figs. S C2–C3, D1), which might be caused by sequence saturation and purifying selection, as discovered from the previous studies on other virus in a macro temporal scale (thousand years). These results suggest that the emergence and divergence of DENV-1 populations especially in respect to the substitution rates at 10 years interval might be featured with dynamicness rather than staticness [65–67]. However, the relationship between the increased substitution rates accompanied with the accelerated expansion of DENV-1 populations as demonstrated in the following is worth further exploring.

The estimated virus population size was viewed to increase sharply at the three respective periods of 1976–1977, 1996–1998, and 2005–2006 worldwide (Fig. 1d, e). Before 2000, the numbers of newly affected countries and newly detected subgenotypes went up steadily though the number of newly isolated strains was at slow elevation, and the number of newly emerging clades dramatically increased during 1985–1990 and then kept climbing through the second and third estimated population bursts (Fig. 1d, e) [22] till 2010 (Additional file 1: Fig. S D1) [26], which is consistent with the epidemic history established by evidence-based method [35]. The number of newly reported infection countries spiked around 2005, and subsequently the numbers of new subgenotypes and clades in 2005–2010 were characterized by the third population burst (Fig. 1d, e). Moreover, the time interval from tMRCA to the first epidemic source of each clade was about 2 years (average 1.92 ± 1.11, 1.74 ± 1.29 and 2.10 ± 2.06 years for clades in genotype I, IV and V, respectively), suggesting a stable and cryptic population expansion during the emergence and outbreak of a new clade (Additional file 1: Table S C2). Above all, the trends of geographical expansion are supposed to be an important reason for DENV-1 population bursting.

### Global geographic distribution and epidemic regional classification

**Fig. 2:**
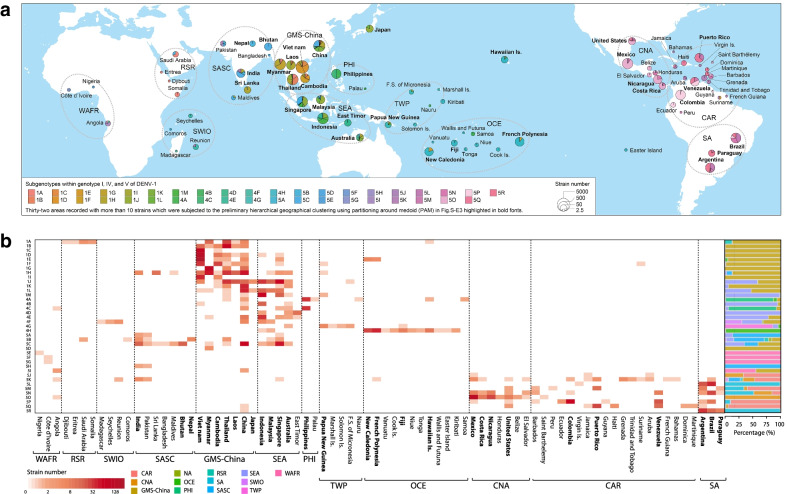
Geographical clustering and classification of the twelve epidemic regions of DENV-1 worldwide by countries/areas at the subgenotype level. **a** Global distribution of the twelve epidemic regions of DENV-1. The pies are sized to indicate the number of strains and the slices are colored by subgenotypes. WAFR, West African Region; RSR, Red Sea Region; SWIO, Southwest Indian Ocean; SASC, South Asia Subcontinent; GMS-China, Great Mekong Subregion-China; SEA, Southeast Asia; PHI, Philippines; TWP, Tropical Western Pacific; OCE, Oceania; CNA, Central North America; CAR, Caribbean; SA, South America. **b** Left panel: heatmap of distribution of subgenotypes of DENV-1 represented by countries/areas in the defined 12 epidemic regions, and the 32 countries/areas recorded with more than 10 strains subject to the preliminary hierarchical geographical clustering using partitioning around medoid (PAM) in Additional file 1: Fig. S-E3 highlighted in bold fonts. Right panel: the proportion of involving epidemic regions counted by the number of strains in each subgenotypes of DENV-1

By geographic distribution analysis, we found that DENV-1 epidemics were evidently constrained geographically by the spatial-genetic pairs of Continent-Genotype (Additional file 1: Fig. S E1), Region-Subgenotype (Fig. 2), and Nation-Clade (Additional file 1: Fig. S E2). Among them, the composition of DENV-1 subgenotypes were significantly similar among the neighboring countries, indicating the characteristic of being highly constrained which is of great significance for the further establishment of regionally coordinated surveillance system. In this way, 12 epidemic regions in our study, namely, GMS-China, Great Mekong Subregion-China; SEA, Southeast Asia; PHI, Philippines; TWP, Tropical Western Pacific; OCE, Oceania; WAFR, West African Region; RSR, Red Sea Region; SWIO, Southwest Indian Ocean; SASC, South Asia Subcontinent; CNA, Central North America; CAR, Caribbean; and SA, South America, were identified from the 78 epidemic countries/areas (Fig. 2; Additional file 1: Figs. S E3–E5; Table S E1).

As for the pair of the Region-Subgenotype, 92.3% and 71.8% of the 39 designated subgenotypes, with ≥ 50% and ≥ 80% isolates respectively, mainly circulated in a single region, while 20.1% were confined to a specific region (Fig. 2; Additional file 1: Fig. S E4). Concretely, the subgenotypes 1A–I and 1K spread restrictively in GMS-China; 1 M, 4B, 4D and 4E in SEA; 5E, 5F, 5G, 5I in WAFR; 5A and 5H in SASC; 4A, 4C in PHI; 5O in CNA; 4H in OCE; 4G in TWP; 5M, 5P, 5Q in CAR; 5L, 5N and 5R in SA (Fig. 2b). The results suggest that the countries/areas in each region may share epidemics and transmission cycles/ecology, potentially creating barriers to cross-regional dispersion.

As for the pair of National-Clade, similarly, 91.8% and 70.2% of the 208 designated clades, with ≥ 50% and ≥ 80% isolates respectively, mainly circulated in a single country, while 54.3% solely constrained in a single country (Additional file 1: Fig. S E6), suggesting that clades may originate from certain countries/areas and spread across countries. Meanwhile, we observed that most of country-dominant clades were recorded in GMS-China (61, majorly in Thailand, Myanmar, China and Vietnam), SEA (28, majorly in Indonesia and Singapore), CNA (19, major in Mexico), CAR (25, much equably distributed in Colombia, Puerto Rico, Venezuela and Barbados); SASC (18, 14 in India), and PHI (16, 15 in the Philippines) (Additional file 1: Fig. S E6), suggesting high DENV-1 population diversity and sustainability occurred in these regions, especially in the above listed countries.

In addition, both the pairs of Region-Subgenotype and Nation-Clade displayed phylogenetic continuity. For example, the subgenotypes 1A–I and 5E–G were seen to remain relatively stable for years in GMS-China and WAFR, respectively (Fig. 2b; Additional file 1: Figs. S E4, E7a). Meanwhile, phylogenetic continuity of country-dominant clades was clearly observed in 11 countries/areas including Thailand, Vietnam, Myanmar, Indonesia, the Philippines, India, Papua New Guinea, Venezuela, Colombia, Mexico and Somalia, e.g. the clades of 5K1–5K6 in India (Additional file 1: Figs. S E6, E7b). These results suggest that the history of continuous variation and evolution of DENV-1 epidemic populations is restricted to specific regions/countries, which is possibly associated with periodical outbreaks and regional/national transmission dynamics.

Co-circulations or cross-transmissions of DENV-1 were also observed in the epidemic regions and countries/areas (Additional file 1: Fig. S E5a). Several subgenotypes including 5K, 5B, 4F, 5C, 5M and 5N were found spreading across several regions (Fig. 2b; Additional file 1: Fig. S E4). Interestingly, 17 clades except 1I1 of the subgenotypes 1H–1L were found co-circulating in more than three countries/areas in GMS-China and SEA. Twenty clades, 1D1, 1E1, 1F1, 4A10, 4C1, 4E4, 4F3, 4G5, 4H1, 5B5, 5C1, 5K12, 5K23, 5N10, 5O1, 5O2, 5P1, 5P9, 5Q1 and 5R1, spread to more than three countries and even some across regions. But it is unclear whether they were caused by large-scale spillovers during outbreaks, thereby it is critical to further investigate the factors driving the outbreaks and rapid spreading of DENV-1 populations.

It is noted that only 15 countries in Asia and Oceania, particularly in GMS-China, SEA, PHI, and seven countries in Americas, were supposed to be under more tense surveillance (Additional file 1: Table S A2), but most countries/areas were still under poor molecular epidemiological surveillance, comparing with 128 countries/areas involved in dengue epidemic worldwide [3]. In Africa and SASC, for example, the earliest isolates of DENV-1 were recorded in Nigeria (Africa, 1968) and India (SASC, 1956 with 8 defined clades, which initially showed a higher population diversity), but no isolate was reported in Africa during the 1970s and India during the 1990s, indicating the probable hidden epidemics due to lack of molecular evidence (Additional file 1: Fig. S E2).

### Global transmission pattern of DENV-1 at subgenotype level

**Fig. 3:**
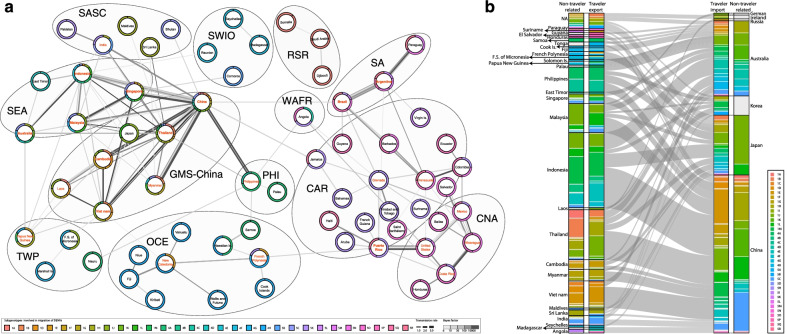
Global transmission patterns of DENV-1 at the subgenotype level. **a** The transmission patterns assessed by phylogeographical inferences using the Bayesian stochastic search variable selection (BSSVS). 29 subgenotypes of DENV-1 and 67 involving epidemic countries/areas were included, under the condition of the subgenotypes of DENV-1 with > 20 strains as well as the viral lineage transmission with the binary indicator (*I*) > 0.50 and Bayes factor (BF) > 6. The pies represent the country/area circled with the coloured composition of subgenotypes involving in transmission. The gray dotted oval lines show the twelve belonging epidemic regions. The names of countries/areas with ≥ 5 transmission pairs are shown in red, and ≥ 10 pairs in bold red. WAFR, West African Region; RSR Red Sea Region; SWIO, Southwest Indian Ocean; SASC, South Asia Subcontinent; GMS-China, Great Mekong Subregion-China; SEA Southeast Asia; PHI, Philippines; TWP, Tropical Western Pacific; OCE, Oceania; CNA, Central North America; CAR, Caribbean; SA, South America. **b** The alluvial plot indicates the subgenotype flow of DENV-1 by travelers. 329 isolates of DENV-1 were recorded from returning travelers. The importing countries and visited countries were confirmed through checking information in Genbank and corresponding references. There were 30 visited countries/areas and 7 importing countries/areas in total. The inner pairs of the alluvial plots show the subgenotype flow of 329 isolates from the visited (exported) countries/areas (Left) to the imported (Right), and the width of alluvial segments indicates the number of isolates. The outer pairs show the subgenotypes proportions of DENV-1 excluded data from travelers in the involving exported (Left) and importing (Right) countries/areas

By the BSSVS, we inferred totally 172 pairs of countries/areas with significantly supported transmission from the 29 included subgenotypes which epidemic in 67 countries/areas, finding that the transmission patterns were nearly specific in terms of the compositions of epidemic regions/countries and subgenotypes (Fig. 3a, Additional file 1: Figs. S F1–F2), evidently indicating DENV-1 populations were geographically constrained. We also found some transregional transmissions mostly in adjacent regions, especially in the regions of Asian or American Continents, but only a few transmissions across the continents. Particularly, the active DENV-1 transmission occurred (Fig. 3a, Additional file 1: Fig. S F2) in such countries/regions with more than 10 transmission pairs as Thailand, Cambodia, Vietnam and China of GMS-China; Singapore, Indonesia, Malaysia and Australia of SEA; Brazil and Argentina of SA and Puerto Rico of CAR, indicating that these countries/regions overwhelmingly affected by DENV-1 play a critical role in the global spreading and control of DENV-1 (Fig. 3a) [19].

Furthermore, we even found that in 16 of the 172 pairs of countries/areas co-circulated more than 3 subgenotypes, among which 12 were within or between GMS-China and SEA, and the rest 4 right in the American regions. For instance, the 5 subgenotypes 1A, 1B, 1G, 1J and 1K were found co-circulating in China-Thailand, 4 ones, 5L, 5N, 5P and 5R in Argentina-Brazil, and another 4 ones 1L, 1M, 4E and 5C in Indonesia-Singapore (Additional file 1: Table S F1). The transmission pairs with an estimated transmission rate ≥ 2 were China-Myanmar (3.03) with 1H co-circulating, China-Singapore (2.82) with 5C, and Indonesia-Singapore (2.38) with 1L (Additional file 1: Table S F2). Noteworthily, the transmission frequencies of subgenotypes 5K, 1J, 1H, 5N, 5C, etc. were high (Additional file 1: Tables S F1–F2), which consisted with their distribution widths (Fig. 2; Additional file 1: Fig. S E4). The complexity and diversity of these cross-region transmission and co-circulation are probably due to the rapidly rising international tourism, which was essentially proved by tracing from Indonesia, Malaysia, Thailand, Cambodia, Vietnam, the Philippines, and Myanmar, where the virus was exported not only into such epidemic countries as China, Japan and Australia, but into those non-epidemic countries such as The Republic of Korea, German, Ireland, Russia (Fig. 3b). Collectively, the transmissions of subgenotypes cross countries/regions, on the rise in recent decades (Fig. 1d), can explain its continuous global expansion to some extent.

### Sources and trend of DENV-1 diffusion at clade level

**Fig. 4:**
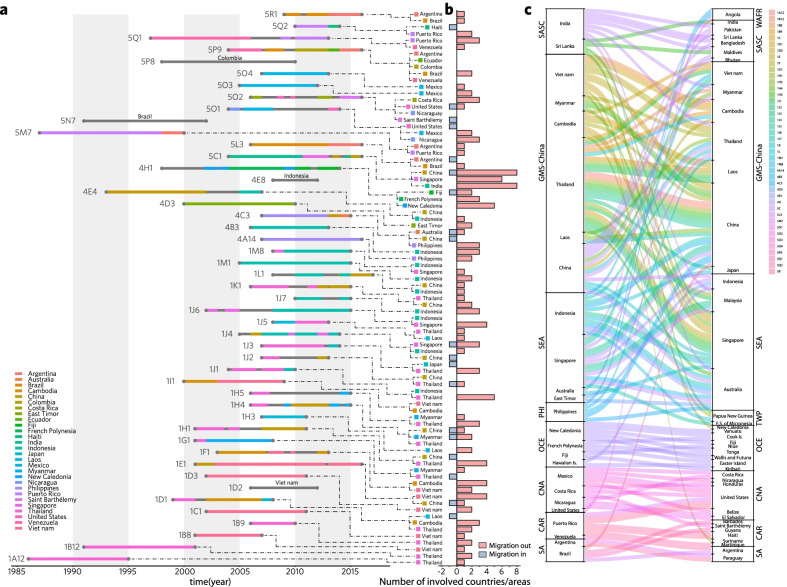
Global diffusion patterns of DENV-1 at the clade level inferred by phylodynamics analysis using posterior analysis of coalescent trees (PACT). **a** The involving epidemic country/area of DENV-1 with a trunk probability > 50% in certain clade in at least 12 consecutive months inferred by PACT, was considered as the dominant epidemic location of this clade. So 31 PACT-inferred dominant countries/areas epidemic with 47 clades of DENV-1 were estimated, represented with colour horizontal lines in the panel. Among them, 1D2, 4E8, 5N7, and 5P8 respectively broke out only in the single country-Vietnam, Indonesia, Brazil, and Colombia. The gray dots show the earliest and latest years of observed isolation for each clade of DENV-1, connected by thin gray lines. **b** The vertical bars show the number of migrant events in the 25 PACT-inferred dominant epidemic countries or areas when the migration rate was estimated > 0.1. Herein, six countries including Japan, Haiti, Paraguay, Saint Barthelemy, Colombia, and Ecuador were excluded due to the migration rate < 0.1. **c** The alluvial plot shows the diffusion of 42 clades (excluding 1D2, 4E8, 5N7, and 5P8 in a single country, and 1I1 in Vietnam and Cambodia) from the 25 PACT-inferred dominant countries/areas to 43 countries/areas in 9 epidemic regions**,** with the width of line indicating the value of migration rate

**Fig. 5:**
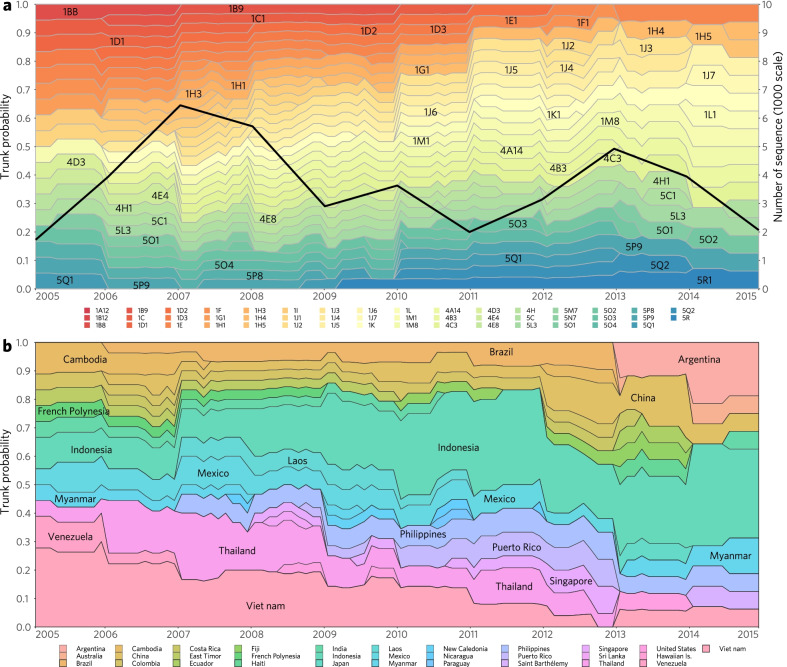
Dynamics and trends of the epidemic trunks of the 47 clades of DENV-1 from 2005 to 2015 and the involved PACT-inferred 31 dominant epidemic countries/areas. The data inferred by PACT showed in Fig. 4 were further subjected to time series dynamics analyses and visualized with 0.1 year step ward. **a** The proportion of epidemic trunks of the 47 clades of DENV-1 is changed dynamically through time. **b** The proportion of trunks of the involved PACT-inferred dominant epidemic countries/areas is changed dynamically through time

To assess the dynamics of epidemics history and predict their epidemic trends, we inferred the epidemic trunks for 47 clades involving 31 dominant countries/areas inferred by PACT (Fig. 4; Additional file 1: Fig. S G1) and then had the acquired data subjected to time series dynamics analyses and visualized with 0.1 year-step ward (Fig. 5), finding that DENV-1 populations drifted gradually in time series dynamics, while the new clades emerged always in parallel with the early clades for several years along within-genotype clade drift (Figs. 4a, 5a). The majority of clades (43/47) of genotypes I, IV or V appeared and circulated after 2005 (Figs. 4a, 5a), consistent with the DENV-1 population burst illustrated in Fig. 1d. Eighteen of the 47 clades circulated dominantly in a single country for years (Fig. 4a) and they were mainly exported to the adjacent countries/areas (Fig. 4b, c; Additional file 1: Fig. S G1).

From the recording of 1A12 in 1986 to the emerging of 1J3 in 2007, Indo-China Peninsula in Asia was the core source of clades of subgenotypes 1A to 1J. After 2000, the majority of the 47 epidemic clades emerged and preferentially transmitted within the countries of GMS-China or SEA (Fig. 4). Specifically in Thailand co-circulated 13 clades, the most in GMS-China (Figs. 4a, 5b). Around 2005, subgenotype 1J mainly stemmed from Thailand (Additional file 1: Figs. S E6a, S G1), and developed in parallel with the clades of subgenotypes 1L, 1M, 4B, 4D, and 4E, which burst and persisted in SEA and then exported to the neighboring GMS-China countries (Fig. 4c), especially in Indonesia circulated 10 clades, the most among SEA (Figs. 4a,5b; Additional file 1: Figs. S E6, S G1). Importantly, the Philippines was the major sourcing pool for subgenotypes 4A and 4C (Additional file 1: Fig. S E6; 4A14 and 4C3 in Fig. 4a), which are persistently circulated in the Philippines from the 1970s to the 2010s. Interestingly here, the clade of 5C1 was inferred to persist for more than one decade in India, and then it spread to Singapore and China, resulting in big outbreaks. Along the spreading process, it spilled over to various GMS-China and SEA countries. Countrywide, Vietnam was also eye catching because 7 clades co-circulated there, 5 of them persisting even more than 5 years (Figs. 4a, 5; Additional file 1: Figs. S G1–G2; Table S G1). As another case, China was a major destination with imported clades from most sourcing countries in Asia (Figs. 3, 4b), where 10 clades mainly co-circulated but 9 of them persisted ≤ 3 years except for 4E4 (Figs. 4a, 5b).

In American continent, all dominant circulating clades belonged to genotype V, and the majority of them emerged and became epidemically active since around 2005 except several clades sourcing in the 1980s (5M7) and the 1990s (5N7, 5P8 and 5Q1). These clades majorly co-circulated within American countries and transmitted restrictively within the three epidemics regions. During the epidemics, these clades were mainly sourced from the countries in/around CNA and CAR countries/areas (Puerto Rico, Mexico, Venezuela, the USA, Nicaragua, Saint Barthelemy, Haiti, Ecuador and Colombia) and SA countries like Argentina and Brazil (Figs. 4a, b, 5b). Actually, 4 clades of genotype V dominantly circulated in Brazil and Argentina in SA and 3 in Mexico of CNA and 3 in Puerto Rico of CAR. Otherwise, the clades of 5P8 and 5Q1 were also dominantly transmitted in Colombia and Venezuela respectively for more than 5 years (Additional file 1: Table S G1). In addition, clade 4H1 was found dominantly circulating in French Polynesia, Fiji, and New Caledonia of OCE. Pitifully, we did not obtain enough data from the regions of African continent for analysis.

## Discussion

Defining a taxon is central to biodiversity science, likewise defining the population of a virus should be the cornerstone for investigating its epidemic heterogeneity. Featured with high heterogeneity, parallel divergence and drift evolution [68], DENV is clonal genetically [21, 27], which determines its diffusion pattern in a spatiotemporal dimension. Despair myriad studies on its patterns geographically at continental level and genetically at genotype layer, a precise-resolution proposal is unseen especially from its respective subordinate levels. Thus, in the present study, reconciling with the relative epidemiological information confirmed by literature research, we established a unified global high-resolution framework of DENV-1. The genotype, subgenotype and clade of DENV-1 have been defined by respective hierarchical MPDs for characterizing its population. This framework in depth demonstrated that DENV-1 population presented stratified spatial-genetic epidemic pairs of Continent-Genotype, Region-Subgenotype and Nation-Clade comparatively, thereby eliciting its power in improving the global-connected DENV-1 surveillance.

Ever since the postulated emergence of DENV, its four serotypes have been spreading globally for centuries [35]. The genotypes of each serotype, circulating globally for decades, are restricted in certain continents [20]. Our stratified phylogenetic framework reveals that the current epidemic patterns of DENV-1 represent strong geographic constraints. The increasing cross-transmission trends were also demonstrated. This comparable global framework is thereby instructive for transmission blocking of DENV-1 which is critical for effective controls of the viruses, especially for the neighboring endemic or epidemic countries/regions [7]. In accordance with the previous studies on the rapidly increasing incidence and global disease burden of dengue [1], we observed an accelerating expansion trend in DENV-1 population worldwide, which increasing sharply at three respective periods. We found that the geographical expansion of the epidemics preceded the emergence of new clades and subsequent new subgenotypes, or in other words, the previous DENV-1 populations were transmitted to new countries with new genetic heterogeneity emerging and circulated persistently there after geographical separation [15]. DENV lineages were observed to increase exponentially in the initial invasion phase [30], which might attribute to lack of herd immunity or competitor virus. Then, our framework can be utilized in monitoring the clades/subgenotypes spreading cross countries/regions. Some clades can frequently transmit cross countries/regions quickly and even trigger consecutive outbreaks. For example, 5K spread out of South Asia about 40 years ago, and has established successfully local clonal expansions in American countries/areas [69]. While 5C/5C1 was traced from India and played an important role in the large outbreaks in Singapore (2013) [70] and consecutively in China (2014) [71]. On the other hand, the geographical range of dengue is expected to further expand due to ongoing global phenomena including climate change and urbanization [3]. Interdicting this process is another important facet to reverse DENV expansion. Our framework can be utilized to monitor and subsequently contribute to hamper the ongoing transition process of epidemic to endemic. For example, besides the traditional endemic countries such as Thailand, Vietnam, Indonesia, the Philippines, India and Brazil still apparently displayed as the persisting dominant source centers, the emerging epidemic countries such as China, Australia, and the USA, where dengue outbreaks were frequently triggered by importation, showed a gradually growing trend of diffusion of DENV-1. These all reveal the urgency and necessity for blocking dengue efficiently and promptly. In addition, the singletons in subgenotypes (12/41) and clades (63/208) recognized in our study imply that they may be only minor in DENV-1 population for one reason, and may be partly caused by widely deficient surveillance for another. We also found that it’s probably hidden epidemics of occurred in Africa and India, which is consistent with previous dengue case estimation [3]. Then, an accurate daily coordinated surveillance based on this defined population compositions of its clades and subgenotypes co-circulating within a single or multiple countries/regions is prospectively valuable to address the issue of inadequate monitoring.

Previous global DENVs control experiences indicated that sustainable dengue control must be developed on a regional basis [7]. In this study, we have designated the control strategies for each of 12 epidemic regions by their epidemic contents and patterns, and demonstrated that DENV-1 transmissions among countries within certain regions are intensive than cross regions. These 12 regions could be recognized as 12 separate units for further establishment of an important coordinated inter-country prevention and control system, which is conducive to monitoring and evaluation of the epidemic risks and target-controlling the virus by referring to those baseline surveillance data of the epidemic pairs of Region-Subgenotype and Nation-Clade. For instance, with the system the customs and border administrations can monitor the major viral subgenotypes/clades specifically from visited countries with high priority, and collect the epidemiological information, thereby tracking the outbreaks abroad and providing feedback to the affected countries for further coordinated controlling regionally. Meanwhile, we could also pay attention to the cross-national, cross-regional, and even cross-continental diffusions of DENV-1, which potentially arouse large epidemics, in reference to the stratified spatial-genetic epidemic pairs. International collaborations based on the framework in promptly pinpointing and periodically updating the hotspots of emerging, transmission, drifting, and replacement of DENV-1 will pave the way to cost-effective surveillance strategy for national, regional, and global control of dengue.

Furthermore, our framework is a reproducible and ready-to-reusable genotyping scheme from real genetic and epidemiological data other than mathematical modeling deduced by simulated virtual data, providing us with a compatible foundation which paves the way to further explorations into genetic epidemics in terms of phylogeny, phylogeography and phylodynamics. Due to the accessibility and affordability of E gene, our high-resolution genotyping can be employed as a transitional or linkage scheme reconciling the classical E gene genotyping with the emerging genomic epidemiology [72], which may facilitate in-depth molecular epidemiological and population genetic investigations, as showed in our another study (unpublished data). Prospectively, the global stratified scheme is surely beneficial for the real-time pathogen surveillance and further establishment of an applicable risk prediction of outbreaks through pathogen-mosquito-disease-associated modeling by way of combining genome, seroprevalence and vaccine coverage, continuous and structured mosquito surveillance, local clinical and traveler surveys, and situational awareness of arbovirus incidence, and ecological data [73].

Actually, what bewildered us in our study is that deficient access to the public epidemic information makes the spatial resolution less possible below country level and at a uniformly geographic distance [74]. This predicament should be improved gradually by broad consensus and full collaboration worldwide [9]. Otherwise, this genotyping scheme is also hoped to enhance its efficacy in surveillance over and control of dengue globally, supposed the data concerning complete historical strains by sequencing reserved samples could be introduced and the clades with insufficient strains be supplemented in the scheme.

## Conclusions

The present study has established a reproduceable and comparable global genotyping framework of DENV-1 with contextualizing spatiotemporal epidemiological information. This framework reveals that the persisting traditional endemic sourcing, the emerging epidemic diffusing, and the probably hidden epidemics are the crucial drivers of the rapid global spread of dengue. Its utilization in quantitatively assessing DENV-1 epidemics at respective Continent-Genotype, Region-Subgenotype, and Nation-Clade levels has laid a foundation and re-unveiled the urgency for developing a stratified coordinated surveillance strategy for blocking global spreading of dengue cost-effectively. The framework is also expected to bridge classical DENV-1 genotyping with current genomic epidemiology and even pathogen-mosquito-disease-associated risk modeling, and we will promote public availability to and periodic updating of it as well.

## Supplementary Information

**Additional file 1.** Supplementary Figures and tables for this study.

**Additional file 2.** Characteristics of 5003 DENV-1 isolates worldwide from 1944 to 2018 inclusive in this study.

**Additional file 3.** Features of the selected 910 E complete sequences for temporal phylogenic reconstruction covering all spatio-temporal distributions and representing all designated subgenotypes and clades in genotypes I, IV, and V from 5003 DENV-1 strains worldwide.

## Data Availability

Additional data that support the findings of this study are available from the corresponding author upon request.

## Abbreviations

DENV: Dengue virus
ViPR: Virus Pathogen Resource
RDP: Recombination detection program
ML: Maximum-likelihood
UFBoot: Ultrafast bootstraps
AIC: Akaike information criterion
AICc: Corrected Akaike information criterion
BIC: Bayesian information criterion
BSSVS: Bayesian stochastic search variable selection
PACT: Posterior analysis of coalescent trees
rhierBAPS: Hierarchical Bayesian Analysis of Population Structure
logML: Log marginal likelihoods
SI: Simpson index
SWI: Shannon–Wiener index
MPD: Mean pairwise distance
MCMC: Markov Chain Monte Carlo
ESS: Effective sample size
MCC: Maximum clade credibility
tMRCA: The most recent common ancestor
PAM: Partitioning around medoid
CTMC: Continuous-time Markov chain
BF: Bayesian factor
GMS: Great Mekong Subregion
SEA: Southeast Asia
PHI: Philippines
TWP: Tropical Western Pacific
OCE: Oceania
WAFR: West African Region
RSR: Red Sea Region
SWIO: Southwest Indian Ocean
SASC: South Asia Subcontinent
CNA: Central North America
CAR: Caribbean
SA: South America
