# Supplementary material for: A unified global genotyping framework of dengue virus serotype-1 for a stratified coordinated surveillance strategy of dengue epidemics
Source: Infect Dis Poverty. 2022 Oct 13;11:107. doi: 10.1186/s40249-022-01024-5 (PMC9556283; doi:10.1186/s40249-022-01024-5)
Supplement: Supplementary file 1 — Additional file 1. Supplementary Figures and tables for this study. [file 40249_2022_1024_MOESM1_ESM.pdf]

Additional file 1 for

A unified global genotyping framework of dengue virus serotype-1 for a stratified coordinated surveillance strategy of dengue epidemics

Liqiang Li#, Xiang Guo#, Xiaoqing Zhang, Lingzhai Zhao, Li Li, Yuji Wang, Tian Xie, Qingqing Yin, Qinlong Jing, Tian Hu, Ziyao Li, Rangke Wu, Wei Zhao, Sherman Xuegang Xin, Benyun Shi, Jiming Liu, Shang Xia, Zhiqiang Peng, Zhicong Yang, Fuchun Zhang*, Xiao-Guang Chen*, Xiaohong Zhou*

#These authors contributed equally to this work.

* Correspondence: Xiaohong Zhou (daizhouxh@163.com) ; Xiao-Guang Chen (xgchen2001@hotmail.com) ; Fuchun Zhang (gz8hzfc@126.com)

List of Supplementary Figures, Tables

Preface

1. **Additional file 1 Fig. S Pre. 1** Blueprint for this study.

Section A. Data selection and quality control

2. **Additional file 1 Table S A1** Recombination events of DENV-1 scanned by seven models.
3. **Additional file 1 Table S A2** Summary of 5003 DENV-1 strains eligible for inclusion in this study.

Section B. Establishment of a unified high-resolution global genotyping framework of DENV-1

4. **Additional file 1 Fig. S B1** The viral population numbers and intra-population diversity of DENV-1 inferred using BAPS hierarchical clustering.
5. **Additional file 1 Fig. S B2** Characteristics of MPDs of BAPS raw clusters.
6. **Additional file 1 Fig. S B3** Demography of DENV-1 subgenotypes and clades.
7. **Additional file 1 Table S B1** Summary of characteristics of the designated subgenotypes in genotypes I, IV, and V of DENV-1.
8. **Additional file 1 Table S B2** Summary of characteristics of the designated clades in genotypes I, IV, and V of DENV-1.

Section C. Ancestral state reconstruction and molecular clock phylogenetic analyses

9. **Additional file 1 Table S C1** Characteristics of each designated subgenotype in genotypes I, IV, and V of DENV-1 worldwide.
10. **Additional file 1 Table S C2** Characteristics of each designated clade in genotypes I, IV, and V of DENV-1 worldwide.

Section D. Characterization of the unified global genotyping framework of DENV-1

11. **Additional file 1 Fig. S D1** Trends of global increasing expansion of DENV-1.
12. **Additional file 1 Table S D1** The associations between the substitution rates and tMRCA of the included clades evaluated by the linear regression model after adjustment of the strains number, MPDs and Simpson Index.

Section E. Global geographic distribution and epidemic regional classification

13. **Additional file 1 Fig. S E1** Global geographical distributions of genotypes I, IV, and V of DENV-1 by countries/areas.
14. **Additional file 1 Fig. S E2** Global spreading of DENV-1 genotypes I, IV, and V clades by countries/areas from 1944 to 2017 counted by every ten years.

15. **Additional file 1 Fig. S E3** Geographical hierarchical clustering of DENV-1 at the genotype and subgenotype levels.

16. **Additional file 1 Fig. S E4** Heatmap of defined clade distribution displayed by 12 epidemic regions by percentages.

17. **Additional file 1 Fig. S E5** Global geographical distributions of genotypes I, IV, and V of DENV-1 represent by 12 classified epidemic regions.

18. **Additional file 1 Fig. S E6** Heatmap of the defined clade distribution displayed by countries/areas by percentage.

19. **Additional file 1 Fig. S E7** Relatively specific circulation patterns of subgenotypes and clades kept relative steady in each epidemic region.

20. **Additional file 1 Table S E1** Compositions of countries/areas in the defined 12 epidemic regions of DENV-1.

Section F. Global transmission pattern of DENV-1 at subgenotype level

21. **Additional file 1 Fig. S F1** Estimates of 29 DENV-1 subgenotypes involving the transmission pairs inferred by BSSVS.

22. **Additional file 1 Fig. S F2** DENV-1 transmission pairs in each epidemic country/area within and without its belonging epidemic regions inferred by BSSVS at the subgenotype level.

23. **Additional file 1 Table S F1** The transmission pairs with involved subgenotypes ≧3.

24. **Additional file 1 Table S F2** The transmission pairs inferred by BSSVS from 29 DENV-1 subgenotypes .

Section G. Sources and trend of DENV-1 diffusion at clade level

25. **Additional file 1 Fig. S G1** Dynamic changes of trunks representing the proportion of PACT-inferred dominant epidemic countries/areas for each DENV-1 clade circulation.

26. **Additional file 1 Fig. S G2** Temporal dynamics of clades in 28 PACT-inferred dominant countries/areas.

27. **Additional file 1 Table S G1** The status of 28 PACT-inferred dominant epidemic countries/areas and the clades of DENV-1 involved.

Preface

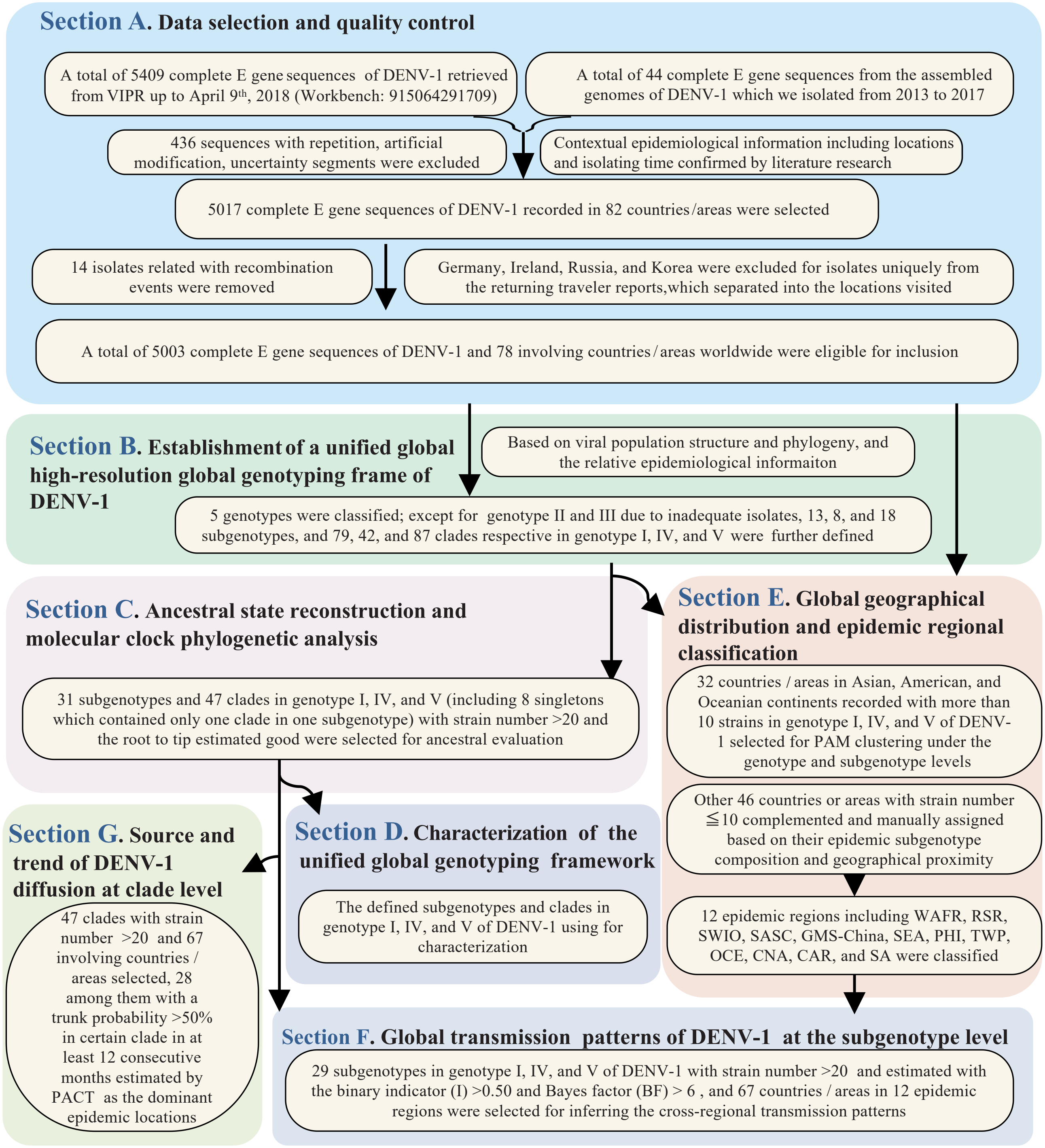

Additional file 1 Fig. S Pre.1 Blueprint for this study

A. Data selection and quality control

Additional file 1 Table S A1 Recombination events of DENV-1 scanned by seven models

Event	Recombination events	Major parent	Minor parent	Detection method						
				R	G	B	M	C	S	T
1	1997|FJ196847|China	2004|EU448386|Thailand	2007|EU448409|Indonesia	+	+	+	+	+	+	+
2	1999|FJ196848|China	1997|AY376737|China	2007|EU448409|Indonesia	+	+	-	+	+	+	+
3	2013|KF184975|Anogla	2013|KU570098|China	2010|JN029812|China	+	-	+	+	+	+	+
4	2010|JN029812|China	2014|KY038891|China	2004|EU448410|China	+	+	+	+	+	+	+
5	1994|AY780642|Puerto_Rico	2000|AY277663|Argentina	Unknown	+	+	+	+	+	-	+
6	2006|EU179861|Brunei	2013|KY818128|Philippine	2014|KT239246|Pakistan	+	+	+	+	+	-	+
7	2011|KY882520|Malaysia	2010|JN415491|Indonesia	2006|KF955414|Venezuela	+	+	+	-	-	+	+
8	2014|KY038891|China	2001|DQ265068|Myanmar	Unknown	+	+	+	+	-	+	+
9	2004|EF032589|China	2007|EU448409|Indonesia	2008|KU509257|Thailand	+	+	+	+	-	-	+
10	1980|D00502|Thailand	1983|AF42561|Australia	2006|KF955414|Venezuela	+	+	+	+	+	+	+
11	1995|AY373427|China	2007|JQ655005|French_Polynesia	1997|AY376737|China	+	+	-	+	+	+	+
12	2015|KX056474|China	Unknown	2013|KR051929|Myanmar	-	-	-	+	+	+	+
13	2005|JF297583|India	2005|EU081258|Singapore	2014|KT239346|Pakistan	+	+	-	-	+	+	+
14	2013|KP903780|Brazil	2013|KP858113|Brazil	Unknown	-	-	-	+	-	-	-

Note: A suite of seven methodologies were implemented in RDP v.4.97 including RDP method, GENECONV, BOOTSCAN, MAXCHI, CHIMAERA, SISCAN, 3SEQ R: RDP; G: GENECONV; B: BootScan; M: MaxChi; C: Chimaera; S: SiScan; T: 3Seq

Additional file 1 Table S A2 Summary of 5003 DENV-1 strains eligible for inclusion in this study

Continent and region	Country/area of isolates (number and name)	Range of isolation dates (year)	Number of isolates[a]
**-Worldwide**	78	1944 - 2017	5003
**Africa**	11	1968 - 2013	42
**WAFR**	3	1968 - 2013	11
	Nigeria	1968	1
	Côte d´lvoire	1985 - 1999	2
	Angola	1988 - 2013	8
**RSR**	4	1988 - 2011	17
	Djibouti	1988	1
	Eritrea	2010	1
	Saudi Arabia	1994 - 2011	10
	Somalia	2011 - 2011	5
**SWIO**	4	1993-2006	14
	Madagascar	2006	1
	Seychelles	2003-2004	3
	Reunion	2004-2004	9
	Comoros	1993	1
**Asia**	19	1956-2017	3487
**SASC**	7	1956-2016	155
	India	1956-2015	73
	Pakistan	2014-2014	10
	Sri Lanka	2004-2014	23
	Bangladesh	2009	1
	Maldives	2011-2016	4
	Bhutan	2013-2013	33
	Nepal	2010-2010	11
**GMS-China**	7	1958-2017	2580
	Viet nam	1998-2016	1165
	Myanmar	1971-2015	343
	Cambodia	1998-2013	105
	Thailand	1958-2015	347
	Laos	1996-2011	91
	China	1979-2017	518
	Japan	2014-2014	11
**SEA**	5	1972-2015	752
	Indonesia	1988-2015	333
	Malaysia	1972-2015	68
	Singapore	1993-2015	297
	Australia	1983-2015	31
	East Timor	2000-2010	23
**Oceania**	19	1944-2016	377
**PHI**	2	1974-2016	129
	Philippines	1974-2016	128
	Palau	2000	1
**TWP**	5	1973-2016	24
	Papua New Guinea	2003-2016	15
	Marshall Is.	2003	1
	Solomon Is.	2002-2002	2
	F.S. of Micronesia	2002-2004	5
	Nauru	1973	1
**OCE**	12	1944-2015	224
	New Caledonia	2001-2012	67

	French Polynesia	1989-2015	102
	Vanuatu	2010	1
	Cook Is.	2002-2007	3
	Fiji	2002-2014	15
	Niue	2012-2102	3
	Tonga	2008-2008	2
	Hawaiian Is.	1944-2001	22
	Wallis and Futuna	2003	1
	Easter Island	2002	1
	Kiribati	2012-2012	4
	Samoa	2001-2001	3
**America**	29	1944-2016	1080
**CNA**	7	1982-2014	385
	Mexico	1982-2013	250
	Costa Rica	1993-2014	21
	Nicaragua	2004-2014	72
	Honduras	2008-2014	4
	United States	1995-2014	29
	Belize	2005-2010	4
	El Salvador	1993-2012	5
**CAR**	19	1944-2016	365
	Barbados	1995-2013	6
	Saint Barthélemy	2016	1
	Peru	1991	1
	Ecuador	2014-2014	6
	Colombia	1985-2015	139
	Virgin Is.	1985	1
	Jamaica	1977-2012	3
	Puerto Rico	1986-2013	92
	Guyana	2008	1
	Haiti	2010-2014	7
	Grenada	1977-1981	9
	Trinidad and Tobago	1978-1986	3
	Suriname	1981-2012	2
	Aruba	1985-2004	3
	Venezuela	1944-2011	83
	French Guiana	1989	1
	Bahamas	1977	1
	Dominica	2010-2010	3
	Martinique	2008-2008	3
**SA**	3	1982-2016	330
	Argentina	1999-2016	130
	Brazil	1982-2014	182
	Paraguay	1999-2011	18
**NA**			17

[a] 5003 DENV-1 isolates including Genotype I: 2952; II:4; III: 2; V:560, and V: 1485 were identified in this study, see details in Fig. 1 and Additional file 1 Section B-D. WAFR, West African Region; RSR, Red Sea Region; SWIO, Southwest Indian Ocean; SASC, South Asia Subcontinent; GMS-China, Great Mekong Subregion-China; SEA, Southeast Asia; PHI, Philippines; TWP, Tropical Western Pacific; OCE, Oceania; CNA, Central North America; CAR, Caribbean; SA, South America. These 12 epidemic regions of DENV-1 were classified in this study, see details in Fig. 2 and Additional file 1 Section E.

B. Establishment of a unified high-resolution global genotyping framework of DENV-1

Our designation of subgenotypes and clades were well supported by BAPS clustered populations and phylogenetic robust bootstrap. Among them, the UFBoot support values of 32 (82%,32/39) designated genotypes and 162 (77.9%, 162/208) clades of DENV-1 genotypes I, IV, and V were higher than 70%, and 30 subgenotypes and 134 clades more than 90% (Table S B1-B2). While the subgenotypes and clades with the UFBoot values lower than 70%, some of them were singletons which containing only one clade or strain-including 4 subgenotypes 1E,1F, 5F and 5I, and 12 clades 1A17, 1M5, 1M6, 4A4, 4A6, 4A7, 4E3, 5F1, 5K18, 5N5, 5N6, and 5P10; some were mainly or uniquely from single country/area such as 1A15 from Somalia, 1D4 and 1E1 from Vietnam, 1G7,1G9, and 1G10 from Myanmar, 1H1 an 1J3 from Thailand, 1M1,1M2,1M9 and 4E8 from Indonesia, 4C2 from Philippines, 5N3 from Venezuela, 5N7 from Brazil, 5N11 from Honduras, 5O3 and 5O4 from Mexico, and 5Q2 from Puerto Rico, and so on (Table S B1-B2). Otherwise, the singletons in subgenotypes (12/41) (Table S C1) and clades (63/208) (Table S C2) in genotype I, IV, and V may suggest that these are DENV-1 population in minority, while otherwise might be partly caused by widely deficient surveillance.

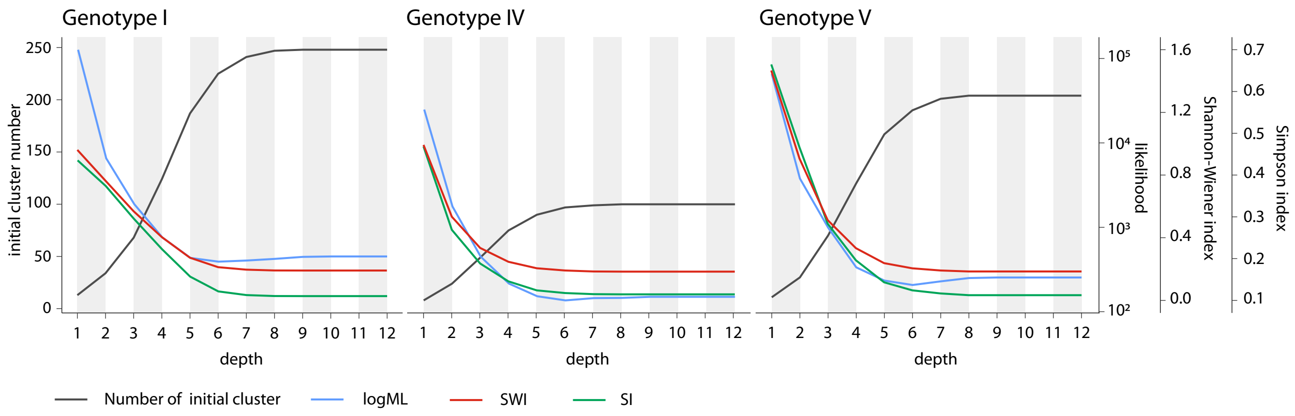

Additional file 1 Fig. S B1 The viral population numbers and intra-population diversity of DENV-1 inferred using BAPS clustering. Three panels represent genotype I (**Left**), IV (**Middle**) and V (**Right**) of DENV-1, respectively. Lines with grey, blue, red, and green colour represent initial cluster number, logML, SWI, and SI, respectively. logML: log maximum likelihood, SWI: Shannon-Wiener index, SI: Simpson index.

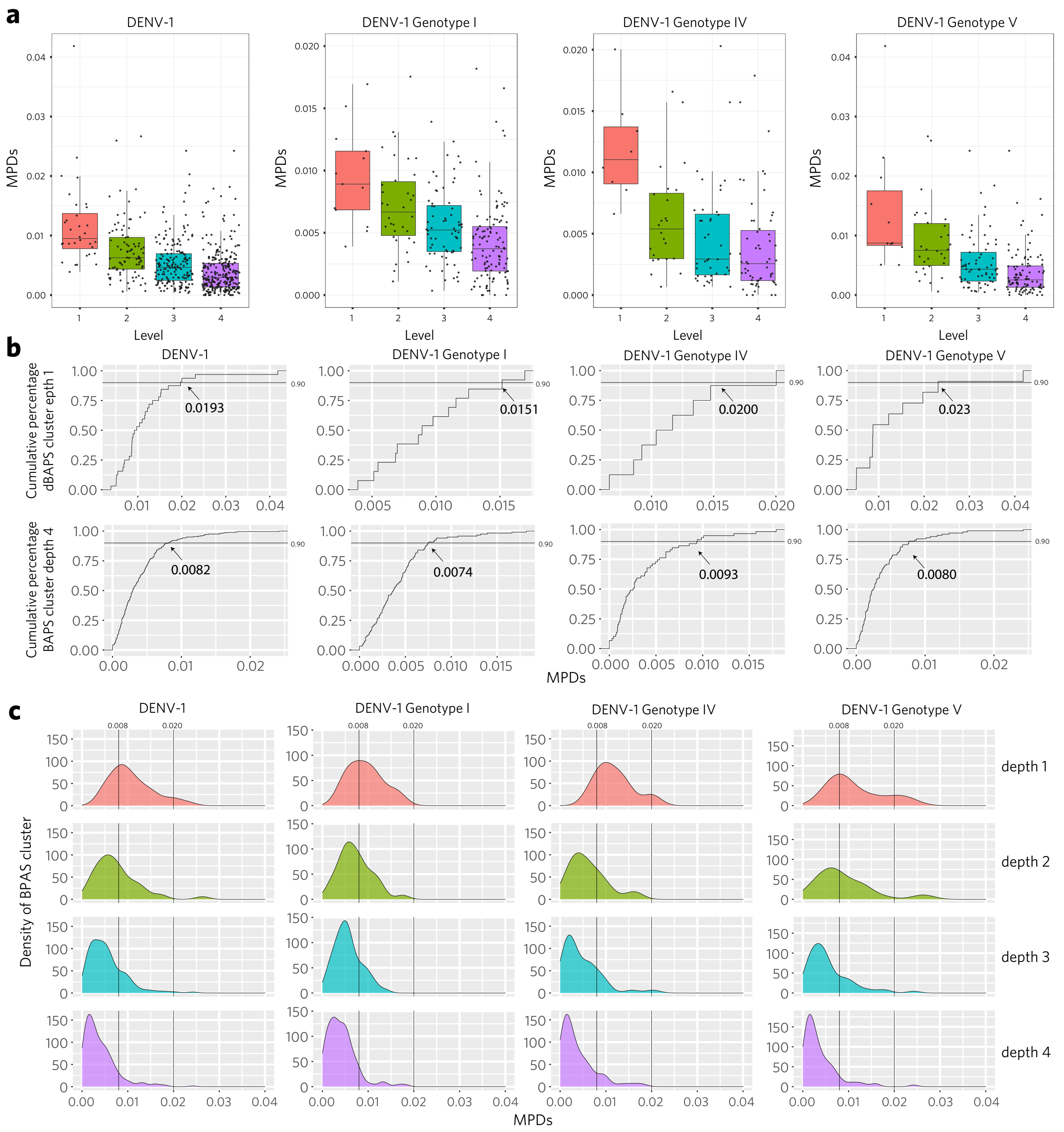

Additional file 1 Fig. S B2 Characteristics of MPDs of BAPS raw clusters. a, MPDs distributions of raw clusters in BAPS hierarchical clustering levels 1-4 for DENV-1 and its genotypes I, IV, and V clustering. **b**, Cumulative distribution plot of MPDs of raw clusters in BAPS levels 1 and 4. MPDs of the majority (90%) of BAPS clusters generated from depth level 1 were below 0.02, and from depth level 4 below 0.008. **c**, Density distributions of MPDs of raw clusters in BAPS levels 1-4 for DENV-1 as well as its genotypes I, IV, and V.

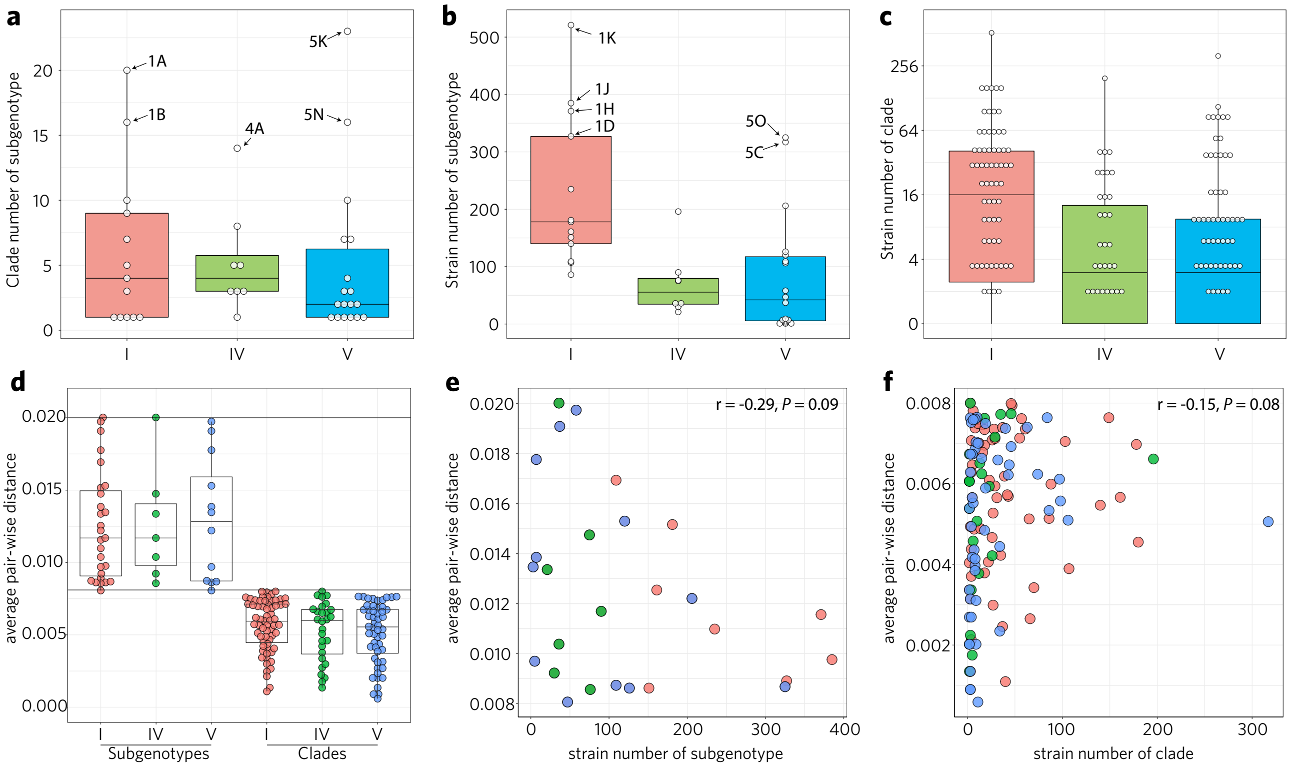

Additional file 1 Fig. S B3 Demography of DENV-1 subgenotypes and clades. a,**b**, Demography of DENV-1 subgenotypes. There were 6.08 ± 4.86, 5.25 ± 2.88, and 4.83 ± 4.31 clades in genotype I, IV, and V, respectively. Five of the subgenotypes represent more abundant clades, 5K with 23, 1A with 20, 1B and 5N with 16, and 4A with 13. There were 227.08 ± 133.11, 70.00 ± 56.88, and 82.50 ± 104.77 strains in each subgenotype in genotype I, IV, and V, respectively. More abundant strains were found in subgenotypes 1E1 (348) and 5C1 (317). **c**, Demography of DENV-1 clades. There were 37.37 ± 38.53, 13.33 ± 15.22, and 17.07 ± 21.76 strains in each clade in genotype I, IV, and V, respectively. **d**, Distribution of the mean pairwise distance (MPDs) within subgenotypes and clades in genotypes I, IV, and V of DENV-1. MPDs within genotypes I, IV, and V were 2.3×10^{-2}, 4.3×10^{-2}, and 3.6×10^{-2}, respectively. **Left panel**: Subgenotypes. MPDs of all 39 subgenotypes were below 1.17×10^{-2}. MPDs of subgenotypes in genotype I, IV, and V were 1.13×10^{-2}, 1.17×10^{-2}, and 1.28×10^{-2}, respectively. Among them, nine subgenotypes with single clade were resulted in lower MPDs than 0.8×10^{-2}, which were not showed in the boxplots. **Right panel**: Clades. MPDs of all 208 clades were below 0.8×10^{-2}. MPDs of clades in genotype I, IV, and V are 0.57×10^{-2}, 0.49×10^{-2}, and $0.51\ \times10^{-2}$, respectively. The values of MPDs in details showed in **Table S B1-B2, C1-C2**. **e-f**, No significant correlation between the designated subgenotypes, clades and their values of MPDs, which were assessed using Spearman's rank correlation coefficient method.

Additional file 1 Table S B1 Summary of characteristics of the designated subgenotypes in genotype I, IV, and V of DENV-1

Genotype	I	IV	V	Total
Subgenotype rank structure				
means of clades in subgenotypes	6.08 ± 4.86	5.25 ± 2.88	4.83 ± 4.31	5.33 ± 4.26
means of strains in subgenotypes	227.08 ± 133.11	70.00 ± 56.88	82.50 ± 104.77	128.13 ± 97.45
BAPS clustering support [a]				
depth 1	13/13 (100%)	8/8 (100%)	9/18 (50%)	30/39 (76.9%)
depth 2	0	0	5/18 (27.8%)	5/39 (15.4%)
depth 3	0	0	2/18 (11.1%)	2/39 (10.3%)
UFBoot values support				
>90	9/13(69.2%)	8/8(100%)	13/18(72.2%)	30/39(76.9%)
70-90	0	0	2/18(11.1%)	2/39(5.1%)
<70	4/13(30.8%)	0	3/18(16.7%)	7/39(17.9%)
Means of MPDs [b]	1.13 [0.86, 1.69]	1.17 [0.86, 2.00]	1.28 [0.81, 1.97]	1.17 [0.81, 2.00]
Geographical diversity				
SWI	0.92 ± 0.51	0.99 ± 0.40	0.97 ± 0.55	0.96 ± 0.49
SI	0.43 ± 0.23	0.47 ± 0.18	0.48 ± 0.21	0.46 ± 0.22

[a] If the DENV-1 strain in a designated subgenotype was exactly consistent with the BAPS cluster at a certain depth, the subgenotype was counted into the BAPS cluster support at that depth. If a subgenotype can be identified at more than one depth in the BAPS cluster, it was then included in the shallowest depth.
[b] Thirteen singletons out of 39 subgenotypes with only one clade were not counted.
MPDs: Mean pairwise distances; SWI: Shannon-Wiener index; SI: Simpson index; UFBoot value: Ultrafast Bootstrap value

Additional file 1 Table S B2 Summary of characteristics of the designated clades in genotype I, IV, and V of DENV-1

Genotype	I	IV	V	Total
Clade rank structure				
means of strains of clades in subgenotypes	37.37 ± 38.53	13.33 ± 15.22	17.07 ± 21.76	24.02 ± 28.36
BAPS clustering support [a]				
depth 1	6/77 (7.8%)	1/42 (2.4%)	1/87 (1.5%)	8/208 (3.8%)
depth 2	14/77 (18.2%)	11/42 (26.2%)	16/87 (18.4%)	41/208 (19.7%)
depth 3	17/77 (22.1%)	9/42 (21.4%)	21/87 (24.1%)	47/208 (22.6%)
depth 4	25/77 (32.5%)	7/42 (16.7%)	18/87 (20.7%)	50/208 (24.0%)
UFBoot values support				
>90	25/79 (31.7%)	32/42 (76.2%)	55/87 (63.2%)	112/208 (53.8%)
70-90	14/79 (17.7%)	1/42 (2.4%)	14/87 (16.1%)	29/208 (13.9%)
<70	19/79 (%)	9/42 (21.4%)	18/87 (20.7%)	46/208 (22.1%)
Means of MPDs [b]	0.59 [0.11, 0.80]	0.57 [0.13, 0.80]	0.55 [0.06, 0.76]	0.57 [0.06, 0.80]
Geographical diversity				
SWI	0.42 ± 0.42	0.32 ± 0.38	0.32 ± 0.40	0.36 ± 0.41
SI	0.22 ± 0.23	0.17 ± 0.20	0.19 ± 0.23	0.20 ± 0.23

[a] If the DENV-1 strain in a designated clade was exactly consistent with the BAPS cluster at a certain depth, the clade was counted into the BAPS cluster support at that depth. If a clade can be identified at more than one depth in the BAPS cluster, it was then included in the shallowest depth.

[b] Sixty-three singletons out of 208 clades with only one strain were not counted.

MPDs: Mean pairwise distances; SWI: Shannon-Wiener index; SI: Simpson index; UFBoot value: Ultrafast Bootstrap value

C. Ancestral state reconstruction and molecular clock phylogenetic analyses

Additional file 1 Table S C1 Characteristics of each designated subgenotype in genotype I, IV, and V of DENV-1 worldwide

Subgenotype	No. of clades	No. of strains	$Year_{first}$	$Year_{latest}$	$PS_{observed}$	MPD	SWI	SI	UFBoot	r	Substitution rate (10^{-4})	tMRCA
1A	20	109	1944	2011	68	0.1694	1.43	0.68	100	0.9	10.66 [8.81, 12.45]	1940.79 [1930.03, 1944.00]
1B	16	181	1990	2011	22	0.0156	1.09	0.51	100	0.72	9.30 [7.76, 10.94]	1980.55 [1972.63, 1985.94]
1C	1	178	2002	2011	10	0.007	0.07	0.02	84	0.83	10.53 [8.72, 12.37]	2001.97 [2001.65, 2002.00]
1D	4	327	1998	2012	15	0.0089	0.43	0.18	100	0.62	9.50 [7.90, 11.11]	1993.67 [1989.20, 1996.50]
1E	1	521	2001	2016	16	0.0068	0.41	0.15	38	0.77	11.96 [10.36, 13.69]	1999.41 [1996.53, 2001.00]
1F	1	140	2003	2013	11	0.0055	1.07	0.58	20	0.63	12.17 [9.66, 15.05]	2002.51 [2000.40, 2003.00]
1G	10	235	1999	2015	17	0.011	0.51	0.26	100	0.66	7.49 [5.82, 9.37]	1993.05 [1988.97, 1995.87]
1H	5	371	2001	2015	15	0.0116	1.92	0.81	66	0.66	12.94 [11.10, 14.83]	1999.25 [1997.46, 2000.36]
1I	1	86	2000	2009	10	0.0051	0.34	0.19	98	0.86	12.71 [9.12, 16.65]	1998.37 [1992.81, 2000.00]
1J	7	385	2002	2015	14	0.0098	1.96	0.83	98	0.76	11.90 [10.31, 13.50]	1999.84 [1997.59, 2001.37]
1K	1	107	2006	2015	10	0.0039	0.58	0.19	98	0.64	14.58 [7.49, 22.57]	2004.14 [1996.14, 2006.00]
1L	3	151	2008	2017	10	0.0086	1.35	0.27	41	0.5	10.37 [7.89, 12.90]	2004.02 [2000.17, 2006.52]
1M	9	161	2005	2015	11	0.0125	0.75	0.41	94	0.7	11.67 [9.25, 14.55]	2003.11 [2000.37, 2004.85]
4A	14	75	1973	2016	44	0.1474	0.7	0.29	97	0.91	9.40 [6.92, 12.05]	1967.48 [1960.82, 1971.71]
4B	3	36	2003	2013	11	0.0104	0.38	0.16	97	0.83	9.77 [6.75, 13.09]	1999.98 [1996.42, 2002.40]
4C	3	76	2007	2016	10	0.0086	0.58	0.26	100	0.43	11.93 [7.25, 16.13]	2006.72 [2004.56, 2007.00]
4D	3	30	1983	2010	28	0.0092	0.76	0.43	100	0.96	11.84 [8.07, 15.91]	1980.63 [1977.12, 1982.78]
4E	8	90	1993	2013	21	0.0117	0.94	0.48	100	0.67	4.44 [3.01, 6.14]	1984.84 [1973.81, 1991.06]
4F	5	36	1988	2011	24	0.02	1.65	0.78	100	0.94	9.98 [6.11, 15.04]	1978.41 [1969.60, 1987.56]
4G	5	21	2002	2016	15	0.0133	1.39	0.67	100	0.96	11.41 [6.30, 17.59]	1999.04 [1993.05, 2001.76]
4H	1	196	1998	2014	17	0.0066	1.53	0.69	99	0.94	9.81 [7.11, 13.17]	1994.95 [1989.70, 1997.99]
5A	3	7	1956	2014	59	0.0178	0.96	0.58	100	/	/	/
5B	7	37	1962	2015	54	0.0191	1.88	0.82	100	0.96	0.03 [91.55, 466.00]	1961.04 [1955.37, 1962.00]
5C	1	317	2004	2016	13	0.0051	1.5	0.72	100	0.61	13.17 [9.94, 16.95]	2000.08 [1994.57, 2003.72]
5K	23	58	1962	2014	53	0.0197	2.59	0.9	99	0.7	5.37 [4.23, 6.51]	1950.38 [1938.01, 1958.20]
5L	3	126	1985	2016	32	0.0086	0.74	0.48	100	0.86	7.49 [5.90, 9.12]	1984.74 [1982.94, 1985.00]
5M	7	47	1987	2003	17	0.0806	1.22	0.62	98	0.71	5.65 [3.59, 7.87]	1980.58 [1968.21, 1986.01]
5N	16	120	1986	2014	29	0.0152	1.72	0.73	100	0.87	7.75 [6.34, 9.23]	1983.70 [1981.07, 1985.26]
5O	4	325	2004	2016	13	0.0087	1.03	0.49	62	0.74	11.28 [9.29, 13.54]	2002.60 [2000.96, 2003.64]
5P	10	206	1994	2016	23	0.0122	1.06	0.52	100	0.72	8.10 [6.64, 9.73]	1991.38 [1986.77, 1994.00]
5Q	2	109	1997	2014	18	0.0087	1.32	0.62	100	0.74	4.74 [3.19, 6.28]	1989.00 [1978.55, 1999.01]
5R	1	106	2009	2016	8	0.0051	0.99	0.57	100	0.74	16.59 [12.10, 21.89]	2008.41 [2007.79, 2008.80]
5D	2	7	1971	1998	28	0.0139	0	0	100	/	/	/
5E	1	1	1968	1968	1	/	0	0	78	/	/	/
5F	1	1	1985	1985	1	/	0	0	34	/	/	/
5G	1	1	1999	1999	1	/	0	0	100	/	/	/
5H	2	5	1970	2014	45	0.0097	0.67	0.48	100	/	/	/
5I	1	9	2013	2013	1	0.002	0.69	0.49	34	/	/	/
5J	2	3	1977	1985	9	0.0135	1.1	0.67	97	/	/	/

$Year_{first}$: the year recorded the first strain of the subgenotype; $Year_{latest}$: the year recorded the latest strain of the subgenotype; PSobserved: $Year_{lates}$-$Year_{first}$; MPD:Mean pairwise distance; SWI:Shannon-Wiener index; SI: Simpson index; UFBoot: $Ultra_{fast}$ Bootstrap value; tMRCA: the most recent common ancestor

Additional file 1 Table S C2 Characteristics of each designated clade in genotype I, IV, and V of DENV-1 worldwide

Clade	No. of strains	$Year_{first}$	$Year_{latest}$	PSobserved	MPD	SWI	SI	UFBoot	r	Substitution rate (10^{-4})	tMRCA
1A7	26	1999	2002	4	0.0047	0	0	100	0.34	4.90 [0, 38.17]	1995.51 [1938.57, 1999.00]
1A12	28	1986	1995	10	0.0074	0.26	0.13	100	0.76	11.33 [7.06, 16.04]	1985.20 [1981.54, 1986.00]
1B8	27	2001	2007	7	0.0053	0.16	0.07	100	0.72	4.16 [0.43, 10.47]	1995.45 [1970.54, 2001.00]
1B9	66	2006	2010	5	0.0027	0.16	0.06	79	0.78	28.86 [14.28, 47.65]	2005.91 [2005.51, 2006.00]
1B12	26	1991	2001	11	0.0071	0.77	0.39	98	0.46	2.89 [0.01, 6.89]	1982.44 [1897.22, 1991.00]
1C1	178	2002	2011	10	0.007	0.07	0.02	84	0.83	10.53 [8.72, 12.37]	2001.97 [2001.65, 2002.00]
1D1	29	1999	2008	10	0.0059	1.09	0.56	100	0.96	12.68 [8.34, 17.50]	1998.72 [1997.49, 1999.00]
1D2	61	2006	2012	7	0.0074	0	0	84	0.56	8.31 [5.30, 11.94]	2000.50 [1995.54, 2003.79]
1D3	57	2002	2011	10	0.0076	0.24	0.1	84	0.75	13.44 [9.21, 18.23]	2001.40 [1999.28, 2002.00]
1D4	180	1998	2009	12	0.0046	0.07	0.02	50	0.39	8.68 [6.38, 10.97]	1997.73 [1996.44, 1998.00]
1E1	521	2001	2016	16	0.0068	0.41	0.15	38	0.77	11.96 [10.36, 13.69]	1999.41 [1996.53, 2001.00]
1F1	140	2003	2013	11	0.0055	10.7	0.58	20	0.63	12.17 [9.66, 15.05]	2002.51 [2000.40, 2003.00]
1G1	31	2001	2008	8	0.0057	0.53	0.35	100	0.62	7.54 [0.19, 16.17]	1996.18 [1981.35, 2000.61]
1G9	47	2000	2002	3	0.0079	0	0	44	0.55	33.14 [21.47, 48.68]	1999.38 [1998.73, 1999.79]
1G10	88	2001	2002	2	0.006	0	0	59	-0.11	1.12 [0, 4.33]	1969.15 [1623.35, 1998.36]
1H1	55	2001	2011	11	0.0071	1.02	0.52	66	0.82	9.48 [7.23, 11.95]	1998.88 [1996.51, 2000.31]
1H2	27	2005	2008	4	0.003	0.32	0.14	100	0.79	14.84 [4.40, 29.25]	2003.97 [1997.86, 2005.00]
1H3	37	2007	2011	5	0.0025	0.61	0.29	75	0.65	8.08 [2.80, 14.43]	2004.98[1999.13, 2007.00]
1H4	103	2006	2015	10	0.007	1.05	0.62	100	0.81	12.20 [8.77, 16.00]	2004.88 [2003.28, 2005.70]
1H5	149	2006	2015	10	0.0076	1.64	0.73	100	0.46	15.01 [11.94, 18.65]	2005.65 [2004.77, 2005.99]
1I1	86	2000	2009	10	0.0051	0.34	0.19	98	0.86	12.71 [9.12, 16.65]	1998.37 [1992.81, 2000.00]
1J1	29	2004	2010	7	0.0071	1.5	0.72	98	0.83	12.06 [2.69, 22.12]	2001.78 [1999.15, 2003.54]
1J2	22	2007	2013	7	0.0041	1.05	0.54	100	0.78	9.01 [4.85, 13.97]	2006.69 [2004.84, 2006.99]
1J3	23	2007	2014	8	0.0061	1.11	0.54	39	0.85	12.27 [6.76, 18.56]	2006.70 [2005.13, 2007.00]
1J4	37	2005	2014	10	0.0074	1.34	0.71	15	0.72	18.81 [13.00, 25.51]	2004.64 [2003.39, 2005.00]
1J5	43	2008	2013	6	0.0057	0.72	0.42	100	0.92	9.96 [3.12, 17.87]	2004.46 [1993.90, 2007.80]
1J6	161	2002	2015	14	0.0057	1.35	0.62	93	0.82	10.84 [8.57, 13.36]	1999.70 [1996.88, 2001.29]
1J7	70	2010	2015	6	0.0034	1.67	0.77	53	0.5	8.37 [5.57, 11.60]	2008.11 [2005.20, 2009.99]
1K1	107	2006	2015	10	0.0039	0.58	0.27	98	0.64	14.58 [7.49, 22.57]	2004.14 [1996.14, 2006.00]
1L1	46	2008	2017	10	0.008	1.35	0.67	100	0.56	9.50 [6.31, 13.29]	2003.58 [1998.97, 2006.88]
1L2	65	2008	2016	9	0.0051	1.36	0.72	41	0.32	10.21 [6.81, 13.90]	2005.59 [2003.43, 2007.05]
1L3	40	2013	2015	3	0.0011	0.85	0.49	97	0.03	7.29 [0, 22.10]	2012.02 [1895.71, 2013.00]
1M1	39	2005	2015	11	0.0062	0.32	0.14	28	0.91	11.45 [8.05, 15.87]	2004.41 [2003.30, 2004.94]
1M8	35	2008	2015	8	0.0042	0.62	0.36	50	0.93	13.87 [7.35, 24.57]	2006.47 [2003.95, 2007.94]
1M9	42	2014	2015	2	0.0057	0	0	20	0.27	2.69 [0, 8.69]	1998.01 [1807.39, 2013.31]
1A1	1	1944	1944	1	/	0	0	100	/	/	/
1A2	16	1980	1998	19	0.0075	0.67	0.49	73	/	/	/
1A3	5	1979	1983	5	0.0065	0.67	0.48	100	/	/	/
1A4	8	1989	1994	6	0.0074	0	0	100	/	/	/
1A5	1	1996	1996	1	/	0	0	81	/	/	/
1A6	1	1992	1992	1	/	0	0	77	/	/	/
1A8	4	1998	2001	4	0.0063	0	0	82	/	/	/
1A9	1	1987	1987	1	/	0	0	100	/	/	/
1A10	1	1989	1989	1	/	0	0	100	/	/	/
1A11	3	1985	1990	6	0.0049	0	0	100	/	/	/
1A13	3	2004	2006	3	0.0031	0	0	100	/	/	/
1A14	3	2005	2011	7	0.0013	0	0	96	/	/	/
1A15	3	2011	2011	1	0.008	0	0	67	/	/	/
1A16	1	2011	2011	1	/	0	0	100	/	/	/
1A17	1	2011	2011	1	/	0	0	67	/	/	/
1A18	1	2004	2004	1	/	0	0	100	/	/	/
1A19	1	2010	2010	1	/	0	0	100	/	/	/
1A20	1	1998	1998	1	/	0	0	100	/	/	/
1B1	10	1990	1996	7	0.0075	0.33	0.18	100	/	/	/
1B2	2	1997	1997	1	0.0054	0	0	99	/	/	/
1B3	2	1999	2000	2	0.004	0	0	100	/	/	/
1B4	11	1993	2004	12	0.0076	0	0	99	/	/	/
1B5	2	1997	1998	2	0.0034	0	0	100	/	/	/
1B6	6	2000	2003	4	0.0078	0.87	0.5	85	/	/	/
1B7	5	2001	2004	4	0.0057	0.5	0.32	77	/	/	/
1B10	1	1994	1994	1	/	0	0	100	/	/	/
1B11	1	1998	1998	1	/	0	0	100	/	/	/
1B13	2	2000	2011	12	0.0061	0.69	0.5	95	/	/	/
1B14	1	1998	1998	1	/	0	0	100	/	/	/
1B15	1	1997	1997	1	/	0	0	100	/	/	/
1B16	18	1994	2009	16	0.007	0.93	0.5	94	/	/	/
1G2	12	2014	2015	2	0.007	0.45	0.28	100	/	/	/
1G3	4	2007	2015	9	0.0044	0.56	0.38	100	/	/	/
1G4	4	2011	2015	5	0.0037	0.56	0.38	100	/	/	/

1G5	18	2009	2015	7	0.0073	0.98	0.59	95	/	/	/
1G6	11	2001	2001	1	0.0075	0	0	82	/	/	/
1G7	4	1999	2001	3	0.0071	0	0	18	/	/	/
1G8	16	2001	2001	1	0.0068	0	0	82	/	/	/
1M2	18	2007	2012	6	0.0038	0.21	0.1	17	/	/	/
1M3	7	2009	2014	6	0.0049	1.15	0.61	100	/	/	/
1M4	4	2010	2010	1	0.0021	0	0	86	/	/	/
1M5	1	2015	2015	1	/	0	0	24	/	/	/
1M6	1	2013	2013	1	/	0	0	24	/	/	/
1M7	14	2010	2013	4	0.0049	0.68	0.49	100	/	/	/
4A14	46	2007	2016	10	0.0077	0.21	0.08	100	0.72	10.88 [6.53, 15.64]	2004.43 [1999.93, 2007.00]
4B3	29	2006	2013	8	0.0072	0.45	0.19	100		9.31 [4.83, 13.64]	2002.71 [1996.43, 2005.57]
4C2	23	2011	2014	4	0.0059	0	0	7	0.52	14.27 [5.16, 27.85]	2009.18 [2004.78, 2010.97]
4C3	35	2007	2015	9	0.0077	0.56	0.26	100	0.62	13.31 [8.69, 18.89]	2006.93 [2005.93, 2007.00]
4D3	26	2000	2010	11	0.0042	0.54	0.27	100	0.94	13.46 [6.69, 22.06]	1999.66 [1997.39, 2000.00]
4E4	25	1993	2007	15	0.0065	0.79	0.4	66	0.52	0.85 [0.01, 4.22]	1945.74 [1321.38, 1992.99]
4E8	40	2008	2012	5	0.003	0	0	66	0.64	10.74 [4.13, 17.17]	2006.94 [2001.94, 2007.99]
4H1	196	1998	2014	17	0.0066	1.53	0.69	99	0.94	9.81 [7.11, 13.17]	1994.95 [1989.70, 1997.99]
4A1	1	1974	1974	1	/	0	0	100	/	/	/
4A2	2	1973	1984	12	0.0054	0.69	0.5	100	/	/	/
4A3	2	1991	1991	1	0.002	0	0	100	/	/	/
4A4	1	1995	1995	1	/	0	0	45	/	/	/
4A5	1	2000	2000	1	/	0	0	97	/	/	/
4A6	1	2002	2002	1	/	0	0	56	/	/	/
4A7	1	2002	2002	1	/	0	0	56	/	/	/
4A8	1	2001	2001	1	/	0	0	100	/	/	/
4A9	2	1999	1999	1	0.0067	0	0	100	/	/	/
4A10	13	2001	2005	5	0.0065	1.16	0.63	94	/	/	/
4A11	1	2002	2002	1	/	0	0	90	/	/	/
4A12	1	2008	2008	1	/	0	0	99	/	/	/
4A13	2	2006	2006	1	/	0	0	100	/	/	/
4B1	3	2003	2009	7	0.008	0	0	100	/	/	/
4B2	4	2012	2013	2	0.0034	0	0	100	/	/	/
4C1	18	2010	2016	7	0.0076	0.85	0.49	11		/	/
4D1	2	1983	1983	1	0.0061	0	0	100	/	/	/
4D2	2	2007	2007	1	0.0061	0	0	100	/	/	/
4E1	9	1998	2011	14	0.0068	0.68	0.37	100	/	/	/
4E2	1	2002	2002	1	/	0	0	100	/	/	/
4E3	1	2010	2010	1	/	0	0	66	/	/	/
4E5	4	2002	2005	4	0.0046	0	0	81	/	/	/
4E6	4	2010	2013	4	0.007	1.04	0.63	65	/	/	/
4E7	6	2008	2010	3	0.0027	0.45	0.28	100	/	/	/
4F1	1	1988	1988	1	/	0	0	100	/	/	/
4F2	2	1988	1988	1	/	0	0	100	/	/	/
4F3	15	2003	2009	7	0.0062	1.49	0.71	90	/	/	/
4F4	12	1995	2004	10	0.0038	0.29	0.15	100	/	/	/
4F5	6	2010	2011	2	0.0046	0.45	0.28	100	/	/	/
4G1	1	2011	2011	1	/	0	0	100	/	/	/
4G2	3	2011	2016	6	0.0022	0	0	100	/	/	/
4G3	2	2015	2015	1	0.0013	0	0	90	/	/	/
4G4	5	2012	2015	4	0.0018	0.5	0.32	100	/	/	/
4G5	10	2002	2004	3	0.0051	1.61	0.76	100	/	/	/
5C1	217	2004	2016	13	0.0051	1.5	0.71	100	0.61	13.17 [9.94, 16.95]	2000.08 [1994.57, 2003.72]
5L2	34	2013	2016	4	0.0023	0.55	0.3	21	0.64	40.80 [11.00, 76.05]	2012.85 [2012.02, 2013.00]
5L3	74	2006	2016	11	0.0062	0.56	0.37	79	0.55	7.47 [5.52, 9.57]	2005.39 [2004.11, 2005.98]
5M7	34	1987	2000	14	0.0044	1.05	0.6	100	0.77	6.98 [4.24, 10.59]	1986.68 [1983.67, 1987.00]
5N7	40	1991	2002	12	0.0074	0	0	43	0.66	8.48 [6.10, 11.08]	1990.03 [1987.43, 1991.00]
5O1	98	2004	2014	11	0.0056	1.18	0.61	62	0.73	10.02 [7.54, 12.68]	2002.63 [2001.28, 2003.74]
5O2	44	2006	2016	11	0.0065	1.33	0.7	100	0.68	6.33 [3.78, 9.23]	2003.37 [1998.23, 2005.93]
5O3	97	2005	2012	8	0.0061	0.26	0.12	31	0.77	12.76 [8.44, 17.78]	2002.31 [1998.27, 2004.53]
5O4	86	2007	2013	7	0.0053	0.15	0.07	64	0.71	11.94 [6.12, 19.15]	2004.26 [1998.55, 2006.73]
5P7	32	2012	2014	3	0.0066	0	0	75	0.21	5.49 [0, 18.63]	2004.53 [1885.30, 2011.96]
5P8	43	1998	2010	13	0.0062	0	0	74	0.51	2.55 [0.55, 5.52]	1986.72 [1953.96, 1998.00]
5P9	84	2004	2016	13	0.0076	1.35	0.7	54	0.9	9.19 [7.04, 11.39]	2002.29 [2000.73, 2003.35]
5Q1	63	1997	2013	17	0.0074	1.57	0.71	100	0.64	3.64 [1.24, 6.98]	1987.81 [1973.82, 1994.98]
5Q2	46	2010	2014	5	0.0069	0.28	0.12	11	0.45	3.55 [0.85, 6.68]	1995.80 [1960.34, 2007.05]
5R	106	2009	2016	8	0.0051	0.99	0.57	100	0.74	16.59 [12.10, 21.88]	2008.41 [2007.79, 2008.80]
5A1	1	1956	1956	1	/	0	0	100	/	/	/
5A2	5	2005	2014	10	0.0042	0.67	0.48	99	/	/	/
5A3	1	2011	2011	1	/	0	0	99	/	/	/
5B1	1	1993	1993	1	/	0	0	99	/	/	/
5B2	4	1993	1994	2	0.0027	0.56	0.38	98	/	/	/

5B3	3	1982	2014	33	0.0063	0.64	0.44	91	/	/	/
5B4	7	1962	1983	22	0.0044	0.41	0.24	100	/	/	/
5B5	9	2004	2015	12	0.007	1.31	0.72	100	/	/	/
5B6	2	2014	2014	1	0.002	0	0	100	/	/	/
5B7	11	2010	2010	1	0.0006	0	0	100	/	/	/
5D1	3	1971	1976	6	0.0076	0	0	100	/	/	/
5D2	4	1996	1998	3	0.0049	0	0	100	/	/	/
5E1	1	1968	1968	1	/	0	0	78	/	/	/
5F1	1	1985	1985	1	/	0	0	34	/	/	/
5G1	1	1999	1999	1	/	0	0	100	/	/	/
5H1	2	1970	2014	45	0.0027	0.69	0.5	100	/	/	/
5H2	3	1971	2014	44	0.0009	0.54	0.44	100	/	/	/
5I1	9	2013	2013	1	0.002	0.69	0.44	34	/	/	/
5J1	1	1977	1977	1	/	0	0	100	/	/	/
5J2	2	1983	1985	3	0.0054	0.69	0.5	100	/	/	/
5K1	1	1962	1962	1	/	0	0	94	/	/	/
5K2	1	1962	1962	1	/	0	0	98	/	/	/
5K3	1	1963	1963	1	/	0	0	97	/	/	/
5K4	1	1963	1963	1	/	0	0	97	/	/	/
5K5	2	1963	2014	52	0.0034	0.69	0.5	100	/	/	/
5K6	3	1963	2014	52	0.0013	0.65	0.44	100	/	/	/
5K7	2	2004	2004	1	0.002	0	0	100	/	/	/
5K8	1	2005	2005	1	/	0	0	100	/	/	/
5K9	1	2006	2006	1	/	0	0	99	/	/	/
5K10	1	2007	2007	1	/	0	0	99	/	/	/
5K11	1	2014	2014	1	/	0	0	100	/	/	/
5K12	19	1977	2004	28	0.0075	1.68	0.73	98	/	/	/
5K13	3	1982	1984	3	0.0031	0	0	100	/	/	/
5K14	1	1986	1986	1	/	0	0	100	/	/	/
5K15	1	1990	1990	1	/	0	0	100	/	/	/
5K16	1	1984	1984	1	/	0	0	100	/	/	/
5K17	1	1978	1978	1	/	0	0	90	/	/	/
5K18	1	1988	1988	1	/	0	0	7	/	/	/
5K19	1	1993	1993	1	/	0	0	97	/	/	/
5K20	3	1993	1996	4	0.0009	0.64	0.44	98	/	/	/
5K21	3	1994	1995	2	0.0067	0	0	98	/	/	/
5K22	5	1985	1995	11	0.0067	0.5	0.32	100	/	/	/
5K23	4	1989	1995	7	0.0075	1.39	0.75	99	/	/	/
5L1	18	1985	2007	23	0.0048	0.21	0.1	100	/	/	/
5M1	1	1991	1991	1	/	0	0	97	/	/	/
5M2	1	1993	1993	1	/	0	0	97	/	/	/
5M3	1	1995	1995	1	/	0	0	100	/	/	/
5M4	1	1995	1995	1	/	0	0	100	/	/	/
5M5	1	2003	2003	1	/	0	0	100	/	/	/
5M6	8	1987	1998	12	0.0069	0	0	93	/	/	/
5N1	6	1986	1990	5	0.0055	0	0	100	/	/	/
5N2	5	1998	2001	4	0.0057	0.67	0.48	80	/	/	/
5N3	5	1994	2004	11	0.0067	0	0	24	/	/	/
5N4	1	2004	2004	1	/	0	0	80	/	/	/
5N5	1	1998	1998	1	/	0	0	35	/	/	/
5N6	1	2004	2004	1	/	0	0	19	/	/	/
5N8	8	1998	2001	4	0.0076	0	0	75	/	/	/
5N9	8	2000	2002	3	0.004	0.9	0.53	100	/	/	/
5N10	10	1997	2013	17	0.0076	1.09	0.66	59	/	/	/
5N11	9	2008	2012	5	0.0031	0	0	50	/	/	/
5N12	15	2009	2014	6	0.0066	0.88	0.55	57	/	/	/
5N13	8	2011	2013	3	0.0041	0.38	0.22	57	/	/	/
5N14	1	2014	2014	1	/	0	0	77	/	/	/
5N15	1	2014	2014	1	/	0	0	77	/	/	/
5N16	1	1995	1995	1	/	0	0	80	/	/	/
5P1	19	1994	2009	16	0.0059	0.86	0.54	100	/	/	/
5P2	6	2008	2014	7	0.0076	0.64	0.44	92	/	/	/
5P3	11	2008	2015	8	0.007	0	0	75	/	/	/
5P4	1	2012	2012	1	/	0	0	78	/	/	/
5P5	1	2013	2013	1	/	0	0	74	/	/	/
5P6	8	2012	2013	2	0.0038	0	0	75	/	/	/
5P10	1	2013	2013	1	/	0	0	40	/	/	/

Note: Year$_{first}$: the year when the first strain of the subgenotype was recorded; Year$_{latest}$: the year when the latest strain of the subgenotype was recorded; PS$_{observed}$:Year$_{latest}$ − Year$_{first;}$ MPD: Mean pairwise distance; SWI:Shannon-Wiener index; SI: Simpson index; UFBoot: Ultrafast Bootstrap value; tMRCA: the most recent common ancestor.

D. Characterization of the unified global genotyping framework of DENV-1

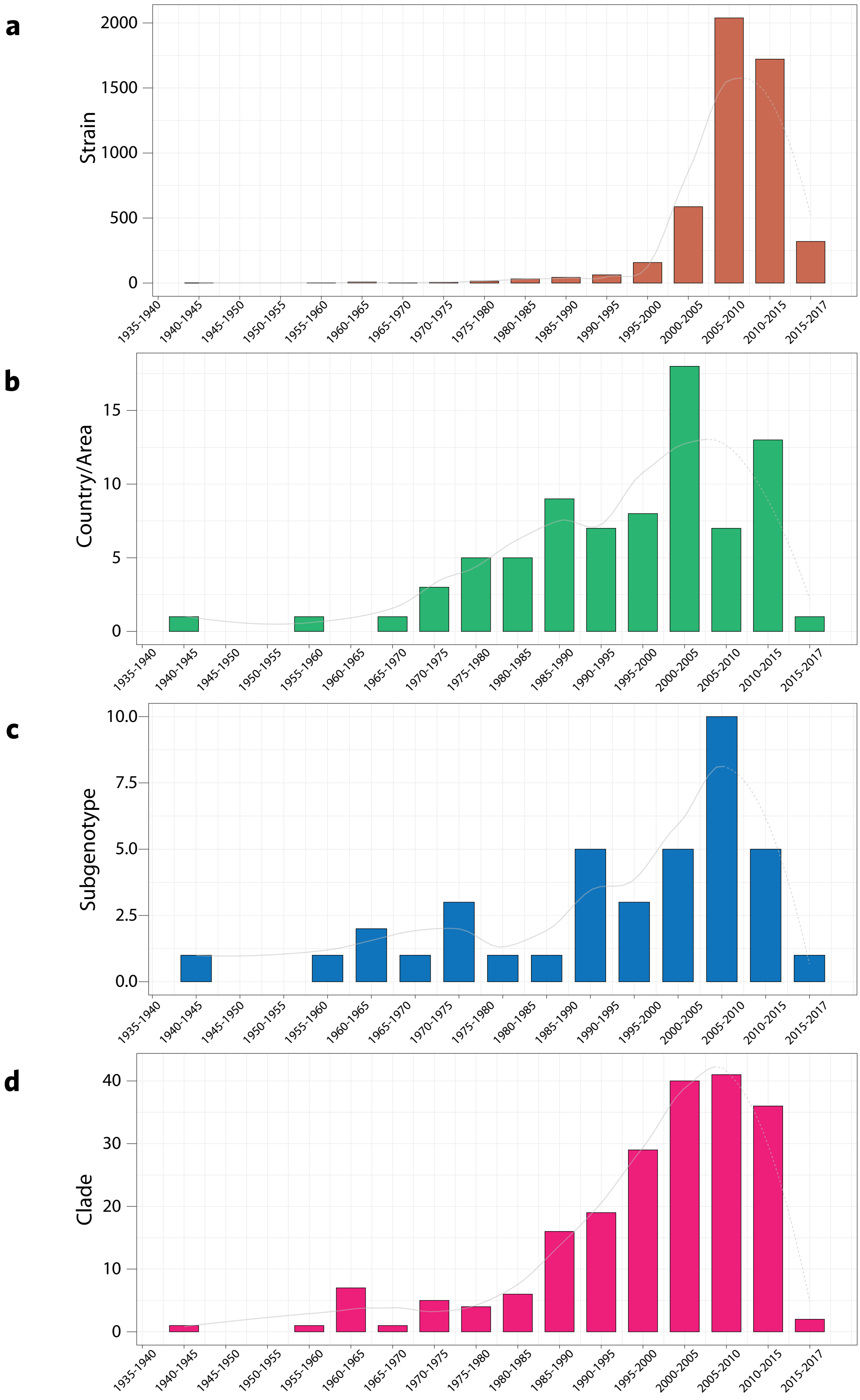

Additional file 1 Fig. S D1 Trends of global increasing expansion of DENV-1. **a**, The new isolated strains of DENV-1, **b**, new reporting DENV-1 isolation countries/areas, **c**, new emerging subgenotypes, **d**, and new emerging clades of DENV-1 were counted each five years.

Additional file 1 Table S D1 The associations between the substitution rates and tMRCAs of the included clades evaluated by the linear regression model after adjustment of the strains number, MPDs and Simpson Index.

Variable	Change with substitution rate	95% CI	*P*
tMRCA	2.9×10^{-5}	$(1.3 - 4.4\times10^{-5})$	<0.001
Number of strains	5.4×10^{-7}	$(-4.4 - 15.2\times10^{-7})$	0.289
MPDs	1.5×10^{-2}	$(-6.1 - 9.1\times10^{-2})$	0.703
SI	-1.6×10^{-5}	$(-37.8 - 34.6\times10^{-5})$	0.932

tMRCA: the most recent common ancestor; MPDs: Mean pairwise distance; SI: Simpson index; CI: confidence interval

E: Global geographic distribution and epidemic regional classification.

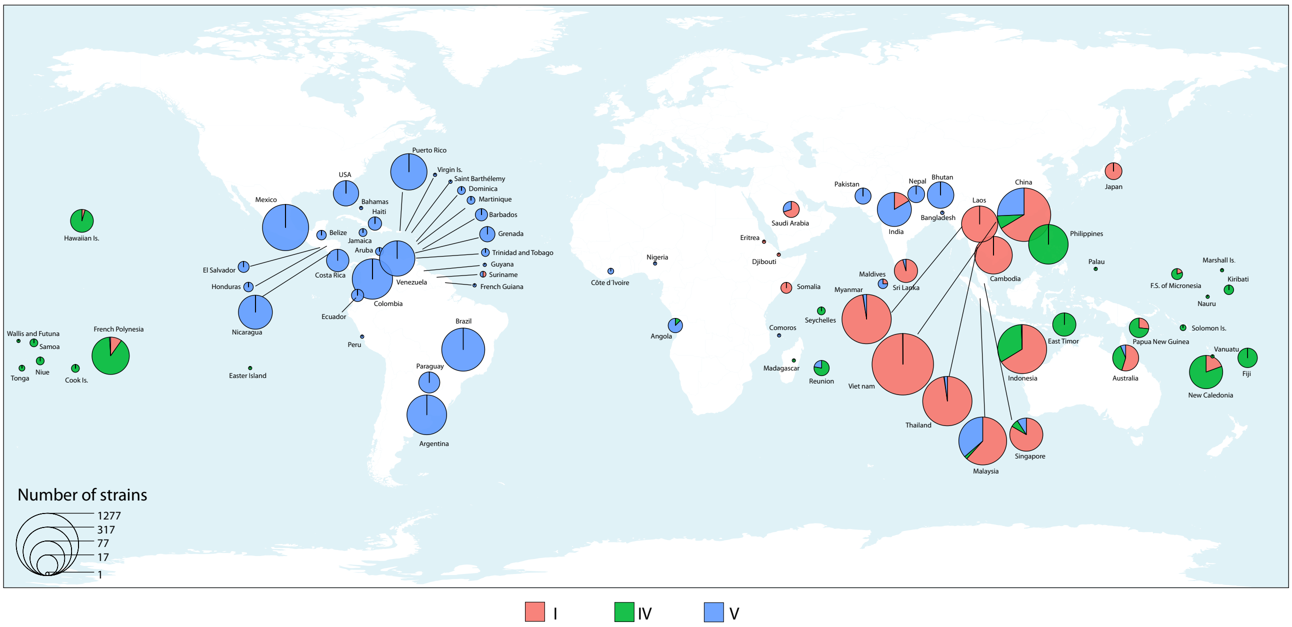

Additional file 1 Fig. S E1 Global geographical distributions of genotype I, IV, and V of DENV-1 by countries/areas. A continental constraint at genotype level was observed, where genotype I was highly restrictively in Asia (98.2%, 2885/2939) especially in East Asia (80.1%, 2374/2939) and Southeast Asia (16.5%, 486/2939), and circulated in Red Sea region in recent years (0.5%,14/2939). Genotype IV was mainly dispersed in island regions near the equator including Oceania (38.3%, 214/559), Malay Archipelago (51.7%, 289/559), and several Southwest Indian Ocean archipelago (2.0%, 11/559). The majority of genotype V was reported from America (72.8%, 1079/1482) and South Asian Subcontinent (8.1%, 120/1482). Meanwhile, genotypes II and III were only recorded in Asia.

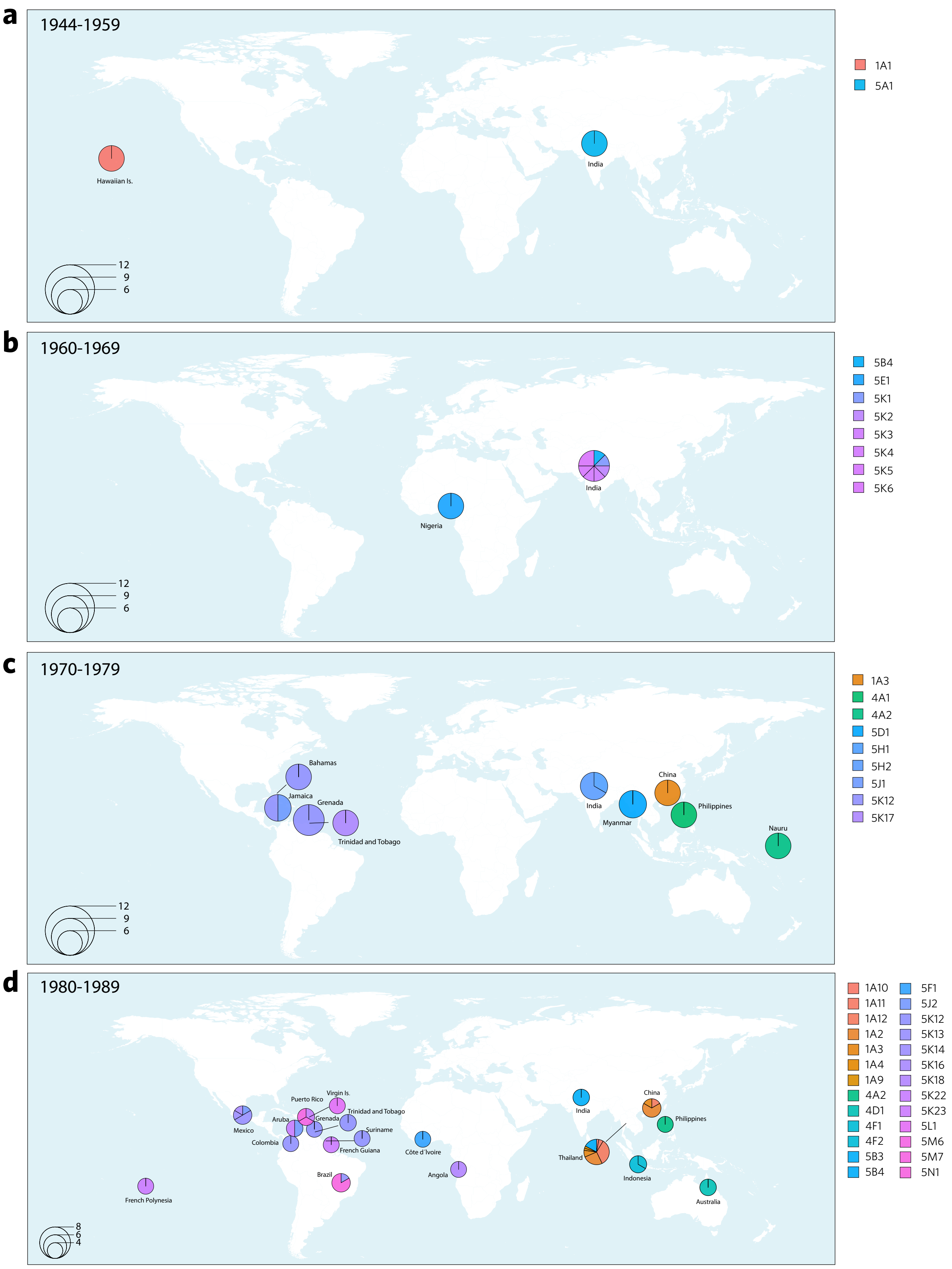
a
1944-1959
Hawaiian Is.
India
12
9
6
1A1
5A1
b
1960-1969
India
Nigeria
12
9
6
5B4
5E1
5K1
5K2
5K3
5K4
5K5
5K6
c
1970-1979
Bahamas
Jamaica
Grenada
Trinidad and Tobago
India
Myanmar
China
Philippines
Nauru
12
9
6
1A3
4A1
4A2
5D1
5H1
5H2
5J1
5K12
5K17
d
1980-1989
Mexico
Puerto Rico
Virgin Is.
Trinidad and Tobago
Aruba
Grenada
Suriname
Colombia
French Guiana
Côte d'Ivoire
Brazil
Angola
French Polynesia
India
China
Philippines
Thailand
Indonesia
Australia
8
6
4
1A10
1A11
1A12
1A2
1A3
1A4
1A9
4A2
4D1
4F1
4F2
5B3
5B4
5F1
5J2
5K12
5K13
5K14
5K16
5K18
5K22
5K23
5L1
5M6
5M7
5N1

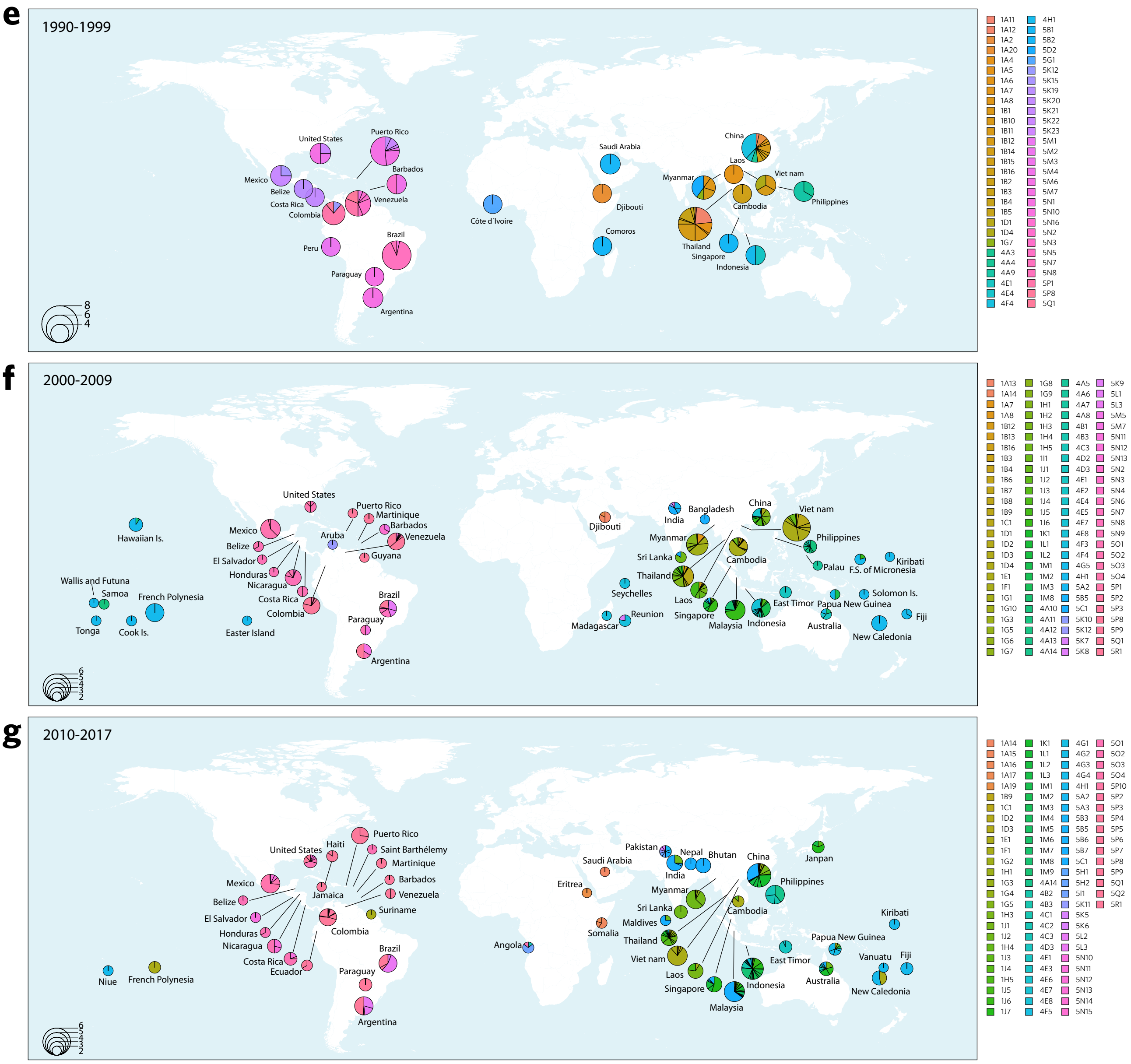
e
1990-1999
United States
Puerto Rico
Mexico
Barbados
Belize
Costa Rica
Venezuela
Colombia
Peru
Brazil
Paraguay
Argentina
Côte d'Ivoire
Saudi Arabia
Djibouti
Comoros
China
Laos
Myanmar
Viet nam
Cambodia
Philippines
Thailand
Singapore
Indonesia
8
6
4
f
2000-2009
United States
Puerto Rico
Martinique
Barbados
Venezuela
Hawaiian Is.
Mexico
Aruba
Belize
El Salvador
Guyana
Honduras
Nicaragua
Costa Rica
Colombia
Wallis and Futuna
Samoa
French Polynesia
Tonga
Cook Is.
Easter Island
Brazil
Paraguay
Argentina
Djibouti
India
Bangladesh
China
Viet nam
Myanmar
Philippines
Sri Lanka
Cambodia
Palau
F.S. of Micronesia
Kiribati
Thailand
Seychelles
Laos
East Timor
Papua New Guinea
Solomon Is.
Singapore
Malaysia
Indonesia
Reunion
Madagascar
Australia
Fiji
New Caledonia
6
5
4
3
2
g
2010-2017
Puerto Rico
Haiti
Saint Barthélemy
United States
Martinique
Barbados
Mexico
Jamaica
Venezuela
Belize
Suriname
El Salvador
Colombia
Honduras
Nicaragua
Costa Rica
Ecuador
Brazil
Paraguay
Argentina
Niue
French Polynesia
Angola
Eritrea
Saudi Arabia
Somalia
Pakistan
Nepal
Bhutan
India
China
Janpan
Myanmar
Philippines
Sri Lanka
Cambodia
Maldives
Thailand
Viet nam
Laos
Singapore
Malaysia
Indonesia
East Timor
Papua New Guinea
Kiribati
Vanuatu
Fiji
Australia
New Caledonia
6
5
4
3
2

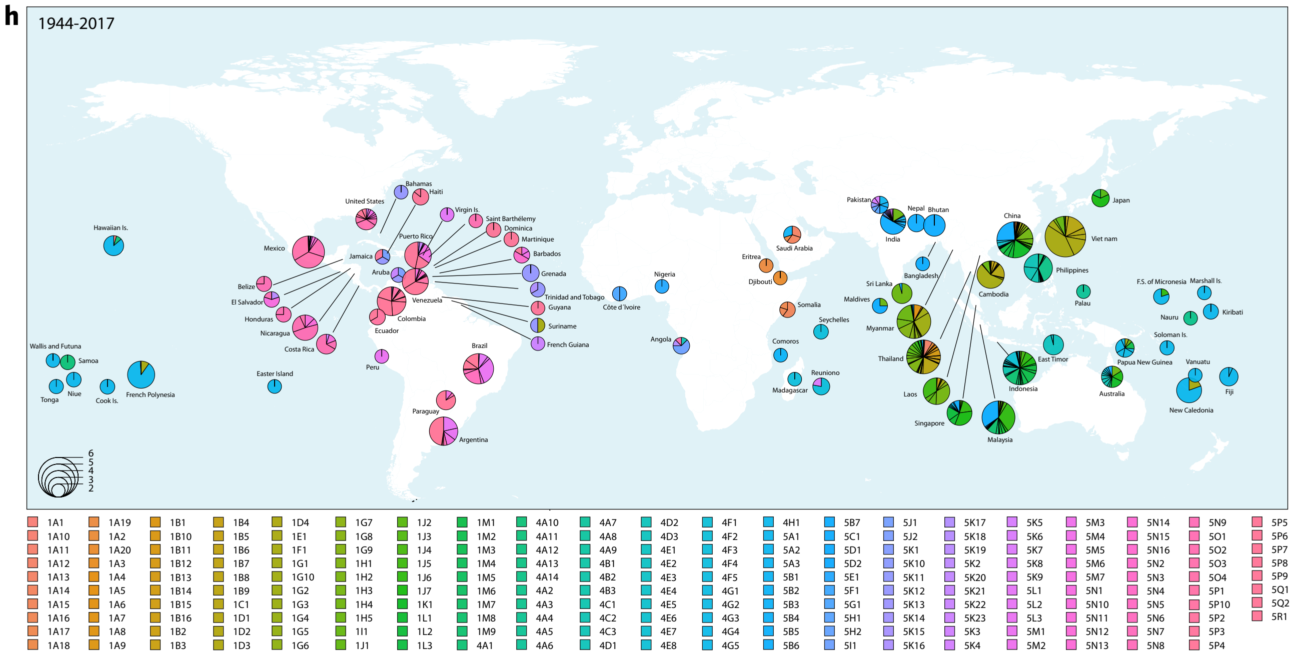

Additional file 1 Fig. S E2 Global spreading of DENV-1 genotype I, IV, and V clades by countries/areas from 1944 to 2017 counted by every ten years. Each panel displayed circulating clades and the corresponding countries in that period. Pies are sized to indicate the number of isolates and the colors in the pies indicate the defined clade. **a**, 1944-1959, **b**, 1960-1969, **c**, 1970-1979, **d**, 1980-1989, **e**, 1990-1999, **f**, 2000-2009, **g**, 2010-2017 **h**, 1944-2017. Before 1970s, DENV-1 isolates were reported in Hawaiian Is. (USA, 1944), Nigeria (Africa,1968), and India (South Asian sub-continent,1956), which indicated that DENV-1 had been epidemic in Oceania, Africa, and Asia continents, and India recorded with 8 defined clades which represented a higher population diversity of DENV-1. After 1970s, DENV-1 showed a rapid expansion, strains were firstly isolated in Jamaica, Grenada, and Bahamas in American continent in 1977, and then 50 countries/areas were instantly affected with 105 and 94 circulating clades of DENV-1 during 2000 to 2009, and 2010-2017, respectively.

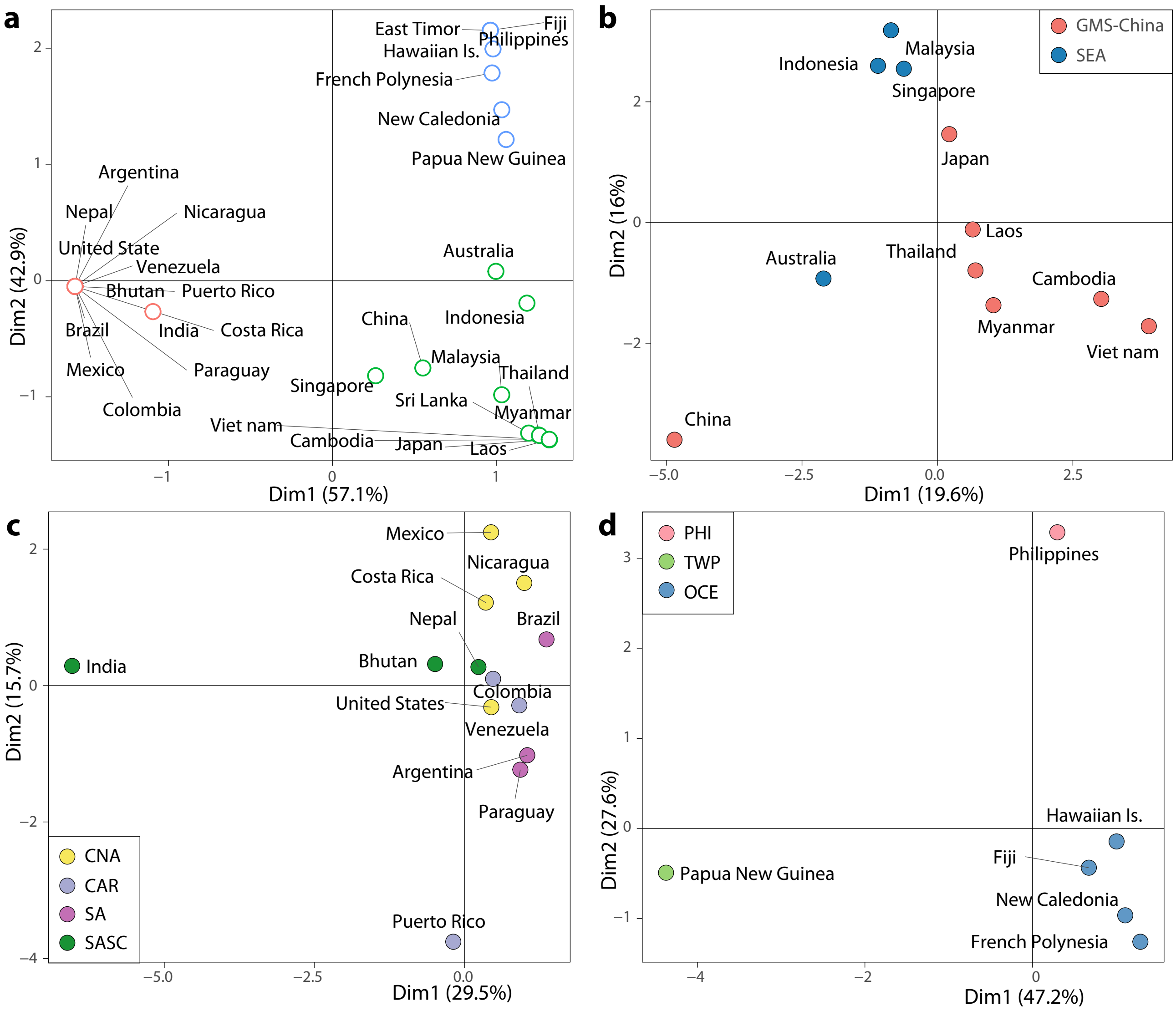

Additional file 1 Fig. S E3 Geographical hierarchical clustering of DENV-1 at the genotype and subgenotype levels. Using partitioning around medoid (PAM), which was implemented in "factoextra" package in R software, 32 countries/areas with more than 10 DENV-1 strains were selected for geographical clustering based on composition and frequencies of genotype or subgenotype. The optimal numbers of clusters were assessed and chosen using silhouette method. **a**, the preliminary three geographical clustering corresponding three genotypes were evidently continentally constraint consistent with the previous studies. According this continental separation, countries/areas within in Asia (**b**), America (**c**) and pacific islands (**d**) were further clustered at subgenotype level for high resolution of DENV-1 circulation pattern.

Additional file 1 Table S E1 Compositions of countries/areas in the defined 12 epidemic regions of DENV-1

Epidemic region	Abbreviation of region	Countries/areas included	Number of included countries/ areas
West African Region	WAFR	Angola, Côte d´lvoire, Nigeria	3
Red Sea Region	RSR	Djibouti, Eritrea, Saudi Arabia, Somalia	4
Southwest Indian Ocean	SWIO	Comoros, Madagascar, Reunion, Seychelles	4
South Asia Subcontinent	SASC	Bangladesh, Bhutan, India, Maldives, Nepal, Pakistan, Sri Lanka	7
Great Mekong Subregion-China	GMS-China	Cambodia, China, Japan, Laos, Myanmar, Thailand, Viet nam	7
Southeast Asia	SEA	Australia, East Timor, Indonesia, Malaysia, Singapore	5
Philippines	PHI	Palau, Philippines	2
Tropical Western Pacific	TWP	Marshall Is., F.S. of Micronesia, Nauru, Papua New Guinea, Solomon Is.	5
Oceania	OCE	Cook Is., Easter Island, Fiji, French Polynesia, Kiribati, New Caledonia, Niue, Samoa, Tonga, Hawaiian Is., Vanuatu, Wallis and Futuna	12
Central North America	CNA	Belize, Costa Rica, Honduras, El Salvador, Mexico, Nicaragua, United States	7
Caribbean	CAR	Aruba, Bahamas, Barbados, Colombia, Dominica, Ecuador, French Guiana, Grenada, Guyana, Haiti, Jamaica, Martinique, Peru, Puerto Rico, Saint Barthélemy, Suriname, Trinidad and Tobago, Venezuela, Virgin Is.	19
South America	SA	Argentina, Brazil, Paraguay	3
Worldwide			78

Note: Based on the first geographical clustering in Fig. S-E3, the detailed information involving epidemiology, geographical proximity, and the main epidemic subgenotype composition were thus complemented in further geographical classification. For example, due to lack of understanding of dengue epidemiologically and the probability of underreporting in African continent, where no African countries recorded more than 10 isolates of DENV-1 except only one strain reported respectively in Djibouti, Eritrea, Nigeria, Madagascar, and Comoros, but all the African countries involved and those with similar rare records of DENV-1 on other continents (46 in total) are still of high concern and then were included for the analyses of global geographical distribution.

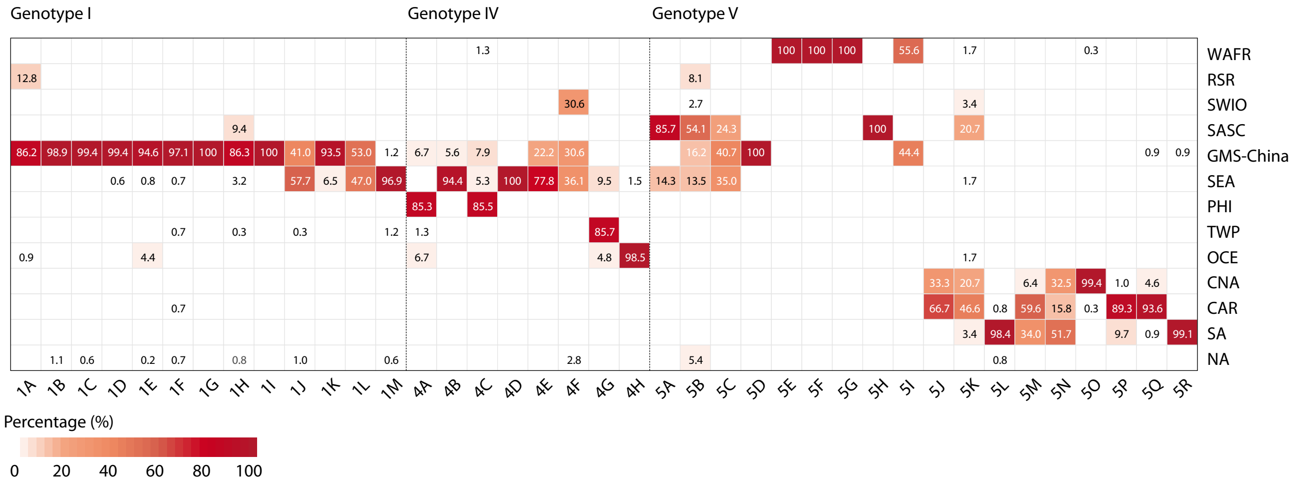

Additional file 1 Fig. S E4 Heatmap of defined clade distribution displayed by 12 epidemic regions by percentages. For certain clade, the distribution in all reported countries/areas equals 100%; the percentages in a specific region showed in the corresponding cell. Subgenotype 5K was found spreading across SEA, SASC, SA, CAR, CNA, OCE, SWIO, and WAFR, while 5B across SEA, GMS-China, SASC, SWIO, and RSR; 4F across SEA, GMS-China, and SWIO; 5C across GMS-China, SEA, and SASC; 5M and 5N across SA, CAR, and CNA. WAFR, West African Region; RSR, Red Sea Region; SWIO, Southwest Indian Ocean; SASC, South Asia Subcontinent; GMS-China, Great Mekong Subregion-China; SEA, Southeast Asia; PHI, Philippines; TWP, Tropical Western Pacific; OCE, Oceania; CNA, Central North America; CAR, Caribbean; SA, South America.

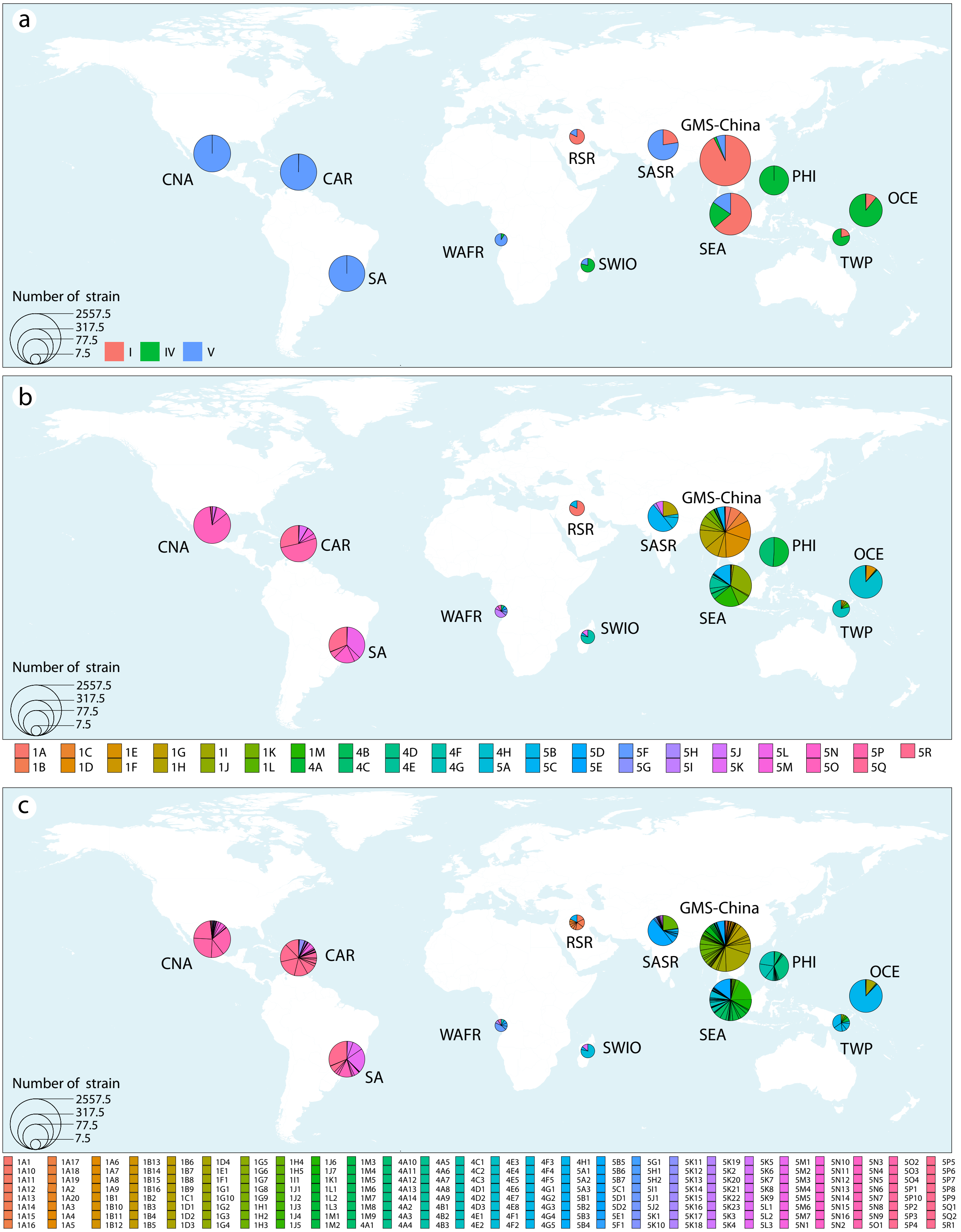

Additional file 1 Fig. S E5 Global geographical distributions of genotypes I, IV, and V of DENV-1 represent by 12 classified epidemic regions. Pies are sized to indicate the number of isolates and colors in the pies indicate genotype (**a**), subgenotype (**b**), and clade (**c**) compositions, respectively. Apparently, Genotypes I, IV and V presented in SEA and GMS-China, while I and IV co-circulated in SASR and RSR, I and V in OCE and TWP. WAFR, West African Region; RSR, Red Sea Region; SWIO, Southwest Indian Ocean; SASC, South Asia Subcontinent; GMS-China, Great Mekong Subregion-China; SEA, Southeast Asia; PHI, Philippines; TWP, Tropical Western Pacific; OCE, Oceania; CNA, Central North America; CAR, Caribbean; SA, South America.

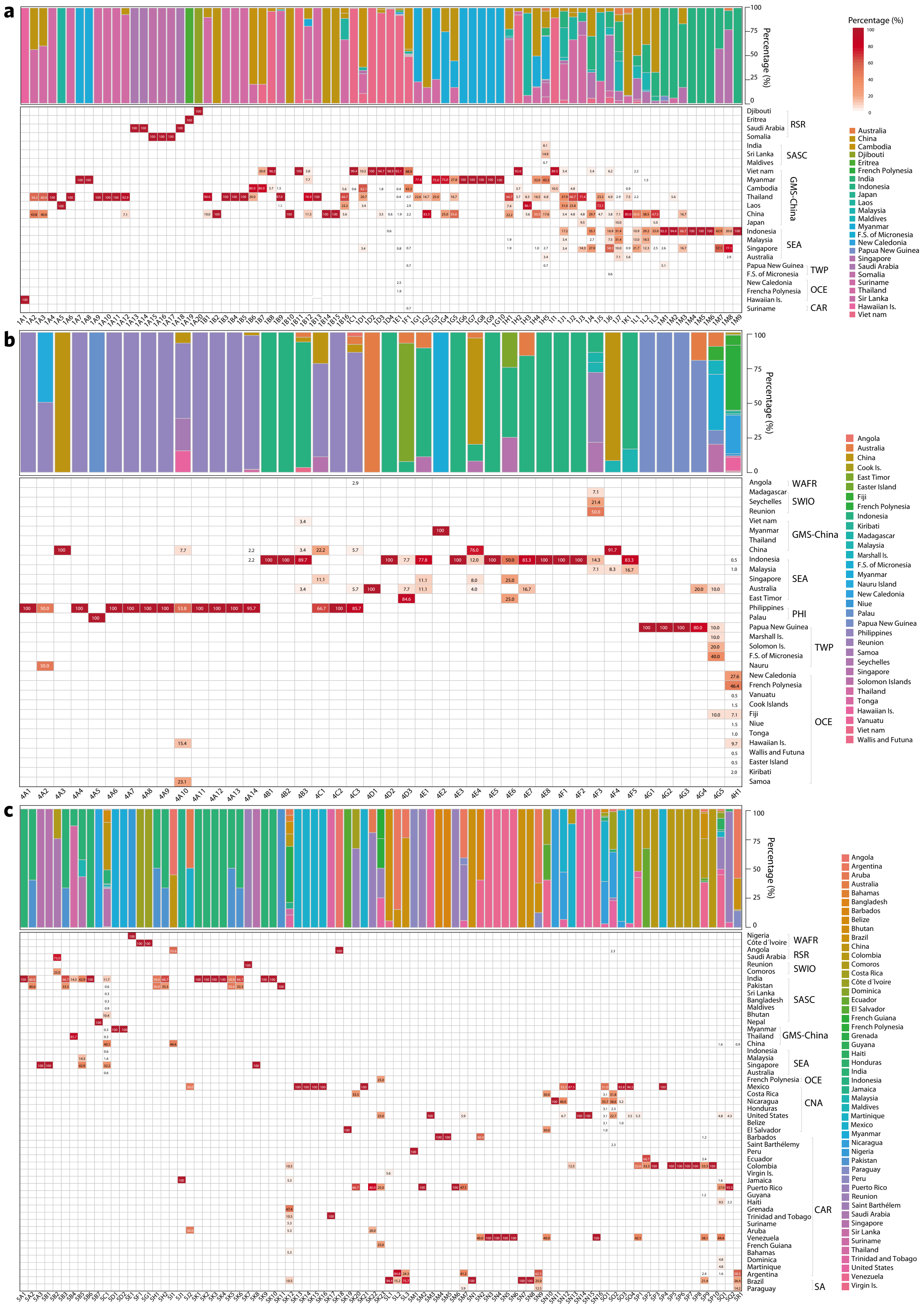

Additional file 1 Fig. S E6 Heatmap of the defined clade distribution displayed by countries/areas by percentage. For a certain clade, the distribution in all reported countries/areas equals 100%; the percentage in each country/area displayed in the corresponding cell.The stacked barplot of proportion in each clade is colored by countries/areas in the panel above. **a**, Clades in genotype I of DENV-1, **b**, Clades in genotype IV of DENV-1, **c**, Clades in genotype I of DENV-1.

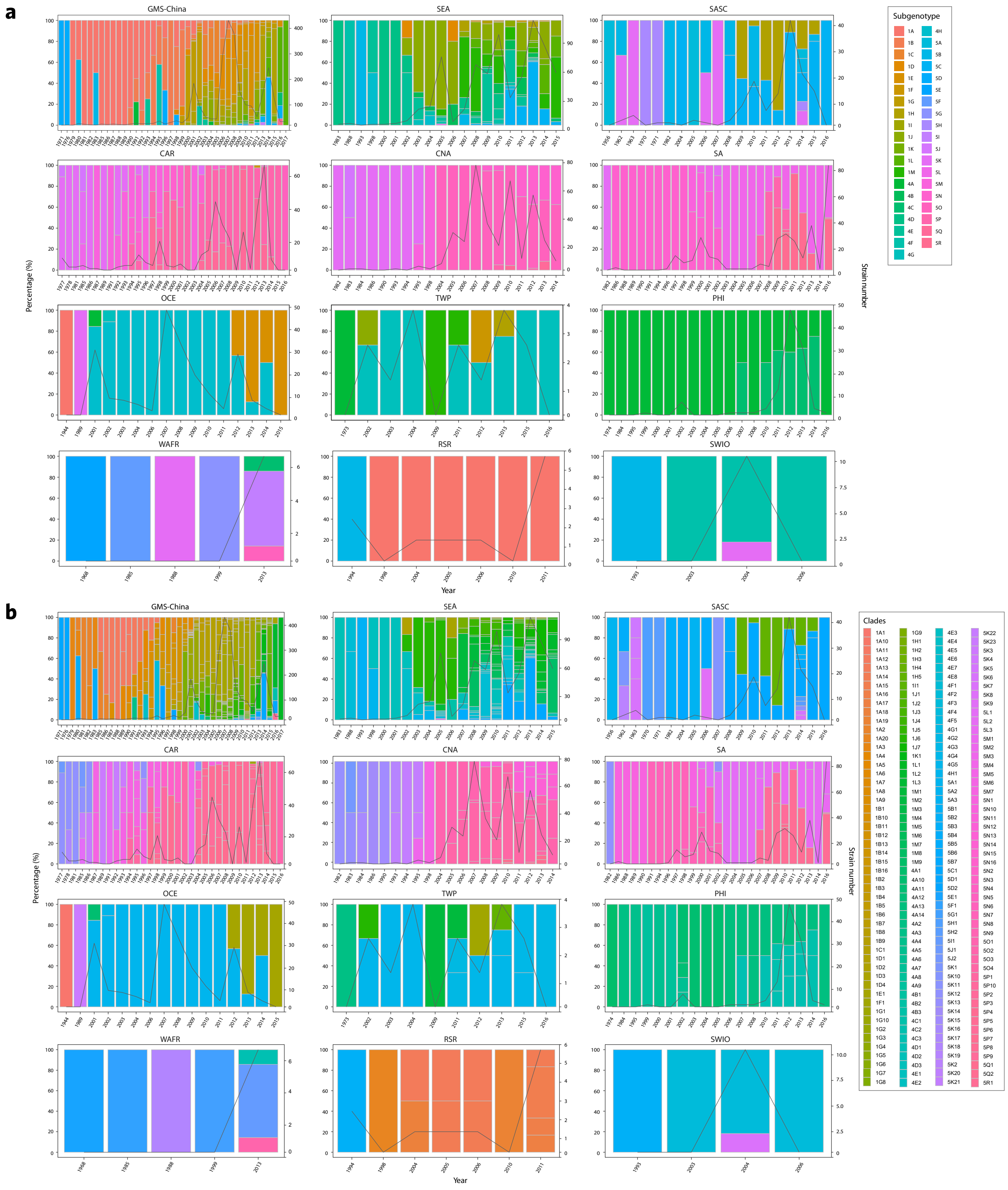

Additional file 1 Fig. S E7 Relatively specific circulation patterns of subgenotypes and clades in each epidemic region. Stacked barplots of proportion are colored by subgenotypes (**a**) and clades (**b**) along time in each epidemic region. WAFR, West African Region; RSR, Red Sea Region; SWIO, Southwest Indian Ocean; SASC, South Asia Subcontinent; GMS-China, Great Mekong Subregion-China; SEA, Southeast Asia; PHI, Philippines; TWP, Tropical Western Pacific; OCE, Oceania; CNA, Central North America; CAR, Caribbean; SA, South America.

F: Global transmission pattern of DENV-1 at subgenotype level

Additional file 1 Table S F1 The transmission pairs with involved subgenotypes ≧3

Transmission pairs	Epidemic regions	Subgenotypes involved	Number
China - Thailand	GMS_China	1A, 1B, 1G, 1J, 1K	5
China - Viet nam	GMS_China	1B, 1D, 1E, 1H, 1J	5
Argentina - Brazil	SA	5L, 5N, 5P, 5R	4
China - Indonesia	GMS_China & SEA	1L, 1M, 4B, 4E	4
Indonesia - Singapore	SEA	1L, 1M, 4E, 5C	4
Argentina - Paraguay	SA	5M, 5N, 5R	3
Australia - Indonesia	SEA	1J, 4B, 4E	3
Cambodia - Laos	GMS_China	1D, 1F, 1J	3
China - Malaysia	GMS_China & SEA	1J, 1L, 4F	3
China - Myanmar	GMS_China	1G, 1H, 5C	3
China - Singapore	GMS_China & SEA	1K, 4C, 5C	3
Mexico - United States	CNA	5N, 5O, 5P	3
Myanmar - Thailand	GMS_China	1A, 1B, 1G	3
Puerto Rico - United States	CNA & CAR	5K, 5M, 5Q	3
Singapore - Viet nam	SEA & GMS_China	1E, 1J, 1L	3
Thailand - Viet nam	GMS_China	1D, 1E, 1J	3

Additional file 1 Table S F2 The transmission pairs inferred by BSSVS from 29 DENV-1 subgenotypes

Subgenotype	Transmission pair	Bayes Factor	Transmission rate	Epidemic region
1A	China-Thailand	28270.73674	1.8204	GMS-China
1B	China-Thailand	19237.83713	1.6583	GMS-China
1B	Cambodia-Thailand	4807.85613	1.1049	GMS-China
1B	Cambodia-Viet nam	2135.637426	1.0577	GMS-China
1D	Cambodia-Viet nam	19237.83713	1.3507	GMS-China
1D	Cambodia-Laos	425.4174552	1.0307	GMS-China
1E	Cambodia-China	23756.42518	1.4306	GMS-China
1E	China-Viet nam	23756.42518	1.2261	GMS-China
1E	Singapore-Viet nam	23756.42518	1.0332	SEA, GMS-China
1F	Cambodia-China	28270.73674	1.428	GMS-China
1G	Myanmar-Thailand	1025.445395	1.0059	GMS-China
1H	China-Myanmar	50810.93968	3.0279	GMS-China
1J	China-Thailand	15.46323389	1.1519	GMS-China
1J	China-Malaysia	15.47291029	1.1504	GMS-China, SEA
1J	Australia-Indonesia	41798.9843	1.085	SEA
1L	Malaysia-Singapore	23756.42518	2.3752	SEA
1L	China-Malaysia	1128.744434	1.3901	GMS-China, SEA
1L	China-Indonesia	414.1860951	1.0852	GMS-China, SEA
1M	Indonesia-Singapore	16.27392156	1.9377	SEA
4A	China-Philippines	19237.83713	1.4004	GMS-China, PHI
4A	Samoa-Hawaiian Is.	64.43676936	1.1365	OCE
4C	China-Philippines	10176.89698	1.4144	GMS-China, PHI
4C	China-Singapore	15.77625651	1.0513	GMS-China, SEA
4E	Australia-Indonesia	14712.71912	1.3137	SEA
4E	Indonesia-Singapore	7355.542187	1.2961	SEA
4F	Indonesia-Malaysia	130.551943	1.3338	SEA
4H	Fiji-New Caledonia	384.0194727	1.4334	OCE
5C	China-Singapore	46305.43292	2.8196	GMS-China, SEA
5C	China-India	2567.664839	1.6255	GMS-China, SASC
5K	Grenada-Trinidad and Tobago	1015.57413	1.2878	CAR
5K	India-Pakistan	189.4203805	1.062	SASC
5K	Grenada-Puerto Rico	22.51325636	1.0223	CAR
5L	Argentina-Brazil	5621.675595	1.6077	SA
5M	Argentina-Paraguay	14712.71912	1.423	SA
5M	Puerto Rico-United States	3676.95372	1.2028	CAR, CNA
5N	El Salvador-Nicaragua	824.6448084	1.4453	CNA
5N	Argentina-Paraguay	843.4809059	1.0197	SA
5O	Costa Rica-Nicaragua	28270.73674	1.9258	CNA
5O	Costa Rica-United States	212.6899363	1.2179	CNA
5O	Costa Rica-Honduras	178.1016143	1.0957	CNA
5P	Colombia-Venezuela	28270.73674	1.4939	CAR
5Q	Puerto Rico-United States	28270.73674	1.8615	CAR, CNA
5Q	Haiti-Puerto Rico	4709.171796	1.2528	CAR
5R	Argentina-Brazil	5621.675595	1.4287	SA

Notes: Only the pairs with transmission rate ≧ 1 from the 172 pairs totally inferred in the study are showed in this table. BSSVS: the Bayesian stochastic search variable selection; WAFR, West African Region; RSR, Red Sea Region; SWIO, Southwest Indian Ocean; SASC, South Asia Subcontinent; GMS-China, Great Mekong Subregion-China; SEA, Southeast Asia; PHI, Philippines; TWP, Tropical Western Pacific; OCE, Oceania; CNA, Central North America; CAR, Caribbean; SA, South America.

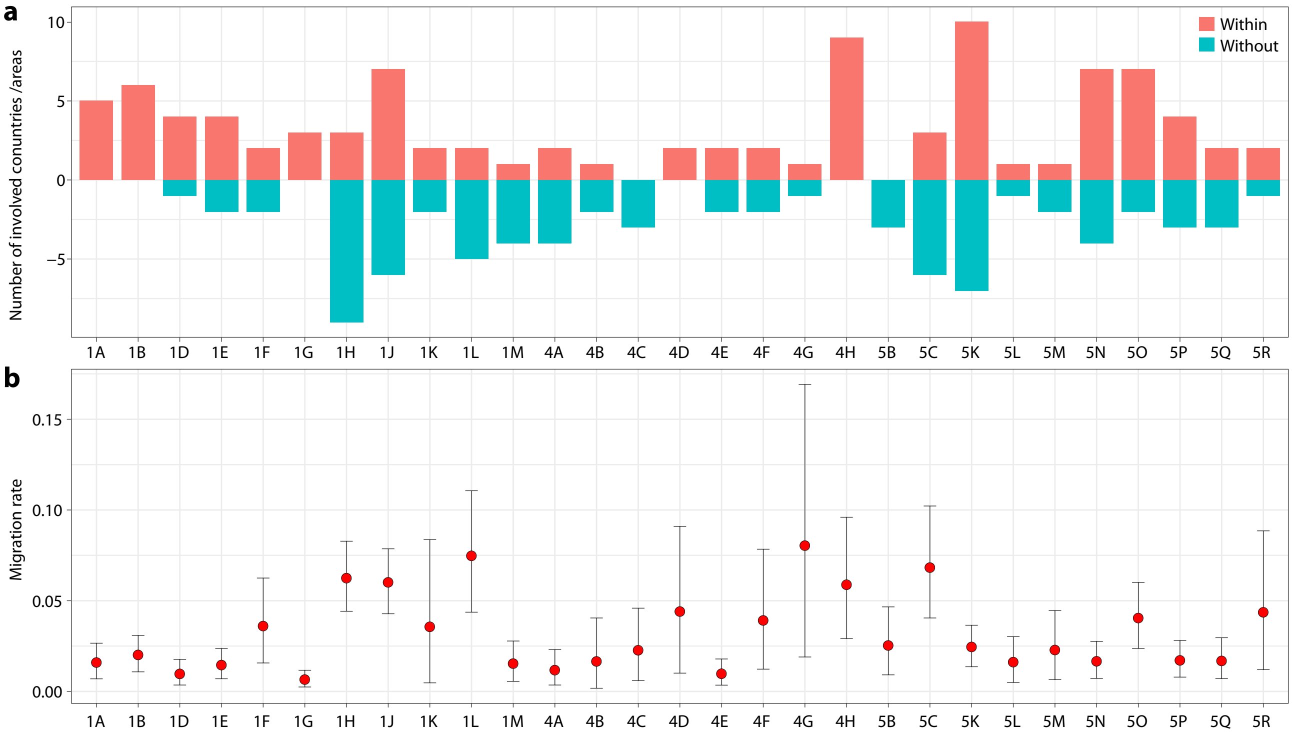

Additional file 1 Fig. S F1 Estimates of 29 DENV-1 subgenotypes involving the transmission pairs inferred by BSSVS. a, Numbers of countries/areas involving the transmission pairs of each subgenotype. b, Estimates of migration rate in each included subgenotype. The red points and error bars show the median and the 95% Bayesian credible intervals, respectively.

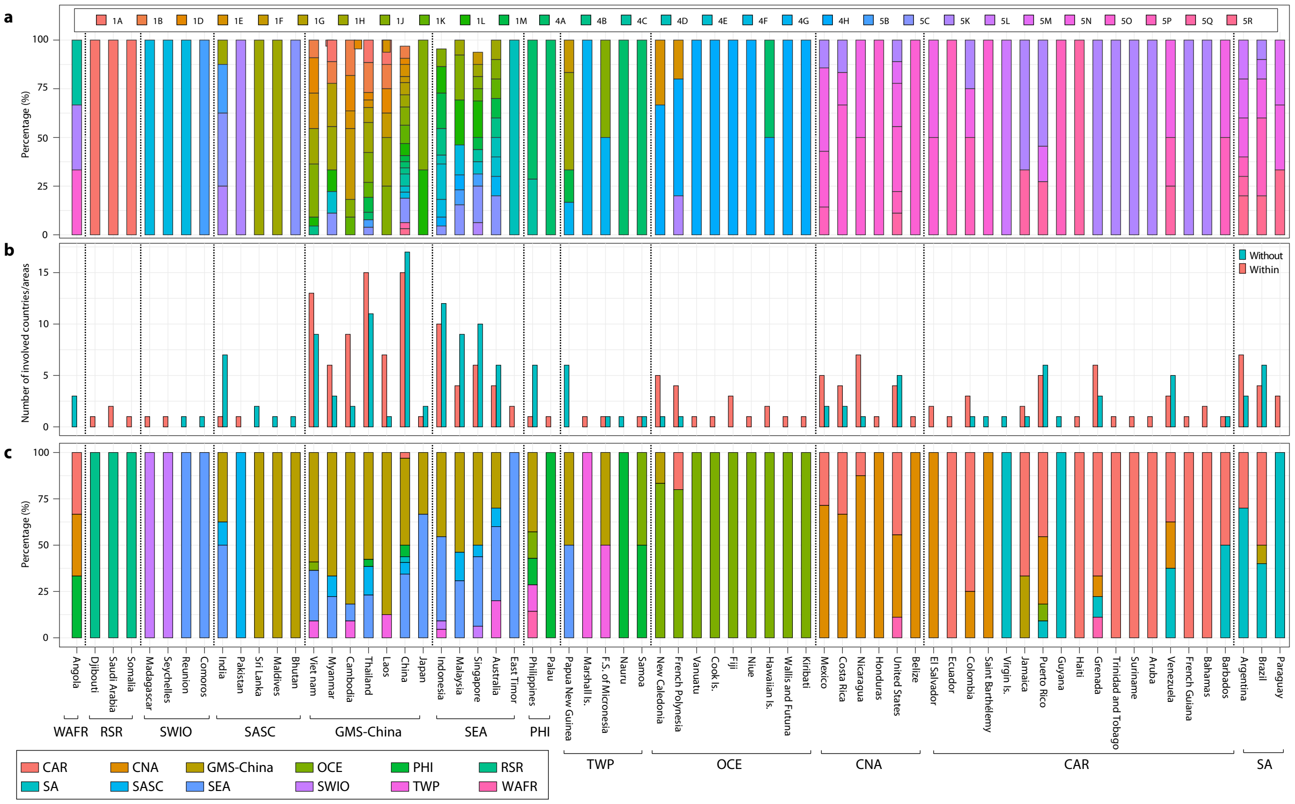

Additional file 1 Fig. S F2 DENV-1 transmission pairs in each epidemic country/area within and without its belonging epidemic regions inferred by BSSVS at the subgenotype level. a, Stacked barplots indicate the proportion of involved subgenotypes in each epidemic country/area. **b**, Numbers of involved transmission countries/areas. **c**, Stacked barplots show the proportion of epidemic regions involved transmission. WAFR, West African Region; RSR, Red Sea Region; SWIO, Southwest Indian Ocean; SASC, South Asia Subcontinent; GMS-China, Great Mekong Subregion-China; SEA, Southeast Asia; PHI, Philippines; TWP, Tropical Western Pacific; OCE, Oceania; CNA, Central North America; CAR, Caribbean; SA, South America.

G：Sources and trend of DENV-1 diffusion at clade level

Spatiotemporal dynamics and viral diversity assessed by PACT at the clade level

For demonstration of spatiotemporal dynamics and global epidemic patterns of DENV-1 at the clade level, PACT was used in the present study as a further extension of the BEAST analyses described above. Consequently, through inferring the trunks of epidemic countries/areas by time and coalescent estimation of migration rates of the involved 43 clades, including 26, 6 and 11 clades from genotypes I, IV and V of DENV-1, respectively, only those with a trunk probability >50% in certain clade in at least 12 consecutive months were estimated by PACT as the dominant epidemic locations (Fig.4, Fig. S G1). As shown in Fig. S-G1, it was obvious that the clades 1B8, 1C1 and 1D3 mainly persisted in Vietnam, 1G1 in Myanmar, 1H3 in Lao, 1M8 and 4B3 in Indonesia, 4A14 in the Philippines, 4D3 in East Timor, 5O3 and 5O4 in Mexico.

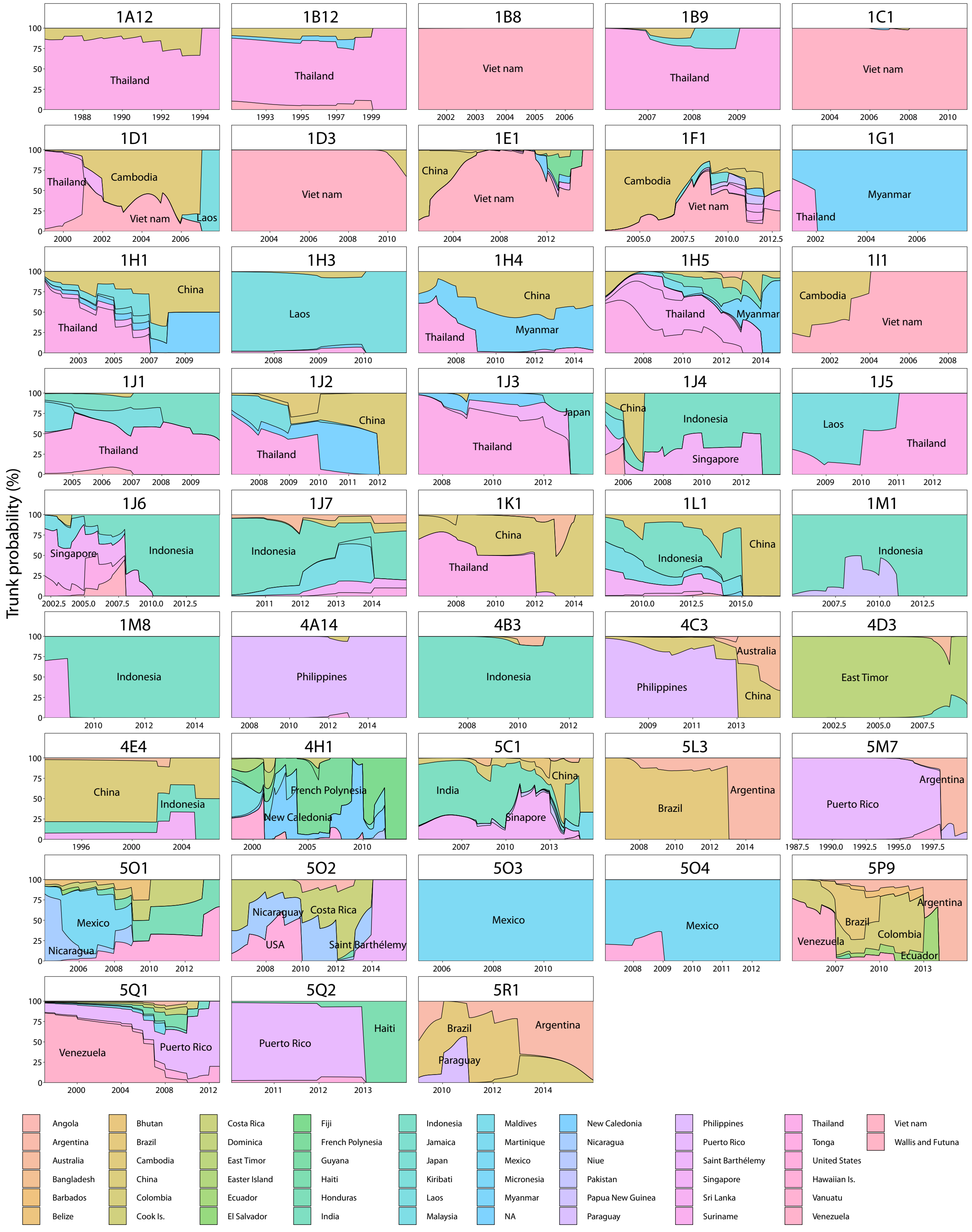

Additional file 1 Fig. S G1 Dynamic changes of trunks representing the proportion of PACT-inferred dominant epidemic countries/areas for each DENV-1 clade circulation. A total of 43 clades of DENV-1 were estimated by PACT. Coloured width at each time point indicates the posterior support for the DENV-1 clade circulating in the inferred dominant epidemic countries/areas, while colors indicate the countries/areas. PACT: posterior analysis of coalescent trees.

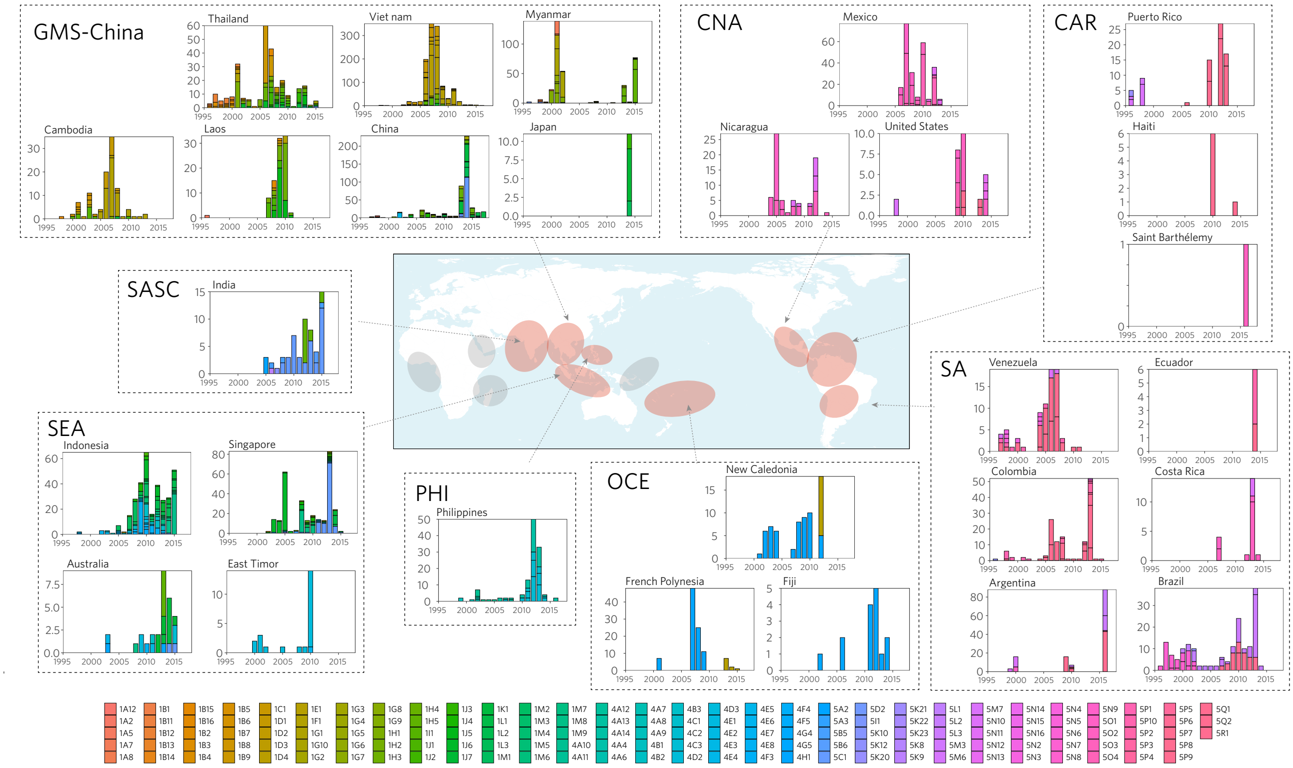

Additional file 1 Fig. S G2 Temporal dynamics of clades in 28 PACT-inferred dominant countries/areas. In each panel, the stacked barplots show reported sequences numbers, and the bars are colored to indicate the defined clades. The panels share the same x-axles (1995-2017), while the y-axles vary the corresponding maximum reported sequences number of source centers. SASC, South Asia Subcontinent; GMS-China, Great Mekong Subregion-China; SEA, Southeast Asia; PHI, Philippines; OCE, Oceania; CNA, Central North America; CAR, Caribbean; SA, South America

Additional file 1 Table S G1 The status of 28 PACT-inferred dominant epidemic countries/areas and the clades of DENV-1 involved

No	Epidemic region	Country/area	Involved DENV-1 clades inferred by PACT (Persisting years)	Clade numbers
1	GMS-China	Thailand	1A12(1986-1994), 1B9(2006-2009), 1B12(1991-2000), 1D1(1999-2000), 1G1(2001), 1H1(2001-2004), 1H4(2006-2007), 1H5(2006-2007), 1J1(2004-2008), 1J2(2007-2008), 1J3(2007-2012), 1J5(2010-2012), 1K1(2006-2011)	13
2		China	1E1(2001-2002), 1H1(2007-2010), 1H4(2009-2012), 1J2(2012), 1J4(2006), 1K1(2012-2014), 1L1(2015-2017), 4C3(2013-2014), 4E4(1993-2002, 2007), 5C1(2013, 2015)	10
3		Viet nam	1B8(2001-2007), 1C1(2002-2010), 1D2(2006-2011), 1D3(2002-2010), 1E1(2002-2015), 1F1(2007-2009), 1I1(2003-2008)	7
4		Cambodia	1D1(2002-2006), 1F1(2003-2007, 2012), 1I1(2000-2002)	3
5		Laos	1D1(2007), 1H3(2007-2010), 1J5(2008-2008)	3
6		Myanmar	1G1(2002-2007), 1H4(2009, 2013-2014)	2
7		Japan	1J3(2013)	1
8	SEA	Indonesia	1J1(2009), 1J4(2007-2013), 1J6(2008-2014), 1J7(2010-2012, 2014), 1L1(2010-2012, 2014), 1M1(2005-2014), 1M8(2009-2014), 4B3(2006-2012), 4E4(2005-2006), 4E8(2008-2011)	10
9		Singapore	1J4(2009, 2012), 1J6(2002-2004), 1M8(2008), 5C1(2010-2012)	4
10		Australia	4C3(2014)	1
11		East Timor	4D3(2000-2009)	1
12	SASC	India	5C1(2004-2014)	1
13	PHI	Philippines	4A14(2007-2015), 4C3(2007-2012)	2
14	OCE	French Polynesia	4H1(2001-2008)	1
15		Fiji	4H1(2006-2013)	1
16		New Caledonia	4H1(2001-2003, 2009)	1
17	SA	Brazil	5L3(2006-2012), 5N7(1991-2001), 5P9(2007-2008), 5R1(2010-2012)	4
18		Argentina	5L3(2013-2015), 5M7(1998-1999), 5P9(2014-2015), 5R1(2013-2015)	4
19	CNA	Mexico	5O1(2005-2007), 5O3(2007-2011), 5O4(2007-2012)	3
20		Nicaragua	5O1(2004)	1
21		United States	5O1(2013)	1
22	CAR	Puerto Rico	5M7(1987-1997), 5Q1(2008-2012), 5Q2(2010-2012)	3
23		Colombia	5P8(1998-2009), 5P9(2010-2012)	2
24		Venezuela	5P9(2004-2006), 5Q1(1997-2005)	2
25		Ecuador	5P9(2013)	1
26		Costa Rica	5O2(2006, 2012-2013)	
27		Saint Barthélemy	5O2(2014-2015)	
28		Haiti	5Q2(2014)	1

PACT: posterior analysis of coalescent trees. The clades are highlighted in light blue with a persistance of more than 5 years in the epidemic country/area.
