# Supplementary material for: A unified global genotyping framework of dengue virus serotype-1 for a stratified coordinated surveillance strategy of dengue epidemics
Source: Infect Dis Poverty. 2022 Oct 13;11:107. doi: 10.1186/s40249-022-01024-5 (PMC9556283; doi:10.1186/s40249-022-01024-5)
Supplement: Supplementary file 2 — Additional file 2. Characteristics of 5003 DENV-1 isolates worldwide from 1944 to 2018 inclusive in this study. [file 40249_2022_1024_MOESM2_ESM.docx]

**Appendix data A1. Characteristics of 5003 DENV-1 isolates worldwide from 1944 to 2018 inclusive in this study.**

| **No.** | **Year** | **Genebank** | **Country or area** | **Genotype** | **Subgenotype** | **Clade** |
| --- | --- | --- | --- | --- | --- | --- |
| 1 | 1944 | KM204119 | USA.Hawaii | I | 1A | 1A1 |
| 2 | 1980 | AY732421 | Thailand | I | 1A | 1A2 |
| 3 | 1981 | AY732385 | Thailand | I | 1A | 1A2 |
| 4 | 1981 | AY732394 | Thailand | I | 1A | 1A2 |
| 5 | 1981 | AY732406 | Thailand | I | 1A | 1A2 |
| 6 | 1981 | AY732483 | Thailand | I | 1A | 1A2 |
| 7 | 1981 | AY732383 | Thailand | I | 1A | 1A2 |
| 8 | 1982 | AY732397 | Thailand | I | 1A | 1A2 |
| 9 | 1982 | AY732481 | Thailand | I | 1A | 1A2 |
| 10 | 1985 | JN029817 | China | I | 1A | 1A2 |
| 11 | 1985 | JQ317728 | China | I | 1A | 1A2 |
| 12 | 1985 | JQ317729 | China | I | 1A | 1A2 |
| 13 | 1985 | JQ317730 | China | I | 1A | 1A2 |
| 14 | 1986 | AY732420 | Thailand | I | 1A | 1A2 |
| 15 | 1998 | JQ317711 | China | I | 1A | 1A2 |
| 16 | 1998 | JQ317712 | China | I | 1A | 1A2 |
| 17 | 1998 | JQ317713 | China | I | 1A | 1A2 |
| 18 | 1979 | JN029818 | China | I | 1A | 1A3 |
| 19 | 1980 | AF350498 | China | I | 1A | 1A3 |
| 20 | 1980 | AF425630 | Thailand | I | 1A | 1A3 |
| 21 | 1982 | AY732378 | Thailand | I | 1A | 1A3 |
| 22 | 1983 | AY732390 | Thailand | I | 1A | 1A3 |
| 23 | 1989 | AY732388 | Thailand | I | 1A | 1A4 |
| 24 | 1990 | AY732448 | Thailand | I | 1A | 1A4 |
| 25 | 1992 | AY732402 | Thailand | I | 1A | 1A4 |
| 26 | 1992 | AY732471 | Thailand | I | 1A | 1A4 |
| 27 | 1993 | AY732399 | Thailand | I | 1A | 1A4 |
| 28 | 1993 | AY732424 | Thailand | I | 1A | 1A4 |
| 29 | 1993 | AY732463 | Thailand | I | 1A | 1A4 |
| 30 | 1994 | AY732456 | Thailand | I | 1A | 1A4 |
| 31 | 1996 | AB003090 | Laos | I | 1A | 1A5 |
| 32 | 1992 | AY732395 | Thailand | I | 1A | 1A6 |
| 33 | 1999 | AY618878 | Myanmar | I | 1A | 1A7 |
| 34 | 2000 | AY618877 | Myanmar | I | 1A | 1A7 |
| 35 | 2000 | AY618879 | Myanmar | I | 1A | 1A7 |
| 36 | 2001 | AY713474 | Myanmar | I | 1A | 1A7 |
| 37 | 2001 | AY713475 | Myanmar | I | 1A | 1A7 |
| 38 | 2001 | DQ264877 | Myanmar | I | 1A | 1A7 |
| 39 | 2001 | DQ264949 | Myanmar | I | 1A | 1A7 |
| 40 | 2001 | DQ264950 | Myanmar | I | 1A | 1A7 |
| 41 | 2001 | DQ264951 | Myanmar | I | 1A | 1A7 |
| 42 | 2001 | DQ264952 | Myanmar | I | 1A | 1A7 |
| 43 | 2001 | DQ264953 | Myanmar | I | 1A | 1A7 |
| 44 | 2001 | DQ264954 | Myanmar | I | 1A | 1A7 |
| 45 | 2001 | DQ264955 | Myanmar | I | 1A | 1A7 |
| 46 | 2001 | DQ264956 | Myanmar | I | 1A | 1A7 |
| 47 | 2001 | DQ264957 | Myanmar | I | 1A | 1A7 |
| 48 | 2001 | DQ264958 | Myanmar | I | 1A | 1A7 |
| 49 | 2001 | DQ264959 | Myanmar | I | 1A | 1A7 |
| 50 | 2001 | DQ264960 | Myanmar | I | 1A | 1A7 |
| 51 | 2001 | DQ264961 | Myanmar | I | 1A | 1A7 |
| 52 | 2001 | DQ264964 | Myanmar | I | 1A | 1A7 |
| 53 | 2001 | DQ264965 | Myanmar | I | 1A | 1A7 |
| 54 | 2001 | DQ264966 | Myanmar | I | 1A | 1A7 |
| 55 | 2001 | DQ264967 | Myanmar | I | 1A | 1A7 |
| 56 | 2001 | DQ265011 | Myanmar | I | 1A | 1A7 |
| 57 | 2001 | DQ265013 | Myanmar | I | 1A | 1A7 |
| 58 | 2002 | AY726552 | Myanmar | I | 1A | 1A7 |
| 59 | 1998 | AY588272 | Myanmar | I | 1A | 1A8 |
| 60 | 1998 | AY726555 | Myanmar | I | 1A | 1A8 |
| 61 | 2001 | AY618210 | Myanmar | I | 1A | 1A8 |
| 62 | 2001 | AY726550 | Myanmar | I | 1A | 1A8 |
| 63 | 1987 | JN638341 | Thailand | I | 1A | 1A9 |
| 64 | 1989 | AY732410 | Thailand | I | 1A | 1A10 |
| 65 | 1985 | AY732426 | Thailand | I | 1A | 1A11 |
| 66 | 1985 | JN638328 | Thailand | I | 1A | 1A11 |
| 67 | 1990 | JN638342 | Thailand | I | 1A | 1A11 |
| 68 | 1986 | AY732432 | Thailand | I | 1A | 1A12 |
| 69 | 1986 | JN638329 | Thailand | I | 1A | 1A12 |
| 70 | 1986 | JN638336 | Thailand | I | 1A | 1A12 |
| 71 | 1987 | AF425628 | China | I | 1A | 1A12 |
| 72 | 1987 | AY732425 | Thailand | I | 1A | 1A12 |
| 73 | 1987 | AY732435 | Thailand | I | 1A | 1A12 |
| 74 | 1987 | AY732440 | Thailand | I | 1A | 1A12 |
| 75 | 1987 | AY732445 | Thailand | I | 1A | 1A12 |
| 76 | 1988 | AY732380 | Thailand | I | 1A | 1A12 |
| 77 | 1988 | AY732381 | Thailand | I | 1A | 1A12 |
| 78 | 1988 | AY732384 | Thailand | I | 1A | 1A12 |
| 79 | 1988 | AY732437 | Thailand | I | 1A | 1A12 |
| 80 | 1989 | AY732433 | Thailand | I | 1A | 1A12 |
| 81 | 1990 | AY732442 | Thailand | I | 1A | 1A12 |
| 82 | 1990 | AY732466 | Thailand | I | 1A | 1A12 |
| 83 | 1990 | JN638337 | Thailand | I | 1A | 1A12 |
| 84 | 1991 | AY732382 | Thailand | I | 1A | 1A12 |
| 85 | 1991 | AY732414 | Thailand | I | 1A | 1A12 |
| 86 | 1991 | AY732436 | Thailand | I | 1A | 1A12 |
| 87 | 1991 | AY732477 | Thailand | I | 1A | 1A12 |
| 88 | 1991 | AY732478 | Thailand | I | 1A | 1A12 |
| 89 | 1992 | AY732405 | Thailand | I | 1A | 1A12 |
| 90 | 1992 | AY732412 | Thailand | I | 1A | 1A12 |
| 91 | 1992 | JN638338 | Thailand | I | 1A | 1A12 |
| 92 | 1993 | AY732465 | Thailand | I | 1A | 1A12 |
| 93 | 1994 | AB608788 | China | I | 1A | 1A12 |
| 94 | 1994 | AY732468 | Thailand | I | 1A | 1A12 |
| 95 | 1995 | JN638344 | Thailand | I | 1A | 1A12 |
| 96 | 2004 | AM746216 | Saudi_Arabia | I | 1A | 1A13 |
| 97 | 2005 | AM746215 | Saudi_Arabia | I | 1A | 1A13 |
| 98 | 2006 | AM746212 | Saudi_Arabia | I | 1A | 1A13 |
| 99 | 2005 | AM746214 | Saudi_Arabia | I | 1A | 1A14 |
| 100 | 2006 | AM746213 | Saudi_Arabia | I | 1A | 1A14 |
| 101 | 2011 | KJ649286 | Saudi_Arabia | I | 1A | 1A14 |
| 102 | 2011 | KC848576 | Somalia | I | 1A | 1A15 |
| 103 | 2011 | KC848577 | Somalia | I | 1A | 1A15 |
| 104 | 2011 | KC848578 | Somalia | I | 1A | 1A15 |
| 105 | 2011 | KC848579 | Somalia | I | 1A | 1A16 |
| 106 | 2011 | KC848580 | Somalia | I | 1A | 1A17 |
| 107 | 2004 | AM746217 | Saudi_Arabia | I | 1A | 1A18 |
| 108 | 2010 | KU509258 | Eritrea | I | 1A | 1A19 |
| 109 | 1998 | AF298808 | Djibouti | I | 1A | 1A20 |
| 110 | 1990 | AY732441 | Thailand | I | 1B | 1B1 |
| 111 | 1991 | JQ317727 | China | I | 1B | 1B1 |
| 112 | 1992 | JN638343 | Thailand | I | 1B | 1B1 |
| 113 | 1993 | AY732461 | Thailand | I | 1B | 1B1 |
| 114 | 1994 | AY732393 | Thailand | I | 1B | 1B1 |
| 115 | 1994 | AY732427 | Thailand | I | 1B | 1B1 |
| 116 | 1994 | AY732475 | Thailand | I | 1B | 1B1 |
| 117 | 1994 | AY732480 | Thailand | I | 1B | 1B1 |
| 118 | 1995 | AY732404 | Thailand | I | 1B | 1B1 |
| 119 | 1996 | AY732423 | Thailand | I | 1B | 1B1 |
| 120 | 1997 | JQ317731 | China | I | 1B | 1B2 |
| 121 | 1997 | JQ317732 | China | I | 1B | 1B2 |
| 122 | 1999 | AY732443 | Thailand | I | 1B | 1B3 |
| 123 | 2000 | AY732407 | Thailand | I | 1B | 1B3 |
| 124 | 1993 | AY732415 | Thailand | I | 1B | 1B4 |
| 125 | 1995 | AY732457 | Thailand | I | 1B | 1B4 |
| 126 | 1995 | AY732473 | Thailand | I | 1B | 1B4 |
| 127 | 1995 | JN638339 | Thailand | I | 1B | 1B4 |
| 128 | 1996 | AY732455 | Thailand | I | 1B | 1B4 |
| 129 | 1997 | AY732450 | Thailand | I | 1B | 1B4 |
| 130 | 1997 | AY732453 | Thailand | I | 1B | 1B4 |
| 131 | 1997 | JN638340 | Thailand | I | 1B | 1B4 |
| 132 | 1999 | AY732470 | Thailand | I | 1B | 1B4 |
| 133 | 2001 | AY732464 | Thailand | I | 1B | 1B4 |
| 134 | 2004 | EU448386 | Thailand | I | 1B | 1B4 |
| 135 | 1997 | AY732459 | Thailand | I | 1B | 1B5 |
| 136 | 1998 | AY732454 | Thailand | I | 1B | 1B5 |
| 137 | 2000 | GQ868637 | Cambodia | I | 1B | 1B6 |
| 138 | 2001 | AB111069 | NA | I | 1B | 1B6 |
| 139 | 2001 | FJ639671 | Cambodia | I | 1B | 1B6 |
| 140 | 2001 | JN819423 | Cambodia | I | 1B | 1B6 |
| 141 | 2003 | FJ639678 | Cambodia | I | 1B | 1B6 |
| 142 | 2003 | JN638323 | Thailand | I | 1B | 1B6 |
| 143 | 2001 | KF955406 | Cambodia | I | 1B | 1B7 |
| 144 | 2003 | FJ639676 | Cambodia | I | 1B | 1B7 |
| 145 | 2003 | FJ850069 | Cambodia | I | 1B | 1B7 |
| 146 | 2003 | JN376779 | Vietnam | I | 1B | 1B7 |
| 147 | 2004 | JQ287664 | Cambodia | I | 1B | 1B7 |
| 148 | 2001 | FJ639673 | Cambodia | I | 1B | 1B8 |
| 149 | 2006 | FJ882520 | Vietnam | I | 1B | 1B8 |
| 150 | 2006 | FJ882537 | Vietnam | I | 1B | 1B8 |
| 151 | 2006 | FJ882541 | Vietnam | I | 1B | 1B8 |
| 152 | 2006 | FJ898407 | Vietnam | I | 1B | 1B8 |
| 153 | 2006 | FJ898423 | Vietnam | I | 1B | 1B8 |
| 154 | 2006 | FJ898424 | Vietnam | I | 1B | 1B8 |
| 155 | 2006 | FJ898429 | Vietnam | I | 1B | 1B8 |
| 156 | 2006 | GQ199843 | Vietnam | I | 1B | 1B8 |
| 157 | 2006 | JF937598 | Vietnam | I | 1B | 1B8 |
| 158 | 2007 | EU482486 | Vietnam | I | 1B | 1B8 |
| 159 | 2007 | EU482495 | Vietnam | I | 1B | 1B8 |
| 160 | 2007 | EU482510 | Vietnam | I | 1B | 1B8 |
| 161 | 2007 | EU660395 | Vietnam | I | 1B | 1B8 |
| 162 | 2007 | EU677151 | Vietnam | I | 1B | 1B8 |
| 163 | 2007 | EU677169 | Vietnam | I | 1B | 1B8 |
| 164 | 2007 | EU677176 | Vietnam | I | 1B | 1B8 |
| 165 | 2007 | FJ182033 | Vietnam | I | 1B | 1B8 |
| 166 | 2007 | FJ373297 | Vietnam | I | 1B | 1B8 |
| 167 | 2007 | FJ410231 | Vietnam | I | 1B | 1B8 |
| 168 | 2007 | FJ461318 | Vietnam | I | 1B | 1B8 |
| 169 | 2007 | FJ882549 | Vietnam | I | 1B | 1B8 |
| 170 | 2007 | FJ898373 | Vietnam | I | 1B | 1B8 |
| 171 | 2007 | GQ199774 | Vietnam | I | 1B | 1B8 |
| 172 | 2007 | GQ199775 | Vietnam | I | 1B | 1B8 |
| 173 | 2007 | GQ199785 | Vietnam | I | 1B | 1B8 |
| 174 | 2007 | JF937649 | Vietnam | I | 1B | 1B8 |
| 175 | 2006 | JQ993118 | Thailand | I | 1B | 1B9 |
| 176 | 2006 | JQ993119 | Thailand | I | 1B | 1B9 |
| 177 | 2006 | JQ993120 | Thailand | I | 1B | 1B9 |
| 178 | 2006 | JQ993152 | Thailand | I | 1B | 1B9 |
| 179 | 2006 | JQ993121 | Thailand | I | 1B | 1B9 |
| 180 | 2006 | JQ993122 | Thailand | I | 1B | 1B9 |
| 181 | 2006 | JQ993123 | Thailand | I | 1B | 1B9 |
| 182 | 2006 | JQ993124 | Thailand | I | 1B | 1B9 |
| 183 | 2006 | JQ993125 | Thailand | I | 1B | 1B9 |
| 184 | 2006 | JQ993126 | Thailand | I | 1B | 1B9 |
| 185 | 2006 | JQ993128 | Thailand | I | 1B | 1B9 |
| 186 | 2006 | JQ993129 | Thailand | I | 1B | 1B9 |
| 187 | 2006 | JQ993153 | Thailand | I | 1B | 1B9 |
| 188 | 2006 | JQ993154 | Thailand | I | 1B | 1B9 |
| 189 | 2006 | JQ993155 | Thailand | I | 1B | 1B9 |
| 190 | 2006 | JQ993156 | Thailand | I | 1B | 1B9 |
| 191 | 2006 | JQ993157 | Thailand | I | 1B | 1B9 |
| 192 | 2006 | JQ993158 | Thailand | I | 1B | 1B9 |
| 193 | 2006 | JQ993159 | Thailand | I | 1B | 1B9 |
| 194 | 2006 | JQ993160 | Thailand | I | 1B | 1B9 |
| 195 | 2006 | JQ993161 | Thailand | I | 1B | 1B9 |
| 196 | 2006 | JQ993162 | Thailand | I | 1B | 1B9 |
| 197 | 2006 | JQ993163 | Thailand | I | 1B | 1B9 |
| 198 | 2006 | JQ993164 | Thailand | I | 1B | 1B9 |
| 199 | 2006 | JQ993165 | Thailand | I | 1B | 1B9 |
| 200 | 2006 | JQ993166 | Thailand | I | 1B | 1B9 |
| 201 | 2006 | JQ993168 | Thailand | I | 1B | 1B9 |
| 202 | 2006 | JQ993169 | Thailand | I | 1B | 1B9 |
| 203 | 2006 | JQ993170 | Thailand | I | 1B | 1B9 |
| 204 | 2006 | JQ993172 | Thailand | I | 1B | 1B9 |
| 205 | 2006 | JQ993175 | Thailand | I | 1B | 1B9 |
| 206 | 2006 | JQ993176 | Thailand | I | 1B | 1B9 |
| 207 | 2006 | JQ993179 | Thailand | I | 1B | 1B9 |
| 208 | 2006 | JQ993180 | Thailand | I | 1B | 1B9 |
| 209 | 2006 | JQ993181 | Thailand | I | 1B | 1B9 |
| 210 | 2006 | JQ993182 | Thailand | I | 1B | 1B9 |
| 211 | 2006 | JQ993185 | Thailand | I | 1B | 1B9 |
| 212 | 2006 | JQ993186 | Thailand | I | 1B | 1B9 |
| 213 | 2006 | JQ993187 | Thailand | I | 1B | 1B9 |
| 214 | 2006 | JQ993190 | Thailand | I | 1B | 1B9 |
| 215 | 2006 | JQ993195 | Thailand | I | 1B | 1B9 |
| 216 | 2006 | JQ993197 | Thailand | I | 1B | 1B9 |
| 217 | 2007 | HM469967 | Thailand | I | 1B | 1B9 |
| 218 | 2007 | JQ993109 | Thailand | I | 1B | 1B9 |
| 219 | 2007 | JQ993113 | Thailand | I | 1B | 1B9 |
| 220 | 2007 | JQ993114 | Thailand | I | 1B | 1B9 |
| 221 | 2007 | JQ993115 | Thailand | I | 1B | 1B9 |
| 222 | 2007 | JQ993116 | Thailand | I | 1B | 1B9 |
| 223 | 2007 | JQ993136 | Thailand | I | 1B | 1B9 |
| 224 | 2007 | JQ993137 | Thailand | I | 1B | 1B9 |
| 225 | 2007 | JQ993138 | Thailand | I | 1B | 1B9 |
| 226 | 2007 | JQ993140 | Thailand | I | 1B | 1B9 |
| 227 | 2007 | JQ993142 | Thailand | I | 1B | 1B9 |
| 228 | 2007 | JQ993144 | Thailand | I | 1B | 1B9 |
| 229 | 2007 | JQ993145 | Thailand | I | 1B | 1B9 |
| 230 | 2007 | JQ993146 | Thailand | I | 1B | 1B9 |
| 231 | 2007 | JQ993202 | Thailand | I | 1B | 1B9 |
| 232 | 2008 | JF967812 | Thailand | I | 1B | 1B9 |
| 233 | 2008 | JN415526 | Thailand | I | 1B | 1B9 |
| 234 | 2008 | KU509259 | Thailand | I | 1B | 1B9 |
| 235 | 2008 | KU509260 | Cambodia | I | 1B | 1B9 |
| 236 | 2008 | KU509262 | Thailand | I | 1B | 1B9 |
| 237 | 2009 | JF967862 | Thailand | I | 1B | 1B9 |
| 238 | 2009 | KC172832 | Laos | I | 1B | 1B9 |
| 239 | 2009 | KU509256 | Thailand | I | 1B | 1B9 |
| 240 | 2010 | HG316481 | Thailand | I | 1B | 1B9 |
| 241 | 1994 | AB608789 | China | I | 1B | 1B10 |
| 242 | 1998 | KC861971 | Vietnam | I | 1B | 1B11 |
| 243 | 1991 | AY732413 | Thailand | I | 1B | 1B12 |
| 244 | 1995 | AY376738 | China | I | 1B | 1B12 |
| 245 | 1995 | AY732430 | Thailand | I | 1B | 1B12 |
| 246 | 1996 | AY732422 | Thailand | I | 1B | 1B12 |
| 247 | 1997 | AY732391 | Thailand | I | 1B | 1B12 |
| 248 | 1997 | AY732416 | Thailand | I | 1B | 1B12 |
| 249 | 1997 | AY732417 | Thailand | I | 1B | 1B12 |
| 250 | 1997 | AY732418 | Thailand | I | 1B | 1B12 |
| 251 | 1997 | AY732444 | Thailand | I | 1B | 1B12 |
| 252 | 1997 | JN638330 | Thailand | I | 1B | 1B12 |
| 253 | 1998 | AY588273 | Myanmar | I | 1B | 1B12 |
| 254 | 1998 | AY726554 | Myanmar | I | 1B | 1B12 |
| 255 | 1998 | AY732396 | Thailand | I | 1B | 1B12 |
| 256 | 1998 | AY732434 | Thailand | I | 1B | 1B12 |
| 257 | 1998 | AY732472 | Thailand | I | 1B | 1B12 |
| 258 | 1998 | EF508198 | China | I | 1B | 1B12 |
| 259 | 1999 | AY732400 | Thailand | I | 1B | 1B12 |
| 260 | 1999 | AY732446 | Thailand | I | 1B | 1B12 |
| 261 | 1999 | AY732449 | Thailand | I | 1B | 1B12 |
| 262 | 1999 | AY732458 | Thailand | I | 1B | 1B12 |
| 263 | 1999 | EF508199 | China | I | 1B | 1B12 |
| 264 | 1999 | KC861947 | Vietnam | I | 1B | 1B12 |
| 265 | 2000 | AY732408 | Thailand | I | 1B | 1B12 |
| 266 | 2000 | AY732452 | Thailand | I | 1B | 1B12 |
| 267 | 2001 | AY732451 | Thailand | I | 1B | 1B12 |
| 268 | 2001 | AY732462 | Thailand | I | 1B | 1B12 |
| 269 | 2000 | AY732460 | Thailand | I | 1B | 1B13 |
| 270 | 2011 | AB111066 | NA | I | 1B | 1B13 |
| 271 | 1998 | JQ317710 | China | I | 1B | 1B14 |
| 272 | 1997 | AY376737 | China | I | 1B | 1B15 |
| 273 | 1994 | AY732431 | Thailand | I | 1B | 1B16 |
| 274 | 1995 | AY732387 | Thailand | I | 1B | 1B16 |
| 275 | 1998 | AB111064 | Thailand | I | 1B | 1B16 |
| 276 | 1998 | KY496856 | China | I | 1B | 1B16 |
| 277 | 1998 | AF309641 | Cambodia | I | 1B | 1B16 |
| 278 | 2000 | AY732409 | Thailand | I | 1B | 1B16 |
| 279 | 2000 | JN638331 | Thailand | I | 1B | 1B16 |
| 280 | 2001 | JN415525 | Thailand | I | 1B | 1B16 |
| 281 | 2002 | AY732403 | Thailand | I | 1B | 1B16 |
| 282 | 2007 | JQ993117 | Thailand | I | 1B | 1B16 |
| 283 | 2007 | JQ993147 | Thailand | I | 1B | 1B16 |
| 284 | 2007 | JQ993149 | Thailand | I | 1B | 1B16 |
| 285 | 2007 | JQ993151 | Thailand | I | 1B | 1B16 |
| 286 | 2007 | JQ993204 | Thailand | I | 1B | 1B16 |
| 287 | 2008 | KC172829 | Laos | I | 1B | 1B16 |
| 288 | 2008 | KC172830 | Laos | I | 1B | 1B16 |
| 289 | 2008 | KC172835 | Laos | I | 1B | 1B16 |
| 290 | 2009 | KY849745 | Laos | I | 1B | 1B16 |
| 291 | 2002 | KC861930 | Vietnam | I | 1C | 1C1 |
| 292 | 2002 | KC861937 | Vietnam | I | 1C | 1C1 |
| 293 | 2002 | KC861979 | Vietnam | I | 1C | 1C1 |
| 294 | 2003 | EU482476 | Vietnam | I | 1C | 1C1 |
| 295 | 2003 | EU482789 | Vietnam | I | 1C | 1C1 |
| 296 | 2003 | EU482791 | Vietnam | I | 1C | 1C1 |
| 297 | 2003 | EU482792 | Vietnam | I | 1C | 1C1 |
| 298 | 2003 | FJ882564 | Vietnam | I | 1C | 1C1 |
| 299 | 2003 | FJ882565 | Vietnam | I | 1C | 1C1 |
| 300 | 2003 | FJ882566 | Vietnam | I | 1C | 1C1 |
| 301 | 2003 | FJ882567 | Vietnam | I | 1C | 1C1 |
| 302 | 2003 | FJ882568 | Vietnam | I | 1C | 1C1 |
| 303 | 2003 | GQ199830 | Vietnam | I | 1C | 1C1 |
| 304 | 2003 | GQ199831 | Vietnam | I | 1C | 1C1 |
| 305 | 2003 | GQ199832 | Vietnam | I | 1C | 1C1 |
| 306 | 2004 | EU448387 | Vietnam | I | 1C | 1C1 |
| 307 | 2004 | FJ882569 | Vietnam | I | 1C | 1C1 |
| 308 | 2004 | GQ199833 | Vietnam | I | 1C | 1C1 |
| 309 | 2004 | JN376780 | Vietnam | I | 1C | 1C1 |
| 310 | 2004 | KC861929 | Vietnam | I | 1C | 1C1 |
| 311 | 2004 | KC861943 | Vietnam | I | 1C | 1C1 |
| 312 | 2004 | KC861951 | Vietnam | I | 1C | 1C1 |
| 313 | 2004 | KC861954 | Vietnam | I | 1C | 1C1 |
| 314 | 2004 | KC861972 | Vietnam | I | 1C | 1C1 |
| 315 | 2004 | KY971685 | Vietnam | I | 1C | 1C1 |
| 316 | 2005 | FJ882570 | Vietnam | I | 1C | 1C1 |
| 317 | 2005 | FJ898386 | Vietnam | I | 1C | 1C1 |
| 318 | 2005 | FJ898389 | Vietnam | I | 1C | 1C1 |
| 319 | 2005 | FJ898392 | Vietnam | I | 1C | 1C1 |
| 320 | 2005 | FJ898394 | Vietnam | I | 1C | 1C1 |
| 321 | 2005 | GQ199834 | Vietnam | I | 1C | 1C1 |
| 322 | 2005 | GQ199835 | Vietnam | I | 1C | 1C1 |
| 323 | 2005 | GQ199836 | Vietnam | I | 1C | 1C1 |
| 324 | 2005 | GQ199837 | Vietnam | I | 1C | 1C1 |
| 325 | 2005 | GQ199838 | Vietnam | I | 1C | 1C1 |
| 326 | 2005 | JN376781 | Vietnam | I | 1C | 1C1 |
| 327 | 2005 | FJ898390 | Vietnam | I | 1C | 1C1 |
| 328 | 2005 | FJ898393 | Vietnam | I | 1C | 1C1 |
| 329 | 2005 | KC861939 | Vietnam | I | 1C | 1C1 |
| 330 | 2006 | EU249494 | Vietnam | I | 1C | 1C1 |
| 331 | 2006 | EU482526 | Vietnam | I | 1C | 1C1 |
| 332 | 2006 | EU482806 | Vietnam | I | 1C | 1C1 |
| 333 | 2006 | FJ882523 | Vietnam | I | 1C | 1C1 |
| 334 | 2006 | FJ882524 | Vietnam | I | 1C | 1C1 |
| 335 | 2006 | FJ882533 | Vietnam | I | 1C | 1C1 |
| 336 | 2006 | FJ898405 | Vietnam | I | 1C | 1C1 |
| 337 | 2006 | FJ898406 | Vietnam | I | 1C | 1C1 |
| 338 | 2006 | FJ898408 | Vietnam | I | 1C | 1C1 |
| 339 | 2006 | FJ898412 | Vietnam | I | 1C | 1C1 |
| 340 | 2006 | FJ898414 | Vietnam | I | 1C | 1C1 |
| 341 | 2006 | FJ898415 | Vietnam | I | 1C | 1C1 |
| 342 | 2006 | GQ199839 | Vietnam | I | 1C | 1C1 |
| 343 | 2006 | GQ199849 | Vietnam | I | 1C | 1C1 |
| 344 | 2006 | HQ588117 | Vietnam | I | 1C | 1C1 |
| 345 | 2006 | EU249495 | Vietnam | I | 1C | 1C1 |
| 346 | 2006 | EU482518 | Vietnam | I | 1C | 1C1 |
| 347 | 2006 | EU482519 | Vietnam | I | 1C | 1C1 |
| 348 | 2006 | EU482531 | Vietnam | I | 1C | 1C1 |
| 349 | 2006 | EU482537 | Vietnam | I | 1C | 1C1 |
| 350 | 2006 | EU482824 | Vietnam | I | 1C | 1C1 |
| 351 | 2006 | EU482827 | Vietnam | I | 1C | 1C1 |
| 352 | 2006 | GQ868605 | Vietnam | I | 1C | 1C1 |
| 353 | 2006 | EU482794 | Vietnam | I | 1C | 1C1 |
| 354 | 2006 | FJ882518 | Vietnam | I | 1C | 1C1 |
| 355 | 2006 | FJ882542 | Vietnam | I | 1C | 1C1 |
| 356 | 2006 | FJ898396 | Vietnam | I | 1C | 1C1 |
| 357 | 2006 | FJ898404 | Vietnam | I | 1C | 1C1 |
| 358 | 2006 | FJ898409 | Vietnam | I | 1C | 1C1 |
| 359 | 2006 | FJ898416 | Vietnam | I | 1C | 1C1 |
| 360 | 2006 | FJ898427 | Vietnam | I | 1C | 1C1 |
| 361 | 2006 | GQ199844 | Vietnam | I | 1C | 1C1 |
| 362 | 2006 | GQ199846 | Vietnam | I | 1C | 1C1 |
| 363 | 2006 | GQ199848 | Vietnam | I | 1C | 1C1 |
| 364 | 2006 | GQ199850 | Vietnam | I | 1C | 1C1 |
| 365 | 2006 | GQ199852 | Vietnam | I | 1C | 1C1 |
| 366 | 2006 | JQ287660 | Vietnam | I | 1C | 1C1 |
| 367 | 2006 | KF921937 | Vietnam | I | 1C | 1C1 |
| 368 | 2006 | EU482535 | Vietnam | I | 1C | 1C1 |
| 369 | 2006 | EU482796 | Vietnam | I | 1C | 1C1 |
| 370 | 2006 | EU482822 | Vietnam | I | 1C | 1C1 |
| 371 | 2006 | EU482825 | Vietnam | I | 1C | 1C1 |
| 372 | 2006 | FJ882515 | Vietnam | I | 1C | 1C1 |
| 373 | 2006 | FJ882543 | Vietnam | I | 1C | 1C1 |
| 374 | 2006 | FJ898425 | Vietnam | I | 1C | 1C1 |
| 375 | 2006 | FJ898428 | Vietnam | I | 1C | 1C1 |
| 376 | 2006 | JQ287661 | Vietnam | I | 1C | 1C1 |
| 377 | 2007 | EU448388 | Vietnam | I | 1C | 1C1 |
| 378 | 2007 | EU677173 | Vietnam | I | 1C | 1C1 |
| 379 | 2007 | FJ410218 | Vietnam | I | 1C | 1C1 |
| 380 | 2007 | FJ432723 | Vietnam | I | 1C | 1C1 |
| 381 | 2007 | FJ432729 | Vietnam | I | 1C | 1C1 |
| 382 | 2007 | FJ432733 | Vietnam | I | 1C | 1C1 |
| 383 | 2007 | FJ432749 | Vietnam | I | 1C | 1C1 |
| 384 | 2007 | FJ547060 | Vietnam | I | 1C | 1C1 |
| 385 | 2007 | FJ898371 | Vietnam | I | 1C | 1C1 |
| 386 | 2007 | FJ898376 | Vietnam | I | 1C | 1C1 |
| 387 | 2007 | FJ898385 | Vietnam | I | 1C | 1C1 |
| 388 | 2007 | GQ199784 | Vietnam | I | 1C | 1C1 |
| 389 | 2007 | GQ199790 | Vietnam | I | 1C | 1C1 |
| 390 | 2007 | GQ199791 | Vietnam | I | 1C | 1C1 |
| 391 | 2007 | GQ199793 | Vietnam | I | 1C | 1C1 |
| 392 | 2007 | GQ199805 | Vietnam | I | 1C | 1C1 |
| 393 | 2007 | GQ868610 | Vietnam | I | 1C | 1C1 |
| 394 | 2007 | HQ588118 | Vietnam | I | 1C | 1C1 |
| 395 | 2007 | HQ588119 | Vietnam | I | 1C | 1C1 |
| 396 | 2007 | JN415523 | NA | I | 1C | 1C1 |
| 397 | 2007 | KC861926 | Vietnam | I | 1C | 1C1 |
| 398 | 2007 | KC861952 | Vietnam | I | 1C | 1C1 |
| 399 | 2007 | KC861976 | Vietnam | I | 1C | 1C1 |
| 400 | 2007 | FJ024456 | Vietnam | I | 1C | 1C1 |
| 401 | 2007 | FJ898374 | Vietnam | I | 1C | 1C1 |
| 402 | 2007 | GQ199781 | Vietnam | I | 1C | 1C1 |
| 403 | 2007 | GQ199787 | Vietnam | I | 1C | 1C1 |
| 404 | 2007 | GQ199809 | Vietnam | I | 1C | 1C1 |
| 405 | 2007 | GQ868607 | Vietnam | I | 1C | 1C1 |
| 406 | 2007 | JF937596 | Vietnam | I | 1C | 1C1 |
| 407 | 2007 | KC861970 | Vietnam | I | 1C | 1C1 |
| 408 | 2007 | EU482481 | Vietnam | I | 1C | 1C1 |
| 409 | 2007 | EU482710 | Vietnam | I | 1C | 1C1 |
| 410 | 2007 | FJ182003 | Vietnam | I | 1C | 1C1 |
| 411 | 2007 | FJ432742 | Vietnam | I | 1C | 1C1 |
| 412 | 2007 | FJ461320 | Vietnam | I | 1C | 1C1 |
| 413 | 2007 | FJ882550 | Vietnam | I | 1C | 1C1 |
| 414 | 2007 | GQ199782 | Vietnam | I | 1C | 1C1 |
| 415 | 2007 | GQ199783 | Vietnam | I | 1C | 1C1 |
| 416 | 2007 | GQ199822 | Vietnam | I | 1C | 1C1 |
| 417 | 2007 | GQ868609 | Vietnam | I | 1C | 1C1 |
| 418 | 2007 | FJ882561 | Vietnam | I | 1C | 1C1 |
| 419 | 2008 | FJ410266 | Vietnam | I | 1C | 1C1 |
| 420 | 2008 | GU131681 | Vietnam | I | 1C | 1C1 |
| 421 | 2008 | HM181966 | Vietnam | I | 1C | 1C1 |
| 422 | 2008 | FJ410269 | Vietnam | I | 1C | 1C1 |
| 423 | 2008 | FJ410284 | Vietnam | I | 1C | 1C1 |
| 424 | 2008 | FJ461335 | Vietnam | I | 1C | 1C1 |
| 425 | 2008 | GQ868614 | Vietnam | I | 1C | 1C1 |
| 426 | 2008 | GQ868636 | Cambodia | I | 1C | 1C1 |
| 427 | 2008 | GU131763 | Vietnam | I | 1C | 1C1 |
| 428 | 2008 | GU131782 | Vietnam | I | 1C | 1C1 |
| 429 | 2008 | GU131820 | Vietnam | I | 1C | 1C1 |
| 430 | 2008 | KF921946 | Vietnam | I | 1C | 1C1 |
| 431 | 2008 | FJ410213 | Vietnam | I | 1C | 1C1 |
| 432 | 2008 | FJ410214 | Vietnam | I | 1C | 1C1 |
| 433 | 2008 | FJ410251 | Vietnam | I | 1C | 1C1 |
| 434 | 2008 | FJ410260 | Vietnam | I | 1C | 1C1 |
| 435 | 2008 | FJ410264 | Vietnam | I | 1C | 1C1 |
| 436 | 2008 | GU131696 | Vietnam | I | 1C | 1C1 |
| 437 | 2008 | GU131705 | Vietnam | I | 1C | 1C1 |
| 438 | 2008 | GU131715 | Vietnam | I | 1C | 1C1 |
| 439 | 2008 | GU131720 | Vietnam | I | 1C | 1C1 |
| 440 | 2008 | GU131726 | Vietnam | I | 1C | 1C1 |
| 441 | 2008 | GU131727 | Vietnam | I | 1C | 1C1 |
| 442 | 2008 | GU131731 | Vietnam | I | 1C | 1C1 |
| 443 | 2008 | GU131737 | Vietnam | I | 1C | 1C1 |
| 444 | 2008 | GU131738 | Vietnam | I | 1C | 1C1 |
| 445 | 2008 | GU131757 | Vietnam | I | 1C | 1C1 |
| 446 | 2008 | GU131768 | Vietnam | I | 1C | 1C1 |
| 447 | 2008 | GU131769 | Vietnam | I | 1C | 1C1 |
| 448 | 2008 | GU131775 | Vietnam | I | 1C | 1C1 |
| 449 | 2008 | GU131792 | Vietnam | I | 1C | 1C1 |
| 450 | 2008 | GU131798 | Vietnam | I | 1C | 1C1 |
| 451 | 2008 | GU131819 | Vietnam | I | 1C | 1C1 |
| 452 | 2008 | HM181968 | Vietnam | I | 1C | 1C1 |
| 453 | 2008 | JF937613 | Vietnam | I | 1C | 1C1 |
| 454 | 2008 | JF937616 | Vietnam | I | 1C | 1C1 |
| 455 | 2008 | JF967815 | Vietnam | I | 1C | 1C1 |
| 456 | 2008 | JF967827 | Vietnam | I | 1C | 1C1 |
| 457 | 2008 | JF967844 | Vietnam | I | 1C | 1C1 |
| 458 | 2008 | KF921950 | Vietnam | I | 1C | 1C1 |
| 459 | 2008 | GU131718 | Vietnam | I | 1C | 1C1 |
| 460 | 2008 | GU131753 | Vietnam | I | 1C | 1C1 |
| 461 | 2008 | GU131766 | Vietnam | I | 1C | 1C1 |
| 462 | 2008 | HQ591550 | Vietnam | I | 1C | 1C1 |
| 463 | 2008 | JF967849 | Vietnam | I | 1C | 1C1 |
| 464 | 2009 | KC861927 | Vietnam | I | 1C | 1C1 |
| 465 | 2009 | KC861953 | Vietnam | I | 1C | 1C1 |
| 466 | 2010 | JF967943 | Vietnam | I | 1C | 1C1 |
| 467 | 2011 | JX093672 | Vietnam | I | 1C | 1C1 |
| 468 | 2011 | JX093684 | Vietnam | I | 1C | 1C1 |
| 469 | 1999 | AY732439 | Thailand | I | 1D | 1D1 |
| 470 | 2000 | AY732469 | Thailand | I | 1D | 1D1 |
| 471 | 2000 | JN638322 | Thailand | I | 1D | 1D1 |
| 472 | 2001 | AY732428 | Thailand | I | 1D | 1D1 |
| 473 | 2001 | AY732438 | Thailand | I | 1D | 1D1 |
| 474 | 2001 | AY732467 | Thailand | I | 1D | 1D1 |
| 475 | 2002 | EU069599 | Singapore | I | 1D | 1D1 |
| 476 | 2002 | JN376778 | Vietnam | I | 1D | 1D1 |
| 477 | 2003 | FJ639675 | Cambodia | I | 1D | 1D1 |
| 478 | 2003 | GQ868618 | Cambodia | I | 1D | 1D1 |
| 479 | 2003 | KC861964 | Vietnam | I | 1D | 1D1 |
| 480 | 2005 | FJ639684 | Cambodia | I | 1D | 1D1 |
| 481 | 2006 | FJ639687 | Cambodia | I | 1D | 1D1 |
| 482 | 2006 | GQ868630 | Cambodia | I | 1D | 1D1 |
| 483 | 2006 | GQ868639 | Cambodia | I | 1D | 1D1 |
| 484 | 2006 | GU131890 | Cambodia | I | 1D | 1D1 |
| 485 | 2006 | GU131926 | Cambodia | I | 1D | 1D1 |
| 486 | 2006 | HM181939 | Cambodia | I | 1D | 1D1 |
| 487 | 2006 | HM181942 | Cambodia | I | 1D | 1D1 |
| 488 | 2007 | FJ639691 | Cambodia | I | 1D | 1D1 |
| 489 | 2007 | FJ639693 | Cambodia | I | 1D | 1D1 |
| 490 | 2007 | FJ639696 | Cambodia | I | 1D | 1D1 |
| 491 | 2007 | FJ882559 | Vietnam | I | 1D | 1D1 |
| 492 | 2007 | HM181944 | Cambodia | I | 1D | 1D1 |
| 493 | 2007 | HM181945 | Cambodia | I | 1D | 1D1 |
| 494 | 2007 | HM181951 | Cambodia | I | 1D | 1D1 |
| 495 | 2007 | JQ287665 | Cambodia | I | 1D | 1D1 |
| 496 | 2007 | KF955440 | Cambodia | I | 1D | 1D1 |
| 497 | 2008 | KC182104 | Laos | I | 1D | 1D1 |
| 498 | 2006 | EU249492 | Vietnam | I | 1D | 1D2 |
| 499 | 2006 | EU482534 | Vietnam | I | 1D | 1D2 |
| 500 | 2006 | EU482793 | Vietnam | I | 1D | 1D2 |
| 501 | 2006 | EU482798 | Vietnam | I | 1D | 1D2 |
| 502 | 2006 | EU482810 | Vietnam | I | 1D | 1D2 |
| 503 | 2006 | EU660392 | Vietnam | I | 1D | 1D2 |
| 504 | 2006 | EU482480 | Vietnam | I | 1D | 1D2 |
| 505 | 2006 | EU482536 | Vietnam | I | 1D | 1D2 |
| 506 | 2006 | EU482538 | Vietnam | I | 1D | 1D2 |
| 507 | 2006 | EU482706 | Vietnam | I | 1D | 1D2 |
| 508 | 2006 | EU482809 | Vietnam | I | 1D | 1D2 |
| 509 | 2006 | EU482826 | Vietnam | I | 1D | 1D2 |
| 510 | 2006 | EU482828 | Vietnam | I | 1D | 1D2 |
| 511 | 2006 | EU660402 | Vietnam | I | 1D | 1D2 |
| 512 | 2006 | EU687247 | Vietnam | I | 1D | 1D2 |
| 513 | 2006 | FJ882532 | Vietnam | I | 1D | 1D2 |
| 514 | 2006 | FJ898398 | Vietnam | I | 1D | 1D2 |
| 515 | 2006 | FJ898410 | Vietnam | I | 1D | 1D2 |
| 516 | 2006 | FJ898413 | Vietnam | I | 1D | 1D2 |
| 517 | 2006 | GQ199845 | Vietnam | I | 1D | 1D2 |
| 518 | 2006 | JF937599 | Vietnam | I | 1D | 1D2 |
| 519 | 2007 | FJ432746 | Vietnam | I | 1D | 1D2 |
| 520 | 2007 | FJ882551 | Vietnam | I | 1D | 1D2 |
| 521 | 2007 | FJ882555 | Vietnam | I | 1D | 1D2 |
| 522 | 2007 | GQ199811 | Vietnam | I | 1D | 1D2 |
| 523 | 2007 | HQ588146 | Vietnam | I | 1D | 1D2 |
| 524 | 2007 | EU448392 | Vietnam | I | 1D | 1D2 |
| 525 | 2007 | EU482484 | Vietnam | I | 1D | 1D2 |
| 526 | 2007 | EU482488 | Vietnam | I | 1D | 1D2 |
| 527 | 2007 | EU482491 | Vietnam | I | 1D | 1D2 |
| 528 | 2007 | EU482714 | Vietnam | I | 1D | 1D2 |
| 529 | 2007 | FJ898377 | Vietnam | I | 1D | 1D2 |
| 530 | 2007 | FJ898378 | Vietnam | I | 1D | 1D2 |
| 531 | 2007 | FJ898384 | Vietnam | I | 1D | 1D2 |
| 532 | 2007 | GQ199799 | Vietnam | I | 1D | 1D2 |
| 533 | 2007 | GQ199817 | Vietnam | I | 1D | 1D2 |
| 534 | 2007 | GQ199820 | Vietnam | I | 1D | 1D2 |
| 535 | 2008 | GU131684 | Vietnam | I | 1D | 1D2 |
| 536 | 2008 | GU131730 | Vietnam | I | 1D | 1D2 |
| 537 | 2008 | GU131756 | Vietnam | I | 1D | 1D2 |
| 538 | 2008 | GU131767 | Vietnam | I | 1D | 1D2 |
| 539 | 2008 | GU131786 | Vietnam | I | 1D | 1D2 |
| 540 | 2008 | GU131794 | Vietnam | I | 1D | 1D2 |
| 541 | 2008 | GU131811 | Vietnam | I | 1D | 1D2 |
| 542 | 2008 | HM181963 | Vietnam | I | 1D | 1D2 |
| 543 | 2008 | HQ591551 | Vietnam | I | 1D | 1D2 |
| 544 | 2008 | JF937602 | Vietnam | I | 1D | 1D2 |
| 545 | 2008 | JF967829 | Vietnam | I | 1D | 1D2 |
| 546 | 2008 | KC861977 | Vietnam | I | 1D | 1D2 |
| 547 | 2008 | KF921939 | Vietnam | I | 1D | 1D2 |
| 548 | 2008 | FJ410238 | Vietnam | I | 1D | 1D2 |
| 549 | 2008 | FJ461339 | Vietnam | I | 1D | 1D2 |
| 550 | 2008 | GU131689 | Vietnam | I | 1D | 1D2 |
| 551 | 2009 | JF967861 | Vietnam | I | 1D | 1D2 |
| 552 | 2009 | JF967881 | Vietnam | I | 1D | 1D2 |
| 553 | 2009 | KC861924 | Vietnam | I | 1D | 1D2 |
| 554 | 2011 | JX093683 | Vietnam | I | 1D | 1D2 |
| 555 | 2011 | JX093693 | Vietnam | I | 1D | 1D2 |
| 556 | 2011 | JX093695 | Vietnam | I | 1D | 1D2 |
| 557 | 2011 | JX093718 | Vietnam | I | 1D | 1D2 |
| 558 | 2012 | JX476090 | Vietnam | I | 1D | 1D2 |
| 559 | 2002 | FJ639674 | Cambodia | I | 1D | 1D3 |
| 560 | 2003 | EU482790 | Vietnam | I | 1D | 1D3 |
| 561 | 2003 | KC861920 | Vietnam | I | 1D | 1D3 |
| 562 | 2004 | KC861921 | Vietnam | I | 1D | 1D3 |
| 563 | 2004 | KC861923 | Vietnam | I | 1D | 1D3 |
| 564 | 2004 | KC861934 | Vietnam | I | 1D | 1D3 |
| 565 | 2004 | KC861956 | Vietnam | I | 1D | 1D3 |
| 566 | 2006 | FJ882529 | Vietnam | I | 1D | 1D3 |
| 567 | 2006 | GQ199840 | Vietnam | I | 1D | 1D3 |
| 568 | 2007 | FJ410252 | Vietnam | I | 1D | 1D3 |
| 569 | 2007 | GQ199800 | Vietnam | I | 1D | 1D3 |
| 570 | 2007 | GQ199814 | Vietnam | I | 1D | 1D3 |
| 571 | 2007 | HQ588128 | Vietnam | I | 1D | 1D3 |
| 572 | 2007 | EU482477 | Vietnam | I | 1D | 1D3 |
| 573 | 2007 | FJ024436 | Vietnam | I | 1D | 1D3 |
| 574 | 2007 | FJ182034 | Vietnam | I | 1D | 1D3 |
| 575 | 2007 | FJ205882 | Vietnam | I | 1D | 1D3 |
| 576 | 2007 | FJ410216 | Vietnam | I | 1D | 1D3 |
| 577 | 2007 | FJ410253 | Vietnam | I | 1D | 1D3 |
| 578 | 2007 | FJ562101 | Vietnam | I | 1D | 1D3 |
| 579 | 2007 | GQ199798 | Vietnam | I | 1D | 1D3 |
| 580 | 2007 | GQ199801 | Vietnam | I | 1D | 1D3 |
| 581 | 2008 | FJ410226 | Vietnam | I | 1D | 1D3 |
| 582 | 2008 | FJ410246 | Vietnam | I | 1D | 1D3 |
| 583 | 2008 | FJ410254 | Vietnam | I | 1D | 1D3 |
| 584 | 2008 | FJ410279 | Vietnam | I | 1D | 1D3 |
| 585 | 2008 | GU131686 | Vietnam | I | 1D | 1D3 |
| 586 | 2008 | GU131722 | Vietnam | I | 1D | 1D3 |
| 587 | 2008 | GU131744 | Vietnam | I | 1D | 1D3 |
| 588 | 2008 | GU131751 | Vietnam | I | 1D | 1D3 |
| 589 | 2008 | GU131754 | Vietnam | I | 1D | 1D3 |
| 590 | 2008 | GU131762 | Vietnam | I | 1D | 1D3 |
| 591 | 2008 | GU131772 | Vietnam | I | 1D | 1D3 |
| 592 | 2008 | GU131781 | Vietnam | I | 1D | 1D3 |
| 593 | 2008 | HQ591552 | Vietnam | I | 1D | 1D3 |
| 594 | 2008 | JF937603 | Vietnam | I | 1D | 1D3 |
| 595 | 2008 | JN415535 | Vietnam | I | 1D | 1D3 |
| 596 | 2008 | KC861974 | Vietnam | I | 1D | 1D3 |
| 597 | 2008 | KF921951 | Vietnam | I | 1D | 1D3 |
| 598 | 2008 | FJ410248 | Vietnam | I | 1D | 1D3 |
| 599 | 2008 | FJ410268 | Vietnam | I | 1D | 1D3 |
| 600 | 2008 | FJ410273 | Vietnam | I | 1D | 1D3 |
| 601 | 2008 | HM181961 | Vietnam | I | 1D | 1D3 |
| 602 | 2009 | JF967871 | Vietnam | I | 1D | 1D3 |
| 603 | 2009 | JX476084 | Vietnam | I | 1D | 1D3 |
| 604 | 2009 | JX569844 | Vietnam | I | 1D | 1D3 |
| 605 | 2009 | KC861931 | Vietnam | I | 1D | 1D3 |
| 606 | 2009 | KC861946 | Vietnam | I | 1D | 1D3 |
| 607 | 2009 | KC861950 | Vietnam | I | 1D | 1D3 |
| 608 | 2009 | KC861957 | Vietnam | I | 1D | 1D3 |
| 609 | 2010 | JF967953 | Vietnam | I | 1D | 1D3 |
| 610 | 2011 | JX093657 | Vietnam | I | 1D | 1D3 |
| 611 | 2011 | JX093663 | Vietnam | I | 1D | 1D3 |
| 612 | 2011 | JX093699 | Vietnam | I | 1D | 1D3 |
| 613 | 2011 | JX093712 | Vietnam | I | 1D | 1D3 |
| 614 | 2011 | KC136240 | China | I | 1D | 1D3 |
| 615 | 2011 | KT827369 | China | I | 1D | 1D3 |
| 616 | 1998 | KC861961 | Vietnam | I | 1D | 1D4 |
| 617 | 2005 | FJ898387 | Vietnam | I | 1D | 1D4 |
| 618 | 2005 | FJ898388 | Vietnam | I | 1D | 1D4 |
| 619 | 2005 | FJ898391 | Vietnam | I | 1D | 1D4 |
| 620 | 2006 | EU249490 | Vietnam | I | 1D | 1D4 |
| 621 | 2006 | EU249493 | Vietnam | I | 1D | 1D4 |
| 622 | 2006 | EU448391 | Indonesia | I | 1D | 1D4 |
| 623 | 2006 | EU482527 | Vietnam | I | 1D | 1D4 |
| 624 | 2006 | EU482528 | Vietnam | I | 1D | 1D4 |
| 625 | 2006 | EU482532 | Vietnam | I | 1D | 1D4 |
| 626 | 2006 | EU482533 | Vietnam | I | 1D | 1D4 |
| 627 | 2006 | EU482795 | Vietnam | I | 1D | 1D4 |
| 628 | 2006 | EU482797 | Vietnam | I | 1D | 1D4 |
| 629 | 2006 | EU482800 | Vietnam | I | 1D | 1D4 |
| 630 | 2006 | EU482802 | Vietnam | I | 1D | 1D4 |
| 631 | 2006 | EU482803 | Vietnam | I | 1D | 1D4 |
| 632 | 2006 | EU482804 | Vietnam | I | 1D | 1D4 |
| 633 | 2006 | EU482807 | Vietnam | I | 1D | 1D4 |
| 634 | 2006 | EU482808 | Vietnam | I | 1D | 1D4 |
| 635 | 2006 | EU482813 | Vietnam | I | 1D | 1D4 |
| 636 | 2006 | EU482814 | Vietnam | I | 1D | 1D4 |
| 637 | 2006 | EU482815 | Vietnam | I | 1D | 1D4 |
| 638 | 2006 | EU482816 | Vietnam | I | 1D | 1D4 |
| 639 | 2006 | EU482817 | Vietnam | I | 1D | 1D4 |
| 640 | 2006 | EU482819 | Vietnam | I | 1D | 1D4 |
| 641 | 2006 | EU482820 | Vietnam | I | 1D | 1D4 |
| 642 | 2006 | EU660391 | Vietnam | I | 1D | 1D4 |
| 643 | 2006 | EU660396 | Vietnam | I | 1D | 1D4 |
| 644 | 2006 | FJ882517 | Vietnam | I | 1D | 1D4 |
| 645 | 2006 | FJ882519 | Vietnam | I | 1D | 1D4 |
| 646 | 2006 | FJ882521 | Vietnam | I | 1D | 1D4 |
| 647 | 2006 | FJ882525 | Vietnam | I | 1D | 1D4 |
| 648 | 2006 | FJ882526 | Vietnam | I | 1D | 1D4 |
| 649 | 2006 | FJ882527 | Vietnam | I | 1D | 1D4 |
| 650 | 2006 | FJ882530 | Vietnam | I | 1D | 1D4 |
| 651 | 2006 | FJ882531 | Vietnam | I | 1D | 1D4 |
| 652 | 2006 | FJ882535 | Vietnam | I | 1D | 1D4 |
| 653 | 2006 | FJ882538 | Vietnam | I | 1D | 1D4 |
| 654 | 2006 | FJ882539 | Vietnam | I | 1D | 1D4 |
| 655 | 2006 | FJ882540 | Vietnam | I | 1D | 1D4 |
| 656 | 2006 | FJ898397 | Vietnam | I | 1D | 1D4 |
| 657 | 2006 | FJ898399 | Vietnam | I | 1D | 1D4 |
| 658 | 2006 | FJ898400 | Vietnam | I | 1D | 1D4 |
| 659 | 2006 | FJ898401 | Vietnam | I | 1D | 1D4 |
| 660 | 2006 | FJ898402 | Vietnam | I | 1D | 1D4 |
| 661 | 2006 | FJ898403 | Vietnam | I | 1D | 1D4 |
| 662 | 2006 | FJ898411 | Vietnam | I | 1D | 1D4 |
| 663 | 2006 | FJ898417 | Vietnam | I | 1D | 1D4 |
| 664 | 2006 | FJ898418 | Vietnam | I | 1D | 1D4 |
| 665 | 2006 | FJ898419 | Vietnam | I | 1D | 1D4 |
| 666 | 2006 | FJ898422 | Vietnam | I | 1D | 1D4 |
| 667 | 2006 | FJ898426 | Vietnam | I | 1D | 1D4 |
| 668 | 2006 | FJ898430 | Vietnam | I | 1D | 1D4 |
| 669 | 2006 | FJ898431 | Vietnam | I | 1D | 1D4 |
| 670 | 2006 | GQ199841 | Vietnam | I | 1D | 1D4 |
| 671 | 2006 | GQ199842 | Vietnam | I | 1D | 1D4 |
| 672 | 2006 | GQ199847 | Vietnam | I | 1D | 1D4 |
| 673 | 2006 | GQ199851 | Vietnam | I | 1D | 1D4 |
| 674 | 2006 | GQ199853 | Vietnam | I | 1D | 1D4 |
| 675 | 2006 | GQ199854 | Vietnam | I | 1D | 1D4 |
| 676 | 2006 | GQ199856 | Vietnam | I | 1D | 1D4 |
| 677 | 2006 | GQ868606 | Vietnam | I | 1D | 1D4 |
| 678 | 2006 | GQ868613 | Vietnam | I | 1D | 1D4 |
| 679 | 2006 | HQ588123 | Vietnam | I | 1D | 1D4 |
| 680 | 2006 | HQ588124 | Vietnam | I | 1D | 1D4 |
| 681 | 2006 | HQ588125 | Vietnam | I | 1D | 1D4 |
| 682 | 2006 | JN415533 | Vietnam | I | 1D | 1D4 |
| 683 | 2006 | KC861922 | Vietnam | I | 1D | 1D4 |
| 684 | 2006 | KC861966 | Vietnam | I | 1D | 1D4 |
| 685 | 2006 | KC861967 | Vietnam | I | 1D | 1D4 |
| 686 | 2006 | KC861975 | Vietnam | I | 1D | 1D4 |
| 687 | 2006 | KC861978 | Vietnam | I | 1D | 1D4 |
| 688 | 2006 | KF921934 | Vietnam | I | 1D | 1D4 |
| 689 | 2006 | KF921936 | Vietnam | I | 1D | 1D4 |
| 690 | 2007 | EU482505 | Vietnam | I | 1D | 1D4 |
| 691 | 2007 | EU482712 | Vietnam | I | 1D | 1D4 |
| 692 | 2007 | EU677150 | Vietnam | I | 1D | 1D4 |
| 693 | 2007 | EU677152 | Vietnam | I | 1D | 1D4 |
| 694 | 2007 | EU677160 | Vietnam | I | 1D | 1D4 |
| 695 | 2007 | EU677162 | Vietnam | I | 1D | 1D4 |
| 696 | 2007 | EU677164 | Vietnam | I | 1D | 1D4 |
| 697 | 2007 | EU677175 | Vietnam | I | 1D | 1D4 |
| 698 | 2007 | EU726781 | Vietnam | I | 1D | 1D4 |
| 699 | 2007 | FJ024459 | Vietnam | I | 1D | 1D4 |
| 700 | 2007 | FJ182020 | Vietnam | I | 1D | 1D4 |
| 701 | 2007 | FJ182024 | Vietnam | I | 1D | 1D4 |
| 702 | 2007 | FJ182032 | Vietnam | I | 1D | 1D4 |
| 703 | 2007 | FJ182035 | Vietnam | I | 1D | 1D4 |
| 704 | 2007 | FJ410199 | Vietnam | I | 1D | 1D4 |
| 705 | 2007 | FJ410207 | Vietnam | I | 1D | 1D4 |
| 706 | 2007 | FJ410227 | Vietnam | I | 1D | 1D4 |
| 707 | 2007 | FJ432730 | Vietnam | I | 1D | 1D4 |
| 708 | 2007 | FJ432735 | Vietnam | I | 1D | 1D4 |
| 709 | 2007 | FJ432739 | Vietnam | I | 1D | 1D4 |
| 710 | 2007 | FJ432744 | Vietnam | I | 1D | 1D4 |
| 711 | 2007 | FJ461312 | Vietnam | I | 1D | 1D4 |
| 712 | 2007 | FJ859029 | Vietnam | I | 1D | 1D4 |
| 713 | 2007 | FJ882552 | Vietnam | I | 1D | 1D4 |
| 714 | 2007 | FJ882558 | Vietnam | I | 1D | 1D4 |
| 715 | 2007 | FJ898375 | Vietnam | I | 1D | 1D4 |
| 716 | 2007 | FJ898381 | Vietnam | I | 1D | 1D4 |
| 717 | 2007 | FJ906964 | Vietnam | I | 1D | 1D4 |
| 718 | 2007 | FJ906965 | Vietnam | I | 1D | 1D4 |
| 719 | 2007 | GQ199773 | Vietnam | I | 1D | 1D4 |
| 720 | 2007 | GQ199780 | Vietnam | I | 1D | 1D4 |
| 721 | 2007 | GQ199788 | Vietnam | I | 1D | 1D4 |
| 722 | 2007 | GQ199789 | Vietnam | I | 1D | 1D4 |
| 723 | 2007 | GQ199797 | Vietnam | I | 1D | 1D4 |
| 724 | 2007 | GQ199806 | Vietnam | I | 1D | 1D4 |
| 725 | 2007 | GQ199807 | Vietnam | I | 1D | 1D4 |
| 726 | 2007 | GQ199808 | Vietnam | I | 1D | 1D4 |
| 727 | 2007 | GQ199813 | Vietnam | I | 1D | 1D4 |
| 728 | 2007 | GQ199816 | Vietnam | I | 1D | 1D4 |
| 729 | 2007 | GQ199821 | Vietnam | I | 1D | 1D4 |
| 730 | 2007 | GQ199824 | Vietnam | I | 1D | 1D4 |
| 731 | 2007 | GQ199826 | Vietnam | I | 1D | 1D4 |
| 732 | 2007 | GQ868608 | Vietnam | I | 1D | 1D4 |
| 733 | 2007 | HQ588126 | Vietnam | I | 1D | 1D4 |
| 734 | 2007 | HQ588127 | Vietnam | I | 1D | 1D4 |
| 735 | 2007 | JN376782 | Vietnam | I | 1D | 1D4 |
| 736 | 2007 | JQ287663 | Vietnam | I | 1D | 1D4 |
| 737 | 2007 | KC861940 | Vietnam | I | 1D | 1D4 |
| 738 | 2008 | FJ410191 | Vietnam | I | 1D | 1D4 |
| 739 | 2008 | FJ410235 | Vietnam | I | 1D | 1D4 |
| 740 | 2008 | FJ410240 | Vietnam | I | 1D | 1D4 |
| 741 | 2008 | FJ410242 | Vietnam | I | 1D | 1D4 |
| 742 | 2008 | FJ410243 | Vietnam | I | 1D | 1D4 |
| 743 | 2008 | FJ410244 | Vietnam | I | 1D | 1D4 |
| 744 | 2008 | FJ410249 | Vietnam | I | 1D | 1D4 |
| 745 | 2008 | FJ410256 | Vietnam | I | 1D | 1D4 |
| 746 | 2008 | FJ410262 | Vietnam | I | 1D | 1D4 |
| 747 | 2008 | FJ410281 | Vietnam | I | 1D | 1D4 |
| 748 | 2008 | FJ461308 | Vietnam | I | 1D | 1D4 |
| 749 | 2008 | GU131679 | Vietnam | I | 1D | 1D4 |
| 750 | 2008 | GU131687 | Vietnam | I | 1D | 1D4 |
| 751 | 2008 | GU131688 | Vietnam | I | 1D | 1D4 |
| 752 | 2008 | GU131692 | Vietnam | I | 1D | 1D4 |
| 753 | 2008 | GU131698 | Vietnam | I | 1D | 1D4 |
| 754 | 2008 | GU131700 | Vietnam | I | 1D | 1D4 |
| 755 | 2008 | GU131717 | Vietnam | I | 1D | 1D4 |
| 756 | 2008 | GU131734 | Vietnam | I | 1D | 1D4 |
| 757 | 2008 | GU131740 | Vietnam | I | 1D | 1D4 |
| 758 | 2008 | GU131741 | Vietnam | I | 1D | 1D4 |
| 759 | 2008 | GU131758 | Vietnam | I | 1D | 1D4 |
| 760 | 2008 | GU131759 | Vietnam | I | 1D | 1D4 |
| 761 | 2008 | GU131765 | Vietnam | I | 1D | 1D4 |
| 762 | 2008 | GU131778 | Vietnam | I | 1D | 1D4 |
| 763 | 2008 | GU131780 | Vietnam | I | 1D | 1D4 |
| 764 | 2008 | GU131784 | Vietnam | I | 1D | 1D4 |
| 765 | 2008 | GU131796 | Vietnam | I | 1D | 1D4 |
| 766 | 2008 | GU131799 | Vietnam | I | 1D | 1D4 |
| 767 | 2008 | GU131805 | Vietnam | I | 1D | 1D4 |
| 768 | 2008 | GU131809 | Vietnam | I | 1D | 1D4 |
| 769 | 2008 | GU131818 | Vietnam | I | 1D | 1D4 |
| 770 | 2008 | GU131821 | Vietnam | I | 1D | 1D4 |
| 771 | 2008 | HE795086 | Vietnam | I | 1D | 1D4 |
| 772 | 2008 | HM631850 | Vietnam | I | 1D | 1D4 |
| 773 | 2008 | HQ591538 | Vietnam | I | 1D | 1D4 |
| 774 | 2008 | HQ591539 | Vietnam | I | 1D | 1D4 |
| 775 | 2008 | HQ591540 | Vietnam | I | 1D | 1D4 |
| 776 | 2008 | HQ591541 | Vietnam | I | 1D | 1D4 |
| 777 | 2008 | HQ591543 | Vietnam | I | 1D | 1D4 |
| 778 | 2008 | HQ591546 | Vietnam | I | 1D | 1D4 |
| 779 | 2008 | HQ591549 | Vietnam | I | 1D | 1D4 |
| 780 | 2008 | HQ591553 | Vietnam | I | 1D | 1D4 |
| 781 | 2008 | HQ591554 | Vietnam | I | 1D | 1D4 |
| 782 | 2008 | JF269176 | Vietnam | I | 1D | 1D4 |
| 783 | 2008 | JF937615 | Vietnam | I | 1D | 1D4 |
| 784 | 2008 | JF967795 | Vietnam | I | 1D | 1D4 |
| 785 | 2008 | JF967799 | Vietnam | I | 1D | 1D4 |
| 786 | 2008 | JN093516 | Vietnam | I | 1D | 1D4 |
| 787 | 2008 | JQ403516 | China | I | 1D | 1D4 |
| 788 | 2008 | KC861938 | Vietnam | I | 1D | 1D4 |
| 789 | 2008 | KC861955 | Vietnam | I | 1D | 1D4 |
| 790 | 2008 | KF921944 | Vietnam | I | 1D | 1D4 |
| 791 | 2008 | KY971687 | Vietnam | I | 1D | 1D4 |
| 792 | 2009 | JF967851 | Vietnam | I | 1D | 1D4 |
| 793 | 2009 | KC861959 | Vietnam | I | 1D | 1D4 |
| 794 | 2009 | KC861960 | Vietnam | I | 1D | 1D4 |
| 795 | 2009 | KC861962 | Vietnam | I | 1D | 1D4 |
| 796 | 2001 | AB111071 | Cambodia | I | 1E | 1E1 |
| 797 | 2002 | JQ317716 | China | I | 1E | 1E1 |
| 798 | 2002 | JQ317746 | China | I | 1E | 1E1 |
| 799 | 2006 | EF508203 | China | I | 1E | 1E1 |
| 800 | 2006 | EU482520 | Vietnam | I | 1E | 1E1 |
| 801 | 2006 | EU482522 | Vietnam | I | 1E | 1E1 |
| 802 | 2006 | EU482523 | Vietnam | I | 1E | 1E1 |
| 803 | 2006 | EU482524 | Vietnam | I | 1E | 1E1 |
| 804 | 2006 | EU482529 | Vietnam | I | 1E | 1E1 |
| 805 | 2006 | EU482530 | Vietnam | I | 1E | 1E1 |
| 806 | 2006 | EU482539 | Vietnam | I | 1E | 1E1 |
| 807 | 2006 | EU482799 | Vietnam | I | 1E | 1E1 |
| 808 | 2006 | EU482801 | Vietnam | I | 1E | 1E1 |
| 809 | 2006 | EU482805 | Vietnam | I | 1E | 1E1 |
| 810 | 2006 | EU482812 | Vietnam | I | 1E | 1E1 |
| 811 | 2006 | EU482818 | Vietnam | I | 1E | 1E1 |
| 812 | 2006 | EU482821 | Vietnam | I | 1E | 1E1 |
| 813 | 2006 | EU482823 | Vietnam | I | 1E | 1E1 |
| 814 | 2006 | EU660393 | Vietnam | I | 1E | 1E1 |
| 815 | 2006 | EU660394 | Vietnam | I | 1E | 1E1 |
| 816 | 2006 | EU660401 | Vietnam | I | 1E | 1E1 |
| 817 | 2006 | EU660403 | Vietnam | I | 1E | 1E1 |
| 818 | 2006 | FJ024444 | Vietnam | I | 1E | 1E1 |
| 819 | 2006 | FJ176780 | China | I | 1E | 1E1 |
| 820 | 2006 | FJ182018 | Vietnam | I | 1E | 1E1 |
| 821 | 2006 | FJ196843 | China | I | 1E | 1E1 |
| 822 | 2006 | FJ373305 | Vietnam | I | 1E | 1E1 |
| 823 | 2006 | FJ410287 | Vietnam | I | 1E | 1E1 |
| 824 | 2006 | FJ882522 | Vietnam | I | 1E | 1E1 |
| 825 | 2006 | FJ882544 | Vietnam | I | 1E | 1E1 |
| 826 | 2006 | GQ199771 | Vietnam | I | 1E | 1E1 |
| 827 | 2006 | GQ199855 | Vietnam | I | 1E | 1E1 |
| 828 | 2006 | EU660397 | Vietnam | I | 1E | 1E1 |
| 829 | 2007 | EU448390 | Vietnam | I | 1E | 1E1 |
| 830 | 2007 | EU482478 | Vietnam | I | 1E | 1E1 |
| 831 | 2007 | EU482479 | Vietnam | I | 1E | 1E1 |
| 832 | 2007 | EU482485 | Vietnam | I | 1E | 1E1 |
| 833 | 2007 | EU482487 | Vietnam | I | 1E | 1E1 |
| 834 | 2007 | EU482490 | Vietnam | I | 1E | 1E1 |
| 835 | 2007 | EU482492 | Vietnam | I | 1E | 1E1 |
| 836 | 2007 | EU482496 | Vietnam | I | 1E | 1E1 |
| 837 | 2007 | EU482499 | Vietnam | I | 1E | 1E1 |
| 838 | 2007 | EU482500 | Vietnam | I | 1E | 1E1 |
| 839 | 2007 | EU482501 | Vietnam | I | 1E | 1E1 |
| 840 | 2007 | EU482502 | Vietnam | I | 1E | 1E1 |
| 841 | 2007 | EU482503 | Vietnam | I | 1E | 1E1 |
| 842 | 2007 | EU482506 | Vietnam | I | 1E | 1E1 |
| 843 | 2007 | EU482507 | Vietnam | I | 1E | 1E1 |
| 844 | 2007 | EU482508 | Vietnam | I | 1E | 1E1 |
| 845 | 2007 | EU482509 | Vietnam | I | 1E | 1E1 |
| 846 | 2007 | EU482512 | Vietnam | I | 1E | 1E1 |
| 847 | 2007 | EU482514 | Vietnam | I | 1E | 1E1 |
| 848 | 2007 | EU482516 | Vietnam | I | 1E | 1E1 |
| 849 | 2007 | EU482711 | Vietnam | I | 1E | 1E1 |
| 850 | 2007 | EU482713 | Vietnam | I | 1E | 1E1 |
| 851 | 2007 | EU482717 | Vietnam | I | 1E | 1E1 |
| 852 | 2007 | EU660418 | Vietnam | I | 1E | 1E1 |
| 853 | 2007 | EU660419 | Vietnam | I | 1E | 1E1 |
| 854 | 2007 | EU677140 | Vietnam | I | 1E | 1E1 |
| 855 | 2007 | EU677153 | Vietnam | I | 1E | 1E1 |
| 856 | 2007 | EU677156 | Vietnam | I | 1E | 1E1 |
| 857 | 2007 | EU677158 | Vietnam | I | 1E | 1E1 |
| 858 | 2007 | EU677168 | Vietnam | I | 1E | 1E1 |
| 859 | 2007 | EU677178 | Vietnam | I | 1E | 1E1 |
| 860 | 2007 | EU687251 | Vietnam | I | 1E | 1E1 |
| 861 | 2007 | EU726778 | Vietnam | I | 1E | 1E1 |
| 862 | 2007 | EU726779 | Vietnam | I | 1E | 1E1 |
| 863 | 2007 | EU726782 | Vietnam | I | 1E | 1E1 |
| 864 | 2007 | FJ024430 | Vietnam | I | 1E | 1E1 |
| 865 | 2007 | FJ024432 | Vietnam | I | 1E | 1E1 |
| 866 | 2007 | FJ024438 | Vietnam | I | 1E | 1E1 |
| 867 | 2007 | FJ024439 | Vietnam | I | 1E | 1E1 |
| 868 | 2007 | FJ024441 | Vietnam | I | 1E | 1E1 |
| 869 | 2007 | FJ024442 | Vietnam | I | 1E | 1E1 |
| 870 | 2007 | FJ024445 | Vietnam | I | 1E | 1E1 |
| 871 | 2007 | FJ024446 | Vietnam | I | 1E | 1E1 |
| 872 | 2007 | FJ024447 | Vietnam | I | 1E | 1E1 |
| 873 | 2007 | FJ024449 | Vietnam | I | 1E | 1E1 |
| 874 | 2007 | FJ024451 | Vietnam | I | 1E | 1E1 |
| 875 | 2007 | FJ182019 | Vietnam | I | 1E | 1E1 |
| 876 | 2007 | FJ182021 | Vietnam | I | 1E | 1E1 |
| 877 | 2007 | FJ182025 | Vietnam | I | 1E | 1E1 |
| 878 | 2007 | FJ182030 | Vietnam | I | 1E | 1E1 |
| 879 | 2007 | FJ205883 | Vietnam | I | 1E | 1E1 |
| 880 | 2007 | FJ373298 | Vietnam | I | 1E | 1E1 |
| 881 | 2007 | FJ390381 | Vietnam | I | 1E | 1E1 |
| 882 | 2007 | FJ390382 | Vietnam | I | 1E | 1E1 |
| 883 | 2007 | FJ390383 | Vietnam | I | 1E | 1E1 |
| 884 | 2007 | FJ410194 | Vietnam | I | 1E | 1E1 |
| 885 | 2007 | FJ410196 | Vietnam | I | 1E | 1E1 |
| 886 | 2007 | FJ410197 | Vietnam | I | 1E | 1E1 |
| 887 | 2007 | FJ410198 | Vietnam | I | 1E | 1E1 |
| 888 | 2007 | FJ410206 | Vietnam | I | 1E | 1E1 |
| 889 | 2007 | FJ410210 | Vietnam | I | 1E | 1E1 |
| 890 | 2007 | FJ410225 | Vietnam | I | 1E | 1E1 |
| 891 | 2007 | FJ432725 | Vietnam | I | 1E | 1E1 |
| 892 | 2007 | FJ432727 | Vietnam | I | 1E | 1E1 |
| 893 | 2007 | FJ432732 | Vietnam | I | 1E | 1E1 |
| 894 | 2007 | FJ432737 | Vietnam | I | 1E | 1E1 |
| 895 | 2007 | FJ432738 | Vietnam | I | 1E | 1E1 |
| 896 | 2007 | FJ432740 | Vietnam | I | 1E | 1E1 |
| 897 | 2007 | FJ432748 | Vietnam | I | 1E | 1E1 |
| 898 | 2007 | FJ461306 | Vietnam | I | 1E | 1E1 |
| 899 | 2007 | FJ461325 | Vietnam | I | 1E | 1E1 |
| 900 | 2007 | FJ461327 | Vietnam | I | 1E | 1E1 |
| 901 | 2007 | FJ547065 | Vietnam | I | 1E | 1E1 |
| 902 | 2007 | FJ639688 | Cambodia | I | 1E | 1E1 |
| 903 | 2007 | FJ882554 | Vietnam | I | 1E | 1E1 |
| 904 | 2007 | FJ882556 | Vietnam | I | 1E | 1E1 |
| 905 | 2007 | FJ898379 | Vietnam | I | 1E | 1E1 |
| 906 | 2007 | FJ898380 | Vietnam | I | 1E | 1E1 |
| 907 | 2007 | FJ898382 | Vietnam | I | 1E | 1E1 |
| 908 | 2007 | FJ898383 | Vietnam | I | 1E | 1E1 |
| 909 | 2007 | GQ199776 | Vietnam | I | 1E | 1E1 |
| 910 | 2007 | GQ199777 | Vietnam | I | 1E | 1E1 |
| 911 | 2007 | GQ199778 | Vietnam | I | 1E | 1E1 |
| 912 | 2007 | GQ199779 | Vietnam | I | 1E | 1E1 |
| 913 | 2007 | GQ199795 | Vietnam | I | 1E | 1E1 |
| 914 | 2007 | GQ199796 | Vietnam | I | 1E | 1E1 |
| 915 | 2007 | GQ199802 | Vietnam | I | 1E | 1E1 |
| 916 | 2007 | GQ199804 | Vietnam | I | 1E | 1E1 |
| 917 | 2007 | GQ199810 | Vietnam | I | 1E | 1E1 |
| 918 | 2007 | GQ199812 | Vietnam | I | 1E | 1E1 |
| 919 | 2007 | GQ199815 | Vietnam | I | 1E | 1E1 |
| 920 | 2007 | GQ199819 | Vietnam | I | 1E | 1E1 |
| 921 | 2007 | GQ199823 | Vietnam | I | 1E | 1E1 |
| 922 | 2007 | GQ199827 | Vietnam | I | 1E | 1E1 |
| 923 | 2007 | GQ199829 | Vietnam | I | 1E | 1E1 |
| 924 | 2007 | KF921935 | Vietnam | I | 1E | 1E1 |
| 925 | 2007 | EU482482 | Vietnam | I | 1E | 1E1 |
| 926 | 2007 | EU482483 | Vietnam | I | 1E | 1E1 |
| 927 | 2007 | EU482493 | Vietnam | I | 1E | 1E1 |
| 928 | 2007 | EU482494 | Vietnam | I | 1E | 1E1 |
| 929 | 2007 | EU482497 | Vietnam | I | 1E | 1E1 |
| 930 | 2007 | EU482498 | Vietnam | I | 1E | 1E1 |
| 931 | 2007 | EU482504 | Vietnam | I | 1E | 1E1 |
| 932 | 2007 | EU482511 | Vietnam | I | 1E | 1E1 |
| 933 | 2007 | EU482515 | Vietnam | I | 1E | 1E1 |
| 934 | 2007 | EU482517 | Vietnam | I | 1E | 1E1 |
| 935 | 2007 | EU482708 | Vietnam | I | 1E | 1E1 |
| 936 | 2007 | EU482709 | Vietnam | I | 1E | 1E1 |
| 937 | 2007 | EU482715 | Vietnam | I | 1E | 1E1 |
| 938 | 2007 | EU482718 | Vietnam | I | 1E | 1E1 |
| 939 | 2007 | EU677139 | Vietnam | I | 1E | 1E1 |
| 940 | 2007 | EU677155 | Vietnam | I | 1E | 1E1 |
| 941 | 2007 | EU677157 | Vietnam | I | 1E | 1E1 |
| 942 | 2007 | EU677163 | Vietnam | I | 1E | 1E1 |
| 943 | 2007 | EU677165 | Vietnam | I | 1E | 1E1 |
| 944 | 2007 | EU677166 | Vietnam | I | 1E | 1E1 |
| 945 | 2007 | EU677167 | Vietnam | I | 1E | 1E1 |
| 946 | 2007 | EU677170 | Vietnam | I | 1E | 1E1 |
| 947 | 2007 | EU677171 | Vietnam | I | 1E | 1E1 |
| 948 | 2007 | EU677177 | Vietnam | I | 1E | 1E1 |
| 949 | 2007 | EU726777 | Vietnam | I | 1E | 1E1 |
| 950 | 2007 | EU726780 | Vietnam | I | 1E | 1E1 |
| 951 | 2007 | FJ024427 | Vietnam | I | 1E | 1E1 |
| 952 | 2007 | FJ024428 | Vietnam | I | 1E | 1E1 |
| 953 | 2007 | FJ024429 | Vietnam | I | 1E | 1E1 |
| 954 | 2007 | FJ024433 | Vietnam | I | 1E | 1E1 |
| 955 | 2007 | FJ024435 | Vietnam | I | 1E | 1E1 |
| 956 | 2007 | FJ024437 | Vietnam | I | 1E | 1E1 |
| 957 | 2007 | FJ024440 | Vietnam | I | 1E | 1E1 |
| 958 | 2007 | FJ024443 | Vietnam | I | 1E | 1E1 |
| 959 | 2007 | FJ024448 | Vietnam | I | 1E | 1E1 |
| 960 | 2007 | FJ024450 | Vietnam | I | 1E | 1E1 |
| 961 | 2007 | FJ024457 | Vietnam | I | 1E | 1E1 |
| 962 | 2007 | FJ024460 | Vietnam | I | 1E | 1E1 |
| 963 | 2007 | FJ024462 | Vietnam | I | 1E | 1E1 |
| 964 | 2007 | FJ024463 | Vietnam | I | 1E | 1E1 |
| 965 | 2007 | FJ024464 | Vietnam | I | 1E | 1E1 |
| 966 | 2007 | FJ024472 | Vietnam | I | 1E | 1E1 |
| 967 | 2007 | FJ182022 | Vietnam | I | 1E | 1E1 |
| 968 | 2007 | FJ182023 | Vietnam | I | 1E | 1E1 |
| 969 | 2007 | FJ182026 | Vietnam | I | 1E | 1E1 |
| 970 | 2007 | FJ182027 | Vietnam | I | 1E | 1E1 |
| 971 | 2007 | FJ182028 | Vietnam | I | 1E | 1E1 |
| 972 | 2007 | FJ182029 | Vietnam | I | 1E | 1E1 |
| 973 | 2007 | FJ182031 | Vietnam | I | 1E | 1E1 |
| 974 | 2007 | FJ182036 | Vietnam | I | 1E | 1E1 |
| 975 | 2007 | FJ205876 | Vietnam | I | 1E | 1E1 |
| 976 | 2007 | FJ205884 | Vietnam | I | 1E | 1E1 |
| 977 | 2007 | FJ390388 | Vietnam | I | 1E | 1E1 |
| 978 | 2007 | FJ410222 | Vietnam | I | 1E | 1E1 |
| 979 | 2007 | FJ410289 | Vietnam | I | 1E | 1E1 |
| 980 | 2007 | FJ461303 | Vietnam | I | 1E | 1E1 |
| 981 | 2007 | FJ547063 | Vietnam | I | 1E | 1E1 |
| 982 | 2007 | FJ882553 | Vietnam | I | 1E | 1E1 |
| 983 | 2007 | GQ199786 | Vietnam | I | 1E | 1E1 |
| 984 | 2007 | GQ199794 | Vietnam | I | 1E | 1E1 |
| 985 | 2007 | JQ287662 | Vietnam | I | 1E | 1E1 |
| 986 | 2007 | EU482489 | Vietnam | I | 1E | 1E1 |
| 987 | 2007 | EU482513 | Vietnam | I | 1E | 1E1 |
| 988 | 2007 | EU482716 | Vietnam | I | 1E | 1E1 |
| 989 | 2007 | EU677154 | Vietnam | I | 1E | 1E1 |
| 990 | 2007 | EU677161 | Vietnam | I | 1E | 1E1 |
| 991 | 2007 | EU677172 | Vietnam | I | 1E | 1E1 |
| 992 | 2007 | EU677174 | Vietnam | I | 1E | 1E1 |
| 993 | 2007 | FJ024425 | Vietnam | I | 1E | 1E1 |
| 994 | 2007 | FJ024426 | Vietnam | I | 1E | 1E1 |
| 995 | 2007 | FJ024453 | Vietnam | I | 1E | 1E1 |
| 996 | 2007 | FJ024455 | Vietnam | I | 1E | 1E1 |
| 997 | 2007 | FJ205881 | Vietnam | I | 1E | 1E1 |
| 998 | 2007 | FJ390386 | Vietnam | I | 1E | 1E1 |
| 999 | 2007 | FJ410205 | Vietnam | I | 1E | 1E1 |
| 1000 | 2007 | FJ432719 | Vietnam | I | 1E | 1E1 |
| 1001 | 2007 | FJ461315 | Vietnam | I | 1E | 1E1 |
| 1002 | 2007 | FJ906963 | Vietnam | I | 1E | 1E1 |
| 1003 | 2007 | GQ868611 | Vietnam | I | 1E | 1E1 |
| 1004 | 2008 | AB608786 | China | I | 1E | 1E1 |
| 1005 | 2008 | FJ410245 | Vietnam | I | 1E | 1E1 |
| 1006 | 2008 | FJ410258 | Vietnam | I | 1E | 1E1 |
| 1007 | 2008 | FJ410263 | Vietnam | I | 1E | 1E1 |
| 1008 | 2008 | FJ410265 | Vietnam | I | 1E | 1E1 |
| 1009 | 2008 | FJ410270 | Vietnam | I | 1E | 1E1 |
| 1010 | 2008 | FJ410272 | Vietnam | I | 1E | 1E1 |
| 1011 | 2008 | FJ410275 | Vietnam | I | 1E | 1E1 |
| 1012 | 2008 | FJ410277 | Vietnam | I | 1E | 1E1 |
| 1013 | 2008 | FJ410278 | Vietnam | I | 1E | 1E1 |
| 1014 | 2008 | FJ410282 | Vietnam | I | 1E | 1E1 |
| 1015 | 2008 | FJ410285 | Vietnam | I | 1E | 1E1 |
| 1016 | 2008 | FJ461310 | Vietnam | I | 1E | 1E1 |
| 1017 | 2008 | FJ461336 | Vietnam | I | 1E | 1E1 |
| 1018 | 2008 | FJ461341 | Vietnam | I | 1E | 1E1 |
| 1019 | 2008 | GU131682 | Vietnam | I | 1E | 1E1 |
| 1020 | 2008 | GU131691 | Vietnam | I | 1E | 1E1 |
| 1021 | 2008 | GU131693 | Vietnam | I | 1E | 1E1 |
| 1022 | 2008 | GU131694 | Vietnam | I | 1E | 1E1 |
| 1023 | 2008 | GU131699 | Vietnam | I | 1E | 1E1 |
| 1024 | 2008 | GU131701 | Vietnam | I | 1E | 1E1 |
| 1025 | 2008 | GU131704 | Vietnam | I | 1E | 1E1 |
| 1026 | 2008 | GU131707 | Vietnam | I | 1E | 1E1 |
| 1027 | 2008 | GU131710 | Vietnam | I | 1E | 1E1 |
| 1028 | 2008 | GU131716 | Vietnam | I | 1E | 1E1 |
| 1029 | 2008 | GU131719 | Vietnam | I | 1E | 1E1 |
| 1030 | 2008 | GU131721 | Vietnam | I | 1E | 1E1 |
| 1031 | 2008 | GU131735 | Vietnam | I | 1E | 1E1 |
| 1032 | 2008 | GU131743 | Vietnam | I | 1E | 1E1 |
| 1033 | 2008 | GU131750 | Vietnam | I | 1E | 1E1 |
| 1034 | 2008 | GU131791 | Vietnam | I | 1E | 1E1 |
| 1035 | 2008 | GU131813 | Vietnam | I | 1E | 1E1 |
| 1036 | 2008 | GU131815 | Vietnam | I | 1E | 1E1 |
| 1037 | 2008 | GU131823 | Vietnam | I | 1E | 1E1 |
| 1038 | 2008 | GU131828 | Vietnam | I | 1E | 1E1 |
| 1039 | 2008 | HM181960 | Vietnam | I | 1E | 1E1 |
| 1040 | 2008 | HM181962 | Vietnam | I | 1E | 1E1 |
| 1041 | 2008 | HM181967 | Vietnam | I | 1E | 1E1 |
| 1042 | 2008 | HQ591542 | Vietnam | I | 1E | 1E1 |
| 1043 | 2008 | JF269178 | Vietnam | I | 1E | 1E1 |
| 1044 | 2008 | JF937600 | Vietnam | I | 1E | 1E1 |
| 1045 | 2008 | JF937601 | Vietnam | I | 1E | 1E1 |
| 1046 | 2008 | JF937604 | Vietnam | I | 1E | 1E1 |
| 1047 | 2008 | JF937605 | Vietnam | I | 1E | 1E1 |
| 1048 | 2008 | JF937612 | Vietnam | I | 1E | 1E1 |
| 1049 | 2008 | JF967816 | Vietnam | I | 1E | 1E1 |
| 1050 | 2008 | JF967830 | Vietnam | I | 1E | 1E1 |
| 1051 | 2008 | JF967836 | Vietnam | I | 1E | 1E1 |
| 1052 | 2008 | JF967841 | Vietnam | I | 1E | 1E1 |
| 1053 | 2008 | JF967842 | Vietnam | I | 1E | 1E1 |
| 1054 | 2008 | JN000935 | Vietnam | I | 1E | 1E1 |
| 1055 | 2008 | JN196566 | Singapore | I | 1E | 1E1 |
| 1056 | 2008 | JN415534 | Vietnam | I | 1E | 1E1 |
| 1057 | 2008 | JQ403518 | China | I | 1E | 1E1 |
| 1058 | 2008 | KC861932 | Vietnam | I | 1E | 1E1 |
| 1059 | 2008 | KF921940 | Vietnam | I | 1E | 1E1 |
| 1060 | 2008 | KF921943 | Vietnam | I | 1E | 1E1 |
| 1061 | 2008 | KF921945 | Vietnam | I | 1E | 1E1 |
| 1062 | 2008 | KF971712 | Vietnam | I | 1E | 1E1 |
| 1063 | 2008 | KY971688 | Vietnam | I | 1E | 1E1 |
| 1064 | 2008 | FJ410192 | Vietnam | I | 1E | 1E1 |
| 1065 | 2008 | FJ410230 | Vietnam | I | 1E | 1E1 |
| 1066 | 2008 | FJ410236 | Vietnam | I | 1E | 1E1 |
| 1067 | 2008 | FJ410255 | Vietnam | I | 1E | 1E1 |
| 1068 | 2008 | FJ410261 | Vietnam | I | 1E | 1E1 |
| 1069 | 2008 | FJ461330 | Vietnam | I | 1E | 1E1 |
| 1070 | 2008 | FJ461340 | Vietnam | I | 1E | 1E1 |
| 1071 | 2008 | GQ868615 | Vietnam | I | 1E | 1E1 |
| 1072 | 2008 | GU131685 | Vietnam | I | 1E | 1E1 |
| 1073 | 2008 | GU131690 | Vietnam | I | 1E | 1E1 |
| 1074 | 2008 | GU131695 | Vietnam | I | 1E | 1E1 |
| 1075 | 2008 | GU131706 | Vietnam | I | 1E | 1E1 |
| 1076 | 2008 | GU131723 | Vietnam | I | 1E | 1E1 |
| 1077 | 2008 | GU131724 | Vietnam | I | 1E | 1E1 |
| 1078 | 2008 | GU131729 | Vietnam | I | 1E | 1E1 |
| 1079 | 2008 | GU131746 | Vietnam | I | 1E | 1E1 |
| 1080 | 2008 | GU131748 | Vietnam | I | 1E | 1E1 |
| 1081 | 2008 | GU131755 | Vietnam | I | 1E | 1E1 |
| 1082 | 2008 | GU131760 | Vietnam | I | 1E | 1E1 |
| 1083 | 2008 | GU131761 | Vietnam | I | 1E | 1E1 |
| 1084 | 2008 | GU131770 | Vietnam | I | 1E | 1E1 |
| 1085 | 2008 | GU131776 | Vietnam | I | 1E | 1E1 |
| 1086 | 2008 | GU131783 | Vietnam | I | 1E | 1E1 |
| 1087 | 2008 | GU131787 | Vietnam | I | 1E | 1E1 |
| 1088 | 2008 | GU131790 | Vietnam | I | 1E | 1E1 |
| 1089 | 2008 | GU131793 | Vietnam | I | 1E | 1E1 |
| 1090 | 2008 | GU131795 | Vietnam | I | 1E | 1E1 |
| 1091 | 2008 | GU131797 | Vietnam | I | 1E | 1E1 |
| 1092 | 2008 | GU131803 | Vietnam | I | 1E | 1E1 |
| 1093 | 2008 | GU131807 | Vietnam | I | 1E | 1E1 |
| 1094 | 2008 | GU131808 | Vietnam | I | 1E | 1E1 |
| 1095 | 2008 | GU131812 | Vietnam | I | 1E | 1E1 |
| 1096 | 2008 | GU131827 | Vietnam | I | 1E | 1E1 |
| 1097 | 2008 | HM181964 | Vietnam | I | 1E | 1E1 |
| 1098 | 2008 | HM181969 | Vietnam | I | 1E | 1E1 |
| 1099 | 2008 | JF269177 | Vietnam | I | 1E | 1E1 |
| 1100 | 2008 | JF937606 | Vietnam | I | 1E | 1E1 |
| 1101 | 2008 | JF937607 | Vietnam | I | 1E | 1E1 |
| 1102 | 2008 | JF937610 | Vietnam | I | 1E | 1E1 |
| 1103 | 2008 | JF937614 | Vietnam | I | 1E | 1E1 |
| 1104 | 2008 | JF967817 | Vietnam | I | 1E | 1E1 |
| 1105 | 2008 | JF967823 | Vietnam | I | 1E | 1E1 |
| 1106 | 2008 | JF967845 | Vietnam | I | 1E | 1E1 |
| 1107 | 2008 | KC861941 | Vietnam | I | 1E | 1E1 |
| 1108 | 2008 | KF921947 | Vietnam | I | 1E | 1E1 |
| 1109 | 2008 | KF921948 | Vietnam | I | 1E | 1E1 |
| 1110 | 2008 | KF921949 | Vietnam | I | 1E | 1E1 |
| 1111 | 2008 | GU131739 | Vietnam | I | 1E | 1E1 |
| 1112 | 2008 | GU131824 | Vietnam | I | 1E | 1E1 |
| 1113 | 2008 | FJ410239 | Vietnam | I | 1E | 1E1 |
| 1114 | 2008 | FJ410276 | Vietnam | I | 1E | 1E1 |
| 1115 | 2008 | FJ410283 | Vietnam | I | 1E | 1E1 |
| 1116 | 2008 | GU131683 | Vietnam | I | 1E | 1E1 |
| 1117 | 2008 | GU131702 | Vietnam | I | 1E | 1E1 |
| 1118 | 2008 | GU131732 | Vietnam | I | 1E | 1E1 |
| 1119 | 2008 | GU131733 | Vietnam | I | 1E | 1E1 |
| 1120 | 2008 | GU131742 | Vietnam | I | 1E | 1E1 |
| 1121 | 2008 | GU131745 | Vietnam | I | 1E | 1E1 |
| 1122 | 2008 | GU131747 | Vietnam | I | 1E | 1E1 |
| 1123 | 2008 | GU131785 | Vietnam | I | 1E | 1E1 |
| 1124 | 2008 | GU131804 | Vietnam | I | 1E | 1E1 |
| 1125 | 2008 | GU131806 | Vietnam | I | 1E | 1E1 |
| 1126 | 2008 | GU131825 | Vietnam | I | 1E | 1E1 |
| 1127 | 2008 | GU131831 | Vietnam | I | 1E | 1E1 |
| 1128 | 2008 | HM488256 | Vietnam | I | 1E | 1E1 |
| 1129 | 2008 | JF937611 | Vietnam | I | 1E | 1E1 |
| 1130 | 2008 | JF967800 | Vietnam | I | 1E | 1E1 |
| 1131 | 2008 | JF967806 | Vietnam | I | 1E | 1E1 |
| 1132 | 2008 | JF967833 | Vietnam | I | 1E | 1E1 |
| 1133 | 2008 | JF967834 | Vietnam | I | 1E | 1E1 |
| 1134 | 2008 | JF967835 | Vietnam | I | 1E | 1E1 |
| 1135 | 2008 | JF967837 | Vietnam | I | 1E | 1E1 |
| 1136 | 2008 | KF921941 | Vietnam | I | 1E | 1E1 |
| 1137 | 2008 | KF921942 | Vietnam | I | 1E | 1E1 |
| 1138 | 2008 | KF955446 | Vietnam | I | 1E | 1E1 |
| 1139 | 2008 | KY971690 | Vietnam | I | 1E | 1E1 |
| 1140 | 2009 | JF269181 | Vietnam | I | 1E | 1E1 |
| 1141 | 2009 | JF967856 | Vietnam | I | 1E | 1E1 |
| 1142 | 2009 | JF967860 | Vietnam | I | 1E | 1E1 |
| 1143 | 2009 | JF967863 | Vietnam | I | 1E | 1E1 |
| 1144 | 2009 | JF967869 | Vietnam | I | 1E | 1E1 |
| 1145 | 2009 | JF967891 | Vietnam | I | 1E | 1E1 |
| 1146 | 2009 | JX446303 | Vietnam | I | 1E | 1E1 |
| 1147 | 2009 | JX446304 | Vietnam | I | 1E | 1E1 |
| 1148 | 2009 | JX446306 | Vietnam | I | 1E | 1E1 |
| 1149 | 2009 | JX569841 | Vietnam | I | 1E | 1E1 |
| 1150 | 2009 | JX569846 | Vietnam | I | 1E | 1E1 |
| 1151 | 2009 | KC861917 | Vietnam | I | 1E | 1E1 |
| 1152 | 2009 | KC861925 | Vietnam | I | 1E | 1E1 |
| 1153 | 2009 | KC861945 | Vietnam | I | 1E | 1E1 |
| 1154 | 2009 | KC861973 | Vietnam | I | 1E | 1E1 |
| 1155 | 2009 | JF269182 | Vietnam | I | 1E | 1E1 |
| 1156 | 2009 | JF967850 | Vietnam | I | 1E | 1E1 |
| 1157 | 2009 | JF967853 | Vietnam | I | 1E | 1E1 |
| 1158 | 2009 | JF967883 | Vietnam | I | 1E | 1E1 |
| 1159 | 2009 | JX446301 | Vietnam | I | 1E | 1E1 |
| 1160 | 2009 | JX569845 | Vietnam | I | 1E | 1E1 |
| 1161 | 2009 | KC861963 | Vietnam | I | 1E | 1E1 |
| 1162 | 2009 | JF967890 | Vietnam | I | 1E | 1E1 |
| 1163 | 2009 | JX446299 | Vietnam | I | 1E | 1E1 |
| 1164 | 2009 | JX446300 | Vietnam | I | 1E | 1E1 |
| 1165 | 2009 | JX446302 | Vietnam | I | 1E | 1E1 |
| 1166 | 2009 | JX446305 | Vietnam | I | 1E | 1E1 |
| 1167 | 2009 | JX476085 | Vietnam | I | 1E | 1E1 |
| 1168 | 2009 | JF269183 | Vietnam | I | 1E | 1E1 |
| 1169 | 2009 | JF967852 | Vietnam | I | 1E | 1E1 |
| 1170 | 2009 | JF967864 | Vietnam | I | 1E | 1E1 |
| 1171 | 2009 | JF967873 | Vietnam | I | 1E | 1E1 |
| 1172 | 2009 | JF967874 | Vietnam | I | 1E | 1E1 |
| 1173 | 2009 | JF967884 | Thailand | I | 1E | 1E1 |
| 1174 | 2009 | JF967885 | Vietnam | I | 1E | 1E1 |
| 1175 | 2009 | JF967886 | Vietnam | I | 1E | 1E1 |
| 1176 | 2009 | JF967892 | Vietnam | I | 1E | 1E1 |
| 1177 | 2009 | JF967893 | Vietnam | I | 1E | 1E1 |
| 1178 | 2009 | JX569839 | Vietnam | I | 1E | 1E1 |
| 1179 | 2009 | JX569843 | Vietnam | I | 1E | 1E1 |
| 1180 | 2010 | JF967927 | Vietnam | I | 1E | 1E1 |
| 1181 | 2010 | JF967929 | Thailand | I | 1E | 1E1 |
| 1182 | 2010 | JF967934 | Vietnam | I | 1E | 1E1 |
| 1183 | 2010 | JF967944 | Vietnam | I | 1E | 1E1 |
| 1184 | 2010 | JF967948 | Vietnam | I | 1E | 1E1 |
| 1185 | 2010 | KY971705 | Vietnam | I | 1E | 1E1 |
| 1186 | 2010 | JF967909 | Vietnam | I | 1E | 1E1 |
| 1187 | 2010 | JF967910 | Vietnam | I | 1E | 1E1 |
| 1188 | 2010 | JF967912 | Vietnam | I | 1E | 1E1 |
| 1189 | 2010 | JF967941 | Vietnam | I | 1E | 1E1 |
| 1190 | 2010 | JQ403521 | China | I | 1E | 1E1 |
| 1191 | 2010 | KY971702 | Vietnam | I | 1E | 1E1 |
| 1192 | 2010 | JF967913 | Vietnam | I | 1E | 1E1 |
| 1193 | 2010 | JF967914 | Vietnam | I | 1E | 1E1 |
| 1194 | 2010 | JF967915 | Vietnam | I | 1E | 1E1 |
| 1195 | 2010 | JF967942 | Vietnam | I | 1E | 1E1 |
| 1196 | 2010 | JN376783 | Vietnam | I | 1E | 1E1 |
| 1197 | 2010 | KY971706 | Vietnam | I | 1E | 1E1 |
| 1198 | 2010 | KY971707 | Vietnam | I | 1E | 1E1 |
| 1199 | 2010 | KY971708 | Vietnam | I | 1E | 1E1 |
| 1200 | 2010 | JN415487 | Vietnam | I | 1E | 1E1 |
| 1201 | 2010 | JF960221 | Singapore | I | 1E | 1E1 |
| 1202 | 2010 | JF967898 | Vietnam | I | 1E | 1E1 |
| 1203 | 2010 | JF967933 | Vietnam | I | 1E | 1E1 |
| 1204 | 2011 | JN376784 | Vietnam | I | 1E | 1E1 |
| 1205 | 2011 | JX093655 | Vietnam | I | 1E | 1E1 |
| 1206 | 2011 | JX093658 | Vietnam | I | 1E | 1E1 |
| 1207 | 2011 | JX093669 | Vietnam | I | 1E | 1E1 |
| 1208 | 2011 | JX093674 | Vietnam | I | 1E | 1E1 |
| 1209 | 2011 | JX093677 | Vietnam | I | 1E | 1E1 |
| 1210 | 2011 | JX093688 | Vietnam | I | 1E | 1E1 |
| 1211 | 2011 | JX093697 | Vietnam | I | 1E | 1E1 |
| 1212 | 2011 | JX093703 | Vietnam | I | 1E | 1E1 |
| 1213 | 2011 | JX093719 | Vietnam | I | 1E | 1E1 |
| 1214 | 2011 | JX093722 | Vietnam | I | 1E | 1E1 |
| 1215 | 2011 | KY818253 | Vietnam | I | 1E | 1E1 |
| 1216 | 2011 | JX093660 | Vietnam | I | 1E | 1E1 |
| 1217 | 2011 | JX093665 | Vietnam | I | 1E | 1E1 |
| 1218 | 2011 | JX093705 | Vietnam | I | 1E | 1E1 |
| 1219 | 2011 | JX093656 | Vietnam | I | 1E | 1E1 |
| 1220 | 2011 | JX093664 | Vietnam | I | 1E | 1E1 |
| 1221 | 2011 | JX093668 | Vietnam | I | 1E | 1E1 |
| 1222 | 2011 | JX093670 | Vietnam | I | 1E | 1E1 |
| 1223 | 2011 | JX093671 | Vietnam | I | 1E | 1E1 |
| 1224 | 2011 | JX093673 | Vietnam | I | 1E | 1E1 |
| 1225 | 2011 | JX093676 | Vietnam | I | 1E | 1E1 |
| 1226 | 2011 | JX093678 | Vietnam | I | 1E | 1E1 |
| 1227 | 2011 | JX093681 | Vietnam | I | 1E | 1E1 |
| 1228 | 2011 | JX093682 | Vietnam | I | 1E | 1E1 |
| 1229 | 2011 | JX093686 | Vietnam | I | 1E | 1E1 |
| 1230 | 2011 | JX093689 | Vietnam | I | 1E | 1E1 |
| 1231 | 2011 | JX093690 | Vietnam | I | 1E | 1E1 |
| 1232 | 2011 | JX093692 | Vietnam | I | 1E | 1E1 |
| 1233 | 2011 | JX093700 | Vietnam | I | 1E | 1E1 |
| 1234 | 2011 | JX093704 | Vietnam | I | 1E | 1E1 |
| 1235 | 2011 | JX093706 | Vietnam | I | 1E | 1E1 |
| 1236 | 2011 | JX093707 | Vietnam | I | 1E | 1E1 |
| 1237 | 2011 | JX093709 | Vietnam | I | 1E | 1E1 |
| 1238 | 2011 | JX093717 | Vietnam | I | 1E | 1E1 |
| 1239 | 2011 | JX093720 | Vietnam | I | 1E | 1E1 |
| 1240 | 2011 | JX093723 | Vietnam | I | 1E | 1E1 |
| 1241 | 2011 | JX093724 | Vietnam | I | 1E | 1E1 |
| 1242 | 2011 | KY818249 | Vietnam | I | 1E | 1E1 |
| 1243 | 2011 | JX093667 | Vietnam | I | 1E | 1E1 |
| 1244 | 2011 | JX093680 | Vietnam | I | 1E | 1E1 |
| 1245 | 2011 | JX093687 | Vietnam | I | 1E | 1E1 |
| 1246 | 2011 | JX093710 | Vietnam | I | 1E | 1E1 |
| 1247 | 2011 | JX093659 | Vietnam | I | 1E | 1E1 |
| 1248 | 2011 | JX093662 | Vietnam | I | 1E | 1E1 |
| 1249 | 2011 | JX093666 | Vietnam | I | 1E | 1E1 |
| 1250 | 2011 | JX093675 | Vietnam | I | 1E | 1E1 |
| 1251 | 2011 | JX093679 | Vietnam | I | 1E | 1E1 |
| 1252 | 2011 | JX093685 | Vietnam | I | 1E | 1E1 |
| 1253 | 2011 | JX093691 | Vietnam | I | 1E | 1E1 |
| 1254 | 2011 | JX093696 | Vietnam | I | 1E | 1E1 |
| 1255 | 2011 | JX093702 | Vietnam | I | 1E | 1E1 |
| 1256 | 2011 | JX093711 | Vietnam | I | 1E | 1E1 |
| 1257 | 2011 | JX093713 | Vietnam | I | 1E | 1E1 |
| 1258 | 2011 | JX093714 | Vietnam | I | 1E | 1E1 |
| 1259 | 2011 | JX093715 | Vietnam | I | 1E | 1E1 |
| 1260 | 2011 | JX093716 | Vietnam | I | 1E | 1E1 |
| 1261 | 2011 | JX093721 | Vietnam | I | 1E | 1E1 |
| 1262 | 2012 | KY818251 | Vietnam | I | 1E | 1E1 |
| 1263 | 2012 | JX476086 | Vietnam | I | 1E | 1E1 |
| 1264 | 2012 | JX476089 | Vietnam | I | 1E | 1E1 |
| 1265 | 2012 | JX476091 | Vietnam | I | 1E | 1E1 |
| 1266 | 2012 | JX476093 | Vietnam | I | 1E | 1E1 |
| 1267 | 2012 | JX476094 | Vietnam | I | 1E | 1E1 |
| 1268 | 2012 | JX476095 | Vietnam | I | 1E | 1E1 |
| 1269 | 2012 | KY818252 | Vietnam | I | 1E | 1E1 |
| 1270 | 2012 | KY971714 | Vietnam | I | 1E | 1E1 |
| 1271 | 2012 | KC316019 | New_Caledonia | I | 1E | 1E1 |
| 1272 | 2012 | KC316020 | New_Caledonia | I | 1E | 1E1 |
| 1273 | 2012 | KC316021 | New_Caledonia | I | 1E | 1E1 |
| 1274 | 2012 | KC316022 | New_Caledonia | I | 1E | 1E1 |
| 1275 | 2012 | KC741438 | New_Caledonia | I | 1E | 1E1 |
| 1276 | 2012 | KC741439 | New_Caledonia | I | 1E | 1E1 |
| 1277 | 2012 | KC741440 | New_Caledonia | I | 1E | 1E1 |
| 1278 | 2012 | KC741441 | New_Caledonia | I | 1E | 1E1 |
| 1279 | 2012 | KC741442 | New_Caledonia | I | 1E | 1E1 |
| 1280 | 2012 | KC854412 | New_Caledonia | I | 1E | 1E1 |
| 1281 | 2012 | KC854413 | New_Caledonia | I | 1E | 1E1 |
| 1282 | 2012 | KC854414 | New_Caledonia | I | 1E | 1E1 |
| 1283 | 2012 | KC854415 | New_Caledonia | I | 1E | 1E1 |
| 1284 | 2012 | KU570095 | China | I | 1E | 1E1 |
| 1285 | 2012 | KY818248 | Vietnam | I | 1E | 1E1 |
| 1286 | 2012 | KY818250 | Vietnam | I | 1E | 1E1 |
| 1287 | 2012 | JX476087 | Vietnam | I | 1E | 1E1 |
| 1288 | 2012 | JX476088 | Vietnam | I | 1E | 1E1 |
| 1289 | 2012 | JX476092 | Vietnam | I | 1E | 1E1 |
| 1290 | 2012 | KY971713 | Vietnam | I | 1E | 1E1 |
| 1291 | 2012 | KY971715 | Vietnam | I | 1E | 1E1 |
| 1292 | 2012 | KY971716 | Vietnam | I | 1E | 1E1 |
| 1293 | 2013 | KJ806952 | Singapore | I | 1E | 1E1 |
| 1294 | 2013 | KY882516 | Vietnam | I | 1E | 1E1 |
| 1295 | 2013 | KY971717 | Vietnam | I | 1E | 1E1 |
| 1296 | 2013 | KT825012 | French_Polynesia | I | 1E | 1E1 |
| 1297 | 2013 | KX685333 | French_Polynesia | I | 1E | 1E1 |
| 1298 | 2013 | KX685334 | French_Polynesia | I | 1E | 1E1 |
| 1299 | 2013 | KX685335 | French_Polynesia | I | 1E | 1E1 |
| 1300 | 2013 | KX685336 | French_Polynesia | I | 1E | 1E1 |
| 1301 | 2013 | KX685337 | French_Polynesia | I | 1E | 1E1 |
| 1302 | 2013 | KY926848 | French_Polynesia | I | 1E | 1E1 |
| 1303 | 2013 | KX595191 | Vietnam | I | 1E | 1E1 |
| 1304 | 2014 | KT175105 | Vietnam | I | 1E | 1E1 |
| 1305 | 2014 | KT175106 | Vietnam | I | 1E | 1E1 |
| 1306 | 2014 | KU509313 | Singapore | I | 1E | 1E1 |
| 1307 | 2014 | KT825024 | NA | I | 1E | 1E1 |
| 1308 | 2014 | KT825033 | Vietnam | I | 1E | 1E1 |
| 1309 | 2014 | KX685338 | French_Polynesia | I | 1E | 1E1 |
| 1310 | 2014 | LC038148 | French_Polynesia | I | 1E | 1E1 |
| 1311 | 2014 | KU570102 | China | I | 1E | 1E1 |
| 1312 | 2015 | KT825044 | Vietnam | I | 1E | 1E1 |
| 1313 | 2015 | KY971719 | Vietnam | I | 1E | 1E1 |
| 1314 | 2015 | KT825035 | French_Polynesia | I | 1E | 1E1 |
| 1315 | 2015 | KY971718 | Vietnam | I | 1E | 1E1 |
| 1316 | 2016 | KY495793 | Vietnam | I | 1E | 1E1 |
| 1317 | 2003 | FJ639679 | Cambodia | I | 1F | 1F1 |
| 1318 | 2005 | FJ639683 | Cambodia | I | 1F | 1F1 |
| 1319 | 2005 | FJ639685 | Cambodia | I | 1F | 1F1 |
| 1320 | 2005 | GU131923 | Cambodia | I | 1F | 1F1 |
| 1321 | 2006 | EU482521 | Vietnam | I | 1F | 1F1 |
| 1322 | 2006 | FJ639686 | Cambodia | I | 1F | 1F1 |
| 1323 | 2006 | FJ744702 | Cambodia | I | 1F | 1F1 |
| 1324 | 2006 | GU131887 | Cambodia | I | 1F | 1F1 |
| 1325 | 2006 | GU131888 | Cambodia | I | 1F | 1F1 |
| 1326 | 2006 | GU131889 | Cambodia | I | 1F | 1F1 |
| 1327 | 2006 | GU131891 | Cambodia | I | 1F | 1F1 |
| 1328 | 2006 | GU131892 | Cambodia | I | 1F | 1F1 |
| 1329 | 2006 | GU131925 | Cambodia | I | 1F | 1F1 |
| 1330 | 2006 | HM181936 | Cambodia | I | 1F | 1F1 |
| 1331 | 2006 | HM181937 | Cambodia | I | 1F | 1F1 |
| 1332 | 2006 | HM181938 | Cambodia | I | 1F | 1F1 |
| 1333 | 2006 | HM181940 | Cambodia | I | 1F | 1F1 |
| 1334 | 2006 | HM181941 | Cambodia | I | 1F | 1F1 |
| 1335 | 2006 | JF269174 | Vietnam | I | 1F | 1F1 |
| 1336 | 2006 | JF269175 | Vietnam | I | 1F | 1F1 |
| 1337 | 2007 | EU448389 | Cambodia | I | 1F | 1F1 |
| 1338 | 2007 | FJ432745 | Vietnam | I | 1F | 1F1 |
| 1339 | 2007 | FJ461316 | Vietnam | I | 1F | 1F1 |
| 1340 | 2007 | FJ461319 | Vietnam | I | 1F | 1F1 |
| 1341 | 2007 | FJ461333 | Vietnam | I | 1F | 1F1 |
| 1342 | 2007 | FJ639689 | Cambodia | I | 1F | 1F1 |
| 1343 | 2007 | FJ639690 | Cambodia | I | 1F | 1F1 |
| 1344 | 2007 | FJ639692 | Cambodia | I | 1F | 1F1 |
| 1345 | 2007 | FJ639694 | Cambodia | I | 1F | 1F1 |
| 1346 | 2007 | FJ639695 | Cambodia | I | 1F | 1F1 |
| 1347 | 2007 | FJ882560 | Vietnam | I | 1F | 1F1 |
| 1348 | 2007 | GQ199825 | Vietnam | I | 1F | 1F1 |
| 1349 | 2007 | GU131893 | Cambodia | I | 1F | 1F1 |
| 1350 | 2007 | HM181943 | Cambodia | I | 1F | 1F1 |
| 1351 | 2007 | HM181946 | Cambodia | I | 1F | 1F1 |
| 1352 | 2007 | HM181947 | Cambodia | I | 1F | 1F1 |
| 1353 | 2007 | HM181948 | Cambodia | I | 1F | 1F1 |
| 1354 | 2007 | HM181949 | Cambodia | I | 1F | 1F1 |
| 1355 | 2007 | HM181950 | Cambodia | I | 1F | 1F1 |
| 1356 | 2007 | HM181953 | Cambodia | I | 1F | 1F1 |
| 1357 | 2007 | HM181954 | Cambodia | I | 1F | 1F1 |
| 1358 | 2007 | HM181955 | Cambodia | I | 1F | 1F1 |
| 1359 | 2007 | HM181956 | Cambodia | I | 1F | 1F1 |
| 1360 | 2007 | HM181957 | Cambodia | I | 1F | 1F1 |
| 1361 | 2007 | HM181958 | Cambodia | I | 1F | 1F1 |
| 1362 | 2007 | HM181959 | Cambodia | I | 1F | 1F1 |
| 1363 | 2007 | HM488255 | Cambodia | I | 1F | 1F1 |
| 1364 | 2007 | JN415496 | Cambodia | I | 1F | 1F1 |
| 1365 | 2007 | KF921932 | Cambodia | I | 1F | 1F1 |
| 1366 | 2007 | KF921933 | Cambodia | I | 1F | 1F1 |
| 1367 | 2007 | KF955444 | Cambodia | I | 1F | 1F1 |
| 1368 | 2008 | FJ410247 | Vietnam | I | 1F | 1F1 |
| 1369 | 2008 | GQ868632 | Cambodia | I | 1F | 1F1 |
| 1370 | 2008 | GQ868633 | Cambodia | I | 1F | 1F1 |
| 1371 | 2008 | GQ868635 | Cambodia | I | 1F | 1F1 |
| 1372 | 2008 | GU131703 | Vietnam | I | 1F | 1F1 |
| 1373 | 2008 | GU131708 | Vietnam | I | 1F | 1F1 |
| 1374 | 2008 | GU131728 | Vietnam | I | 1F | 1F1 |
| 1375 | 2008 | GU131771 | Vietnam | I | 1F | 1F1 |
| 1376 | 2008 | GU131774 | Vietnam | I | 1F | 1F1 |
| 1377 | 2008 | GU131777 | Vietnam | I | 1F | 1F1 |
| 1378 | 2008 | GU131779 | Vietnam | I | 1F | 1F1 |
| 1379 | 2008 | GU131788 | Vietnam | I | 1F | 1F1 |
| 1380 | 2008 | GU131789 | Vietnam | I | 1F | 1F1 |
| 1381 | 2008 | GU131800 | Vietnam | I | 1F | 1F1 |
| 1382 | 2008 | GU131810 | Vietnam | I | 1F | 1F1 |
| 1383 | 2008 | GU131814 | Vietnam | I | 1F | 1F1 |
| 1384 | 2008 | GU131822 | Vietnam | I | 1F | 1F1 |
| 1385 | 2008 | GU131826 | Vietnam | I | 1F | 1F1 |
| 1386 | 2008 | GU131894 | Cambodia | I | 1F | 1F1 |
| 1387 | 2008 | GU131919 | Cambodia | I | 1F | 1F1 |
| 1388 | 2008 | GU131920 | Cambodia | I | 1F | 1F1 |
| 1389 | 2008 | GU131921 | Cambodia | I | 1F | 1F1 |
| 1390 | 2008 | GU131922 | Cambodia | I | 1F | 1F1 |
| 1391 | 2008 | HQ591555 | Vietnam | I | 1F | 1F1 |
| 1392 | 2008 | JF937608 | Vietnam | I | 1F | 1F1 |
| 1393 | 2008 | JF937609 | Vietnam | I | 1F | 1F1 |
| 1394 | 2008 | JF937617 | Vietnam | I | 1F | 1F1 |
| 1395 | 2008 | JF937618 | Vietnam | I | 1F | 1F1 |
| 1396 | 2008 | JF937651 | Cambodia | I | 1F | 1F1 |
| 1397 | 2008 | JF967808 | Cambodia | I | 1F | 1F1 |
| 1398 | 2008 | KC861933 | Vietnam | I | 1F | 1F1 |
| 1399 | 2008 | KC861948 | Vietnam | I | 1F | 1F1 |
| 1400 | 2008 | KY971686 | Vietnam | I | 1F | 1F1 |
| 1401 | 2009 | GU131895 | Cambodia | I | 1F | 1F1 |
| 1402 | 2009 | JF967858 | Vietnam | I | 1F | 1F1 |
| 1403 | 2009 | JF967870 | Vietnam | I | 1F | 1F1 |
| 1404 | 2009 | JF967872 | Vietnam | I | 1F | 1F1 |
| 1405 | 2009 | JF967882 | Vietnam | I | 1F | 1F1 |
| 1406 | 2009 | JF967889 | Vietnam | I | 1F | 1F1 |
| 1407 | 2009 | JF967894 | Vietnam | I | 1F | 1F1 |
| 1408 | 2009 | KC861919 | Vietnam | I | 1F | 1F1 |
| 1409 | 2009 | KC861936 | Vietnam | I | 1F | 1F1 |
| 1410 | 2009 | KC861942 | Vietnam | I | 1F | 1F1 |
| 1411 | 2009 | KC861944 | Vietnam | I | 1F | 1F1 |
| 1412 | 2009 | KC861949 | Vietnam | I | 1F | 1F1 |
| 1413 | 2009 | KC861968 | Vietnam | I | 1F | 1F1 |
| 1414 | 2009 | KY849720 | Laos | I | 1F | 1F1 |
| 1415 | 2009 | KY971691 | Vietnam | I | 1F | 1F1 |
| 1416 | 2009 | KY971692 | Vietnam | I | 1F | 1F1 |
| 1417 | 2009 | KY971693 | Vietnam | I | 1F | 1F1 |
| 1418 | 2009 | KY971694 | Vietnam | I | 1F | 1F1 |
| 1419 | 2009 | KY971695 | Vietnam | I | 1F | 1F1 |
| 1420 | 2009 | KY971696 | Vietnam | I | 1F | 1F1 |
| 1421 | 2009 | KY971697 | Vietnam | I | 1F | 1F1 |
| 1422 | 2009 | KY971698 | Vietnam | I | 1F | 1F1 |
| 1423 | 2009 | KY971699 | Vietnam | I | 1F | 1F1 |
| 1424 | 2009 | KY971700 | Vietnam | I | 1F | 1F1 |
| 1425 | 2010 | JF967916 | Vietnam | I | 1F | 1F1 |
| 1426 | 2010 | JF967924 | Cambodia | I | 1F | 1F1 |
| 1427 | 2010 | JF967928 | Cambodia | I | 1F | 1F1 |
| 1428 | 2010 | JF967952 | Cambodia | I | 1F | 1F1 |
| 1429 | 2010 | JQ403520 | China | I | 1F | 1F1 |
| 1430 | 2010 | KC182093 | Laos | I | 1F | 1F1 |
| 1431 | 2010 | KC182094 | Laos | I | 1F | 1F1 |
| 1432 | 2010 | KY849743 | Laos | I | 1F | 1F1 |
| 1433 | 2010 | KY971701 | Vietnam | I | 1F | 1F1 |
| 1434 | 2010 | KY971703 | Vietnam | I | 1F | 1F1 |
| 1435 | 2010 | KY971704 | Vietnam | I | 1F | 1F1 |
| 1436 | 2010 | KY971709 | Vietnam | I | 1F | 1F1 |
| 1437 | 2010 | KY971710 | Vietnam | I | 1F | 1F1 |
| 1438 | 2010 | KY971711 | Vietnam | I | 1F | 1F1 |
| 1439 | 2010 | KY971712 | Vietnam | I | 1F | 1F1 |
| 1440 | 2011 | JX093661 | Vietnam | I | 1F | 1F1 |
| 1441 | 2011 | JX093694 | Vietnam | I | 1F | 1F1 |
| 1442 | 2011 | JX093698 | Vietnam | I | 1F | 1F1 |
| 1443 | 2011 | JX093701 | Vietnam | I | 1F | 1F1 |
| 1444 | 2011 | JX093708 | Vietnam | I | 1F | 1F1 |
| 1445 | 2011 | KT175107 | Cambodia | I | 1F | 1F1 |
| 1446 | 2012 | KC759167 | Suriname | I | 1F | 1F1 |
| 1447 | 2012 | KJ545450 | China | I | 1F | 1F1 |
| 1448 | 2012 | KT175078 | China | I | 1F | 1F1 |
| 1449 | 2012 | KT824985 | Cambodia | I | 1F | 1F1 |
| 1450 | 2012 | KT824990 | Papua_New_Guinea | I | 1F | 1F1 |
| 1451 | 2012 | KU509265 | NA | I | 1F | 1F1 |
| 1452 | 2012 | KX380798 | Singapore | I | 1F | 1F1 |
| 1453 | 2013 | AB873104 | Cambodia | I | 1F | 1F1 |
| 1454 | 2013 | KF887994 | Thailand | I | 1F | 1F1 |
| 1455 | 2013 | KT825017 | Vietnam | I | 1F | 1F1 |
| 1456 | 2013 | KT825055 | Cambodia | I | 1F | 1F1 |
| 1457 | 2001 | AY620946 | Myanmar | I | 1G | 1G1 |
| 1458 | 2001 | AY620947 | Myanmar | I | 1G | 1G1 |
| 1459 | 2001 | DQ264870 | Myanmar | I | 1G | 1G1 |
| 1460 | 2001 | DQ264883 | Myanmar | I | 1G | 1G1 |
| 1461 | 2001 | DQ264928 | Myanmar | I | 1G | 1G1 |
| 1462 | 2001 | DQ264929 | Myanmar | I | 1G | 1G1 |
| 1463 | 2001 | DQ264930 | Myanmar | I | 1G | 1G1 |
| 1464 | 2001 | DQ264931 | Myanmar | I | 1G | 1G1 |
| 1465 | 2001 | DQ264932 | Myanmar | I | 1G | 1G1 |
| 1466 | 2001 | DQ264933 | Myanmar | I | 1G | 1G1 |
| 1467 | 2001 | DQ264934 | Myanmar | I | 1G | 1G1 |
| 1468 | 2001 | DQ264935 | Myanmar | I | 1G | 1G1 |
| 1469 | 2001 | DQ264936 | Myanmar | I | 1G | 1G1 |
| 1470 | 2001 | DQ264937 | Myanmar | I | 1G | 1G1 |
| 1471 | 2001 | DQ264938 | Myanmar | I | 1G | 1G1 |
| 1472 | 2001 | DQ264939 | Myanmar | I | 1G | 1G1 |
| 1473 | 2001 | DQ264940 | Myanmar | I | 1G | 1G1 |
| 1474 | 2001 | DQ264941 | Myanmar | I | 1G | 1G1 |
| 1475 | 2001 | DQ264942 | Myanmar | I | 1G | 1G1 |
| 1476 | 2001 | DQ264944 | Myanmar | I | 1G | 1G1 |
| 1477 | 2001 | DQ264945 | Myanmar | I | 1G | 1G1 |
| 1478 | 2001 | DQ264946 | Myanmar | I | 1G | 1G1 |
| 1479 | 2001 | DQ264947 | Myanmar | I | 1G | 1G1 |
| 1480 | 2001 | EU117309 | Thailand | I | 1G | 1G1 |
| 1481 | 2001 | EU117310 | Thailand | I | 1G | 1G1 |
| 1482 | 2001 | EU117311 | Thailand | I | 1G | 1G1 |
| 1483 | 2001 | FJ687430 | Thailand | I | 1G | 1G1 |
| 1484 | 2001 | FJ687432 | Thailand | I | 1G | 1G1 |
| 1485 | 2001 | FJ687433 | Thailand | I | 1G | 1G1 |
| 1486 | 2002 | AY732386 | Thailand | I | 1G | 1G1 |
| 1487 | 2008 | JF967840 | Myanmar | I | 1G | 1G1 |
| 1488 | 2014 | KT827371 | China | I | 1G | 1G2 |
| 1489 | 2014 | KX056445 | China | I | 1G | 1G2 |
| 1490 | 2014 | KX056446 | China | I | 1G | 1G2 |
| 1491 | 2014 | KX056448 | China | I | 1G | 1G2 |
| 1492 | 2014 | KX056451 | China | I | 1G | 1G2 |
| 1493 | 2014 | KX056452 | China | I | 1G | 1G2 |
| 1494 | 2014 | KY038892 | China | I | 1G | 1G2 |
| 1495 | 2014 | KY038893 | China | I | 1G | 1G2 |
| 1496 | 2014 | KY038894 | China | I | 1G | 1G2 |
| 1497 | 2014 | KY038895 | China | I | 1G | 1G2 |
| 1498 | 2015 | KY234169 | Thailand | I | 1G | 1G2 |
| 1499 | 2015 | KY234170 | Thailand | I | 1G | 1G2 |
| 1500 | 2007 | KT175102 | Myanmar | I | 1G | 1G3 |
| 1501 | 2008 | JF967848 | Myanmar | I | 1G | 1G3 |
| 1502 | 2008 | JF967822 | Thailand | I | 1G | 1G3 |
| 1503 | 2015 | KX357962 | Myanmar | I | 1G | 1G3 |
| 1504 | 2011 | KT175077 | China | I | 1G | 1G4 |
| 1505 | 2013 | KR051909 | Myanmar | I | 1G | 1G4 |
| 1506 | 2013 | KR051908 | Myanmar | I | 1G | 1G4 |
| 1507 | 2015 | KX357952 | Myanmar | I | 1G | 1G4 |
| 1508 | 2009 | KT373893 | Thailand | I | 1G | 1G5 |
| 1509 | 2011 | KT175103 | Myanmar | I | 1G | 1G5 |
| 1510 | 2013 | KJ545447 | China | I | 1G | 1G5 |
| 1511 | 2013 | KJ545448 | China | I | 1G | 1G5 |
| 1512 | 2013 | KJ545453 | China | I | 1G | 1G5 |
| 1513 | 2013 | KJ545454 | China | I | 1G | 1G5 |
| 1514 | 2013 | KJ545455 | China | I | 1G | 1G5 |
| 1515 | 2013 | KJ545466 | China | I | 1G | 1G5 |
| 1516 | 2013 | KJ545467 | China | I | 1G | 1G5 |
| 1517 | 2013 | KR051929 | Myanmar | I | 1G | 1G5 |
| 1518 | 2013 | KR051930 | Myanmar | I | 1G | 1G5 |
| 1519 | 2013 | KT175079 | China | I | 1G | 1G5 |
| 1520 | 2013 | KU509290 | Thailand | I | 1G | 1G5 |
| 1521 | 2014 | KU509294 | Thailand | I | 1G | 1G5 |
| 1522 | 2014 | KX620454 | China | I | 1G | 1G5 |
| 1523 | 2014 | LC038146 | Myanmar | I | 1G | 1G5 |
| 1524 | 2015 | KX056467 | China | I | 1G | 1G5 |
| 1525 | 2015 | KX357943 | Myanmar | I | 1G | 1G5 |
| 1526 | 2001 | DQ264868 | Myanmar | I | 1G | 1G6 |
| 1527 | 2001 | DQ264869 | Myanmar | I | 1G | 1G6 |
| 1528 | 2001 | DQ264871 | Myanmar | I | 1G | 1G6 |
| 1529 | 2001 | DQ264872 | Myanmar | I | 1G | 1G6 |
| 1530 | 2001 | DQ264873 | Myanmar | I | 1G | 1G6 |
| 1531 | 2001 | DQ264874 | Myanmar | I | 1G | 1G6 |
| 1532 | 2001 | DQ264875 | Myanmar | I | 1G | 1G6 |
| 1533 | 2001 | DQ264876 | Myanmar | I | 1G | 1G6 |
| 1534 | 2001 | DQ264878 | Myanmar | I | 1G | 1G6 |
| 1535 | 2001 | DQ264879 | Myanmar | I | 1G | 1G6 |
| 1536 | 2001 | DQ264881 | Myanmar | I | 1G | 1G6 |
| 1537 | 1999 | AY620950 | Myanmar | I | 1G | 1G7 |
| 1538 | 2001 | DQ264917 | Myanmar | I | 1G | 1G7 |
| 1539 | 2001 | DQ264913 | Myanmar | I | 1G | 1G7 |
| 1540 | 2001 | DQ264909 | Myanmar | I | 1G | 1G7 |
| 1541 | 2001 | DQ264908 | Myanmar | I | 1G | 1G8 |
| 1542 | 2001 | DQ264910 | Myanmar | I | 1G | 1G8 |
| 1543 | 2001 | DQ264911 | Myanmar | I | 1G | 1G8 |
| 1544 | 2001 | DQ264912 | Myanmar | I | 1G | 1G8 |
| 1545 | 2001 | AY620949 | Myanmar | I | 1G | 1G8 |
| 1546 | 2001 | DQ264914 | Myanmar | I | 1G | 1G8 |
| 1547 | 2001 | DQ264915 | Myanmar | I | 1G | 1G8 |
| 1548 | 2001 | DQ264916 | Myanmar | I | 1G | 1G8 |
| 1549 | 2001 | DQ264918 | Myanmar | I | 1G | 1G8 |
| 1550 | 2001 | DQ264921 | Myanmar | I | 1G | 1G8 |
| 1551 | 2001 | DQ264922 | Myanmar | I | 1G | 1G8 |
| 1552 | 2001 | DQ264923 | Myanmar | I | 1G | 1G8 |
| 1553 | 2001 | DQ264924 | Myanmar | I | 1G | 1G8 |
| 1554 | 2001 | DQ264925 | Myanmar | I | 1G | 1G8 |
| 1555 | 2001 | DQ264926 | Myanmar | I | 1G | 1G8 |
| 1556 | 2001 | DQ264927 | Myanmar | I | 1G | 1G8 |
| 1557 | 2000 | AY618880 | Myanmar | I | 1G | 1G9 |
| 1558 | 2000 | AY620951 | Myanmar | I | 1G | 1G9 |
| 1559 | 2000 | AY620952 | Myanmar | I | 1G | 1G9 |
| 1560 | 2000 | DQ265078 | Myanmar | I | 1G | 1G9 |
| 1561 | 2000 | DQ265079 | Myanmar | I | 1G | 1G9 |
| 1562 | 2000 | DQ265080 | Myanmar | I | 1G | 1G9 |
| 1563 | 2000 | DQ265081 | Myanmar | I | 1G | 1G9 |
| 1564 | 2000 | DQ265082 | Myanmar | I | 1G | 1G9 |
| 1565 | 2000 | DQ265083 | Myanmar | I | 1G | 1G9 |
| 1566 | 2000 | DQ265084 | Myanmar | I | 1G | 1G9 |
| 1567 | 2000 | DQ265085 | Myanmar | I | 1G | 1G9 |
| 1568 | 2000 | DQ265086 | Myanmar | I | 1G | 1G9 |
| 1569 | 2000 | DQ265087 | Myanmar | I | 1G | 1G9 |
| 1570 | 2000 | DQ265088 | Myanmar | I | 1G | 1G9 |
| 1571 | 2000 | DQ265089 | Myanmar | I | 1G | 1G9 |
| 1572 | 2000 | DQ265090 | Myanmar | I | 1G | 1G9 |
| 1573 | 2000 | DQ265091 | Myanmar | I | 1G | 1G9 |
| 1574 | 2000 | DQ265092 | Myanmar | I | 1G | 1G9 |
| 1575 | 2000 | DQ265093 | Myanmar | I | 1G | 1G9 |
| 1576 | 2000 | DQ265094 | Myanmar | I | 1G | 1G9 |
| 1577 | 2000 | DQ265095 | Myanmar | I | 1G | 1G9 |
| 1578 | 2001 | AY620948 | Myanmar | I | 1G | 1G9 |
| 1579 | 2001 | AY620953 | Myanmar | I | 1G | 1G9 |
| 1580 | 2001 | AY708047 | Myanmar | I | 1G | 1G9 |
| 1581 | 2001 | AY713476 | Myanmar | I | 1G | 1G9 |
| 1582 | 2001 | DQ264880 | Myanmar | I | 1G | 1G9 |
| 1583 | 2001 | DQ264886 | Myanmar | I | 1G | 1G9 |
| 1584 | 2001 | DQ264887 | Myanmar | I | 1G | 1G9 |
| 1585 | 2001 | DQ264899 | Myanmar | I | 1G | 1G9 |
| 1586 | 2001 | DQ264989 | Myanmar | I | 1G | 1G9 |
| 1587 | 2001 | DQ265060 | Myanmar | I | 1G | 1G9 |
| 1588 | 2001 | DQ265064 | Myanmar | I | 1G | 1G9 |
| 1589 | 2001 | DQ265069 | Myanmar | I | 1G | 1G9 |
| 1590 | 2001 | DQ265072 | Myanmar | I | 1G | 1G9 |
| 1591 | 2001 | DQ265073 | Myanmar | I | 1G | 1G9 |
| 1592 | 2001 | DQ265075 | Myanmar | I | 1G | 1G9 |
| 1593 | 2001 | DQ265076 | Myanmar | I | 1G | 1G9 |
| 1594 | 2001 | DQ265077 | Myanmar | I | 1G | 1G9 |
| 1595 | 2002 | DQ264981 | Myanmar | I | 1G | 1G9 |
| 1596 | 2002 | DQ265023 | Myanmar | I | 1G | 1G9 |
| 1597 | 2002 | DQ265035 | Myanmar | I | 1G | 1G9 |
| 1598 | 2002 | DQ265040 | Myanmar | I | 1G | 1G9 |
| 1599 | 2002 | DQ265046 | Myanmar | I | 1G | 1G9 |
| 1600 | 2002 | DQ265051 | Myanmar | I | 1G | 1G9 |
| 1601 | 2002 | DQ265056 | Myanmar | I | 1G | 1G9 |
| 1602 | 2002 | DQ265128 | Myanmar | I | 1G | 1G9 |
| 1603 | 2002 | DQ265137 | Myanmar | I | 1G | 1G9 |
| 1604 | 2001 | AY606062 | Myanmar | I | 1G | 1G10 |
| 1605 | 2001 | AY618211 | Myanmar | I | 1G | 1G10 |
| 1606 | 2001 | AY726549 | Myanmar | I | 1G | 1G10 |
| 1607 | 2001 | AY726551 | Myanmar | I | 1G | 1G10 |
| 1608 | 2001 | DQ264884 | Myanmar | I | 1G | 1G10 |
| 1609 | 2001 | DQ264889 | Myanmar | I | 1G | 1G10 |
| 1610 | 2001 | DQ264891 | Myanmar | I | 1G | 1G10 |
| 1611 | 2001 | DQ264892 | Myanmar | I | 1G | 1G10 |
| 1612 | 2001 | DQ264893 | Myanmar | I | 1G | 1G10 |
| 1613 | 2001 | DQ264897 | Myanmar | I | 1G | 1G10 |
| 1614 | 2001 | DQ264898 | Myanmar | I | 1G | 1G10 |
| 1615 | 2001 | DQ264900 | Myanmar | I | 1G | 1G10 |
| 1616 | 2001 | DQ264902 | Myanmar | I | 1G | 1G10 |
| 1617 | 2001 | DQ264904 | Myanmar | I | 1G | 1G10 |
| 1618 | 2001 | DQ264905 | Myanmar | I | 1G | 1G10 |
| 1619 | 2001 | DQ264907 | Myanmar | I | 1G | 1G10 |
| 1620 | 2001 | DQ264991 | Myanmar | I | 1G | 1G10 |
| 1621 | 2001 | DQ264996 | Myanmar | I | 1G | 1G10 |
| 1622 | 2001 | DQ264999 | Myanmar | I | 1G | 1G10 |
| 1623 | 2001 | DQ265002 | Myanmar | I | 1G | 1G10 |
| 1624 | 2001 | DQ265004 | Myanmar | I | 1G | 1G10 |
| 1625 | 2001 | DQ265005 | Myanmar | I | 1G | 1G10 |
| 1626 | 2001 | DQ265007 | Myanmar | I | 1G | 1G10 |
| 1627 | 2001 | DQ265010 | Myanmar | I | 1G | 1G10 |
| 1628 | 2001 | DQ265057 | Myanmar | I | 1G | 1G10 |
| 1629 | 2001 | DQ265058 | Myanmar | I | 1G | 1G10 |
| 1630 | 2001 | DQ265061 | Myanmar | I | 1G | 1G10 |
| 1631 | 2001 | DQ265062 | Myanmar | I | 1G | 1G10 |
| 1632 | 2001 | DQ265063 | Myanmar | I | 1G | 1G10 |
| 1633 | 2001 | DQ265065 | Myanmar | I | 1G | 1G10 |
| 1634 | 2001 | DQ265066 | Myanmar | I | 1G | 1G10 |
| 1635 | 2001 | DQ265068 | Myanmar | I | 1G | 1G10 |
| 1636 | 2001 | DQ265070 | Myanmar | I | 1G | 1G10 |
| 1637 | 2001 | DQ265071 | Myanmar | I | 1G | 1G10 |
| 1638 | 2001 | DQ265074 | Myanmar | I | 1G | 1G10 |
| 1639 | 2001 | DQ265098 | Myanmar | I | 1G | 1G10 |
| 1640 | 2001 | DQ265101 | Myanmar | I | 1G | 1G10 |
| 1641 | 2001 | DQ265102 | Myanmar | I | 1G | 1G10 |
| 1642 | 2001 | DQ265104 | Myanmar | I | 1G | 1G10 |
| 1643 | 2001 | DQ265105 | Myanmar | I | 1G | 1G10 |
| 1644 | 2001 | DQ265107 | Myanmar | I | 1G | 1G10 |
| 1645 | 2001 | DQ265109 | Myanmar | I | 1G | 1G10 |
| 1646 | 2001 | DQ265111 | Myanmar | I | 1G | 1G10 |
| 1647 | 2001 | DQ265115 | Myanmar | I | 1G | 1G10 |
| 1648 | 2001 | DQ265117 | Myanmar | I | 1G | 1G10 |
| 1649 | 2002 | AY726553 | Myanmar | I | 1G | 1G10 |
| 1650 | 2002 | DQ264970 | Myanmar | I | 1G | 1G10 |
| 1651 | 2002 | DQ264971 | Myanmar | I | 1G | 1G10 |
| 1652 | 2002 | DQ264973 | Myanmar | I | 1G | 1G10 |
| 1653 | 2002 | DQ264974 | Myanmar | I | 1G | 1G10 |
| 1654 | 2002 | DQ264979 | Myanmar | I | 1G | 1G10 |
| 1655 | 2002 | DQ264980 | Myanmar | I | 1G | 1G10 |
| 1656 | 2002 | DQ264982 | Myanmar | I | 1G | 1G10 |
| 1657 | 2002 | DQ264983 | Myanmar | I | 1G | 1G10 |
| 1658 | 2002 | DQ264984 | Myanmar | I | 1G | 1G10 |
| 1659 | 2002 | DQ265018 | Myanmar | I | 1G | 1G10 |
| 1660 | 2002 | DQ265019 | Myanmar | I | 1G | 1G10 |
| 1661 | 2002 | DQ265020 | Myanmar | I | 1G | 1G10 |
| 1662 | 2002 | DQ265022 | Myanmar | I | 1G | 1G10 |
| 1663 | 2002 | DQ265026 | Myanmar | I | 1G | 1G10 |
| 1664 | 2002 | DQ265030 | Myanmar | I | 1G | 1G10 |
| 1665 | 2002 | DQ265031 | Myanmar | I | 1G | 1G10 |
| 1666 | 2002 | DQ265039 | Myanmar | I | 1G | 1G10 |
| 1667 | 2002 | DQ265041 | Myanmar | I | 1G | 1G10 |
| 1668 | 2002 | DQ265044 | Myanmar | I | 1G | 1G10 |
| 1669 | 2002 | DQ265045 | Myanmar | I | 1G | 1G10 |
| 1670 | 2002 | DQ265047 | Myanmar | I | 1G | 1G10 |
| 1671 | 2002 | DQ265048 | Myanmar | I | 1G | 1G10 |
| 1672 | 2002 | DQ265049 | Myanmar | I | 1G | 1G10 |
| 1673 | 2002 | DQ265050 | Myanmar | I | 1G | 1G10 |
| 1674 | 2002 | DQ265052 | Myanmar | I | 1G | 1G10 |
| 1675 | 2002 | DQ265053 | Myanmar | I | 1G | 1G10 |
| 1676 | 2002 | DQ265054 | Myanmar | I | 1G | 1G10 |
| 1677 | 2002 | DQ265055 | Myanmar | I | 1G | 1G10 |
| 1678 | 2002 | DQ265119 | Myanmar | I | 1G | 1G10 |
| 1679 | 2002 | DQ265121 | Myanmar | I | 1G | 1G10 |
| 1680 | 2002 | DQ265123 | Myanmar | I | 1G | 1G10 |
| 1681 | 2002 | DQ265132 | Myanmar | I | 1G | 1G10 |
| 1682 | 2002 | DQ265136 | Myanmar | I | 1G | 1G10 |
| 1683 | 2002 | DQ265138 | Myanmar | I | 1G | 1G10 |
| 1684 | 2002 | DQ265139 | Myanmar | I | 1G | 1G10 |
| 1685 | 2002 | DQ265143 | Myanmar | I | 1G | 1G10 |
| 1686 | 2002 | DQ265145 | Myanmar | I | 1G | 1G10 |
| 1687 | 2002 | DQ265147 | Myanmar | I | 1G | 1G10 |
| 1688 | 2002 | DQ265148 | Myanmar | I | 1G | 1G10 |
| 1689 | 2002 | DQ265152 | Myanmar | I | 1G | 1G10 |
| 1690 | 2002 | DQ265155 | Myanmar | I | 1G | 1G10 |
| 1691 | 2002 | DQ265156 | Myanmar | I | 1G | 1G10 |
| 1692 | 2001 | AB111072 | Thailand | I | 1H | 1H1 |
| 1693 | 2001 | AY732389 | Thailand | I | 1H | 1H1 |
| 1694 | 2001 | AY732392 | Thailand | I | 1H | 1H1 |
| 1695 | 2001 | AY732401 | Thailand | I | 1H | 1H1 |
| 1696 | 2001 | AY732419 | Thailand | I | 1H | 1H1 |
| 1697 | 2001 | AY732479 | Thailand | I | 1H | 1H1 |
| 1698 | 2001 | AY732482 | Thailand | I | 1H | 1H1 |
| 1699 | 2001 | EF508200 | China | I | 1H | 1H1 |
| 1700 | 2001 | EU117304 | Thailand | I | 1H | 1H1 |
| 1701 | 2001 | EU117305 | Thailand | I | 1H | 1H1 |
| 1702 | 2001 | EU117306 | Thailand | I | 1H | 1H1 |
| 1703 | 2001 | EU117307 | Thailand | I | 1H | 1H1 |
| 1704 | 2001 | EU117308 | Thailand | I | 1H | 1H1 |
| 1705 | 2001 | EU117312 | Thailand | I | 1H | 1H1 |
| 1706 | 2001 | FJ687426 | Thailand | I | 1H | 1H1 |
| 1707 | 2001 | FJ687427 | Thailand | I | 1H | 1H1 |
| 1708 | 2001 | FJ687428 | Thailand | I | 1H | 1H1 |
| 1709 | 2001 | FJ687429 | Thailand | I | 1H | 1H1 |
| 1710 | 2001 | FJ687431 | Thailand | I | 1H | 1H1 |
| 1711 | 2001 | FJ850068 | Thailand | I | 1H | 1H1 |
| 1712 | 2001 | JQ317752 | China | I | 1H | 1H1 |
| 1713 | 2001 | JQ317753 | China | I | 1H | 1H1 |
| 1714 | 2002 | AB111078 | Thailand | I | 1H | 1H1 |
| 1715 | 2002 | AY732398 | Thailand | I | 1H | 1H1 |
| 1716 | 2003 | EU448393 | Thailand | I | 1H | 1H1 |
| 1717 | 2003 | HM134239 | Thailand | I | 1H | 1H1 |
| 1718 | 2003 | JN556045 | Thailand | I | 1H | 1H1 |
| 1719 | 2003 | JN638332 | Thailand | I | 1H | 1H1 |
| 1720 | 2004 | AY835999 | China | I | 1H | 1H1 |
| 1721 | 2004 | AY871812 | China | I | 1H | 1H1 |
| 1722 | 2004 | DQ836632 | China | I | 1H | 1H1 |
| 1723 | 2005 | EF654109 | Thailand | I | 1H | 1H1 |
| 1724 | 2005 | JN638324 | Thailand | I | 1H | 1H1 |
| 1725 | 2005 | JN638333 | Thailand | I | 1H | 1H1 |
| 1726 | 2005 | JQ993131 | Thailand | I | 1H | 1H1 |
| 1727 | 2005 | JQ993194 | Thailand | I | 1H | 1H1 |
| 1728 | 2006 | JN638325 | Thailand | I | 1H | 1H1 |
| 1729 | 2006 | JQ993171 | Thailand | I | 1H | 1H1 |
| 1730 | 2006 | JQ993193 | Thailand | I | 1H | 1H1 |
| 1731 | 2007 | EU448394 | Thailand | I | 1H | 1H1 |
| 1732 | 2007 | EU448395 | Malaysia | I | 1H | 1H1 |
| 1733 | 2007 | GQ357689 | Singapore | I | 1H | 1H1 |
| 1734 | 2007 | JQ993148 | Thailand | I | 1H | 1H1 |
| 1735 | 2007 | JQ993150 | Thailand | I | 1H | 1H1 |
| 1736 | 2007 | KC182097 | Laos | I | 1H | 1H1 |
| 1737 | 2007 | KC182101 | Laos | I | 1H | 1H1 |
| 1738 | 2008 | AB608787 | China | I | 1H | 1H1 |
| 1739 | 2008 | JN029815 | China | I | 1H | 1H1 |
| 1740 | 2008 | JQ317734 | China | I | 1H | 1H1 |
| 1741 | 2008 | JQ317735 | China | I | 1H | 1H1 |
| 1742 | 2008 | JQ403517 | China | I | 1H | 1H1 |
| 1743 | 2008 | KC172834 | Laos | I | 1H | 1H1 |
| 1744 | 2008 | KY849741 | Laos | I | 1H | 1H1 |
| 1745 | 2011 | AB111067 | NA | I | 1H | 1H1 |
| 1746 | 2011 | JQ317754 | China | I | 1H | 1H1 |
| 1747 | 2005 | EF654108 | Thailand | I | 1H | 1H2 |
| 1748 | 2006 | EU482811 | Vietnam | I | 1H | 1H2 |
| 1749 | 2007 | FJ410212 | Vietnam | I | 1H | 1H2 |
| 1750 | 2007 | FJ461328 | Vietnam | I | 1H | 1H2 |
| 1751 | 2007 | HM181952 | Cambodia | I | 1H | 1H2 |
| 1752 | 2008 | FJ410220 | Vietnam | I | 1H | 1H2 |
| 1753 | 2008 | FJ410286 | Vietnam | I | 1H | 1H2 |
| 1754 | 2008 | FJ461307 | Vietnam | I | 1H | 1H2 |
| 1755 | 2008 | GU131678 | Vietnam | I | 1H | 1H2 |
| 1756 | 2008 | GU131680 | Vietnam | I | 1H | 1H2 |
| 1757 | 2008 | GU131709 | Vietnam | I | 1H | 1H2 |
| 1758 | 2008 | GU131711 | Vietnam | I | 1H | 1H2 |
| 1759 | 2008 | GU131712 | Vietnam | I | 1H | 1H2 |
| 1760 | 2008 | GU131713 | Vietnam | I | 1H | 1H2 |
| 1761 | 2008 | GU131736 | Vietnam | I | 1H | 1H2 |
| 1762 | 2008 | GU131749 | Vietnam | I | 1H | 1H2 |
| 1763 | 2008 | GU131764 | Vietnam | I | 1H | 1H2 |
| 1764 | 2008 | GU131773 | Vietnam | I | 1H | 1H2 |
| 1765 | 2008 | GU131801 | Vietnam | I | 1H | 1H2 |
| 1766 | 2008 | GU131802 | Vietnam | I | 1H | 1H2 |
| 1767 | 2008 | GU131829 | Vietnam | I | 1H | 1H2 |
| 1768 | 2008 | GU131830 | Vietnam | I | 1H | 1H2 |
| 1769 | 2008 | HM181965 | Vietnam | I | 1H | 1H2 |
| 1770 | 2008 | JF937619 | Vietnam | I | 1H | 1H2 |
| 1771 | 2008 | JF967805 | Vietnam | I | 1H | 1H2 |
| 1772 | 2008 | JF967843 | Vietnam | I | 1H | 1H2 |
| 1773 | 2008 | KY971689 | Vietnam | I | 1H | 1H2 |
| 1774 | 2007 | FJ687474 | Thailand | I | 1H | 1H3 |
| 1775 | 2008 | KY849706 | Laos | I | 1H | 1H3 |
| 1776 | 2009 | KY849724 | Laos | I | 1H | 1H3 |
| 1777 | 2009 | KY849725 | Laos | I | 1H | 1H3 |
| 1778 | 2009 | KY849726 | Laos | I | 1H | 1H3 |
| 1779 | 2009 | KY849728 | Laos | I | 1H | 1H3 |
| 1780 | 2009 | KY849729 | Laos | I | 1H | 1H3 |
| 1781 | 2009 | KY849746 | Laos | I | 1H | 1H3 |
| 1782 | 2010 | JF967921 | Thailand | I | 1H | 1H3 |
| 1783 | 2010 | JF967940 | Thailand | I | 1H | 1H3 |
| 1784 | 2010 | JN029808 | China | I | 1H | 1H3 |
| 1785 | 2010 | JQ896294 | NA | I | 1H | 1H3 |
| 1786 | 2010 | KC182082 | Laos | I | 1H | 1H3 |
| 1787 | 2010 | KC182083 | Laos | I | 1H | 1H3 |
| 1788 | 2010 | KC182084 | Laos | I | 1H | 1H3 |
| 1789 | 2010 | KC182095 | Laos | I | 1H | 1H3 |
| 1790 | 2010 | KC182108 | Laos | I | 1H | 1H3 |
| 1791 | 2010 | KC182109 | Laos | I | 1H | 1H3 |
| 1792 | 2010 | KC182110 | Laos | I | 1H | 1H3 |
| 1793 | 2010 | KC182111 | Laos | I | 1H | 1H3 |
| 1794 | 2010 | KC182112 | Laos | I | 1H | 1H3 |
| 1795 | 2010 | KC182113 | Laos | I | 1H | 1H3 |
| 1796 | 2010 | KU570094 | China | I | 1H | 1H3 |
| 1797 | 2010 | KY849702 | Laos | I | 1H | 1H3 |
| 1798 | 2010 | KY849703 | Laos | I | 1H | 1H3 |
| 1799 | 2010 | KY849730 | Laos | I | 1H | 1H3 |
| 1800 | 2010 | KY849731 | Laos | I | 1H | 1H3 |
| 1801 | 2010 | KY849732 | Laos | I | 1H | 1H3 |
| 1802 | 2010 | KY849733 | Laos | I | 1H | 1H3 |
| 1803 | 2010 | KY849734 | Laos | I | 1H | 1H3 |
| 1804 | 2010 | KY849735 | Laos | I | 1H | 1H3 |
| 1805 | 2010 | KY849736 | Laos | I | 1H | 1H3 |
| 1806 | 2010 | KY849737 | Laos | I | 1H | 1H3 |
| 1807 | 2010 | KY849738 | Laos | I | 1H | 1H3 |
| 1808 | 2010 | KY849739 | Laos | I | 1H | 1H3 |
| 1809 | 2010 | KY849747 | Laos | I | 1H | 1H3 |
| 1810 | 2011 | KF926700 | Laos | I | 1H | 1H3 |
| 1811 | 2006 | EF113152 | China | I | 1H | 1H4 |
| 1812 | 2006 | EF113153 | China | I | 1H | 1H4 |
| 1813 | 2006 | EF508205 | China | I | 1H | 1H4 |
| 1814 | 2006 | FJ176779 | China | I | 1H | 1H4 |
| 1815 | 2006 | FJ196844 | China | I | 1H | 1H4 |
| 1816 | 2006 | FJ196855 | China | I | 1H | 1H4 |
| 1817 | 2006 | FJ196856 | China | I | 1H | 1H4 |
| 1818 | 2006 | FJ196857 | China | I | 1H | 1H4 |
| 1819 | 2006 | FJ196858 | China | I | 1H | 1H4 |
| 1820 | 2006 | FJ196859 | China | I | 1H | 1H4 |
| 1821 | 2006 | FJ196860 | China | I | 1H | 1H4 |
| 1822 | 2006 | JQ993127 | Thailand | I | 1H | 1H4 |
| 1823 | 2006 | JQ993130 | Thailand | I | 1H | 1H4 |
| 1824 | 2006 | JQ993177 | Thailand | I | 1H | 1H4 |
| 1825 | 2006 | JQ993183 | Thailand | I | 1H | 1H4 |
| 1826 | 2006 | JQ993184 | Thailand | I | 1H | 1H4 |
| 1827 | 2006 | JQ993188 | Thailand | I | 1H | 1H4 |
| 1828 | 2006 | JQ993189 | Thailand | I | 1H | 1H4 |
| 1829 | 2006 | JQ993191 | Thailand | I | 1H | 1H4 |
| 1830 | 2006 | JQ993192 | Thailand | I | 1H | 1H4 |
| 1831 | 2006 | JQ993203 | Thailand | I | 1H | 1H4 |
| 1832 | 2006 | KT827365 | China | I | 1H | 1H4 |
| 1833 | 2006 | KX225491 | China | I | 1H | 1H4 |
| 1834 | 2007 | EU280167 | China | I | 1H | 1H4 |
| 1835 | 2007 | JQ993108 | Thailand | I | 1H | 1H4 |
| 1836 | 2007 | JQ993132 | Thailand | I | 1H | 1H4 |
| 1837 | 2007 | JQ993133 | Thailand | I | 1H | 1H4 |
| 1838 | 2007 | JQ993198 | Thailand | I | 1H | 1H4 |
| 1839 | 2008 | JF967810 | Myanmar | I | 1H | 1H4 |
| 1840 | 2008 | JN415527 | Thailand | I | 1H | 1H4 |
| 1841 | 2009 | JF967879 | Thailand | I | 1H | 1H4 |
| 1842 | 2013 | KJ470713 | China | I | 1H | 1H4 |
| 1843 | 2013 | KJ470715 | China | I | 1H | 1H4 |
| 1844 | 2013 | KJ470718 | China | I | 1H | 1H4 |
| 1845 | 2013 | KJ470719 | China | I | 1H | 1H4 |
| 1846 | 2013 | KJ470720 | China | I | 1H | 1H4 |
| 1847 | 2013 | KJ470721 | China | I | 1H | 1H4 |
| 1848 | 2013 | KJ470723 | China | I | 1H | 1H4 |
| 1849 | 2013 | KJ470727 | China | I | 1H | 1H4 |
| 1850 | 2013 | KJ470738 | Myanmar | I | 1H | 1H4 |
| 1851 | 2013 | KJ470740 | Myanmar | I | 1H | 1H4 |
| 1852 | 2013 | KR051926 | Myanmar | I | 1H | 1H4 |
| 1853 | 2013 | KU570100 | China | I | 1H | 1H4 |
| 1854 | 2013 | KX056460 | China | I | 1H | 1H4 |
| 1855 | 2013 | KY038884 | China | I | 1H | 1H4 |
| 1856 | 2013 | KY038885 | China | I | 1H | 1H4 |
| 1857 | 2013 | KJ470712 | China | I | 1H | 1H4 |
| 1858 | 2013 | KJ470714 | China | I | 1H | 1H4 |
| 1859 | 2013 | KJ470716 | China | I | 1H | 1H4 |
| 1860 | 2013 | KJ470728 | China | I | 1H | 1H4 |
| 1861 | 2013 | KJ470731 | China | I | 1H | 1H4 |
| 1862 | 2013 | KJ470732 | China | I | 1H | 1H4 |
| 1863 | 2013 | KJ470733 | China | I | 1H | 1H4 |
| 1864 | 2013 | KJ470734 | China | I | 1H | 1H4 |
| 1865 | 2013 | KJ470736 | China | I | 1H | 1H4 |
| 1866 | 2013 | KJ470737 | China | I | 1H | 1H4 |
| 1867 | 2013 | KJ470739 | Myanmar | I | 1H | 1H4 |
| 1868 | 2013 | KJ470741 | Myanmar | I | 1H | 1H4 |
| 1869 | 2013 | KR051922 | Myanmar | I | 1H | 1H4 |
| 1870 | 2013 | KR051923 | Myanmar | I | 1H | 1H4 |
| 1871 | 2013 | KR051924 | Myanmar | I | 1H | 1H4 |
| 1872 | 2013 | KR051925 | Myanmar | I | 1H | 1H4 |
| 1873 | 2013 | KT825001 | Myanmar | I | 1H | 1H4 |
| 1874 | 2013 | KX225490 | China | I | 1H | 1H4 |
| 1875 | 2013 | KY038882 | China | I | 1H | 1H4 |
| 1876 | 2013 | KY038883 | China | I | 1H | 1H4 |
| 1877 | 2013 | KJ806941 | Singapore | I | 1H | 1H4 |
| 1878 | 2013 | KR051920 | Myanmar | I | 1H | 1H4 |
| 1879 | 2013 | KR051921 | Myanmar | I | 1H | 1H4 |
| 1880 | 2013 | KR051927 | Myanmar | I | 1H | 1H4 |
| 1881 | 2013 | KR051928 | Myanmar | I | 1H | 1H4 |
| 1882 | 2014 | KT175104 | Myanmar | I | 1H | 1H4 |
| 1883 | 2015 | KX357970 | Myanmar | I | 1H | 1H4 |
| 1884 | 2015 | KX056462 | China | I | 1H | 1H4 |
| 1885 | 2015 | KX056463 | China | I | 1H | 1H4 |
| 1886 | 2015 | KX056464 | China | I | 1H | 1H4 |
| 1887 | 2015 | KX056465 | China | I | 1H | 1H4 |
| 1888 | 2015 | KX056468 | China | I | 1H | 1H4 |
| 1889 | 2015 | KX056471 | China | I | 1H | 1H4 |
| 1890 | 2015 | KX357933 | Myanmar | I | 1H | 1H4 |
| 1891 | 2015 | KX357953 | Myanmar | I | 1H | 1H4 |
| 1892 | 2015 | KX357958 | Myanmar | I | 1H | 1H4 |
| 1893 | 2015 | KX056456 | China | I | 1H | 1H4 |
| 1894 | 2015 | KX056457 | China | I | 1H | 1H4 |
| 1895 | 2015 | KX056458 | China | I | 1H | 1H4 |
| 1896 | 2015 | KX056459 | China | I | 1H | 1H4 |
| 1897 | 2015 | KX056466 | China | I | 1H | 1H4 |
| 1898 | 2015 | KX056470 | China | I | 1H | 1H4 |
| 1899 | 2015 | KX357917 | Myanmar | I | 1H | 1H4 |
| 1900 | 2015 | KX357927 | Myanmar | I | 1H | 1H4 |
| 1901 | 2015 | KX357930 | Myanmar | I | 1H | 1H4 |
| 1902 | 2015 | KX357935 | Myanmar | I | 1H | 1H4 |
| 1903 | 2015 | KX357938 | Myanmar | I | 1H | 1H4 |
| 1904 | 2015 | KX357940 | Myanmar | I | 1H | 1H4 |
| 1905 | 2015 | KX357942 | Myanmar | I | 1H | 1H4 |
| 1906 | 2015 | KX357946 | Myanmar | I | 1H | 1H4 |
| 1907 | 2015 | KX357949 | Myanmar | I | 1H | 1H4 |
| 1908 | 2015 | KX357954 | Myanmar | I | 1H | 1H4 |
| 1909 | 2015 | KX357959 | Myanmar | I | 1H | 1H4 |
| 1910 | 2015 | KX357963 | Myanmar | I | 1H | 1H4 |
| 1911 | 2015 | KX357967 | Myanmar | I | 1H | 1H4 |
| 1912 | 2015 | KY038886 | China | I | 1H | 1H4 |
| 1913 | 2015 | KY495792 | Thailand | I | 1H | 1H4 |
| 1914 | 2006 | EF508204 | China | I | 1H | 1H5 |
| 1915 | 2006 | JQ317743 | China | I | 1H | 1H5 |
| 1916 | 2006 | JQ317744 | China | I | 1H | 1H5 |
| 1917 | 2006 | JQ317745 | China | I | 1H | 1H5 |
| 1918 | 2009 | HQ891313 | Sri_Lanka | I | 1H | 1H5 |
| 1919 | 2009 | HQ891314 | Sri_Lanka | I | 1H | 1H5 |
| 1920 | 2009 | HQ891315 | Sri_Lanka | I | 1H | 1H5 |
| 1921 | 2009 | HQ891316 | Sri_Lanka | I | 1H | 1H5 |
| 1922 | 2009 | JF960217 | Singapore | I | 1H | 1H5 |
| 1923 | 2009 | JF960219 | Singapore | I | 1H | 1H5 |
| 1924 | 2009 | JN054256 | Sri_Lanka | I | 1H | 1H5 |
| 1925 | 2009 | KT373894 | Thailand | I | 1H | 1H5 |
| 1926 | 2009 | KT373895 | Thailand | I | 1H | 1H5 |
| 1927 | 2009 | JN638327 | Thailand | I | 1H | 1H5 |
| 1928 | 2009 | KT373902 | Thailand | I | 1H | 1H5 |
| 1929 | 2010 | JF960220 | Singapore | I | 1H | 1H5 |
| 1930 | 2010 | JN054255 | Sri_Lanka | I | 1H | 1H5 |
| 1931 | 2011 | KT824979 | Sri_Lanka | I | 1H | 1H5 |
| 1932 | 2011 | KT824980 | Sri_Lanka | I | 1H | 1H5 |
| 1933 | 2011 | KT825049 | Sri_Lanka | I | 1H | 1H5 |
| 1934 | 2011 | LC148030 | Maldives | I | 1H | 1H5 |
| 1935 | 2012 | KJ726662 | Sri_Lanka | I | 1H | 1H5 |
| 1936 | 2012 | KJ726663 | Sri_Lanka | I | 1H | 1H5 |
| 1937 | 2012 | KJ726664 | Sri_Lanka | I | 1H | 1H5 |
| 1938 | 2012 | KJ726665 | Sri_Lanka | I | 1H | 1H5 |
| 1939 | 2012 | KR527488 | India | I | 1H | 1H5 |
| 1940 | 2012 | KY978432 | India | I | 1H | 1H5 |
| 1941 | 2012 | KY978433 | India | I | 1H | 1H5 |
| 1942 | 2012 | KY978434 | India | I | 1H | 1H5 |
| 1943 | 2012 | KY978435 | India | I | 1H | 1H5 |
| 1944 | 2012 | KY978436 | India | I | 1H | 1H5 |
| 1945 | 2012 | KY978437 | India | I | 1H | 1H5 |
| 1946 | 2012 | KY978438 | India | I | 1H | 1H5 |
| 1947 | 2012 | KT824992 | NA | I | 1H | 1H5 |
| 1948 | 2012 | KT824994 | Thailand | I | 1H | 1H5 |
| 1949 | 2012 | KT824995 | Thailand | I | 1H | 1H5 |
| 1950 | 2012 | KU509250 | Thailand | I | 1H | 1H5 |
| 1951 | 2013 | KJ415097 | Sri_Lanka | I | 1H | 1H5 |
| 1952 | 2013 | KJ468234 | Sri_Lanka | I | 1H | 1H5 |
| 1953 | 2013 | KJ755855 | India | I | 1H | 1H5 |
| 1954 | 2013 | KR527487 | India | I | 1H | 1H5 |
| 1955 | 2013 | KT825008 | Papua_New_Guinea | I | 1H | 1H5 |
| 1956 | 2013 | KU509310 | Sri_Lanka | I | 1H | 1H5 |
| 1957 | 2013 | KJ470722 | China | I | 1H | 1H5 |
| 1958 | 2013 | KJ470724 | China | I | 1H | 1H5 |
| 1959 | 2013 | KJ470725 | China | I | 1H | 1H5 |
| 1960 | 2013 | KJ470726 | China | I | 1H | 1H5 |
| 1961 | 2013 | KJ470729 | China | I | 1H | 1H5 |
| 1962 | 2013 | KJ470735 | China | I | 1H | 1H5 |
| 1963 | 2013 | KJ470742 | Myanmar | I | 1H | 1H5 |
| 1964 | 2013 | KJ545443 | China | I | 1H | 1H5 |
| 1965 | 2013 | KJ545444 | China | I | 1H | 1H5 |
| 1966 | 2013 | KJ545482 | Thailand | I | 1H | 1H5 |
| 1967 | 2013 | KJ806946 | Singapore | I | 1H | 1H5 |
| 1968 | 2013 | KR051910 | Myanmar | I | 1H | 1H5 |
| 1969 | 2013 | KR051911 | Myanmar | I | 1H | 1H5 |
| 1970 | 2013 | KR051912 | Myanmar | I | 1H | 1H5 |
| 1971 | 2013 | KR051913 | Myanmar | I | 1H | 1H5 |
| 1972 | 2013 | KR051914 | Myanmar | I | 1H | 1H5 |
| 1973 | 2013 | KR051915 | Myanmar | I | 1H | 1H5 |
| 1974 | 2013 | KR051916 | Myanmar | I | 1H | 1H5 |
| 1975 | 2013 | KR051917 | Myanmar | I | 1H | 1H5 |
| 1976 | 2013 | KR051918 | Myanmar | I | 1H | 1H5 |
| 1977 | 2013 | KR051919 | Myanmar | I | 1H | 1H5 |
| 1978 | 2013 | KT825003 | Australia | I | 1H | 1H5 |
| 1979 | 2013 | KT825004 | Australia | I | 1H | 1H5 |
| 1980 | 2013 | KT825005 | Australia | I | 1H | 1H5 |
| 1981 | 2013 | KT825014 | Thailand | I | 1H | 1H5 |
| 1982 | 2013 | KT825016 | Australia | I | 1H | 1H5 |
| 1983 | 2013 | KT825018 | Australia | I | 1H | 1H5 |
| 1984 | 2013 | KT825058 | Thailand | I | 1H | 1H5 |
| 1985 | 2014 | KP398852 | Sri_Lanka | I | 1H | 1H5 |
| 1986 | 2014 | KT825027 | Sri_Lanka | I | 1H | 1H5 |
| 1987 | 2014 | KT825028 | Sri_Lanka | I | 1H | 1H5 |
| 1988 | 2014 | KT825029 | Sri_Lanka | I | 1H | 1H5 |
| 1989 | 2014 | KT825030 | Sri_Lanka | I | 1H | 1H5 |
| 1990 | 2014 | LC038143 | Sri_Lanka | I | 1H | 1H5 |
| 1991 | 2014 | KX056447 | China | I | 1H | 1H5 |
| 1992 | 2014 | KX056449 | China | I | 1H | 1H5 |
| 1993 | 2014 | KX056450 | China | I | 1H | 1H5 |
| 1994 | 2014 | KX056453 | China | I | 1H | 1H5 |
| 1995 | 2014 | KX056454 | China | I | 1H | 1H5 |
| 1996 | 2014 | KX056455 | China | I | 1H | 1H5 |
| 1997 | 2014 | KY038887 | China | I | 1H | 1H5 |
| 1998 | 2014 | KY038888 | China | I | 1H | 1H5 |
| 1999 | 2014 | KY038889 | China | I | 1H | 1H5 |
| 2000 | 2015 | KY021894 | India | I | 1H | 1H5 |
| 2001 | 2015 | KY978439 | India | I | 1H | 1H5 |
| 2002 | 2015 | KX056469 | China | I | 1H | 1H5 |
| 2003 | 2015 | KX056472 | China | I | 1H | 1H5 |
| 2004 | 2015 | KX056473 | China | I | 1H | 1H5 |
| 2005 | 2015 | KX357900 | Myanmar | I | 1H | 1H5 |
| 2006 | 2015 | KX357901 | Myanmar | I | 1H | 1H5 |
| 2007 | 2015 | KX357902 | Myanmar | I | 1H | 1H5 |
| 2008 | 2015 | KX357903 | Myanmar | I | 1H | 1H5 |
| 2009 | 2015 | KX357904 | Myanmar | I | 1H | 1H5 |
| 2010 | 2015 | KX357905 | Myanmar | I | 1H | 1H5 |
| 2011 | 2015 | KX357906 | Myanmar | I | 1H | 1H5 |
| 2012 | 2015 | KX357907 | Myanmar | I | 1H | 1H5 |
| 2013 | 2015 | KX357908 | Myanmar | I | 1H | 1H5 |
| 2014 | 2015 | KX357909 | Myanmar | I | 1H | 1H5 |
| 2015 | 2015 | KX357910 | Myanmar | I | 1H | 1H5 |
| 2016 | 2015 | KX357911 | Myanmar | I | 1H | 1H5 |
| 2017 | 2015 | KX357912 | Myanmar | I | 1H | 1H5 |
| 2018 | 2015 | KX357913 | Myanmar | I | 1H | 1H5 |
| 2019 | 2015 | KX357914 | Myanmar | I | 1H | 1H5 |
| 2020 | 2015 | KX357915 | Myanmar | I | 1H | 1H5 |
| 2021 | 2015 | KX357916 | Myanmar | I | 1H | 1H5 |
| 2022 | 2015 | KX357918 | Myanmar | I | 1H | 1H5 |
| 2023 | 2015 | KX357919 | Myanmar | I | 1H | 1H5 |
| 2024 | 2015 | KX357920 | Myanmar | I | 1H | 1H5 |
| 2025 | 2015 | KX357921 | Myanmar | I | 1H | 1H5 |
| 2026 | 2015 | KX357922 | Myanmar | I | 1H | 1H5 |
| 2027 | 2015 | KX357923 | Myanmar | I | 1H | 1H5 |
| 2028 | 2015 | KX357924 | Myanmar | I | 1H | 1H5 |
| 2029 | 2015 | KX357925 | Myanmar | I | 1H | 1H5 |
| 2030 | 2015 | KX357926 | Myanmar | I | 1H | 1H5 |
| 2031 | 2015 | KX357928 | Myanmar | I | 1H | 1H5 |
| 2032 | 2015 | KX357929 | Myanmar | I | 1H | 1H5 |
| 2033 | 2015 | KX357931 | Myanmar | I | 1H | 1H5 |
| 2034 | 2015 | KX357932 | Myanmar | I | 1H | 1H5 |
| 2035 | 2015 | KX357934 | Myanmar | I | 1H | 1H5 |
| 2036 | 2015 | KX357936 | Myanmar | I | 1H | 1H5 |
| 2037 | 2015 | KX357937 | Myanmar | I | 1H | 1H5 |
| 2038 | 2015 | KX357939 | Myanmar | I | 1H | 1H5 |
| 2039 | 2015 | KX357941 | Myanmar | I | 1H | 1H5 |
| 2040 | 2015 | KX357944 | Myanmar | I | 1H | 1H5 |
| 2041 | 2015 | KX357945 | Myanmar | I | 1H | 1H5 |
| 2042 | 2015 | KX357947 | Myanmar | I | 1H | 1H5 |
| 2043 | 2015 | KX357948 | Myanmar | I | 1H | 1H5 |
| 2044 | 2015 | KX357950 | Myanmar | I | 1H | 1H5 |
| 2045 | 2015 | KX357951 | Myanmar | I | 1H | 1H5 |
| 2046 | 2015 | KX357955 | Myanmar | I | 1H | 1H5 |
| 2047 | 2015 | KX357956 | Myanmar | I | 1H | 1H5 |
| 2048 | 2015 | KX357957 | Myanmar | I | 1H | 1H5 |
| 2049 | 2015 | KX357960 | Myanmar | I | 1H | 1H5 |
| 2050 | 2015 | KX357961 | Myanmar | I | 1H | 1H5 |
| 2051 | 2015 | KX357964 | Myanmar | I | 1H | 1H5 |
| 2052 | 2015 | KX357965 | Myanmar | I | 1H | 1H5 |
| 2053 | 2015 | KX357966 | Myanmar | I | 1H | 1H5 |
| 2054 | 2015 | KX357968 | Myanmar | I | 1H | 1H5 |
| 2055 | 2015 | KX357969 | Myanmar | I | 1H | 1H5 |
| 2056 | 2015 | KX357971 | Myanmar | I | 1H | 1H5 |
| 2057 | 2015 | KX357972 | Myanmar | I | 1H | 1H5 |
| 2058 | 2015 | KX357973 | Myanmar | I | 1H | 1H5 |
| 2059 | 2015 | KX357974 | Myanmar | I | 1H | 1H5 |
| 2060 | 2015 | KX357975 | Myanmar | I | 1H | 1H5 |
| 2061 | 2015 | KY038890 | China | I | 1H | 1H5 |
| 2062 | 2015 | MF405201 | China | I | 1H | 1H5 |
| 2063 | 2000 | FJ639669 | Cambodia | I | 1I | 1I1 |
| 2064 | 2001 | FJ639670 | Cambodia | I | 1I | 1I1 |
| 2065 | 2001 | FJ639672 | Cambodia | I | 1I | 1I1 |
| 2066 | 2003 | FJ639677 | Cambodia | I | 1I | 1I1 |
| 2067 | 2003 | FJ639680 | Cambodia | I | 1I | 1I1 |
| 2068 | 2003 | FJ639681 | Cambodia | I | 1I | 1I1 |
| 2069 | 2003 | FJ882562 | Vietnam | I | 1I | 1I1 |
| 2070 | 2003 | FJ882563 | Vietnam | I | 1I | 1I1 |
| 2071 | 2003 | GQ868619 | Cambodia | I | 1I | 1I1 |
| 2072 | 2003 | KF955445 | Cambodia | I | 1I | 1I1 |
| 2073 | 2004 | FJ639682 | Cambodia | I | 1I | 1I1 |
| 2074 | 2004 | KC861958 | Vietnam | I | 1I | 1I1 |
| 2075 | 2005 | FJ898395 | Vietnam | I | 1I | 1I1 |
| 2076 | 2006 | EU249491 | Vietnam | I | 1I | 1I1 |
| 2077 | 2006 | EU482525 | Vietnam | I | 1I | 1I1 |
| 2078 | 2006 | EU482540 | Vietnam | I | 1I | 1I1 |
| 2079 | 2006 | EU482707 | Vietnam | I | 1I | 1I1 |
| 2080 | 2006 | EU660390 | Vietnam | I | 1I | 1I1 |
| 2081 | 2006 | FJ373296 | Vietnam | I | 1I | 1I1 |
| 2082 | 2006 | FJ882516 | Vietnam | I | 1I | 1I1 |
| 2083 | 2006 | FJ882528 | Vietnam | I | 1I | 1I1 |
| 2084 | 2006 | FJ882534 | Vietnam | I | 1I | 1I1 |
| 2085 | 2006 | FJ882536 | Vietnam | I | 1I | 1I1 |
| 2086 | 2006 | FJ898420 | Vietnam | I | 1I | 1I1 |
| 2087 | 2006 | FJ898421 | Vietnam | I | 1I | 1I1 |
| 2088 | 2006 | GQ199772 | Vietnam | I | 1I | 1I1 |
| 2089 | 2006 | GQ868612 | Vietnam | I | 1I | 1I1 |
| 2090 | 2006 | HQ588120 | Vietnam | I | 1I | 1I1 |
| 2091 | 2006 | HQ588121 | Vietnam | I | 1I | 1I1 |
| 2092 | 2007 | EU660412 | Vietnam | I | 1I | 1I1 |
| 2093 | 2007 | EU677159 | Vietnam | I | 1I | 1I1 |
| 2094 | 2007 | FJ024431 | Vietnam | I | 1I | 1I1 |
| 2095 | 2007 | FJ024434 | Vietnam | I | 1I | 1I1 |
| 2096 | 2007 | FJ410201 | Vietnam | I | 1I | 1I1 |
| 2097 | 2007 | FJ410203 | Vietnam | I | 1I | 1I1 |
| 2098 | 2007 | FJ410204 | Vietnam | I | 1I | 1I1 |
| 2099 | 2007 | FJ410209 | Vietnam | I | 1I | 1I1 |
| 2100 | 2007 | FJ410211 | Vietnam | I | 1I | 1I1 |
| 2101 | 2007 | FJ410232 | Vietnam | I | 1I | 1I1 |
| 2102 | 2007 | FJ410234 | Vietnam | I | 1I | 1I1 |
| 2103 | 2007 | FJ410250 | Vietnam | I | 1I | 1I1 |
| 2104 | 2007 | FJ432734 | Vietnam | I | 1I | 1I1 |
| 2105 | 2007 | FJ432736 | Vietnam | I | 1I | 1I1 |
| 2106 | 2007 | FJ432747 | Vietnam | I | 1I | 1I1 |
| 2107 | 2007 | FJ461313 | Vietnam | I | 1I | 1I1 |
| 2108 | 2007 | FJ461317 | Vietnam | I | 1I | 1I1 |
| 2109 | 2007 | FJ461323 | Vietnam | I | 1I | 1I1 |
| 2110 | 2007 | FJ461324 | Vietnam | I | 1I | 1I1 |
| 2111 | 2007 | FJ882545 | Vietnam | I | 1I | 1I1 |
| 2112 | 2007 | FJ882546 | Vietnam | I | 1I | 1I1 |
| 2113 | 2007 | FJ882547 | Vietnam | I | 1I | 1I1 |
| 2114 | 2007 | FJ882548 | Vietnam | I | 1I | 1I1 |
| 2115 | 2007 | FJ882557 | Vietnam | I | 1I | 1I1 |
| 2116 | 2007 | FJ898372 | Vietnam | I | 1I | 1I1 |
| 2117 | 2007 | FJ906728 | Vietnam | I | 1I | 1I1 |
| 2118 | 2007 | GQ199792 | Vietnam | I | 1I | 1I1 |
| 2119 | 2007 | GQ199803 | Vietnam | I | 1I | 1I1 |
| 2120 | 2007 | GQ199818 | Vietnam | I | 1I | 1I1 |
| 2121 | 2007 | GQ199828 | Vietnam | I | 1I | 1I1 |
| 2122 | 2007 | HQ588122 | Vietnam | I | 1I | 1I1 |
| 2123 | 2007 | JF937597 | Vietnam | I | 1I | 1I1 |
| 2124 | 2008 | FJ410257 | Vietnam | I | 1I | 1I1 |
| 2125 | 2008 | FJ410267 | Vietnam | I | 1I | 1I1 |
| 2126 | 2008 | FJ410274 | Vietnam | I | 1I | 1I1 |
| 2127 | 2008 | FJ410280 | Vietnam | I | 1I | 1I1 |
| 2128 | 2008 | FJ461331 | Vietnam | I | 1I | 1I1 |
| 2129 | 2008 | FJ461332 | Vietnam | I | 1I | 1I1 |
| 2130 | 2008 | GU131697 | Vietnam | I | 1I | 1I1 |
| 2131 | 2008 | GU131714 | Vietnam | I | 1I | 1I1 |
| 2132 | 2008 | GU131725 | Vietnam | I | 1I | 1I1 |
| 2133 | 2008 | GU131752 | Vietnam | I | 1I | 1I1 |
| 2134 | 2008 | GU131816 | Vietnam | I | 1I | 1I1 |
| 2135 | 2008 | GU131817 | Vietnam | I | 1I | 1I1 |
| 2136 | 2008 | HM181970 | Vietnam | I | 1I | 1I1 |
| 2137 | 2008 | JF937650 | Vietnam | I | 1I | 1I1 |
| 2138 | 2008 | JF967802 | Vietnam | I | 1I | 1I1 |
| 2139 | 2008 | JF967828 | Vietnam | I | 1I | 1I1 |
| 2140 | 2008 | JQ287667 | Vietnam | I | 1I | 1I1 |
| 2141 | 2008 | KF921952 | Vietnam | I | 1I | 1I1 |
| 2142 | 2009 | JF269179 | Vietnam | I | 1I | 1I1 |
| 2143 | 2009 | JF269180 | Vietnam | I | 1I | 1I1 |
| 2144 | 2009 | JF269184 | Vietnam | I | 1I | 1I1 |
| 2145 | 2009 | JF967859 | Vietnam | I | 1I | 1I1 |
| 2146 | 2009 | JX569840 | Vietnam | I | 1I | 1I1 |
| 2147 | 2009 | JX569842 | Vietnam | I | 1I | 1I1 |
| 2148 | 2009 | KC861928 | Vietnam | I | 1I | 1I1 |
| 2149 | 2004 | EU069618 | Singapore | I | 1J | 1J1 |
| 2150 | 2005 | JN697058 | Malaysia | I | 1J | 1J1 |
| 2151 | 2007 | JQ993110 | Thailand | I | 1J | 1J1 |
| 2152 | 2007 | JQ993111 | Thailand | I | 1J | 1J1 |
| 2153 | 2007 | JQ993134 | Thailand | I | 1J | 1J1 |
| 2154 | 2007 | LC148029 | Indonesia | I | 1J | 1J1 |
| 2155 | 2007 | EU448397 | Vietnam | I | 1J | 1J1 |
| 2156 | 2007 | JN415509 | Laos | I | 1J | 1J1 |
| 2157 | 2007 | KC182096 | Laos | I | 1J | 1J1 |
| 2158 | 2007 | KC182098 | Laos | I | 1J | 1J1 |
| 2159 | 2007 | KC182099 | Laos | I | 1J | 1J1 |
| 2160 | 2007 | KC182100 | Laos | I | 1J | 1J1 |
| 2161 | 2007 | KC182102 | Laos | I | 1J | 1J1 |
| 2162 | 2007 | JN638334 | Thailand | I | 1J | 1J1 |
| 2163 | 2007 | KX951690 | China | I | 1J | 1J1 |
| 2164 | 2008 | JF967811 | Thailand | I | 1J | 1J1 |
| 2165 | 2008 | JF967819 | Thailand | I | 1J | 1J1 |
| 2166 | 2008 | KC182105 | Laos | I | 1J | 1J1 |
| 2167 | 2008 | KC182106 | Laos | I | 1J | 1J1 |
| 2168 | 2008 | KC182107 | Laos | I | 1J | 1J1 |
| 2169 | 2008 | JF967838 | Thailand | I | 1J | 1J1 |
| 2170 | 2009 | JF967875 | Indonesia | I | 1J | 1J1 |
| 2171 | 2009 | KT373896 | Thailand | I | 1J | 1J1 |
| 2172 | 2009 | KT373901 | Thailand | I | 1J | 1J1 |
| 2173 | 2010 | JN415491 | Indonesia | I | 1J | 1J1 |
| 2174 | 2010 | AB624553 | Indonesia | I | 1J | 1J1 |
| 2175 | 2010 | AB624554 | Indonesia | I | 1J | 1J1 |
| 2176 | 2010 | JF967923 | Thailand | I | 1J | 1J1 |
| 2177 | 2010 | JF967945 | Thailand | I | 1J | 1J1 |
| 2178 | 2007 | HM469966 | Thailand | I | 1J | 1J2 |
| 2179 | 2007 | JQ993112 | Thailand | I | 1J | 1J2 |
| 2180 | 2007 | JQ993135 | Thailand | I | 1J | 1J2 |
| 2181 | 2007 | JQ993139 | Thailand | I | 1J | 1J2 |
| 2182 | 2007 | JQ993141 | Thailand | I | 1J | 1J2 |
| 2183 | 2007 | JQ993143 | Thailand | I | 1J | 1J2 |
| 2184 | 2007 | JQ993199 | Thailand | I | 1J | 1J2 |
| 2185 | 2007 | JQ993200 | Thailand | I | 1J | 1J2 |
| 2186 | 2007 | JQ993201 | Thailand | I | 1J | 1J2 |
| 2187 | 2008 | KC172831 | Laos | I | 1J | 1J2 |
| 2188 | 2008 | KY849749 | Laos | I | 1J | 1J2 |
| 2189 | 2008 | JF967801 | Thailand | I | 1J | 1J2 |
| 2190 | 2009 | KC182086 | Laos | I | 1J | 1J2 |
| 2191 | 2009 | KT373899 | Thailand | I | 1J | 1J2 |
| 2192 | 2009 | KY849727 | Laos | I | 1J | 1J2 |
| 2193 | 2009 | KY849750 | Laos | I | 1J | 1J2 |
| 2194 | 2009 | JN638326 | Thailand | I | 1J | 1J2 |
| 2195 | 2010 | HG316482 | Thailand | I | 1J | 1J2 |
| 2196 | 2010 | JN415528 | Thailand | I | 1J | 1J2 |
| 2197 | 2010 | JF967919 | Cambodia | I | 1J | 1J2 |
| 2198 | 2012 | JQ896297 | NA | I | 1J | 1J2 |
| 2199 | 2013 | KJ438293 | China | I | 1J | 1J2 |
| 2200 | 2007 | HM469968 | Thailand | I | 1J | 1J3 |
| 2201 | 2008 | JF967803 | Thailand | I | 1J | 1J3 |
| 2202 | 2009 | JF967880 | Thailand | I | 1J | 1J3 |
| 2203 | 2009 | JF967888 | Thailand | I | 1J | 1J3 |
| 2204 | 2009 | JQ403519 | China | I | 1J | 1J3 |
| 2205 | 2009 | KT373897 | Thailand | I | 1J | 1J3 |
| 2206 | 2009 | KT824965 | NA | I | 1J | 1J3 |
| 2207 | 2011 | KT824981 | Thailand | I | 1J | 1J3 |
| 2208 | 2012 | KT824996 | NA | I | 1J | 1J3 |
| 2209 | 2012 | KJ806934 | Singapore | I | 1J | 1J3 |
| 2210 | 2012 | KT824993 | Thailand | I | 1J | 1J3 |
| 2211 | 2012 | KU509263 | Thailand | I | 1J | 1J3 |
| 2212 | 2012 | KU509266 | Thailand | I | 1J | 1J3 |
| 2213 | 2012 | KY818155 | Thailand | I | 1J | 1J3 |
| 2214 | 2012 | KY818156 | Thailand | I | 1J | 1J3 |
| 2215 | 2012 | KY818157 | Thailand | I | 1J | 1J3 |
| 2216 | 2012 | KY818158 | Thailand | I | 1J | 1J3 |
| 2217 | 2013 | KT825013 | Thailand | I | 1J | 1J3 |
| 2218 | 2013 | KU509314 | Thailand | I | 1J | 1J3 |
| 2219 | 2013 | KJ806943 | Singapore | I | 1J | 1J3 |
| 2220 | 2013 | KJ806944 | Singapore | I | 1J | 1J3 |
| 2221 | 2014 | LC011949 | Japan | I | 1J | 1J3 |
| 2222 | 2014 | LC012535 | Japan | I | 1J | 1J3 |
| 2223 | 2005 | EU081276 | Singapore | I | 1J | 1J4 |
| 2224 | 2006 | EU448400 | Malaysia | I | 1J | 1J4 |
| 2225 | 2006 | KC861918 | Vietnam | I | 1J | 1J4 |
| 2226 | 2006 | KC861965 | Vietnam | I | 1J | 1J4 |
| 2227 | 2007 | EU359008 | China | I | 1J | 1J4 |
| 2228 | 2007 | FJ158609 | China | I | 1J | 1J4 |
| 2229 | 2007 | FJ158610 | China | I | 1J | 1J4 |
| 2230 | 2007 | FJ158611 | China | I | 1J | 1J4 |
| 2231 | 2007 | FJ158612 | China | I | 1J | 1J4 |
| 2232 | 2007 | JQ317738 | China | I | 1J | 1J4 |
| 2233 | 2007 | JQ317739 | China | I | 1J | 1J4 |
| 2234 | 2007 | JQ317740 | China | I | 1J | 1J4 |
| 2235 | 2007 | JQ317741 | China | I | 1J | 1J4 |
| 2236 | 2007 | JQ317742 | China | I | 1J | 1J4 |
| 2237 | 2007 | KT827368 | China | I | 1J | 1J4 |
| 2238 | 2008 | GQ357683 | Singapore | I | 1J | 1J4 |
| 2239 | 2008 | GQ357684 | Singapore | I | 1J | 1J4 |
| 2240 | 2008 | GQ357685 | Singapore | I | 1J | 1J4 |
| 2241 | 2008 | JF967798 | Indonesia | I | 1J | 1J4 |
| 2242 | 2008 | JF967807 | Indonesia | I | 1J | 1J4 |
| 2243 | 2009 | JF960214 | Singapore | I | 1J | 1J4 |
| 2244 | 2009 | JF967855 | Indonesia | I | 1J | 1J4 |
| 2245 | 2009 | JF967868 | Indonesia | I | 1J | 1J4 |
| 2246 | 2009 | KX646376 | Indonesia | I | 1J | 1J4 |
| 2247 | 2009 | KX646377 | Indonesia | I | 1J | 1J4 |
| 2248 | 2009 | KX646380 | Indonesia | I | 1J | 1J4 |
| 2249 | 2009 | KX646381 | Indonesia | I | 1J | 1J4 |
| 2250 | 2010 | JF960222 | Singapore | I | 1J | 1J4 |
| 2251 | 2010 | JF967896 | Indonesia | I | 1J | 1J4 |
| 2252 | 2010 | JF967899 | Indonesia | I | 1J | 1J4 |
| 2253 | 2010 | JF967906 | Indonesia | I | 1J | 1J4 |
| 2254 | 2010 | JF967917 | Singapore | I | 1J | 1J4 |
| 2255 | 2010 | JN380806 | Singapore | I | 1J | 1J4 |
| 2256 | 2010 | JN380807 | Singapore | I | 1J | 1J4 |
| 2257 | 2012 | KF052647 | Indonesia | I | 1J | 1J4 |
| 2258 | 2013 | KJ806947 | Singapore | I | 1J | 1J4 |
| 2259 | 2014 | KU529696 | Indonesia | I | 1J | 1J4 |
| 2260 | 2008 | JQ317736 | China | I | 1J | 1J5 |
| 2261 | 2008 | JQ317737 | China | I | 1J | 1J5 |
| 2262 | 2008 | KC172833 | Laos | I | 1J | 1J5 |
| 2263 | 2008 | KY849705 | Laos | I | 1J | 1J5 |
| 2264 | 2008 | KY849740 | Laos | I | 1J | 1J5 |
| 2265 | 2009 | JF967878 | Thailand | I | 1J | 1J5 |
| 2266 | 2009 | JN638335 | Thailand | I | 1J | 1J5 |
| 2267 | 2009 | KC182087 | Laos | I | 1J | 1J5 |
| 2268 | 2009 | KC182088 | Laos | I | 1J | 1J5 |
| 2269 | 2009 | KC182089 | Laos | I | 1J | 1J5 |
| 2270 | 2009 | KC182090 | Laos | I | 1J | 1J5 |
| 2271 | 2009 | KY849701 | Laos | I | 1J | 1J5 |
| 2272 | 2009 | KY849707 | Laos | I | 1J | 1J5 |
| 2273 | 2009 | KY849708 | Laos | I | 1J | 1J5 |
| 2274 | 2009 | KY849709 | Laos | I | 1J | 1J5 |
| 2275 | 2009 | KY849710 | Laos | I | 1J | 1J5 |
| 2276 | 2009 | KY849711 | Laos | I | 1J | 1J5 |
| 2277 | 2009 | KY849712 | Laos | I | 1J | 1J5 |
| 2278 | 2009 | KY849713 | Laos | I | 1J | 1J5 |
| 2279 | 2009 | KY849714 | Laos | I | 1J | 1J5 |
| 2280 | 2009 | KY849715 | Laos | I | 1J | 1J5 |
| 2281 | 2009 | KY849716 | Laos | I | 1J | 1J5 |
| 2282 | 2009 | KY849717 | Laos | I | 1J | 1J5 |
| 2283 | 2009 | KY849718 | Laos | I | 1J | 1J5 |
| 2284 | 2009 | KY849719 | Laos | I | 1J | 1J5 |
| 2285 | 2009 | KY849742 | Laos | I | 1J | 1J5 |
| 2286 | 2009 | KY849748 | Laos | I | 1J | 1J5 |
| 2287 | 2010 | KC182091 | Laos | I | 1J | 1J5 |
| 2288 | 2010 | KC182092 | Laos | I | 1J | 1J5 |
| 2289 | 2010 | KY849721 | Laos | I | 1J | 1J5 |
| 2290 | 2010 | KY849722 | Laos | I | 1J | 1J5 |
| 2291 | 2010 | KY849723 | Laos | I | 1J | 1J5 |
| 2292 | 2010 | KY849744 | Laos | I | 1J | 1J5 |
| 2293 | 2010 | KY849751 | Laos | I | 1J | 1J5 |
| 2294 | 2011 | KY849704 | Laos | I | 1J | 1J5 |
| 2295 | 2012 | KY818146 | Thailand | I | 1J | 1J5 |
| 2296 | 2013 | KY818147 | Thailand | I | 1J | 1J5 |
| 2297 | 2013 | KY818148 | Thailand | I | 1J | 1J5 |
| 2298 | 2013 | KY818149 | Thailand | I | 1J | 1J5 |
| 2299 | 2013 | KY818150 | Thailand | I | 1J | 1J5 |
| 2300 | 2013 | KY818151 | Thailand | I | 1J | 1J5 |
| 2301 | 2013 | KY818152 | Thailand | I | 1J | 1J5 |
| 2302 | 2013 | KY882504 | Thailand | I | 1J | 1J5 |
| 2303 | 2002 | AB111076 | Thailand | I | 1J | 1J6 |
| 2304 | 2002 | AB111077 | Thailand | I | 1J | 1J6 |
| 2305 | 2002 | AB111079 | Thailand | I | 1J | 1J6 |
| 2306 | 2002 | AB178040 | Micronesia | I | 1J | 1J6 |
| 2307 | 2002 | EU069600 | Singapore | I | 1J | 1J6 |
| 2308 | 2003 | EU069598 | Singapore | I | 1J | 1J6 |
| 2309 | 2003 | EU069601 | Singapore | I | 1J | 1J6 |
| 2310 | 2003 | EU069607 | Singapore | I | 1J | 1J6 |
| 2311 | 2003 | EU069608 | Singapore | I | 1J | 1J6 |
| 2312 | 2003 | EU069610 | Singapore | I | 1J | 1J6 |
| 2313 | 2003 | EU069612 | Singapore | I | 1J | 1J6 |
| 2314 | 2003 | EU069614 | Singapore | I | 1J | 1J6 |
| 2315 | 2003 | EU069615 | Singapore | I | 1J | 1J6 |
| 2316 | 2003 | EU069616 | Singapore | I | 1J | 1J6 |
| 2317 | 2003 | EU069617 | Singapore | I | 1J | 1J6 |
| 2318 | 2003 | EU448396 | Thailand | I | 1J | 1J6 |
| 2319 | 2003 | FJ469907 | Singapore | I | 1J | 1J6 |
| 2320 | 2003 | FJ469908 | Singapore | I | 1J | 1J6 |
| 2321 | 2003 | FJ469909 | Singapore | I | 1J | 1J6 |
| 2322 | 2004 | DQ193572 | China | I | 1J | 1J6 |
| 2323 | 2004 | EF508202 | China | I | 1J | 1J6 |
| 2324 | 2004 | EU069593 | Singapore | I | 1J | 1J6 |
| 2325 | 2004 | EU069603 | Singapore | I | 1J | 1J6 |
| 2326 | 2004 | EU069605 | Singapore | I | 1J | 1J6 |
| 2327 | 2004 | EU069606 | Singapore | I | 1J | 1J6 |
| 2328 | 2004 | EU069609 | Singapore | I | 1J | 1J6 |
| 2329 | 2004 | EU069613 | Singapore | I | 1J | 1J6 |
| 2330 | 2004 | EU069619 | Singapore | I | 1J | 1J6 |
| 2331 | 2004 | EU069620 | Singapore | I | 1J | 1J6 |
| 2332 | 2004 | EU069621 | Singapore | I | 1J | 1J6 |
| 2333 | 2004 | EU069622 | Singapore | I | 1J | 1J6 |
| 2334 | 2004 | EU069623 | Singapore | I | 1J | 1J6 |
| 2335 | 2004 | EU069624 | Singapore | I | 1J | 1J6 |
| 2336 | 2004 | FR666922 | Malaysia | I | 1J | 1J6 |
| 2337 | 2004 | FR666923 | Malaysia | I | 1J | 1J6 |
| 2338 | 2004 | FR666926 | Malaysia | I | 1J | 1J6 |
| 2339 | 2004 | GQ328923 | Malaysia | I | 1J | 1J6 |
| 2340 | 2004 | JQ317718 | China | I | 1J | 1J6 |
| 2341 | 2004 | KU570091 | China | I | 1J | 1J6 |
| 2342 | 2004 | KU570092 | China | I | 1J | 1J6 |
| 2343 | 2004 | KU570093 | China | I | 1J | 1J6 |
| 2344 | 2004 | LC148022 | Malaysia | I | 1J | 1J6 |
| 2345 | 2005 | EU069594 | Singapore | I | 1J | 1J6 |
| 2346 | 2005 | EU069595 | Singapore | I | 1J | 1J6 |
| 2347 | 2005 | EU069596 | Singapore | I | 1J | 1J6 |
| 2348 | 2005 | EU069602 | Singapore | I | 1J | 1J6 |
| 2349 | 2005 | EU081226 | Singapore | I | 1J | 1J6 |
| 2350 | 2005 | EU081227 | Singapore | I | 1J | 1J6 |
| 2351 | 2005 | EU081228 | Singapore | I | 1J | 1J6 |
| 2352 | 2005 | EU081229 | Singapore | I | 1J | 1J6 |
| 2353 | 2005 | EU081230 | Singapore | I | 1J | 1J6 |
| 2354 | 2005 | EU081231 | Singapore | I | 1J | 1J6 |
| 2355 | 2005 | EU081232 | Singapore | I | 1J | 1J6 |
| 2356 | 2005 | EU081233 | Singapore | I | 1J | 1J6 |
| 2357 | 2005 | EU081234 | Singapore | I | 1J | 1J6 |
| 2358 | 2005 | EU081235 | Singapore | I | 1J | 1J6 |
| 2359 | 2005 | EU081236 | Singapore | I | 1J | 1J6 |
| 2360 | 2005 | EU081237 | Singapore | I | 1J | 1J6 |
| 2361 | 2005 | EU081238 | Singapore | I | 1J | 1J6 |
| 2362 | 2005 | EU081239 | Singapore | I | 1J | 1J6 |
| 2363 | 2005 | EU081240 | Singapore | I | 1J | 1J6 |
| 2364 | 2005 | EU081241 | Singapore | I | 1J | 1J6 |
| 2365 | 2005 | EU081242 | Singapore | I | 1J | 1J6 |
| 2366 | 2005 | EU081243 | Singapore | I | 1J | 1J6 |
| 2367 | 2005 | EU081244 | Singapore | I | 1J | 1J6 |
| 2368 | 2005 | EU081245 | Singapore | I | 1J | 1J6 |
| 2369 | 2005 | EU081246 | Singapore | I | 1J | 1J6 |
| 2370 | 2005 | EU081247 | Singapore | I | 1J | 1J6 |
| 2371 | 2005 | EU081248 | Singapore | I | 1J | 1J6 |
| 2372 | 2005 | EU081249 | Singapore | I | 1J | 1J6 |
| 2373 | 2005 | EU081250 | Singapore | I | 1J | 1J6 |
| 2374 | 2005 | EU081251 | Singapore | I | 1J | 1J6 |
| 2375 | 2005 | EU081252 | Singapore | I | 1J | 1J6 |
| 2376 | 2005 | EU081253 | Singapore | I | 1J | 1J6 |
| 2377 | 2005 | EU081254 | Singapore | I | 1J | 1J6 |
| 2378 | 2005 | EU081255 | Singapore | I | 1J | 1J6 |
| 2379 | 2005 | EU081256 | Singapore | I | 1J | 1J6 |
| 2380 | 2005 | EU081257 | Singapore | I | 1J | 1J6 |
| 2381 | 2005 | EU081259 | Singapore | I | 1J | 1J6 |
| 2382 | 2005 | EU081260 | Singapore | I | 1J | 1J6 |
| 2383 | 2005 | EU081261 | Singapore | I | 1J | 1J6 |
| 2384 | 2005 | EU081262 | Singapore | I | 1J | 1J6 |
| 2385 | 2005 | EU081263 | Singapore | I | 1J | 1J6 |
| 2386 | 2005 | EU081264 | Singapore | I | 1J | 1J6 |
| 2387 | 2005 | EU081265 | Singapore | I | 1J | 1J6 |
| 2388 | 2005 | EU081266 | Singapore | I | 1J | 1J6 |
| 2389 | 2005 | EU081267 | Singapore | I | 1J | 1J6 |
| 2390 | 2005 | EU081268 | Singapore | I | 1J | 1J6 |
| 2391 | 2005 | EU081269 | Singapore | I | 1J | 1J6 |
| 2392 | 2005 | EU081270 | Singapore | I | 1J | 1J6 |
| 2393 | 2005 | EU081271 | Singapore | I | 1J | 1J6 |
| 2394 | 2005 | EU081272 | Singapore | I | 1J | 1J6 |
| 2395 | 2005 | EU081273 | Singapore | I | 1J | 1J6 |
| 2396 | 2005 | EU081274 | Singapore | I | 1J | 1J6 |
| 2397 | 2005 | EU081275 | Singapore | I | 1J | 1J6 |
| 2398 | 2005 | EU081277 | Singapore | I | 1J | 1J6 |
| 2399 | 2005 | EU081278 | Singapore | I | 1J | 1J6 |
| 2400 | 2005 | EU081279 | Singapore | I | 1J | 1J6 |
| 2401 | 2005 | EU448399 | Singapore | I | 1J | 1J6 |
| 2402 | 2005 | FR666924 | Malaysia | I | 1J | 1J6 |
| 2403 | 2005 | FR666925 | Malaysia | I | 1J | 1J6 |
| 2404 | 2005 | FR666927 | Malaysia | I | 1J | 1J6 |
| 2405 | 2005 | FR666928 | Malaysia | I | 1J | 1J6 |
| 2406 | 2005 | JN415511 | Malaysia | I | 1J | 1J6 |
| 2407 | 2005 | JN415529 | NA | I | 1J | 1J6 |
| 2408 | 2005 | JN697057 | Malaysia | I | 1J | 1J6 |
| 2409 | 2005 | KJ806933 | Singapore | I | 1J | 1J6 |
| 2410 | 2006 | EU081280 | Singapore | I | 1J | 1J6 |
| 2411 | 2006 | EU081281 | Singapore | I | 1J | 1J6 |
| 2412 | 2006 | JQ993167 | Thailand | I | 1J | 1J6 |
| 2413 | 2006 | JQ993173 | Thailand | I | 1J | 1J6 |
| 2414 | 2006 | JQ993174 | Thailand | I | 1J | 1J6 |
| 2415 | 2006 | JQ993178 | Thailand | I | 1J | 1J6 |
| 2416 | 2006 | JQ993196 | Thailand | I | 1J | 1J6 |
| 2417 | 2007 | EU448398 | Thailand | I | 1J | 1J6 |
| 2418 | 2008 | FJ687478 | Malaysia | I | 1J | 1J6 |
| 2419 | 2008 | GQ357681 | Singapore | I | 1J | 1J6 |
| 2420 | 2008 | GQ357682 | Singapore | I | 1J | 1J6 |
| 2421 | 2008 | GQ357686 | Singapore | I | 1J | 1J6 |
| 2422 | 2008 | GU370049 | Singapore | I | 1J | 1J6 |
| 2423 | 2008 | HQ591537 | Vietnam | I | 1J | 1J6 |
| 2424 | 2008 | HQ591544 | Vietnam | I | 1J | 1J6 |
| 2425 | 2008 | HQ591545 | Vietnam | I | 1J | 1J6 |
| 2426 | 2008 | HQ591547 | Vietnam | I | 1J | 1J6 |
| 2427 | 2008 | HQ591548 | Vietnam | I | 1J | 1J6 |
| 2428 | 2008 | HQ591556 | Vietnam | I | 1J | 1J6 |
| 2429 | 2008 | JF967826 | Thailand | I | 1J | 1J6 |
| 2430 | 2008 | JF967831 | Vietnam | I | 1J | 1J6 |
| 2431 | 2008 | JF967846 | Vietnam | I | 1J | 1J6 |
| 2432 | 2008 | JN022601 | Singapore | I | 1J | 1J6 |
| 2433 | 2008 | KC861935 | Vietnam | I | 1J | 1J6 |
| 2434 | 2008 | KC861969 | Vietnam | I | 1J | 1J6 |
| 2435 | 2009 | JF960212 | Singapore | I | 1J | 1J6 |
| 2436 | 2010 | AB597966 | Indonesia | I | 1J | 1J6 |
| 2437 | 2010 | AB597967 | Indonesia | I | 1J | 1J6 |
| 2438 | 2010 | AB597968 | Indonesia | I | 1J | 1J6 |
| 2439 | 2010 | AB597969 | Indonesia | I | 1J | 1J6 |
| 2440 | 2010 | AB597970 | Indonesia | I | 1J | 1J6 |
| 2441 | 2010 | AB597971 | Indonesia | I | 1J | 1J6 |
| 2442 | 2010 | AB597972 | Indonesia | I | 1J | 1J6 |
| 2443 | 2010 | AB597973 | Indonesia | I | 1J | 1J6 |
| 2444 | 2010 | AB597974 | Indonesia | I | 1J | 1J6 |
| 2445 | 2010 | AB597975 | Indonesia | I | 1J | 1J6 |
| 2446 | 2010 | AB597976 | Indonesia | I | 1J | 1J6 |
| 2447 | 2010 | AB597977 | Indonesia | I | 1J | 1J6 |
| 2448 | 2010 | JF960224 | Singapore | I | 1J | 1J6 |
| 2449 | 2010 | JF967895 | Indonesia | I | 1J | 1J6 |
| 2450 | 2010 | KM216666 | Indonesia | I | 1J | 1J6 |
| 2451 | 2010 | KT204460 | Indonesia | I | 1J | 1J6 |
| 2452 | 2011 | KT825072 | Indonesia | I | 1J | 1J6 |
| 2453 | 2012 | KY216157 | Indonesia | I | 1J | 1J6 |
| 2454 | 2012 | KY216158 | Indonesia | I | 1J | 1J6 |
| 2455 | 2012 | KY216159 | Indonesia | I | 1J | 1J6 |
| 2456 | 2012 | AB915377 | Indonesia | I | 1J | 1J6 |
| 2457 | 2012 | KT204449 | Indonesia | I | 1J | 1J6 |
| 2458 | 2012 | KT204451 | Indonesia | I | 1J | 1J6 |
| 2459 | 2012 | KT204452 | Indonesia | I | 1J | 1J6 |
| 2460 | 2012 | KT204454 | Indonesia | I | 1J | 1J6 |
| 2461 | 2013 | AB915376 | Indonesia | I | 1J | 1J6 |
| 2462 | 2013 | KU509309 | Indonesia | I | 1J | 1J6 |
| 2463 | 2015 | KU529727 | Indonesia | I | 1J | 1J6 |
| 2464 | 2010 | JN415489 | Indonesia | I | 1J | 1J7 |
| 2465 | 2011 | KM216688 | Indonesia | I | 1J | 1J7 |
| 2466 | 2012 | KT824983 | Indonesia | I | 1J | 1J7 |
| 2467 | 2012 | KT824984 | Indonesia | I | 1J | 1J7 |
| 2468 | 2012 | KT824997 | Australia | I | 1J | 1J7 |
| 2469 | 2012 | KT825050 | Australia | I | 1J | 1J7 |
| 2470 | 2012 | KT825051 | Indonesia | I | 1J | 1J7 |
| 2471 | 2013 | KJ545459 | China | I | 1J | 1J7 |
| 2472 | 2013 | KJ806953 | Singapore | I | 1J | 1J7 |
| 2473 | 2013 | KT824998 | Indonesia | I | 1J | 1J7 |
| 2474 | 2013 | KT824999 | Indonesia | I | 1J | 1J7 |
| 2475 | 2013 | KT825006 | Australia | I | 1J | 1J7 |
| 2476 | 2013 | KT825015 | Australia | I | 1J | 1J7 |
| 2477 | 2013 | KT825053 | Indonesia | I | 1J | 1J7 |
| 2478 | 2013 | KY818132 | Indonesia | I | 1J | 1J7 |
| 2479 | 2013 | KY818133 | Indonesia | I | 1J | 1J7 |
| 2480 | 2013 | KY818136 | Indonesia | I | 1J | 1J7 |
| 2481 | 2013 | KY818137 | Indonesia | I | 1J | 1J7 |
| 2482 | 2014 | KJ806962 | Singapore | I | 1J | 1J7 |
| 2483 | 2014 | KJ806963 | Singapore | I | 1J | 1J7 |
| 2484 | 2014 | KP191493 | China | I | 1J | 1J7 |
| 2485 | 2014 | KP723476 | China | I | 1J | 1J7 |
| 2486 | 2014 | KT175083 | Malaysia | I | 1J | 1J7 |
| 2487 | 2014 | KT175085 | Malaysia | I | 1J | 1J7 |
| 2488 | 2014 | KT175088 | Malaysia | I | 1J | 1J7 |
| 2489 | 2014 | KT453229 | China | I | 1J | 1J7 |
| 2490 | 2014 | KT825020 | Indonesia | I | 1J | 1J7 |
| 2491 | 2014 | KT825031 | Thailand | I | 1J | 1J7 |
| 2492 | 2014 | KT825059 | Indonesia | I | 1J | 1J7 |
| 2493 | 2014 | KT831765 | Indonesia | I | 1J | 1J7 |
| 2494 | 2014 | KU666940 | Malaysia | I | 1J | 1J7 |
| 2495 | 2014 | KU666941 | Malaysia | I | 1J | 1J7 |
| 2496 | 2014 | KU666942 | Malaysia | I | 1J | 1J7 |
| 2497 | 2014 | KX224256 | Singapore | I | 1J | 1J7 |
| 2498 | 2014 | KX224257 | Singapore | I | 1J | 1J7 |
| 2499 | 2014 | KX452050 | Malaysia | I | 1J | 1J7 |
| 2500 | 2014 | KX452051 | Malaysia | I | 1J | 1J7 |
| 2501 | 2014 | KX452052 | Malaysia | I | 1J | 1J7 |
| 2502 | 2014 | KX452054 | Malaysia | I | 1J | 1J7 |
| 2503 | 2014 | KX452055 | Malaysia | I | 1J | 1J7 |
| 2504 | 2014 | KX452056 | Malaysia | I | 1J | 1J7 |
| 2505 | 2014 | KX452057 | Malaysia | I | 1J | 1J7 |
| 2506 | 2014 | KX452058 | Malaysia | I | 1J | 1J7 |
| 2507 | 2014 | KX452059 | Malaysia | I | 1J | 1J7 |
| 2508 | 2014 | KX452060 | Malaysia | I | 1J | 1J7 |
| 2509 | 2014 | KX452061 | Malaysia | I | 1J | 1J7 |
| 2510 | 2014 | KX452062 | Malaysia | I | 1J | 1J7 |
| 2511 | 2014 | KX452063 | Malaysia | I | 1J | 1J7 |
| 2512 | 2014 | KX452064 | Malaysia | I | 1J | 1J7 |
| 2513 | 2014 | KX452065 | Malaysia | I | 1J | 1J7 |
| 2514 | 2014 | KX452068 | Malaysia | I | 1J | 1J7 |
| 2515 | 2014 | LC002828 | Japan | I | 1J | 1J7 |
| 2516 | 2014 | LC006123 | Japan | I | 1J | 1J7 |
| 2517 | 2014 | LC011945 | Japan | I | 1J | 1J7 |
| 2518 | 2014 | LC011946 | Japan | I | 1J | 1J7 |
| 2519 | 2014 | LC011947 | Japan | I | 1J | 1J7 |
| 2520 | 2014 | LC011948 | Japan | I | 1J | 1J7 |
| 2521 | 2014 | LC012534 | Singapore | I | 1J | 1J7 |
| 2522 | 2014 | LC016760 | Japan | I | 1J | 1J7 |
| 2523 | 2014 | LC038150 | Indonesia | I | 1J | 1J7 |
| 2524 | 2015 | KT306907 | China | I | 1J | 1J7 |
| 2525 | 2015 | KT825071 | Australia | I | 1J | 1J7 |
| 2526 | 2015 | KU509289 | Thailand | I | 1J | 1J7 |
| 2527 | 2015 | KY006132 | Indonesia | I | 1J | 1J7 |
| 2528 | 2015 | KY006134 | Indonesia | I | 1J | 1J7 |
| 2529 | 2015 | KY006135 | Indonesia | I | 1J | 1J7 |
| 2530 | 2015 | KY006136 | Indonesia | I | 1J | 1J7 |
| 2531 | 2015 | KY006138 | Indonesia | I | 1J | 1J7 |
| 2532 | 2015 | KY709181 | Indonesia | I | 1J | 1J7 |
| 2533 | 2015 | KY921902 | Singapore | I | 1J | 1J7 |
| 2534 | 2006 | EF508206 | China | I | 1K | 1K1 |
| 2535 | 2006 | EF508207 | China | I | 1K | 1K1 |
| 2536 | 2008 | JF967818 | Thailand | I | 1K | 1K1 |
| 2537 | 2008 | JF967820 | Thailand | I | 1K | 1K1 |
| 2538 | 2008 | KU509257 | Thailand | I | 1K | 1K1 |
| 2539 | 2008 | LC148025 | Cambodia | I | 1K | 1K1 |
| 2540 | 2009 | HQ149731 | China | I | 1K | 1K1 |
| 2541 | 2009 | KT373898 | Thailand | I | 1K | 1K1 |
| 2542 | 2009 | KT373900 | Thailand | I | 1K | 1K1 |
| 2543 | 2012 | KY818154 | Thailand | I | 1K | 1K1 |
| 2544 | 2012 | KY818159 | Thailand | I | 1K | 1K1 |
| 2545 | 2012 | KY818160 | Thailand | I | 1K | 1K1 |
| 2546 | 2013 | KF971869 | China | I | 1K | 1K1 |
| 2547 | 2013 | KF971870 | China | I | 1K | 1K1 |
| 2548 | 2013 | KF971871 | China | I | 1K | 1K1 |
| 2549 | 2013 | KJ438294 | China | I | 1K | 1K1 |
| 2550 | 2013 | KJ438295 | China | I | 1K | 1K1 |
| 2551 | 2013 | KJ438296 | China | I | 1K | 1K1 |
| 2552 | 2013 | KJ438297 | China | I | 1K | 1K1 |
| 2553 | 2013 | KJ545437 | China | I | 1K | 1K1 |
| 2554 | 2013 | KJ545438 | China | I | 1K | 1K1 |
| 2555 | 2013 | KJ545439 | China | I | 1K | 1K1 |
| 2556 | 2013 | KJ545441 | China | I | 1K | 1K1 |
| 2557 | 2013 | KJ545445 | China | I | 1K | 1K1 |
| 2558 | 2013 | KJ545446 | China | I | 1K | 1K1 |
| 2559 | 2013 | KJ545449 | China | I | 1K | 1K1 |
| 2560 | 2013 | KJ545452 | China | I | 1K | 1K1 |
| 2561 | 2013 | KJ545456 | China | I | 1K | 1K1 |
| 2562 | 2013 | KJ545458 | China | I | 1K | 1K1 |
| 2563 | 2013 | KJ545460 | China | I | 1K | 1K1 |
| 2564 | 2013 | KJ545461 | China | I | 1K | 1K1 |
| 2565 | 2013 | KJ545462 | China | I | 1K | 1K1 |
| 2566 | 2013 | KJ545463 | China | I | 1K | 1K1 |
| 2567 | 2013 | KJ545464 | China | I | 1K | 1K1 |
| 2568 | 2013 | KJ545465 | China | I | 1K | 1K1 |
| 2569 | 2013 | KJ545475 | China | I | 1K | 1K1 |
| 2570 | 2013 | KJ545476 | China | I | 1K | 1K1 |
| 2571 | 2013 | KJ545477 | China | I | 1K | 1K1 |
| 2572 | 2013 | KJ806945 | Singapore | I | 1K | 1K1 |
| 2573 | 2013 | KT825002 | Australia | I | 1K | 1K1 |
| 2574 | 2013 | KT827370 | China | I | 1K | 1K1 |
| 2575 | 2013 | KX082948 | China | I | 1K | 1K1 |
| 2576 | 2013 | KX082949 | China | I | 1K | 1K1 |
| 2577 | 2013 | KX082950 | China | I | 1K | 1K1 |
| 2578 | 2013 | KX082951 | China | I | 1K | 1K1 |
| 2579 | 2013 | KX225488 | China | I | 1K | 1K1 |
| 2580 | 2013 | KX225489 | China | I | 1K | 1K1 |
| 2581 | 2013 | KX621251 | China | I | 1K | 1K1 |
| 2582 | 2014 | KP185303 | China | I | 1K | 1K1 |
| 2583 | 2014 | KP191501 | China | I | 1K | 1K1 |
| 2584 | 2014 | KP191511 | China | I | 1K | 1K1 |
| 2585 | 2014 | KP191512 | China | I | 1K | 1K1 |
| 2586 | 2014 | KP191516 | China | I | 1K | 1K1 |
| 2587 | 2014 | KP723474 | China | I | 1K | 1K1 |
| 2588 | 2014 | KT037103 | China | I | 1K | 1K1 |
| 2589 | 2014 | KT037105 | China | I | 1K | 1K1 |
| 2590 | 2014 | KT037106 | China | I | 1K | 1K1 |
| 2591 | 2014 | KT037109 | China | I | 1K | 1K1 |
| 2592 | 2014 | KT175101 | China | I | 1K | 1K1 |
| 2593 | 2014 | KT187564 | China | I | 1K | 1K1 |
| 2594 | 2014 | KT232178 | China | I | 1K | 1K1 |
| 2595 | 2014 | KT232179 | China | I | 1K | 1K1 |
| 2596 | 2014 | KT232180 | China | I | 1K | 1K1 |
| 2597 | 2014 | KT232181 | China | I | 1K | 1K1 |
| 2598 | 2014 | KT232182 | China | I | 1K | 1K1 |
| 2599 | 2014 | KT232184 | China | I | 1K | 1K1 |
| 2600 | 2014 | KT232185 | China | I | 1K | 1K1 |
| 2601 | 2014 | KT232188 | China | I | 1K | 1K1 |
| 2602 | 2014 | KT232189 | China | I | 1K | 1K1 |
| 2603 | 2014 | KT232190 | China | I | 1K | 1K1 |
| 2604 | 2014 | KT232191 | China | I | 1K | 1K1 |
| 2605 | 2014 | KT232192 | China | I | 1K | 1K1 |
| 2606 | 2014 | KT232193 | China | I | 1K | 1K1 |
| 2607 | 2014 | KT232194 | China | I | 1K | 1K1 |
| 2608 | 2014 | KT382295 | China | I | 1K | 1K1 |
| 2609 | 2014 | KT382297 | China | I | 1K | 1K1 |
| 2610 | 2014 | KT382298 | China | I | 1K | 1K1 |
| 2611 | 2014 | KT453224 | China | I | 1K | 1K1 |
| 2612 | 2014 | KT453225 | China | I | 1K | 1K1 |
| 2613 | 2014 | KT453226 | China | I | 1K | 1K1 |
| 2614 | 2014 | KT825023 | Australia | I | 1K | 1K1 |
| 2615 | 2014 | KT825025 | Australia | I | 1K | 1K1 |
| 2616 | 2014 | KT825032 | Australia | I | 1K | 1K1 |
| 2617 | 2014 | KT825062 | Australia | I | 1K | 1K1 |
| 2618 | 2014 | KT825064 | Australia | I | 1K | 1K1 |
| 2619 | 2014 | KT827372 | China | I | 1K | 1K1 |
| 2620 | 2014 | KT827376 | China | I | 1K | 1K1 |
| 2621 | 2014 | KU509293 | China | I | 1K | 1K1 |
| 2622 | 2014 | KU570115 | China | I | 1K | 1K1 |
| 2623 | 2014 | KU570116 | China | I | 1K | 1K1 |
| 2624 | 2014 | KU672342 | China | I | 1K | 1K1 |
| 2625 | 2014 | KX082952 | China | I | 1K | 1K1 |
| 2626 | 2014 | KX082953 | China | I | 1K | 1K1 |
| 2627 | 2014 | KX082954 | China | I | 1K | 1K1 |
| 2628 | 2014 | KX082955 | China | I | 1K | 1K1 |
| 2629 | 2014 | KX082957 | China | I | 1K | 1K1 |
| 2630 | 2014 | KX082958 | China | I | 1K | 1K1 |
| 2631 | 2014 | KX458013 | China | I | 1K | 1K1 |
| 2632 | 2014 | KX458014 | China | I | 1K | 1K1 |
| 2633 | 2014 | KX620455 | China | I | 1K | 1K1 |
| 2634 | 2014 | KX621252 | China | I | 1K | 1K1 |
| 2635 | 2015 | KT345174 | China | I | 1K | 1K1 |
| 2636 | 2015 | KT751340 | China | I | 1K | 1K1 |
| 2637 | 2015 | KT751341 | China | I | 1K | 1K1 |
| 2638 | 2015 | KT751344 | China | I | 1K | 1K1 |
| 2639 | 2015 | KT751345 | China | I | 1K | 1K1 |
| 2640 | 2015 | KT827379 | China | I | 1K | 1K1 |
| 2641 | 2008 | JF967796 | Malaysia | I | 1L | 1L1 |
| 2642 | 2009 | JF967876 | Malaysia | I | 1L | 1L1 |
| 2643 | 2009 | JF967877 | Indonesia | I | 1L | 1L1 |
| 2644 | 2009 | KX646378 | Indonesia | I | 1L | 1L1 |
| 2645 | 2009 | KX646379 | Indonesia | I | 1L | 1L1 |
| 2646 | 2010 | JF967911 | Malaysia | I | 1L | 1L1 |
| 2647 | 2010 | JF967920 | Malaysia | I | 1L | 1L1 |
| 2648 | 2010 | JN029807 | China | I | 1L | 1L1 |
| 2649 | 2010 | JN029811 | China | I | 1L | 1L1 |
| 2650 | 2010 | JN029813 | China | I | 1L | 1L1 |
| 2651 | 2010 | JN029814 | China | I | 1L | 1L1 |
| 2652 | 2010 | JQ317733 | China | I | 1L | 1L1 |
| 2653 | 2010 | KM216674 | Indonesia | I | 1L | 1L1 |
| 2654 | 2012 | KY818153 | Malaysia | I | 1L | 1L1 |
| 2655 | 2013 | KJ806949 | Singapore | I | 1L | 1L1 |
| 2656 | 2013 | KU509291 | Thailand | I | 1L | 1L1 |
| 2657 | 2014 | KJ806960 | Singapore | I | 1L | 1L1 |
| 2658 | 2014 | KJ806961 | Singapore | I | 1L | 1L1 |
| 2659 | 2014 | KU509292 | Vietnam | I | 1L | 1L1 |
| 2660 | 2014 | KX224258 | Singapore | I | 1L | 1L1 |
| 2661 | 2014 | KX224259 | Singapore | I | 1L | 1L1 |
| 2662 | 2014 | KX224260 | Singapore | I | 1L | 1L1 |
| 2663 | 2014 | KX224262 | Singapore | I | 1L | 1L1 |
| 2664 | 2014 | KX224263 | Singapore | I | 1L | 1L1 |
| 2665 | 2014 | KX224264 | Singapore | I | 1L | 1L1 |
| 2666 | 2014 | KX224265 | Singapore | I | 1L | 1L1 |
| 2667 | 2014 | KX452053 | Malaysia | I | 1L | 1L1 |
| 2668 | 2014 | KU570117 | China | I | 1L | 1L1 |
| 2669 | 2015 | KU529702 | Indonesia | I | 1L | 1L1 |
| 2670 | 2017 | GZ-29492 | China | I | 1L | 1L1 |
| 2671 | 2017 | GZ-40277 | China | I | 1L | 1L1 |
| 2672 | 2017 | GZ-8Y2 | China | I | 1L | 1L1 |
| 2673 | 2017 | GZ-8Y3 | China | I | 1L | 1L1 |
| 2674 | 2017 | GZ-8Y4 | China | I | 1L | 1L1 |
| 2675 | 2017 | GZ-8Y5 | China | I | 1L | 1L1 |
| 2676 | 2017 | GZ-CSH | China | I | 1L | 1L1 |
| 2677 | 2017 | GZ-HYY | China | I | 1L | 1L1 |
| 2678 | 2017 | GZ-LHQ | China | I | 1L | 1L1 |
| 2679 | 2017 | GZ-p1-consensus | China | I | 1L | 1L1 |
| 2680 | 2017 | GZ-xue-consensus | China | I | 1L | 1L1 |
| 2681 | 2017 | GZ-YLY | China | I | 1L | 1L1 |
| 2682 | 2017 | GZ-ZRH | China | I | 1L | 1L1 |
| 2683 | 2017 | MF681692 | China | I | 1L | 1L1 |
| 2684 | 2017 | MF681693 | China | I | 1L | 1L1 |
| 2685 | 2017 | MF683116 | China | I | 1L | 1L1 |
| 2686 | 2017 | MF683117 | China | I | 1L | 1L1 |
| 2687 | 2008 | GQ357687 | Singapore | I | 1L | 1L1 |
| 2688 | 2008 | JF967821 | Malaysia | I | 1L | 1L1 |
| 2689 | 2008 | JF967839 | Malaysia | I | 1L | 1L1 |
| 2690 | 2008 | JF967847 | Malaysia | I | 1L | 1L1 |
| 2691 | 2008 | JN415512 | Malaysia | I | 1L | 1L1 |
| 2692 | 2009 | JF960215 | Singapore | I | 1L | 1L1 |
| 2693 | 2009 | JF960216 | Singapore | I | 1L | 1L1 |
| 2694 | 2009 | JF960218 | Singapore | I | 1L | 1L1 |
| 2695 | 2009 | JF967887 | Malaysia | I | 1L | 1L1 |
| 2696 | 2010 | JF960223 | Singapore | I | 1L | 1L1 |
| 2697 | 2010 | JF960228 | Singapore | I | 1L | 1L1 |
| 2698 | 2010 | JF967897 | Malaysia | I | 1L | 1L1 |
| 2699 | 2012 | AB915381 | Indonesia | I | 1L | 1L1 |
| 2700 | 2012 | KT204438 | Indonesia | I | 1L | 1L1 |
| 2701 | 2012 | KT204440 | Indonesia | I | 1L | 1L1 |
| 2702 | 2012 | KT204441 | Indonesia | I | 1L | 1L1 |
| 2703 | 2012 | KT204445 | Indonesia | I | 1L | 1L1 |
| 2704 | 2012 | KT204448 | Indonesia | I | 1L | 1L1 |
| 2705 | 2012 | KT204450 | Indonesia | I | 1L | 1L1 |
| 2706 | 2013 | AB915380 | Indonesia | I | 1L | 1L1 |
| 2707 | 2013 | KT825000 | Indonesia | I | 1L | 1L1 |
| 2708 | 2013 | KY818145 | Indonesia | I | 1L | 1L1 |
| 2709 | 2014 | KT175086 | Malaysia | I | 1L | 1L1 |
| 2710 | 2014 | KT825061 | Indonesia | I | 1L | 1L1 |
| 2711 | 2014 | KY216156 | Indonesia | I | 1L | 1L1 |
| 2712 | 2014 | LC038149 | Indonesia | I | 1L | 1L1 |
| 2713 | 2014 | KJ806959 | Singapore | I | 1L | 1L1 |
| 2714 | 2014 | KR028435 | China | I | 1L | 1L1 |
| 2715 | 2014 | KT037107 | China | I | 1L | 1L1 |
| 2716 | 2014 | KT037108 | China | I | 1L | 1L1 |
| 2717 | 2014 | KT175084 | Malaysia | I | 1L | 1L1 |
| 2718 | 2014 | KT232183 | China | I | 1L | 1L1 |
| 2719 | 2014 | KT232186 | China | I | 1L | 1L1 |
| 2720 | 2014 | KT232187 | China | I | 1L | 1L1 |
| 2721 | 2014 | KT382304 | China | I | 1L | 1L1 |
| 2722 | 2014 | KT428609 | China | I | 1L | 1L1 |
| 2723 | 2014 | KT453227 | China | I | 1L | 1L1 |
| 2724 | 2014 | KT453228 | China | I | 1L | 1L1 |
| 2725 | 2014 | KT819303 | China | I | 1L | 1L1 |
| 2726 | 2014 | KT827373 | China | I | 1L | 1L1 |
| 2727 | 2014 | KU666939 | Malaysia | I | 1L | 1L1 |
| 2728 | 2014 | KX224261 | Singapore | I | 1L | 1L1 |
| 2729 | 2014 | KX225492 | China | I | 1L | 1L1 |
| 2730 | 2014 | KX225493 | China | I | 1L | 1L1 |
| 2731 | 2014 | KX621250 | China | I | 1L | 1L1 |
| 2732 | 2015 | KU529701 | Indonesia | I | 1L | 1L1 |
| 2733 | 2015 | KY006129 | Indonesia | I | 1L | 1L1 |
| 2734 | 2015 | KY006131 | Indonesia | I | 1L | 1L1 |
| 2735 | 2015 | KY709182 | Indonesia | I | 1L | 1L1 |
| 2736 | 2015 | KY709183 | Indonesia | I | 1L | 1L1 |
| 2737 | 2015 | KT825034 | Indonesia | I | 1L | 1L1 |
| 2738 | 2015 | KT825037 | Malaysia | I | 1L | 1L1 |
| 2739 | 2015 | KT825038 | Malaysia | I | 1L | 1L1 |
| 2740 | 2015 | KT825039 | Myanmar | I | 1L | 1L1 |
| 2741 | 2015 | KT825069 | Malaysia | I | 1L | 1L1 |
| 2742 | 2016 | GZ-14861 | China | I | 1L | 1L1 |
| 2743 | 2016 | GZ-15334 | China | I | 1L | 1L1 |
| 2744 | 2016 | GZ-18241 | China | I | 1L | 1L1 |
| 2745 | 2016 | GZ-545 | China | I | 1L | 1L1 |
| 2746 | 2016 | KX621253 | China | I | 1L | 1L1 |
| 2747 | 2016 | KY886976 | China | I | 1L | 1L1 |
| 2748 | 2016 | KY886977 | China | I | 1L | 1L1 |
| 2749 | 2016 | KY886978 | China | I | 1L | 1L1 |
| 2750 | 2016 | KY886979 | China | I | 1L | 1L1 |
| 2751 | 2016 | KY886980 | China | I | 1L | 1L1 |
| 2752 | 2013 | KJ806951 | Singapore | I | 1L | 1L1 |
| 2753 | 2013 | KT825052 | Indonesia | I | 1L | 1L1 |
| 2754 | 2014 | KP298004 | Japan | I | 1L | 1L1 |
| 2755 | 2014 | KR136279 | China | I | 1L | 1L1 |
| 2756 | 2014 | KR136280 | China | I | 1L | 1L1 |
| 2757 | 2014 | KR136281 | China | I | 1L | 1L1 |
| 2758 | 2014 | KR136282 | China | I | 1L | 1L1 |
| 2759 | 2014 | KR136283 | China | I | 1L | 1L1 |
| 2760 | 2014 | KR136284 | China | I | 1L | 1L1 |
| 2761 | 2014 | KR136285 | China | I | 1L | 1L1 |
| 2762 | 2014 | KR136286 | China | I | 1L | 1L1 |
| 2763 | 2014 | KR136287 | China | I | 1L | 1L1 |
| 2764 | 2014 | KR136288 | China | I | 1L | 1L1 |
| 2765 | 2014 | KR136289 | China | I | 1L | 1L1 |
| 2766 | 2014 | KR136290 | China | I | 1L | 1L1 |
| 2767 | 2014 | KR136291 | China | I | 1L | 1L1 |
| 2768 | 2014 | KR136292 | China | I | 1L | 1L1 |
| 2769 | 2014 | KR136293 | China | I | 1L | 1L1 |
| 2770 | 2014 | KR136294 | China | I | 1L | 1L1 |
| 2771 | 2014 | KR136295 | China | I | 1L | 1L1 |
| 2772 | 2014 | KR136296 | China | I | 1L | 1L1 |
| 2773 | 2014 | KR136297 | China | I | 1L | 1L1 |
| 2774 | 2014 | KR136298 | China | I | 1L | 1L1 |
| 2775 | 2014 | KR136299 | China | I | 1L | 1L1 |
| 2776 | 2014 | KR136300 | China | I | 1L | 1L1 |
| 2777 | 2014 | KR136301 | China | I | 1L | 1L1 |
| 2778 | 2014 | KR136302 | China | I | 1L | 1L1 |
| 2779 | 2014 | KR136303 | China | I | 1L | 1L1 |
| 2780 | 2014 | KT175080 | China | I | 1L | 1L1 |
| 2781 | 2014 | KT175092 | Indonesia | I | 1L | 1L1 |
| 2782 | 2014 | KT175093 | Indonesia | I | 1L | 1L1 |
| 2783 | 2014 | KT175094 | Indonesia | I | 1L | 1L1 |
| 2784 | 2014 | KT825021 | Indonesia | I | 1L | 1L1 |
| 2785 | 2014 | KT825022 | Indonesia | I | 1L | 1L1 |
| 2786 | 2014 | KT825060 | Indonesia | I | 1L | 1L1 |
| 2787 | 2014 | KU365900 | China | I | 1L | 1L1 |
| 2788 | 2014 | LC038144 | Indonesia | I | 1L | 1L1 |
| 2789 | 2014 | LC062957 | Japan | I | 1L | 1L1 |
| 2790 | 2015 | KY006130 | Indonesia | I | 1L | 1L1 |
| 2791 | 2015 | KY006137 | Indonesia | I | 1L | 1L1 |
| 2792 | 2005 | EF654105 | Indonesia | I | 1M | 1M1 |
| 2793 | 2005 | EF654106 | Indonesia | I | 1M | 1M1 |
| 2794 | 2005 | EF654107 | Indonesia | I | 1M | 1M1 |
| 2795 | 2005 | EU069604 | Singapore | I | 1M | 1M1 |
| 2796 | 2005 | KP406802 | Indonesia | I | 1M | 1M1 |
| 2797 | 2007 | KC762623 | Indonesia | I | 1M | 1M1 |
| 2798 | 2007 | KC762625 | Indonesia | I | 1M | 1M1 |
| 2799 | 2007 | KC762632 | Indonesia | I | 1M | 1M1 |
| 2800 | 2007 | KC762635 | Indonesia | I | 1M | 1M1 |
| 2801 | 2007 | KC762636 | Indonesia | I | 1M | 1M1 |
| 2802 | 2007 | KC762637 | Indonesia | I | 1M | 1M1 |
| 2803 | 2007 | KC762646 | Indonesia | I | 1M | 1M1 |
| 2804 | 2007 | KC762649 | Indonesia | I | 1M | 1M1 |
| 2805 | 2007 | KC762650 | Indonesia | I | 1M | 1M1 |
| 2806 | 2007 | KC762654 | Indonesia | I | 1M | 1M1 |
| 2807 | 2008 | KC762622 | Indonesia | I | 1M | 1M1 |
| 2808 | 2008 | KC762627 | Indonesia | I | 1M | 1M1 |
| 2809 | 2008 | KC762629 | Indonesia | I | 1M | 1M1 |
| 2810 | 2008 | KC762631 | Indonesia | I | 1M | 1M1 |
| 2811 | 2008 | KC762633 | Indonesia | I | 1M | 1M1 |
| 2812 | 2008 | KC762634 | Indonesia | I | 1M | 1M1 |
| 2813 | 2008 | KC762638 | Indonesia | I | 1M | 1M1 |
| 2814 | 2008 | KC762641 | Indonesia | I | 1M | 1M1 |
| 2815 | 2008 | KC762643 | Indonesia | I | 1M | 1M1 |
| 2816 | 2008 | KC762644 | Indonesia | I | 1M | 1M1 |
| 2817 | 2008 | KC762645 | Indonesia | I | 1M | 1M1 |
| 2818 | 2008 | KC762648 | Indonesia | I | 1M | 1M1 |
| 2819 | 2008 | KC762652 | Indonesia | I | 1M | 1M1 |
| 2820 | 2008 | KC762621 | Indonesia | I | 1M | 1M1 |
| 2821 | 2008 | KC762624 | Indonesia | I | 1M | 1M1 |
| 2822 | 2008 | KC762626 | Indonesia | I | 1M | 1M1 |
| 2823 | 2009 | JN415519 | Papua_New_Guinea | I | 1M | 1M1 |
| 2824 | 2010 | KC762639 | Indonesia | I | 1M | 1M1 |
| 2825 | 2010 | KC762647 | Indonesia | I | 1M | 1M1 |
| 2826 | 2011 | KT824977 | Papua_New_Guinea | I | 1M | 1M1 |
| 2827 | 2011 | KM216690 | Indonesia | I | 1M | 1M1 |
| 2828 | 2011 | KM216691 | Indonesia | I | 1M | 1M1 |
| 2829 | 2014 | KT175095 | Indonesia | I | 1M | 1M1 |
| 2830 | 2015 | KY006133 | Indonesia | I | 1M | 1M1 |
| 2831 | 2007 | EU448401 | Indonesia | I | 1M | 1M2 |
| 2832 | 2008 | FJ687477 | Indonesia | I | 1M | 1M2 |
| 2833 | 2008 | JF967825 | Indonesia | I | 1M | 1M2 |
| 2834 | 2010 | JF967903 | Indonesia | I | 1M | 1M2 |
| 2835 | 2010 | JF967904 | Indonesia | I | 1M | 1M2 |
| 2836 | 2010 | JF967905 | Indonesia | I | 1M | 1M2 |
| 2837 | 2010 | JF967907 | Indonesia | I | 1M | 1M2 |
| 2838 | 2010 | JF967908 | Indonesia | I | 1M | 1M2 |
| 2839 | 2010 | JF967922 | Indonesia | I | 1M | 1M2 |
| 2840 | 2010 | JF967926 | Thailand | I | 1M | 1M2 |
| 2841 | 2010 | JF967947 | Indonesia | I | 1M | 1M2 |
| 2842 | 2010 | JN415490 | Indonesia | I | 1M | 1M2 |
| 2843 | 2010 | KM216670 | Indonesia | I | 1M | 1M2 |
| 2844 | 2010 | KM216681 | Indonesia | I | 1M | 1M2 |
| 2845 | 2011 | KM216689 | Indonesia | I | 1M | 1M2 |
| 2846 | 2011 | KT824970 | Indonesia | I | 1M | 1M2 |
| 2847 | 2012 | KC589008 | Indonesia | I | 1M | 1M2 |
| 2848 | 2012 | KT204436 | Indonesia | I | 1M | 1M2 |
| 2849 | 2009 | HQ149730 | China | I | 1M | 1M3 |
| 2850 | 2009 | JF967857 | Indonesia | I | 1M | 1M3 |
| 2851 | 2009 | JF967866 | Indonesia | I | 1M | 1M3 |
| 2852 | 2010 | JF960234 | Singapore | I | 1M | 1M3 |
| 2853 | 2010 | JF967931 | Indonesia | I | 1M | 1M3 |
| 2854 | 2013 | KY818092 | Indonesia | I | 1M | 1M3 |
| 2855 | 2014 | KT825066 | NA | I | 1M | 1M3 |
| 2856 | 2010 | JF967900 | Indonesia | I | 1M | 1M4 |
| 2857 | 2010 | JF967901 | Indonesia | I | 1M | 1M4 |
| 2858 | 2010 | KM216680 | Indonesia | I | 1M | 1M4 |
| 2859 | 2010 | KU509253 | Indonesia | I | 1M | 1M4 |
| 2860 | 2015 | KU529712 | Indonesia | I | 1M | 1M5 |
| 2861 | 2013 | KY818138 | Indonesia | I | 1M | 1M6 |
| 2862 | 2010 | JF960225 | Singapore | I | 1M | 1M7 |
| 2863 | 2010 | JF960226 | Singapore | I | 1M | 1M7 |
| 2864 | 2010 | JF960227 | Singapore | I | 1M | 1M7 |
| 2865 | 2010 | JF960229 | Singapore | I | 1M | 1M7 |
| 2866 | 2010 | JF960230 | Singapore | I | 1M | 1M7 |
| 2867 | 2010 | JF960231 | Singapore | I | 1M | 1M7 |
| 2868 | 2010 | JF960232 | Singapore | I | 1M | 1M7 |
| 2869 | 2010 | JF960233 | Singapore | I | 1M | 1M7 |
| 2870 | 2012 | KT204442 | Indonesia | I | 1M | 1M7 |
| 2871 | 2012 | KT204446 | Indonesia | I | 1M | 1M7 |
| 2872 | 2013 | AB915378 | Indonesia | I | 1M | 1M7 |
| 2873 | 2013 | AB915379 | Indonesia | I | 1M | 1M7 |
| 2874 | 2013 | KY818091 | Indonesia | I | 1M | 1M7 |
| 2875 | 2013 | KY818093 | Indonesia | I | 1M | 1M7 |
| 2876 | 2008 | GQ357666 | Singapore | I | 1M | 1M8 |
| 2877 | 2008 | GQ357667 | Singapore | I | 1M | 1M8 |
| 2878 | 2008 | GQ357668 | Singapore | I | 1M | 1M8 |
| 2879 | 2008 | GQ357669 | Singapore | I | 1M | 1M8 |
| 2880 | 2008 | GQ357670 | Singapore | I | 1M | 1M8 |
| 2881 | 2008 | GQ357671 | Singapore | I | 1M | 1M8 |
| 2882 | 2008 | GQ357672 | Singapore | I | 1M | 1M8 |
| 2883 | 2008 | GQ357673 | Singapore | I | 1M | 1M8 |
| 2884 | 2008 | GQ357674 | Singapore | I | 1M | 1M8 |
| 2885 | 2008 | GQ357675 | Singapore | I | 1M | 1M8 |
| 2886 | 2008 | GQ357676 | Singapore | I | 1M | 1M8 |
| 2887 | 2008 | GQ357677 | Singapore | I | 1M | 1M8 |
| 2888 | 2008 | GQ357678 | Singapore | I | 1M | 1M8 |
| 2889 | 2008 | GQ357679 | Singapore | I | 1M | 1M8 |
| 2890 | 2008 | GQ357680 | Singapore | I | 1M | 1M8 |
| 2891 | 2008 | GQ398255 | Singapore | I | 1M | 1M8 |
| 2892 | 2008 | GU370048 | Singapore | I | 1M | 1M8 |
| 2893 | 2008 | JF967824 | Singapore | I | 1M | 1M8 |
| 2894 | 2008 | JN022600 | Singapore | I | 1M | 1M8 |
| 2895 | 2008 | JN196565 | Singapore | I | 1M | 1M8 |
| 2896 | 2008 | JN415521 | Singapore | I | 1M | 1M8 |
| 2897 | 2008 | JN415531 | Australia | I | 1M | 1M8 |
| 2898 | 2008 | JF967813 | Singapore | I | 1M | 1M8 |
| 2899 | 2008 | JF967832 | Indonesia | I | 1M | 1M8 |
| 2900 | 2009 | JF960210 | Singapore | I | 1M | 1M8 |
| 2901 | 2009 | JF960213 | Singapore | I | 1M | 1M8 |
| 2902 | 2009 | JN380803 | Singapore | I | 1M | 1M8 |
| 2903 | 2009 | JN380804 | Singapore | I | 1M | 1M8 |
| 2904 | 2009 | JN380805 | Singapore | I | 1M | 1M8 |
| 2905 | 2010 | KC762642 | Indonesia | I | 1M | 1M8 |
| 2906 | 2012 | KC589009 | Indonesia | I | 1M | 1M8 |
| 2907 | 2012 | KT204443 | Indonesia | I | 1M | 1M8 |
| 2908 | 2014 | KT175090 | Indonesia | I | 1M | 1M8 |
| 2909 | 2014 | KT175091 | Indonesia | I | 1M | 1M8 |
| 2910 | 2015 | KU529738 | Indonesia | I | 1M | 1M8 |
| 2911 | 2014 | KU529691 | Indonesia | I | 1M | 1M9 |
| 2912 | 2014 | KU529692 | Indonesia | I | 1M | 1M9 |
| 2913 | 2014 | KU529693 | Indonesia | I | 1M | 1M9 |
| 2914 | 2014 | KU529694 | Indonesia | I | 1M | 1M9 |
| 2915 | 2014 | KU529695 | Indonesia | I | 1M | 1M9 |
| 2916 | 2014 | KU529697 | Indonesia | I | 1M | 1M9 |
| 2917 | 2014 | KU529699 | Indonesia | I | 1M | 1M9 |
| 2918 | 2014 | KU529703 | Indonesia | I | 1M | 1M9 |
| 2919 | 2014 | KU529731 | Indonesia | I | 1M | 1M9 |
| 2920 | 2014 | KU529698 | Indonesia | I | 1M | 1M9 |
| 2921 | 2015 | KU529700 | Indonesia | I | 1M | 1M9 |
| 2922 | 2015 | KU529704 | Indonesia | I | 1M | 1M9 |
| 2923 | 2015 | KU529705 | Indonesia | I | 1M | 1M9 |
| 2924 | 2015 | KU529706 | Indonesia | I | 1M | 1M9 |
| 2925 | 2015 | KU529707 | Indonesia | I | 1M | 1M9 |
| 2926 | 2015 | KU529708 | Indonesia | I | 1M | 1M9 |
| 2927 | 2015 | KU529713 | Indonesia | I | 1M | 1M9 |
| 2928 | 2015 | KU529714 | Indonesia | I | 1M | 1M9 |
| 2929 | 2015 | KU529715 | Indonesia | I | 1M | 1M9 |
| 2930 | 2015 | KU529716 | Indonesia | I | 1M | 1M9 |
| 2931 | 2015 | KU529717 | Indonesia | I | 1M | 1M9 |
| 2932 | 2015 | KU529718 | Indonesia | I | 1M | 1M9 |
| 2933 | 2015 | KU529719 | Indonesia | I | 1M | 1M9 |
| 2934 | 2015 | KU529720 | Indonesia | I | 1M | 1M9 |
| 2935 | 2015 | KU529721 | Indonesia | I | 1M | 1M9 |
| 2936 | 2015 | KU529722 | Indonesia | I | 1M | 1M9 |
| 2937 | 2015 | KU529723 | Indonesia | I | 1M | 1M9 |
| 2938 | 2015 | KU529724 | Indonesia | I | 1M | 1M9 |
| 2939 | 2015 | KU529725 | Indonesia | I | 1M | 1M9 |
| 2940 | 2015 | KU529726 | Indonesia | I | 1M | 1M9 |
| 2941 | 2015 | KU529728 | Indonesia | I | 1M | 1M9 |
| 2942 | 2015 | KU529730 | Indonesia | I | 1M | 1M9 |
| 2943 | 2015 | KU529732 | Indonesia | I | 1M | 1M9 |
| 2944 | 2015 | KU529734 | Indonesia | I | 1M | 1M9 |
| 2945 | 2015 | KU529735 | Indonesia | I | 1M | 1M9 |
| 2946 | 2015 | KU529709 | Indonesia | I | 1M | 1M9 |
| 2947 | 2015 | KU529710 | Indonesia | I | 1M | 1M9 |
| 2948 | 2015 | KU529711 | Indonesia | I | 1M | 1M9 |
| 2949 | 2015 | KU529729 | Indonesia | I | 1M | 1M9 |
| 2950 | 2015 | KU529733 | Indonesia | I | 1M | 1M9 |
| 2951 | 2015 | KU529736 | Indonesia | I | 1M | 1M9 |
| 2952 | 2015 | KU529737 | Indonesia | I | 1M | 1M9 |
| 2953 | 1958 | D10513 | Thailand | II | 2A | 2A1 |
| 2954 | 1960 | JF297570 | Thailand | II | 2A | 2A1 |
| 2955 | 1963 | AF425629 | Thailand | II | 2A | 2A1 |
| 2956 | 2012 | KC589010 | Indonesia | II | 2A | 2A1 |
| 2957 | 1972 | AF425622 | Malaysia | III | 3A | 3A1 |
| 2958 | 2005 | FN825674 | Malaysia | III | 3A | 3A1 |
| 2959 | 1974 | AF425627 | Philippines | IV | 4A | 4A1 |
| 2960 | 1973 | U88535 | Nauru_Island | IV | 4A | 4A2 |
| 2961 | 1984 | D00503 | Philippines | IV | 4A | 4A2 |
| 2962 | 1991 | FJ196845 | China | IV | 4A | 4A3 |
| 2963 | 1991 | JN029816 | China | IV | 4A | 4A3 |
| 2964 | 1995 | AY422784 | Philippines | IV | 4A | 4A4 |
| 2965 | 2000 | JN415515 | Palau | IV | 4A | 4A5 |
| 2966 | 2002 | AY422778 | Philippines | IV | 4A | 4A6 |
| 2967 | 2002 | AY422781 | Philippines | IV | 4A | 4A7 |
| 2968 | 2001 | AY422777 | Philippines | IV | 4A | 4A8 |
| 2969 | 1999 | AY422785 | Philippines | IV | 4A | 4A9 |
| 2970 | 1999 | AY422786 | Philippines | IV | 4A | 4A9 |
| 2971 | 2001 | AB111068 | Samoa | IV | 4A | 4A10 |
| 2972 | 2001 | DQ091261 | USA.Hawaii | IV | 4A | 4A10 |
| 2973 | 2001 | DQ672564 | USA.Hawaii | IV | 4A | 4A10 |
| 2974 | 2001 | JN415520 | Samoa | IV | 4A | 4A10 |
| 2975 | 2001 | JQ655095 | Samoa | IV | 4A | 4A10 |
| 2976 | 2002 | AY422779 | Philippines | IV | 4A | 4A10 |
| 2977 | 2002 | AY422780 | Philippines | IV | 4A | 4A10 |
| 2978 | 2002 | AY422782 | Philippines | IV | 4A | 4A10 |
| 2979 | 2002 | AY422783 | Philippines | IV | 4A | 4A10 |
| 2980 | 2003 | EU448406 | Philippines | IV | 4A | 4A10 |
| 2981 | 2004 | GQ868602 | Philippines | IV | 4A | 4A10 |
| 2982 | 2004 | KX951689 | China | IV | 4A | 4A10 |
| 2983 | 2005 | JN415516 | Philippines | IV | 4A | 4A10 |
| 2984 | 2002 | AB111074 | Philippines | IV | 4A | 4A11 |
| 2985 | 2008 | KJ946238 | Philippines | IV | 4A | 4A12 |
| 2986 | 2006 | EF654110 | Philippines | IV | 4A | 4A13 |
| 2987 | 2006 | KP406803 | Philippines | IV | 4A | 4A13 |
| 2988 | 2007 | EU448405 | Philippines | IV | 4A | 4A14 |
| 2989 | 2008 | KJ946237 | Philippines | IV | 4A | 4A14 |
| 2990 | 2010 | JF967936 | Philippines | IV | 4A | 4A14 |
| 2991 | 2010 | JF967938 | Philippines | IV | 4A | 4A14 |
| 2992 | 2011 | KT825047 | Philippines | IV | 4A | 4A14 |
| 2993 | 2011 | KY818103 | Philippines | IV | 4A | 4A14 |
| 2994 | 2011 | KY818104 | Philippines | IV | 4A | 4A14 |
| 2995 | 2011 | KY818130 | Philippines | IV | 4A | 4A14 |
| 2996 | 2011 | KY818162 | Philippines | IV | 4A | 4A14 |
| 2997 | 2012 | KY818108 | Philippines | IV | 4A | 4A14 |
| 2998 | 2012 | KY818112 | Philippines | IV | 4A | 4A14 |
| 2999 | 2012 | KY818113 | Philippines | IV | 4A | 4A14 |
| 3000 | 2012 | KY818118 | Philippines | IV | 4A | 4A14 |
| 3001 | 2012 | KY818120 | Philippines | IV | 4A | 4A14 |
| 3002 | 2012 | KY818124 | Philippines | IV | 4A | 4A14 |
| 3003 | 2012 | KY818125 | Philippines | IV | 4A | 4A14 |
| 3004 | 2012 | KY818129 | Philippines | IV | 4A | 4A14 |
| 3005 | 2012 | KY818131 | Philippines | IV | 4A | 4A14 |
| 3006 | 2012 | KY818183 | Philippines | IV | 4A | 4A14 |
| 3007 | 2012 | KY882513 | Philippines | IV | 4A | 4A14 |
| 3008 | 2012 | KY818166 | Philippines | IV | 4A | 4A14 |
| 3009 | 2012 | KY818167 | Philippines | IV | 4A | 4A14 |
| 3010 | 2012 | KY818168 | Philippines | IV | 4A | 4A14 |
| 3011 | 2012 | KY818172 | Philippines | IV | 4A | 4A14 |
| 3012 | 2012 | KY818180 | Philippines | IV | 4A | 4A14 |
| 3013 | 2012 | KY818185 | Philippines | IV | 4A | 4A14 |
| 3014 | 2012 | KY818195 | Philippines | IV | 4A | 4A14 |
| 3015 | 2012 | KY818199 | Philippines | IV | 4A | 4A14 |
| 3016 | 2012 | KY818204 | Philippines | IV | 4A | 4A14 |
| 3017 | 2013 | KT825007 | Philippines | IV | 4A | 4A14 |
| 3018 | 2013 | KU509315 | Philippines | IV | 4A | 4A14 |
| 3019 | 2013 | KU509316 | Thailand | IV | 4A | 4A14 |
| 3020 | 2013 | KY818121 | Philippines | IV | 4A | 4A14 |
| 3021 | 2013 | KY818123 | Philippines | IV | 4A | 4A14 |
| 3022 | 2013 | KY818126 | Philippines | IV | 4A | 4A14 |
| 3023 | 2013 | KY818127 | Philippines | IV | 4A | 4A14 |
| 3024 | 2013 | KU570101 | China | IV | 4A | 4A14 |
| 3025 | 2013 | KY818173 | Philippines | IV | 4A | 4A14 |
| 3026 | 2013 | KY818176 | Philippines | IV | 4A | 4A14 |
| 3027 | 2013 | KY818178 | Philippines | IV | 4A | 4A14 |
| 3028 | 2013 | KY818179 | Philippines | IV | 4A | 4A14 |
| 3029 | 2013 | KY818200 | Philippines | IV | 4A | 4A14 |
| 3030 | 2013 | KY818201 | Philippines | IV | 4A | 4A14 |
| 3031 | 2014 | KT175098 | Philippines | IV | 4A | 4A14 |
| 3032 | 2016 | KY495796 | Philippines | IV | 4A | 4A14 |
| 3033 | 2016 | LC128301 | Philippines | IV | 4A | 4A14 |
| 3034 | 2003 | EU448404 | Indonesia | IV | 4B | 4B1 |
| 3035 | 2004 | AY858983 | Indonesia | IV | 4B | 4B1 |
| 3036 | 2009 | JF967865 | Indonesia | IV | 4B | 4B1 |
| 3037 | 2012 | AB915368 | Indonesia | IV | 4B | 4B2 |
| 3038 | 2012 | AB915370 | Indonesia | IV | 4B | 4B2 |
| 3039 | 2012 | KT204437 | Indonesia | IV | 4B | 4B2 |
| 3040 | 2013 | AB915369 | Indonesia | IV | 4B | 4B2 |
| 3041 | 2006 | EU448402 | Indonesia | IV | 4B | 4B3 |
| 3042 | 2006 | EU448403 | Vietnam | IV | 4B | 4B3 |
| 3043 | 2008 | KC762628 | Indonesia | IV | 4B | 4B3 |
| 3044 | 2008 | KC762653 | Indonesia | IV | 4B | 4B3 |
| 3045 | 2008 | KC762640 | Indonesia | IV | 4B | 4B3 |
| 3046 | 2010 | JF967949 | Indonesia | IV | 4B | 4B3 |
| 3047 | 2010 | JN029810 | China | IV | 4B | 4B3 |
| 3048 | 2010 | JN415492 | Indonesia | IV | 4B | 4B3 |
| 3049 | 2010 | KM216664 | Indonesia | IV | 4B | 4B3 |
| 3050 | 2010 | KM216665 | Indonesia | IV | 4B | 4B3 |
| 3051 | 2010 | KM216669 | Indonesia | IV | 4B | 4B3 |
| 3052 | 2010 | KM216671 | Indonesia | IV | 4B | 4B3 |
| 3053 | 2010 | KM216673 | Indonesia | IV | 4B | 4B3 |
| 3054 | 2010 | KM216677 | Indonesia | IV | 4B | 4B3 |
| 3055 | 2010 | KT824966 | Indonesia | IV | 4B | 4B3 |
| 3056 | 2010 | KT204461 | Indonesia | IV | 4B | 4B3 |
| 3057 | 2011 | KT824972 | Indonesia | IV | 4B | 4B3 |
| 3058 | 2011 | KT824973 | Indonesia | IV | 4B | 4B3 |
| 3059 | 2011 | KT824982 | Australia | IV | 4B | 4B3 |
| 3060 | 2012 | AB915366 | Indonesia | IV | 4B | 4B3 |
| 3061 | 2012 | AB915367 | Indonesia | IV | 4B | 4B3 |
| 3062 | 2012 | KT204453 | Indonesia | IV | 4B | 4B3 |
| 3063 | 2012 | KT204439 | Indonesia | IV | 4B | 4B3 |
| 3064 | 2012 | KT204444 | Indonesia | IV | 4B | 4B3 |
| 3065 | 2012 | KT204447 | Indonesia | IV | 4B | 4B3 |
| 3066 | 2013 | KY818090 | Indonesia | IV | 4B | 4B3 |
| 3067 | 2013 | AB915382 | Indonesia | IV | 4B | 4B3 |
| 3068 | 2013 | AB915383 | Indonesia | IV | 4B | 4B3 |
| 3069 | 2013 | AB915384 | Indonesia | IV | 4B | 4B3 |
| 3070 | 2010 | KT827367 | China | IV | 4C | 4C1 |
| 3071 | 2011 | KY818169 | Philippines | IV | 4C | 4C1 |
| 3072 | 2011 | KY818116 | Philippines | IV | 4C | 4C1 |
| 3073 | 2012 | KJ933413 | China | IV | 4C | 4C1 |
| 3074 | 2012 | KX380797 | Singapore | IV | 4C | 4C1 |
| 3075 | 2012 | KY818164 | Philippines | IV | 4C | 4C1 |
| 3076 | 2012 | KY818175 | Philippines | IV | 4C | 4C1 |
| 3077 | 2012 | KY818181 | Philippines | IV | 4C | 4C1 |
| 3078 | 2012 | KY882506 | Philippines | IV | 4C | 4C1 |
| 3079 | 2012 | KY882512 | Philippines | IV | 4C | 4C1 |
| 3080 | 2013 | KJ806939 | Singapore | IV | 4C | 4C1 |
| 3081 | 2013 | KY818202 | Philippines | IV | 4C | 4C1 |
| 3082 | 2013 | KY882508 | Philippines | IV | 4C | 4C1 |
| 3083 | 2013 | KY882509 | Philippines | IV | 4C | 4C1 |
| 3084 | 2013 | KY882511 | Philippines | IV | 4C | 4C1 |
| 3085 | 2014 | KT175096 | Philippines | IV | 4C | 4C1 |
| 3086 | 2016 | KY496854 | China | IV | 4C | 4C1 |
| 3087 | 2016 | KY496855 | China | IV | 4C | 4C1 |
| 3088 | 2011 | KY818161 | Philippines | IV | 4C | 4C2 |
| 3089 | 2011 | KY818170 | Philippines | IV | 4C | 4C2 |
| 3090 | 2011 | KY818171 | Philippines | IV | 4C | 4C2 |
| 3091 | 2011 | KY882505 | Philippines | IV | 4C | 4C2 |
| 3092 | 2012 | KT824988 | Philippines | IV | 4C | 4C2 |
| 3093 | 2012 | KY818163 | Philippines | IV | 4C | 4C2 |
| 3094 | 2012 | KY818165 | Philippines | IV | 4C | 4C2 |
| 3095 | 2012 | KY818182 | Philippines | IV | 4C | 4C2 |
| 3096 | 2012 | KY818189 | Philippines | IV | 4C | 4C2 |
| 3097 | 2012 | KY818190 | Philippines | IV | 4C | 4C2 |
| 3098 | 2012 | KY818192 | Philippines | IV | 4C | 4C2 |
| 3099 | 2012 | KY818207 | Philippines | IV | 4C | 4C2 |
| 3100 | 2012 | KY882507 | Philippines | IV | 4C | 4C2 |
| 3101 | 2012 | LC148027 | Philippines | IV | 4C | 4C2 |
| 3102 | 2013 | KY818174 | Philippines | IV | 4C | 4C2 |
| 3103 | 2013 | KY818177 | Philippines | IV | 4C | 4C2 |
| 3104 | 2013 | KY818184 | Philippines | IV | 4C | 4C2 |
| 3105 | 2013 | KY818196 | Philippines | IV | 4C | 4C2 |
| 3106 | 2013 | KY818205 | Philippines | IV | 4C | 4C2 |
| 3107 | 2013 | KY882510 | Philippines | IV | 4C | 4C2 |
| 3108 | 2013 | KY818193 | Philippines | IV | 4C | 4C2 |
| 3109 | 2014 | KT175097 | Philippines | IV | 4C | 4C2 |
| 3110 | 2014 | KT175099 | Philippines | IV | 4C | 4C2 |
| 3111 | 2007 | FJ687476 | Philippines | IV | 4C | 4C3 |
| 3112 | 2010 | JF967937 | Philippines | IV | 4C | 4C3 |
| 3113 | 2010 | JN415517 | Philippines | IV | 4C | 4C3 |
| 3114 | 2011 | KY818102 | Philippines | IV | 4C | 4C3 |
| 3115 | 2011 | LC148026 | Philippines | IV | 4C | 4C3 |
| 3116 | 2012 | KT824989 | Philippines | IV | 4C | 4C3 |
| 3117 | 2012 | KY818105 | Philippines | IV | 4C | 4C3 |
| 3118 | 2012 | KY818106 | Philippines | IV | 4C | 4C3 |
| 3119 | 2012 | KY818107 | Philippines | IV | 4C | 4C3 |
| 3120 | 2012 | KY818109 | Philippines | IV | 4C | 4C3 |
| 3121 | 2012 | KY818110 | Philippines | IV | 4C | 4C3 |
| 3122 | 2012 | KY818111 | Philippines | IV | 4C | 4C3 |
| 3123 | 2012 | KY818114 | Philippines | IV | 4C | 4C3 |
| 3124 | 2012 | KY818115 | Philippines | IV | 4C | 4C3 |
| 3125 | 2012 | KY818117 | Philippines | IV | 4C | 4C3 |
| 3126 | 2012 | KY818122 | Philippines | IV | 4C | 4C3 |
| 3127 | 2012 | KY818186 | Philippines | IV | 4C | 4C3 |
| 3128 | 2012 | KY818191 | Philippines | IV | 4C | 4C3 |
| 3129 | 2012 | KY818206 | Philippines | IV | 4C | 4C3 |
| 3130 | 2012 | KY882502 | Philippines | IV | 4C | 4C3 |
| 3131 | 2013 | KJ415096 | Angola | IV | 4C | 4C3 |
| 3132 | 2013 | KJ415098 | Philippines | IV | 4C | 4C3 |
| 3133 | 2013 | KY818119 | Philippines | IV | 4C | 4C3 |
| 3134 | 2013 | KY818128 | Philippines | IV | 4C | 4C3 |
| 3135 | 2013 | KY818187 | Philippines | IV | 4C | 4C3 |
| 3136 | 2013 | KY818188 | Philippines | IV | 4C | 4C3 |
| 3137 | 2013 | KY818194 | Philippines | IV | 4C | 4C3 |
| 3138 | 2013 | KY818197 | Philippines | IV | 4C | 4C3 |
| 3139 | 2013 | KY818198 | Philippines | IV | 4C | 4C3 |
| 3140 | 2013 | KY818203 | Philippines | IV | 4C | 4C3 |
| 3141 | 2013 | KY818208 | Philippines | IV | 4C | 4C3 |
| 3142 | 2014 | KT175081 | China | IV | 4C | 4C3 |
| 3143 | 2015 | KT825036 | Australia | IV | 4C | 4C3 |
| 3144 | 2015 | KT825043 | Australia | IV | 4C | 4C3 |
| 3145 | 2015 | KU310948 | China | IV | 4C | 4C3 |
| 3146 | 1983 | AF425611 | Australia | IV | 4D | 4D1 |
| 3147 | 1983 | AF425612 | Australia | IV | 4D | 4D1 |
| 3148 | 2007 | KC762620 | Indonesia | IV | 4D | 4D2 |
| 3149 | 2007 | KC762651 | Indonesia | IV | 4D | 4D2 |
| 3150 | 2000 | EF440432 | East_Timor | IV | 4D | 4D3 |
| 3151 | 2000 | JN415499 | East_Timor | IV | 4D | 4D3 |
| 3152 | 2001 | KY275186 | East_Timor | IV | 4D | 4D3 |
| 3153 | 2001 | KY275187 | East_Timor | IV | 4D | 4D3 |
| 3154 | 2001 | KY275196 | East_Timor | IV | 4D | 4D3 |
| 3155 | 2002 | KY275188 | East_Timor | IV | 4D | 4D3 |
| 3156 | 2005 | AB219136 | East_Timor | IV | 4D | 4D3 |
| 3157 | 2008 | JN415500 | East_Timor | IV | 4D | 4D3 |
| 3158 | 2009 | JN415501 | East_Timor | IV | 4D | 4D3 |
| 3159 | 2009 | JN415532 | Australia | IV | 4D | 4D3 |
| 3160 | 2009 | KT825045 | Australia | IV | 4D | 4D3 |
| 3161 | 2010 | JF967946 | Indonesia | IV | 4D | 4D3 |
| 3162 | 2010 | JN415502 | East_Timor | IV | 4D | 4D3 |
| 3163 | 2010 | JN415510 | Indonesia | IV | 4D | 4D3 |
| 3164 | 2010 | KT824967 | East_Timor | IV | 4D | 4D3 |
| 3165 | 2010 | KT824968 | East_Timor | IV | 4D | 4D3 |
| 3166 | 2010 | KY275183 | East_Timor | IV | 4D | 4D3 |
| 3167 | 2010 | KY275184 | East_Timor | IV | 4D | 4D3 |
| 3168 | 2010 | KY275185 | East_Timor | IV | 4D | 4D3 |
| 3169 | 2010 | KY275189 | East_Timor | IV | 4D | 4D3 |
| 3170 | 2010 | KY275191 | East_Timor | IV | 4D | 4D3 |
| 3171 | 2010 | KY275192 | East_Timor | IV | 4D | 4D3 |
| 3172 | 2010 | KY275193 | East_Timor | IV | 4D | 4D3 |
| 3173 | 2010 | KY275194 | East_Timor | IV | 4D | 4D3 |
| 3174 | 2010 | KY275195 | East_Timor | IV | 4D | 4D3 |
| 3175 | 2010 | KY275197 | East_Timor | IV | 4D | 4D3 |
| 3176 | 1998 | AB189121 | Indonesia | IV | 4E | 4E1 |
| 3177 | 2005 | EU448407 | Indonesia | IV | 4E | 4E1 |
| 3178 | 2009 | JF967867 | Indonesia | IV | 4E | 4E1 |
| 3179 | 2010 | JF967925 | Indonesia | IV | 4E | 4E1 |
| 3180 | 2010 | JF967930 | Indonesia | IV | 4E | 4E1 |
| 3181 | 2010 | KU509261 | Indonesia | IV | 4E | 4E1 |
| 3182 | 2011 | JN544411 | Singapore | IV | 4E | 4E1 |
| 3183 | 2011 | KT824971 | Indonesia | IV | 4E | 4E1 |
| 3184 | 2011 | KT824976 | Australia | IV | 4E | 4E1 |
| 3185 | 2002 | JF459993 | Myanmar | IV | 4E | 4E2 |
| 3186 | 2010 | JF967918 | Indonesia | IV | 4E | 4E3 |
| 3187 | 1993 | DQ211348 | China | IV | 4E | 4E4 |
| 3188 | 1993 | DQ211349 | China | IV | 4E | 4E4 |
| 3189 | 2002 | DQ855296 | China | IV | 4E | 4E4 |
| 3190 | 2002 | EF025110 | China | IV | 4E | 4E4 |
| 3191 | 2002 | EF079826 | China | IV | 4E | 4E4 |
| 3192 | 2002 | EF508201 | China | IV | 4E | 4E4 |
| 3193 | 2002 | JN205310 | China | IV | 4E | 4E4 |
| 3194 | 2002 | JQ317714 | China | IV | 4E | 4E4 |
| 3195 | 2002 | JQ317715 | China | IV | 4E | 4E4 |
| 3196 | 2002 | JQ317717 | China | IV | 4E | 4E4 |
| 3197 | 2002 | JQ317747 | China | IV | 4E | 4E4 |
| 3198 | 2002 | JQ317748 | China | IV | 4E | 4E4 |
| 3199 | 2002 | JQ317749 | China | IV | 4E | 4E4 |
| 3200 | 2002 | JQ317750 | China | IV | 4E | 4E4 |
| 3201 | 2002 | JQ317751 | China | IV | 4E | 4E4 |
| 3202 | 2002 | KT827364 | China | IV | 4E | 4E4 |
| 3203 | 2003 | EU069611 | Singapore | IV | 4E | 4E4 |
| 3204 | 2003 | EU448408 | Indonesia | IV | 4E | 4E4 |
| 3205 | 2003 | FJ196841 | China | IV | 4E | 4E4 |
| 3206 | 2003 | FJ196842 | China | IV | 4E | 4E4 |
| 3207 | 2003 | JN415488 | Indonesia | IV | 4E | 4E4 |
| 3208 | 2003 | JN415514 | Australia | IV | 4E | 4E4 |
| 3209 | 2005 | EU069597 | Singapore | IV | 4E | 4E4 |
| 3210 | 2007 | EU448409 | Indonesia | IV | 4E | 4E4 |
| 3211 | 2007 | KT827366 | China | IV | 4E | 4E4 |
| 3212 | 2002 | AB111073 | Indonesia | IV | 4E | 4E5 |
| 3213 | 2002 | AB111075 | Indonesia | IV | 4E | 4E5 |
| 3214 | 2002 | AB232666 | Indonesia | IV | 4E | 4E5 |
| 3215 | 2005 | LC148024 | Indonesia | IV | 4E | 4E5 |
| 3216 | 2010 | KY275190 | East_Timor | IV | 4E | 4E6 |
| 3217 | 2011 | JN544410 | Singapore | IV | 4E | 4E6 |
| 3218 | 2013 | KY818134 | Indonesia | IV | 4E | 4E6 |
| 3219 | 2013 | KY818135 | Indonesia | IV | 4E | 4E6 |
| 3220 | 2008 | JF967793 | Indonesia | IV | 4E | 4E7 |
| 3221 | 2008 | JF967794 | Indonesia | IV | 4E | 4E7 |
| 3222 | 2009 | JF967854 | Indonesia | IV | 4E | 4E7 |
| 3223 | 2010 | HQ871946 | Australia | IV | 4E | 4E7 |
| 3224 | 2010 | JN415494 | Indonesia | IV | 4E | 4E7 |
| 3225 | 2010 | KM216668 | Indonesia | IV | 4E | 4E7 |
| 3226 | 2008 | JF967809 | Indonesia | IV | 4E | 4E8 |
| 3227 | 2008 | KC762630 | Indonesia | IV | 4E | 4E8 |
| 3228 | 2009 | AB550408 | Indonesia | IV | 4E | 4E8 |
| 3229 | 2009 | AB550409 | Indonesia | IV | 4E | 4E8 |
| 3230 | 2009 | AB550410 | Indonesia | IV | 4E | 4E8 |
| 3231 | 2009 | AB550411 | Indonesia | IV | 4E | 4E8 |
| 3232 | 2009 | AB550412 | Indonesia | IV | 4E | 4E8 |
| 3233 | 2009 | AB550413 | Indonesia | IV | 4E | 4E8 |
| 3234 | 2009 | AB550414 | Indonesia | IV | 4E | 4E8 |
| 3235 | 2009 | AB550415 | Indonesia | IV | 4E | 4E8 |
| 3236 | 2009 | AB550416 | Indonesia | IV | 4E | 4E8 |
| 3237 | 2009 | AB550417 | Indonesia | IV | 4E | 4E8 |
| 3238 | 2009 | AB550418 | Indonesia | IV | 4E | 4E8 |
| 3239 | 2009 | AB550419 | Indonesia | IV | 4E | 4E8 |
| 3240 | 2009 | AB550420 | Indonesia | IV | 4E | 4E8 |
| 3241 | 2009 | AB550421 | Indonesia | IV | 4E | 4E8 |
| 3242 | 2009 | AB550422 | Indonesia | IV | 4E | 4E8 |
| 3243 | 2009 | AB550423 | Indonesia | IV | 4E | 4E8 |
| 3244 | 2009 | AB550424 | Indonesia | IV | 4E | 4E8 |
| 3245 | 2009 | AB550425 | Indonesia | IV | 4E | 4E8 |
| 3246 | 2009 | AB550426 | Indonesia | IV | 4E | 4E8 |
| 3247 | 2009 | AB550427 | Indonesia | IV | 4E | 4E8 |
| 3248 | 2009 | AB550428 | Indonesia | IV | 4E | 4E8 |
| 3249 | 2009 | AB550429 | Indonesia | IV | 4E | 4E8 |
| 3250 | 2009 | AB597978 | Indonesia | IV | 4E | 4E8 |
| 3251 | 2009 | AB597979 | Indonesia | IV | 4E | 4E8 |
| 3252 | 2009 | AB597980 | Indonesia | IV | 4E | 4E8 |
| 3253 | 2010 | JF967902 | Indonesia | IV | 4E | 4E8 |
| 3254 | 2010 | JN415508 | Indonesia | IV | 4E | 4E8 |
| 3255 | 2011 | AB915374 | Indonesia | IV | 4E | 4E8 |
| 3256 | 2011 | AB915375 | Indonesia | IV | 4E | 4E8 |
| 3257 | 2011 | KM236498 | Indonesia | IV | 4E | 4E8 |
| 3258 | 2011 | KM236499 | Indonesia | IV | 4E | 4E8 |
| 3259 | 2011 | KM236500 | Indonesia | IV | 4E | 4E8 |
| 3260 | 2011 | KM236501 | Indonesia | IV | 4E | 4E8 |
| 3261 | 2012 | KF052648 | Indonesia | IV | 4E | 4E8 |
| 3262 | 2012 | KF052649 | Indonesia | IV | 4E | 4E8 |
| 3263 | 2012 | AB915371 | Indonesia | IV | 4E | 4E8 |
| 3264 | 2012 | AB915372 | Indonesia | IV | 4E | 4E8 |
| 3265 | 2012 | AB915373 | Indonesia | IV | 4E | 4E8 |
| 3266 | 1988 | AB600922 | Indonesia | IV | 4F | 4F1 |
| 3267 | 1988 | AB600923 | Indonesia | IV | 4F | 4F2 |
| 3268 | 1988 | AB600924 | Indonesia | IV | 4F | 4F2 |
| 3269 | 2003 | AB195673 | Seychelles | IV | 4F | 4F3 |
| 3270 | 2004 | DQ285549 | Reunion | IV | 4F | 4F3 |
| 3271 | 2004 | DQ285551 | Reunion | IV | 4F | 4F3 |
| 3272 | 2004 | DQ285552 | Reunion | IV | 4F | 4F3 |
| 3273 | 2004 | DQ285553 | Reunion | IV | 4F | 4F3 |
| 3274 | 2004 | DQ285557 | Seychelles | IV | 4F | 4F3 |
| 3275 | 2004 | DQ285558 | Reunion | IV | 4F | 4F3 |
| 3276 | 2004 | DQ285560 | Reunion | IV | 4F | 4F3 |
| 3277 | 2004 | DQ285561 | Seychelles | IV | 4F | 4F3 |
| 3278 | 2004 | EU282328 | Reunion | IV | 4F | 4F3 |
| 3279 | 2005 | EU448411 | Indonesia | IV | 4F | 4F3 |
| 3280 | 2005 | JN697056 | Malaysia | IV | 4F | 4F3 |
| 3281 | 2006 | EU448412 | Madagascar | IV | 4F | 4F3 |
| 3282 | 2008 | KT825073 | NA | IV | 4F | 4F3 |
| 3283 | 2009 | KX646375 | Indonesia | IV | 4F | 4F3 |
| 3284 | 1995 | DQ855297 | China | IV | 4F | 4F4 |
| 3285 | 1995 | EF032590 | China | IV | 4F | 4F4 |
| 3286 | 1995 | FJ196846 | China | IV | 4F | 4F4 |
| 3287 | 1995 | JQ317719 | China | IV | 4F | 4F4 |
| 3288 | 1995 | JQ317720 | China | IV | 4F | 4F4 |
| 3289 | 1995 | JQ317721 | China | IV | 4F | 4F4 |
| 3290 | 1995 | JQ317722 | China | IV | 4F | 4F4 |
| 3291 | 1995 | JQ317723 | China | IV | 4F | 4F4 |
| 3292 | 1995 | JQ317724 | China | IV | 4F | 4F4 |
| 3293 | 1995 | JQ317725 | China | IV | 4F | 4F4 |
| 3294 | 1995 | JQ317726 | China | IV | 4F | 4F4 |
| 3295 | 2004 | EU448410 | Malaysia | IV | 4F | 4F4 |
| 3296 | 2010 | JF967950 | Indonesia | IV | 4F | 4F5 |
| 3297 | 2010 | JF967951 | Indonesia | IV | 4F | 4F5 |
| 3298 | 2010 | JN415513 | Malaysia | IV | 4F | 4F5 |
| 3299 | 2010 | KM216682 | Indonesia | IV | 4F | 4F5 |
| 3300 | 2010 | JN415493 | Indonesia | IV | 4F | 4F5 |
| 3301 | 2011 | LC148028 | Indonesia | IV | 4F | 4F5 |
| 3302 | 2011 | KT824978 | Papua_New_Guinea | IV | 4G | 4G1 |
| 3303 | 2011 | KT825048 | Papua_New_Guinea | IV | 4G | 4G2 |
| 3304 | 2013 | KT825010 | Papua_New_Guinea | IV | 4G | 4G2 |
| 3305 | 2016 | KY495801 | Papua_New_Guinea | IV | 4G | 4G2 |
| 3306 | 2015 | KT825040 | Papua_New_Guinea | IV | 4G | 4G3 |
| 3307 | 2015 | KT825070 | Papua_New_Guinea | IV | 4G | 4G3 |
| 3308 | 2012 | KT824991 | Papua_New_Guinea | IV | 4G | 4G4 |
| 3309 | 2013 | KT825011 | Australia | IV | 4G | 4G4 |
| 3310 | 2013 | KT825057 | Papua_New_Guinea | IV | 4G | 4G4 |
| 3311 | 2013 | KT825009 | Papua_New_Guinea | IV | 4G | 4G4 |
| 3312 | 2015 | KY495800 | Papua_New_Guinea | IV | 4G | 4G4 |
| 3313 | 2002 | JN415503 | Fiji | IV | 4G | 4G5 |
| 3314 | 2002 | JN415522 | Solomon_Islands | IV | 4G | 4G5 |
| 3315 | 2002 | JQ655094 | Solomon_Islands | IV | 4G | 4G5 |
| 3316 | 2003 | JN415495 | Australia | IV | 4G | 4G5 |
| 3317 | 2003 | JN415518 | Papua_New_Guinea | IV | 4G | 4G5 |
| 3318 | 2003 | JX891658 | Marshall_Islands | IV | 4G | 4G5 |
| 3319 | 2004 | AB188830 | Micronesia | IV | 4G | 4G5 |
| 3320 | 2004 | AB188831 | Micronesia | IV | 4G | 4G5 |
| 3321 | 2004 | AB204803 | Micronesia | IV | 4G | 4G5 |
| 3322 | 2004 | LC148023 | Micronesia | IV | 4G | 4G5 |
| 3323 | 1998 | AB189120 | Indonesia | IV | 4H | 4H1 |
| 3324 | 2001 | AB111070 | French_Polynesia | IV | 4H | 4H1 |
| 3325 | 2001 | AY630407 | French_Polynesia | IV | 4H | 4H1 |
| 3326 | 2001 | DQ091258 | USA.Hawaii | IV | 4H | 4H1 |
| 3327 | 2001 | DQ091259 | USA.Hawaii | IV | 4H | 4H1 |
| 3328 | 2001 | DQ091260 | USA.Hawaii | IV | 4H | 4H1 |
| 3329 | 2001 | DQ091262 | USA.Hawaii | IV | 4H | 4H1 |
| 3330 | 2001 | DQ091263 | USA.Hawaii | IV | 4H | 4H1 |
| 3331 | 2001 | DQ091264 | USA.Hawaii | IV | 4H | 4H1 |
| 3332 | 2001 | DQ091265 | USA.Hawaii | IV | 4H | 4H1 |
| 3333 | 2001 | DQ091266 | USA.Hawaii | IV | 4H | 4H1 |
| 3334 | 2001 | DQ091267 | USA.Hawaii | IV | 4H | 4H1 |
| 3335 | 2001 | DQ091268 | USA.Hawaii | IV | 4H | 4H1 |
| 3336 | 2001 | DQ091269 | USA.Hawaii | IV | 4H | 4H1 |
| 3337 | 2001 | DQ091270 | USA.Hawaii | IV | 4H | 4H1 |
| 3338 | 2001 | DQ091271 | USA.Hawaii | IV | 4H | 4H1 |
| 3339 | 2001 | DQ091272 | USA.Hawaii | IV | 4H | 4H1 |
| 3340 | 2001 | DQ091273 | USA.Hawaii | IV | 4H | 4H1 |
| 3341 | 2001 | DQ672556 | French_Polynesia | IV | 4H | 4H1 |
| 3342 | 2001 | DQ672557 | French_Polynesia | IV | 4H | 4H1 |
| 3343 | 2001 | DQ672558 | French_Polynesia | IV | 4H | 4H1 |
| 3344 | 2001 | DQ672559 | French_Polynesia | IV | 4H | 4H1 |
| 3345 | 2001 | DQ672560 | USA.Hawaii | IV | 4H | 4H1 |
| 3346 | 2001 | DQ672561 | USA.Hawaii | IV | 4H | 4H1 |
| 3347 | 2001 | DQ672562 | USA.Hawaii | IV | 4H | 4H1 |
| 3348 | 2001 | DQ672563 | USA.Hawaii | IV | 4H | 4H1 |
| 3349 | 2001 | FJ898448 | French_Polynesia | IV | 4H | 4H1 |
| 3350 | 2001 | JQ655049 | New_Caledonia | IV | 4H | 4H1 |
| 3351 | 2002 | EU863650 | Easter_Island | IV | 4H | 4H1 |
| 3352 | 2002 | JN415497 | Cook_Islands | IV | 4H | 4H1 |
| 3353 | 2002 | JQ655050 | New_Caledonia | IV | 4H | 4H1 |
| 3354 | 2002 | JQ655051 | New_Caledonia | IV | 4H | 4H1 |
| 3355 | 2002 | JQ655052 | New_Caledonia | IV | 4H | 4H1 |
| 3356 | 2002 | JQ655053 | New_Caledonia | IV | 4H | 4H1 |
| 3357 | 2002 | JQ655054 | New_Caledonia | IV | 4H | 4H1 |
| 3358 | 2002 | JQ915077 | New_Caledonia | IV | 4H | 4H1 |
| 3359 | 2003 | JQ655055 | New_Caledonia | IV | 4H | 4H1 |
| 3360 | 2003 | JQ655056 | New_Caledonia | IV | 4H | 4H1 |
| 3361 | 2003 | JQ655057 | New_Caledonia | IV | 4H | 4H1 |
| 3362 | 2003 | JQ655058 | New_Caledonia | IV | 4H | 4H1 |
| 3363 | 2003 | JQ655059 | New_Caledonia | IV | 4H | 4H1 |
| 3364 | 2003 | JQ655060 | New_Caledonia | IV | 4H | 4H1 |
| 3365 | 2003 | JQ655061 | New_Caledonia | IV | 4H | 4H1 |
| 3366 | 2003 | JQ655097 | Wallis_and_Futuna | IV | 4H | 4H1 |
| 3367 | 2004 | FR666920 | Malaysia | IV | 4H | 4H1 |
| 3368 | 2004 | FR666921 | Malaysia | IV | 4H | 4H1 |
| 3369 | 2004 | JQ655062 | New_Caledonia | IV | 4H | 4H1 |
| 3370 | 2004 | JQ655063 | New_Caledonia | IV | 4H | 4H1 |
| 3371 | 2004 | JQ655064 | New_Caledonia | IV | 4H | 4H1 |
| 3372 | 2004 | JQ655065 | New_Caledonia | IV | 4H | 4H1 |
| 3373 | 2004 | JQ655066 | New_Caledonia | IV | 4H | 4H1 |
| 3374 | 2004 | JQ915078 | New_Caledonia | IV | 4H | 4H1 |
| 3375 | 2006 | JN415498 | Cook_Islands | IV | 4H | 4H1 |
| 3376 | 2006 | JN415504 | Fiji | IV | 4H | 4H1 |
| 3377 | 2006 | JN415505 | Fiji | IV | 4H | 4H1 |
| 3378 | 2007 | JQ654972 | French_Polynesia | IV | 4H | 4H1 |
| 3379 | 2007 | JQ654973 | French_Polynesia | IV | 4H | 4H1 |
| 3380 | 2007 | JQ654979 | French_Polynesia | IV | 4H | 4H1 |
| 3381 | 2007 | JQ654981 | French_Polynesia | IV | 4H | 4H1 |
| 3382 | 2007 | JQ654982 | French_Polynesia | IV | 4H | 4H1 |
| 3383 | 2007 | JQ654983 | French_Polynesia | IV | 4H | 4H1 |
| 3384 | 2007 | JQ654986 | French_Polynesia | IV | 4H | 4H1 |
| 3385 | 2007 | JQ654990 | French_Polynesia | IV | 4H | 4H1 |
| 3386 | 2007 | JQ654991 | French_Polynesia | IV | 4H | 4H1 |
| 3387 | 2007 | JQ654992 | French_Polynesia | IV | 4H | 4H1 |
| 3388 | 2007 | JQ654996 | French_Polynesia | IV | 4H | 4H1 |
| 3389 | 2007 | JQ654998 | French_Polynesia | IV | 4H | 4H1 |
| 3390 | 2007 | JQ655000 | French_Polynesia | IV | 4H | 4H1 |
| 3391 | 2007 | JQ655004 | French_Polynesia | IV | 4H | 4H1 |
| 3392 | 2007 | JQ655006 | French_Polynesia | IV | 4H | 4H1 |
| 3393 | 2007 | JQ655008 | French_Polynesia | IV | 4H | 4H1 |
| 3394 | 2007 | JQ655009 | French_Polynesia | IV | 4H | 4H1 |
| 3395 | 2007 | JQ655012 | French_Polynesia | IV | 4H | 4H1 |
| 3396 | 2007 | JQ655067 | New_Caledonia | IV | 4H | 4H1 |
| 3397 | 2007 | JQ655068 | New_Caledonia | IV | 4H | 4H1 |
| 3398 | 2007 | JQ915071 | French_Polynesia | IV | 4H | 4H1 |
| 3399 | 2007 | KT824964 | Cook_Islands | IV | 4H | 4H1 |
| 3400 | 2007 | JQ654974 | French_Polynesia | IV | 4H | 4H1 |
| 3401 | 2007 | JQ654975 | French_Polynesia | IV | 4H | 4H1 |
| 3402 | 2007 | JQ654976 | French_Polynesia | IV | 4H | 4H1 |
| 3403 | 2007 | JQ654977 | French_Polynesia | IV | 4H | 4H1 |
| 3404 | 2007 | JQ654978 | French_Polynesia | IV | 4H | 4H1 |
| 3405 | 2007 | JQ654980 | French_Polynesia | IV | 4H | 4H1 |
| 3406 | 2007 | JQ654984 | French_Polynesia | IV | 4H | 4H1 |
| 3407 | 2007 | JQ654987 | French_Polynesia | IV | 4H | 4H1 |
| 3408 | 2007 | JQ654988 | French_Polynesia | IV | 4H | 4H1 |
| 3409 | 2007 | JQ654989 | French_Polynesia | IV | 4H | 4H1 |
| 3410 | 2007 | JQ654994 | French_Polynesia | IV | 4H | 4H1 |
| 3411 | 2007 | JQ654995 | French_Polynesia | IV | 4H | 4H1 |
| 3412 | 2007 | JQ654997 | French_Polynesia | IV | 4H | 4H1 |
| 3413 | 2007 | JQ654999 | French_Polynesia | IV | 4H | 4H1 |
| 3414 | 2007 | JQ655001 | French_Polynesia | IV | 4H | 4H1 |
| 3415 | 2007 | JQ655002 | French_Polynesia | IV | 4H | 4H1 |
| 3416 | 2007 | JQ655003 | French_Polynesia | IV | 4H | 4H1 |
| 3417 | 2007 | JQ655005 | French_Polynesia | IV | 4H | 4H1 |
| 3418 | 2007 | JQ655010 | French_Polynesia | IV | 4H | 4H1 |
| 3419 | 2007 | JQ655011 | French_Polynesia | IV | 4H | 4H1 |
| 3420 | 2007 | JQ655013 | French_Polynesia | IV | 4H | 4H1 |
| 3421 | 2007 | JQ655015 | French_Polynesia | IV | 4H | 4H1 |
| 3422 | 2007 | JQ915072 | French_Polynesia | IV | 4H | 4H1 |
| 3423 | 2007 | JQ654985 | French_Polynesia | IV | 4H | 4H1 |
| 3424 | 2007 | JQ654993 | French_Polynesia | IV | 4H | 4H1 |
| 3425 | 2007 | JQ655007 | French_Polynesia | IV | 4H | 4H1 |
| 3426 | 2007 | JQ655014 | French_Polynesia | IV | 4H | 4H1 |
| 3427 | 2007 | JQ655016 | French_Polynesia | IV | 4H | 4H1 |
| 3428 | 2007 | JQ655017 | French_Polynesia | IV | 4H | 4H1 |
| 3429 | 2008 | JF967797 | Tonga | IV | 4H | 4H1 |
| 3430 | 2008 | JN415530 | Tonga | IV | 4H | 4H1 |
| 3431 | 2008 | JQ655021 | French_Polynesia | IV | 4H | 4H1 |
| 3432 | 2008 | JQ655029 | French_Polynesia | IV | 4H | 4H1 |
| 3433 | 2008 | JQ655031 | French_Polynesia | IV | 4H | 4H1 |
| 3434 | 2008 | JQ655035 | French_Polynesia | IV | 4H | 4H1 |
| 3435 | 2008 | JQ655037 | French_Polynesia | IV | 4H | 4H1 |
| 3436 | 2008 | JQ655039 | French_Polynesia | IV | 4H | 4H1 |
| 3437 | 2008 | JQ655069 | New_Caledonia | IV | 4H | 4H1 |
| 3438 | 2008 | JQ655070 | New_Caledonia | IV | 4H | 4H1 |
| 3439 | 2008 | JQ655071 | New_Caledonia | IV | 4H | 4H1 |
| 3440 | 2008 | JQ655072 | New_Caledonia | IV | 4H | 4H1 |
| 3441 | 2008 | JQ655073 | New_Caledonia | IV | 4H | 4H1 |
| 3442 | 2008 | JQ655074 | New_Caledonia | IV | 4H | 4H1 |
| 3443 | 2008 | JQ655075 | New_Caledonia | IV | 4H | 4H1 |
| 3444 | 2008 | JQ655076 | New_Caledonia | IV | 4H | 4H1 |
| 3445 | 2008 | JQ655019 | French_Polynesia | IV | 4H | 4H1 |
| 3446 | 2008 | JQ655022 | French_Polynesia | IV | 4H | 4H1 |
| 3447 | 2008 | JQ655023 | French_Polynesia | IV | 4H | 4H1 |
| 3448 | 2008 | JQ915073 | French_Polynesia | IV | 4H | 4H1 |
| 3449 | 2008 | JQ655018 | French_Polynesia | IV | 4H | 4H1 |
| 3450 | 2008 | JQ655020 | French_Polynesia | IV | 4H | 4H1 |
| 3451 | 2008 | JQ655025 | French_Polynesia | IV | 4H | 4H1 |
| 3452 | 2008 | JQ655027 | French_Polynesia | IV | 4H | 4H1 |
| 3453 | 2008 | JQ655028 | French_Polynesia | IV | 4H | 4H1 |
| 3454 | 2008 | JQ655034 | French_Polynesia | IV | 4H | 4H1 |
| 3455 | 2008 | JQ655036 | French_Polynesia | IV | 4H | 4H1 |
| 3456 | 2008 | JQ915074 | French_Polynesia | IV | 4H | 4H1 |
| 3457 | 2008 | KY926849 | French_Polynesia | IV | 4H | 4H1 |
| 3458 | 2008 | JQ655024 | French_Polynesia | IV | 4H | 4H1 |
| 3459 | 2008 | JQ655026 | French_Polynesia | IV | 4H | 4H1 |
| 3460 | 2008 | JQ655030 | French_Polynesia | IV | 4H | 4H1 |
| 3461 | 2008 | JQ655032 | French_Polynesia | IV | 4H | 4H1 |
| 3462 | 2008 | JQ655033 | French_Polynesia | IV | 4H | 4H1 |
| 3463 | 2008 | JQ655038 | French_Polynesia | IV | 4H | 4H1 |
| 3464 | 2009 | JQ655040 | French_Polynesia | IV | 4H | 4H1 |
| 3465 | 2009 | JQ655041 | French_Polynesia | IV | 4H | 4H1 |
| 3466 | 2009 | JQ655046 | French_Polynesia | IV | 4H | 4H1 |
| 3467 | 2009 | JQ655078 | New_Caledonia | IV | 4H | 4H1 |
| 3468 | 2009 | JQ655079 | New_Caledonia | IV | 4H | 4H1 |
| 3469 | 2009 | JQ655080 | New_Caledonia | IV | 4H | 4H1 |
| 3470 | 2009 | JQ655083 | New_Caledonia | IV | 4H | 4H1 |
| 3471 | 2009 | JQ655084 | New_Caledonia | IV | 4H | 4H1 |
| 3472 | 2009 | JQ915075 | French_Polynesia | IV | 4H | 4H1 |
| 3473 | 2009 | JQ655042 | French_Polynesia | IV | 4H | 4H1 |
| 3474 | 2009 | JQ655043 | French_Polynesia | IV | 4H | 4H1 |
| 3475 | 2009 | JQ655044 | French_Polynesia | IV | 4H | 4H1 |
| 3476 | 2009 | JQ655045 | French_Polynesia | IV | 4H | 4H1 |
| 3477 | 2009 | JQ655047 | French_Polynesia | IV | 4H | 4H1 |
| 3478 | 2009 | JQ655048 | French_Polynesia | IV | 4H | 4H1 |
| 3479 | 2009 | JQ915076 | French_Polynesia | IV | 4H | 4H1 |
| 3480 | 2009 | JQ655077 | New_Caledonia | IV | 4H | 4H1 |
| 3481 | 2009 | JQ655081 | New_Caledonia | IV | 4H | 4H1 |
| 3482 | 2009 | JQ655082 | New_Caledonia | IV | 4H | 4H1 |
| 3483 | 2009 | JQ915079 | New_Caledonia | IV | 4H | 4H1 |
| 3484 | 2010 | JQ655085 | New_Caledonia | IV | 4H | 4H1 |
| 3485 | 2010 | JQ655086 | New_Caledonia | IV | 4H | 4H1 |
| 3486 | 2010 | JQ655087 | New_Caledonia | IV | 4H | 4H1 |
| 3487 | 2010 | JQ655088 | New_Caledonia | IV | 4H | 4H1 |
| 3488 | 2010 | JQ655089 | New_Caledonia | IV | 4H | 4H1 |
| 3489 | 2010 | JQ655090 | New_Caledonia | IV | 4H | 4H1 |
| 3490 | 2010 | JQ655091 | New_Caledonia | IV | 4H | 4H1 |
| 3491 | 2010 | JQ655092 | New_Caledonia | IV | 4H | 4H1 |
| 3492 | 2010 | JQ655093 | New_Caledonia | IV | 4H | 4H1 |
| 3493 | 2010 | JQ655096 | Vanuatu | IV | 4H | 4H1 |
| 3494 | 2010 | JQ915080 | New_Caledonia | IV | 4H | 4H1 |
| 3495 | 2011 | JX298570 | Fiji | IV | 4H | 4H1 |
| 3496 | 2011 | JX298571 | Fiji | IV | 4H | 4H1 |
| 3497 | 2011 | KT824974 | Fiji | IV | 4H | 4H1 |
| 3498 | 2011 | KT825046 | Fiji | IV | 4H | 4H1 |
| 3499 | 2012 | JX298567 | Fiji | IV | 4H | 4H1 |
| 3500 | 2012 | JX298568 | Fiji | IV | 4H | 4H1 |
| 3501 | 2012 | JX298569 | Fiji | IV | 4H | 4H1 |
| 3502 | 2012 | JX298572 | Niue | IV | 4H | 4H1 |
| 3503 | 2012 | JX298573 | Niue | IV | 4H | 4H1 |
| 3504 | 2012 | JX298574 | Kiribati | IV | 4H | 4H1 |
| 3505 | 2012 | JX298575 | Kiribati | IV | 4H | 4H1 |
| 3506 | 2012 | KC316014 | New_Caledonia | IV | 4H | 4H1 |
| 3507 | 2012 | KC316015 | New_Caledonia | IV | 4H | 4H1 |
| 3508 | 2012 | KC316016 | New_Caledonia | IV | 4H | 4H1 |
| 3509 | 2012 | KC316017 | New_Caledonia | IV | 4H | 4H1 |
| 3510 | 2012 | KC316018 | New_Caledonia | IV | 4H | 4H1 |
| 3511 | 2012 | KT824986 | Fiji | IV | 4H | 4H1 |
| 3512 | 2012 | KT824987 | Kiribati | IV | 4H | 4H1 |
| 3513 | 2012 | KY495797 | Fiji | IV | 4H | 4H1 |
| 3514 | 2012 | KY495798 | Kiribati | IV | 4H | 4H1 |
| 3515 | 2012 | KY495799 | Niue | IV | 4H | 4H1 |
| 3516 | 2013 | KT825019 | Fiji | IV | 4H | 4H1 |
| 3517 | 2014 | KM279390 | Fiji | IV | 4H | 4H1 |
| 3518 | 2014 | KM279391 | Fiji | IV | 4H | 4H1 |
| 3519 | 1956 | KF289073 | India | V | 5A | 5A1 |
| 3520 | 2005 | JF297581 | India | V | 5A | 5A2 |
| 3521 | 2005 | JF297582 | India | V | 5A | 5A2 |
| 3522 | 2005 | JQ922548 | India | V | 5A | 5A2 |
| 3523 | 2014 | KT239346 | Pakistan | V | 5A | 5A2 |
| 3524 | 2014 | KT239347 | Pakistan | V | 5A | 5A2 |
| 3525 | 2011 | JN544409 | Singapore | V | 5A | 5A3 |
| 3526 | 1993 | AY762084 | Singapore | V | 5B | 5B1 |
| 3527 | 1993 | DQ285562 | Comoros | V | 5B | 5B2 |
| 3528 | 1994 | AM746218 | Saudi_Arabia | V | 5B | 5B2 |
| 3529 | 1994 | AM746219 | Saudi_Arabia | V | 5B | 5B2 |
| 3530 | 1994 | AM746220 | Saudi_Arabia | V | 5B | 5B2 |
| 3531 | 1982 | JF297580 | India | V | 5B | 5B3 |
| 3532 | 1982 | JQ922545 | India | V | 5B | 5B3 |
| 3533 | 2014 | KT239348 | Pakistan | V | 5B | 5B3 |
| 3534 | 1962 | JF297571 | India | V | 5B | 5B4 |
| 3535 | 1980 | AY732411 | Thailand | V | 5B | 5B4 |
| 3536 | 1980 | AY732429 | Thailand | V | 5B | 5B4 |
| 3537 | 1980 | AY732447 | Thailand | V | 5B | 5B4 |
| 3538 | 1980 | AY732474 | Thailand | V | 5B | 5B4 |
| 3539 | 1980 | AY732476 | Thailand | V | 5B | 5B4 |
| 3540 | 1983 | AY732379 | Thailand | V | 5B | 5B4 |
| 3541 | 2004 | EF654104 | NA | V | 5B | 5B5 |
| 3542 | 2004 | KP406801 | NA | V | 5B | 5B5 |
| 3543 | 2006 | FJ687475 | India | V | 5B | 5B5 |
| 3544 | 2007 | GQ357690 | Singapore | V | 5B | 5B5 |
| 3545 | 2007 | GQ357691 | Singapore | V | 5B | 5B5 |
| 3546 | 2008 | JF967814 | India | V | 5B | 5B5 |
| 3547 | 2010 | JF967935 | Malaysia | V | 5B | 5B5 |
| 3548 | 2013 | KJ806950 | Singapore | V | 5B | 5B5 |
| 3549 | 2015 | KY021900 | India | V | 5B | 5B5 |
| 3550 | 2014 | KU551904 | India | V | 5B | 5B6 |
| 3551 | 2014 | KU551905 | India | V | 5B | 5B6 |
| 3552 | 2010 | JF754980 | Nepal | V | 5B | 5B7 |
| 3553 | 2010 | JF754981 | Nepal | V | 5B | 5B7 |
| 3554 | 2010 | JF754982 | Nepal | V | 5B | 5B7 |
| 3555 | 2010 | JF754983 | Nepal | V | 5B | 5B7 |
| 3556 | 2010 | JF754984 | Nepal | V | 5B | 5B7 |
| 3557 | 2010 | JF754985 | Nepal | V | 5B | 5B7 |
| 3558 | 2010 | JF754986 | Nepal | V | 5B | 5B7 |
| 3559 | 2010 | JF754987 | Nepal | V | 5B | 5B7 |
| 3560 | 2010 | JF754988 | Nepal | V | 5B | 5B7 |
| 3561 | 2010 | JF754989 | Nepal | V | 5B | 5B7 |
| 3562 | 2010 | JF800928 | Nepal | V | 5B | 5B7 |
| 3563 | 2004 | JN415524 | Sri_Lanka | V | 5C | 5C1 |
| 3564 | 2008 | GQ357692 | Singapore | V | 5C | 5C1 |
| 3565 | 2008 | JN903579 | India | V | 5C | 5C1 |
| 3566 | 2008 | JN415507 | India | V | 5C | 5C1 |
| 3567 | 2009 | JN903580 | India | V | 5C | 5C1 |
| 3568 | 2009 | JN903581 | India | V | 5C | 5C1 |
| 3569 | 2009 | JF960211 | Singapore | V | 5C | 5C1 |
| 3570 | 2009 | HQ149732 | China | V | 5C | 5C1 |
| 3571 | 2009 | HQ149733 | China | V | 5C | 5C1 |
| 3572 | 2009 | JN036371 | Bangladesh | V | 5C | 5C1 |
| 3573 | 2009 | JQ917404 | India | V | 5C | 5C1 |
| 3574 | 2010 | JF967932 | India | V | 5C | 5C1 |
| 3575 | 2010 | JF967939 | India | V | 5C | 5C1 |
| 3576 | 2010 | JN029809 | China | V | 5C | 5C1 |
| 3577 | 2010 | JN415486 | India | V | 5C | 5C1 |
| 3578 | 2010 | JQ692085 | India | V | 5C | 5C1 |
| 3579 | 2010 | KC863940 | India | V | 5C | 5C1 |
| 3580 | 2010 | KM216667 | India | V | 5C | 5C1 |
| 3581 | 2010 | KT824969 | India | V | 5C | 5C1 |
| 3582 | 2011 | KF289072 | India | V | 5C | 5C1 |
| 3583 | 2011 | KT824975 | India | V | 5C | 5C1 |
| 3584 | 2011 | KU509255 | India | V | 5C | 5C1 |
| 3585 | 2011 | JN544400 | Singapore | V | 5C | 5C1 |
| 3586 | 2011 | JN544401 | Singapore | V | 5C | 5C1 |
| 3587 | 2011 | JN544402 | Singapore | V | 5C | 5C1 |
| 3588 | 2011 | JN544403 | Singapore | V | 5C | 5C1 |
| 3589 | 2011 | JN544404 | Singapore | V | 5C | 5C1 |
| 3590 | 2011 | JN544405 | Singapore | V | 5C | 5C1 |
| 3591 | 2011 | JN544406 | Singapore | V | 5C | 5C1 |
| 3592 | 2011 | JN544407 | Singapore | V | 5C | 5C1 |
| 3593 | 2011 | JN544408 | Singapore | V | 5C | 5C1 |
| 3594 | 2011 | KP792535 | Singapore | V | 5C | 5C1 |
| 3595 | 2011 | KR779783 | Singapore | V | 5C | 5C1 |
| 3596 | 2012 | KY581728 | India | V | 5C | 5C1 |
| 3597 | 2012 | KY581729 | India | V | 5C | 5C1 |
| 3598 | 2012 | KJ806936 | Singapore | V | 5C | 5C1 |
| 3599 | 2012 | KJ806937 | Singapore | V | 5C | 5C1 |
| 3600 | 2012 | KJ806938 | Singapore | V | 5C | 5C1 |
| 3601 | 2012 | KP685233 | Singapore | V | 5C | 5C1 |
| 3602 | 2012 | KX380796 | Singapore | V | 5C | 5C1 |
| 3603 | 2012 | KM403575 | Singapore | V | 5C | 5C1 |
| 3604 | 2012 | KU570099 | China | V | 5C | 5C1 |
| 3605 | 2012 | KX380799 | Singapore | V | 5C | 5C1 |
| 3606 | 2012 | KX380800 | Singapore | V | 5C | 5C1 |
| 3607 | 2012 | KX380801 | Singapore | V | 5C | 5C1 |
| 3608 | 2012 | KX380802 | Singapore | V | 5C | 5C1 |
| 3609 | 2013 | KJ415094 | Myanmar | V | 5C | 5C1 |
| 3610 | 2013 | KJ415095 | India | V | 5C | 5C1 |
| 3611 | 2013 | KJ470717 | China | V | 5C | 5C1 |
| 3612 | 2013 | KJ470730 | China | V | 5C | 5C1 |
| 3613 | 2013 | KJ545435 | China | V | 5C | 5C1 |
| 3614 | 2013 | KP849868 | Bhutan | V | 5C | 5C1 |
| 3615 | 2013 | KX056461 | China | V | 5C | 5C1 |
| 3616 | 2013 | KY038896 | China | V | 5C | 5C1 |
| 3617 | 2013 | KY581730 | India | V | 5C | 5C1 |
| 3618 | 2013 | KY581731 | India | V | 5C | 5C1 |
| 3619 | 2013 | KY581732 | India | V | 5C | 5C1 |
| 3620 | 2013 | KY581733 | India | V | 5C | 5C1 |
| 3621 | 2013 | KY581734 | India | V | 5C | 5C1 |
| 3622 | 2013 | KJ545479 | China | V | 5C | 5C1 |
| 3623 | 2013 | KJ806954 | Singapore | V | 5C | 5C1 |
| 3624 | 2013 | KJ806955 | Singapore | V | 5C | 5C1 |
| 3625 | 2013 | KJ806956 | Singapore | V | 5C | 5C1 |
| 3626 | 2013 | KJ806957 | Singapore | V | 5C | 5C1 |
| 3627 | 2013 | KJ806958 | Singapore | V | 5C | 5C1 |
| 3628 | 2013 | KM403576 | Singapore | V | 5C | 5C1 |
| 3629 | 2013 | KM403577 | Singapore | V | 5C | 5C1 |
| 3630 | 2013 | KM403578 | Singapore | V | 5C | 5C1 |
| 3631 | 2013 | KM403579 | Singapore | V | 5C | 5C1 |
| 3632 | 2013 | KM403580 | Singapore | V | 5C | 5C1 |
| 3633 | 2013 | KM403581 | Singapore | V | 5C | 5C1 |
| 3634 | 2013 | KM403582 | Singapore | V | 5C | 5C1 |
| 3635 | 2013 | KM403583 | Singapore | V | 5C | 5C1 |
| 3636 | 2013 | KM403584 | Singapore | V | 5C | 5C1 |
| 3637 | 2013 | KM403585 | Singapore | V | 5C | 5C1 |
| 3638 | 2013 | KM403586 | Singapore | V | 5C | 5C1 |
| 3639 | 2013 | KM403587 | Singapore | V | 5C | 5C1 |
| 3640 | 2013 | KM403588 | Singapore | V | 5C | 5C1 |
| 3641 | 2013 | KM403589 | Singapore | V | 5C | 5C1 |
| 3642 | 2013 | KM403590 | Singapore | V | 5C | 5C1 |
| 3643 | 2013 | KM403591 | Singapore | V | 5C | 5C1 |
| 3644 | 2013 | KM403592 | Singapore | V | 5C | 5C1 |
| 3645 | 2013 | KM403593 | Singapore | V | 5C | 5C1 |
| 3646 | 2013 | KM403594 | Singapore | V | 5C | 5C1 |
| 3647 | 2013 | KM403595 | Singapore | V | 5C | 5C1 |
| 3648 | 2013 | KM403596 | Singapore | V | 5C | 5C1 |
| 3649 | 2013 | KM403597 | Singapore | V | 5C | 5C1 |
| 3650 | 2013 | KM403598 | Singapore | V | 5C | 5C1 |
| 3651 | 2013 | KM403599 | Singapore | V | 5C | 5C1 |
| 3652 | 2013 | KM403600 | Singapore | V | 5C | 5C1 |
| 3653 | 2013 | KM403601 | Singapore | V | 5C | 5C1 |
| 3654 | 2013 | KM403602 | Singapore | V | 5C | 5C1 |
| 3655 | 2013 | KM403603 | Singapore | V | 5C | 5C1 |
| 3656 | 2013 | KM403604 | Singapore | V | 5C | 5C1 |
| 3657 | 2013 | KM403605 | Singapore | V | 5C | 5C1 |
| 3658 | 2013 | KM403606 | Singapore | V | 5C | 5C1 |
| 3659 | 2013 | KM403607 | Singapore | V | 5C | 5C1 |
| 3660 | 2013 | KM403608 | Singapore | V | 5C | 5C1 |
| 3661 | 2013 | KM403609 | Singapore | V | 5C | 5C1 |
| 3662 | 2013 | KM403610 | Singapore | V | 5C | 5C1 |
| 3663 | 2013 | KM403611 | Singapore | V | 5C | 5C1 |
| 3664 | 2013 | KM403612 | Singapore | V | 5C | 5C1 |
| 3665 | 2013 | KM403613 | Singapore | V | 5C | 5C1 |
| 3666 | 2013 | KM403614 | Singapore | V | 5C | 5C1 |
| 3667 | 2013 | KM403615 | Singapore | V | 5C | 5C1 |
| 3668 | 2013 | KM403616 | Singapore | V | 5C | 5C1 |
| 3669 | 2013 | KM403617 | Singapore | V | 5C | 5C1 |
| 3670 | 2013 | KM403618 | Singapore | V | 5C | 5C1 |
| 3671 | 2013 | KM403619 | Singapore | V | 5C | 5C1 |
| 3672 | 2013 | KM403620 | Singapore | V | 5C | 5C1 |
| 3673 | 2013 | KM403621 | Singapore | V | 5C | 5C1 |
| 3674 | 2013 | KM403622 | Singapore | V | 5C | 5C1 |
| 3675 | 2013 | KM403623 | Singapore | V | 5C | 5C1 |
| 3676 | 2013 | KM403624 | Singapore | V | 5C | 5C1 |
| 3677 | 2013 | KM403625 | Singapore | V | 5C | 5C1 |
| 3678 | 2013 | KM403626 | Singapore | V | 5C | 5C1 |
| 3679 | 2013 | KM403627 | Singapore | V | 5C | 5C1 |
| 3680 | 2013 | KM403628 | Singapore | V | 5C | 5C1 |
| 3681 | 2013 | KM403629 | Singapore | V | 5C | 5C1 |
| 3682 | 2013 | KM403630 | Singapore | V | 5C | 5C1 |
| 3683 | 2013 | KM403636 | Singapore | V | 5C | 5C1 |
| 3684 | 2013 | KP685234 | Singapore | V | 5C | 5C1 |
| 3685 | 2013 | KP849860 | Bhutan | V | 5C | 5C1 |
| 3686 | 2013 | KP849861 | Bhutan | V | 5C | 5C1 |
| 3687 | 2013 | KP849862 | Bhutan | V | 5C | 5C1 |
| 3688 | 2013 | KP849863 | Bhutan | V | 5C | 5C1 |
| 3689 | 2013 | KP849864 | Bhutan | V | 5C | 5C1 |
| 3690 | 2013 | KP849865 | Bhutan | V | 5C | 5C1 |
| 3691 | 2013 | KP849866 | Bhutan | V | 5C | 5C1 |
| 3692 | 2013 | KP849867 | Bhutan | V | 5C | 5C1 |
| 3693 | 2013 | KP849869 | Bhutan | V | 5C | 5C1 |
| 3694 | 2013 | KP849870 | Bhutan | V | 5C | 5C1 |
| 3695 | 2013 | KP849871 | Bhutan | V | 5C | 5C1 |
| 3696 | 2013 | KP849872 | Bhutan | V | 5C | 5C1 |
| 3697 | 2013 | KP849873 | Bhutan | V | 5C | 5C1 |
| 3698 | 2013 | KP849874 | Bhutan | V | 5C | 5C1 |
| 3699 | 2013 | KP849875 | Bhutan | V | 5C | 5C1 |
| 3700 | 2013 | KP849876 | Bhutan | V | 5C | 5C1 |
| 3701 | 2013 | KP849877 | Bhutan | V | 5C | 5C1 |
| 3702 | 2013 | KP849878 | Bhutan | V | 5C | 5C1 |
| 3703 | 2013 | KP849879 | Bhutan | V | 5C | 5C1 |
| 3704 | 2013 | KP849880 | Bhutan | V | 5C | 5C1 |
| 3705 | 2013 | KP849881 | Bhutan | V | 5C | 5C1 |
| 3706 | 2013 | KP849882 | Bhutan | V | 5C | 5C1 |
| 3707 | 2013 | KP849883 | Bhutan | V | 5C | 5C1 |
| 3708 | 2013 | KP849884 | Bhutan | V | 5C | 5C1 |
| 3709 | 2013 | KP849885 | Bhutan | V | 5C | 5C1 |
| 3710 | 2013 | KP849886 | Bhutan | V | 5C | 5C1 |
| 3711 | 2013 | KP849887 | Bhutan | V | 5C | 5C1 |
| 3712 | 2013 | KP849888 | Bhutan | V | 5C | 5C1 |
| 3713 | 2013 | KP849889 | Bhutan | V | 5C | 5C1 |
| 3714 | 2013 | KP849890 | Bhutan | V | 5C | 5C1 |
| 3715 | 2013 | KP849891 | Bhutan | V | 5C | 5C1 |
| 3716 | 2013 | KP849892 | Bhutan | V | 5C | 5C1 |
| 3717 | 2013 | KT825056 | Indonesia | V | 5C | 5C1 |
| 3718 | 2013 | KX380803 | Singapore | V | 5C | 5C1 |
| 3719 | 2013 | KX380804 | Singapore | V | 5C | 5C1 |
| 3720 | 2013 | KX380805 | Singapore | V | 5C | 5C1 |
| 3721 | 2013 | KX380806 | Singapore | V | 5C | 5C1 |
| 3722 | 2013 | KM403631 | Singapore | V | 5C | 5C1 |
| 3723 | 2013 | KM403632 | Singapore | V | 5C | 5C1 |
| 3724 | 2013 | KM403633 | Singapore | V | 5C | 5C1 |
| 3725 | 2013 | KM403634 | Singapore | V | 5C | 5C1 |
| 3726 | 2013 | KM403635 | Singapore | V | 5C | 5C1 |
| 3727 | 2013 | KX225487 | China | V | 5C | 5C1 |
| 3728 | 2014 | KT175087 | Malaysia | V | 5C | 5C1 |
| 3729 | 2014 | KT175110 | India | V | 5C | 5C1 |
| 3730 | 2014 | KT239344 | Pakistan | V | 5C | 5C1 |
| 3731 | 2014 | KT825026 | Maldives | V | 5C | 5C1 |
| 3732 | 2014 | KT825063 | Australia | V | 5C | 5C1 |
| 3733 | 2014 | KY495794 | Maldives | V | 5C | 5C1 |
| 3734 | 2014 | KY581735 | India | V | 5C | 5C1 |
| 3735 | 2014 | KJ806964 | Singapore | V | 5C | 5C1 |
| 3736 | 2014 | KJ806965 | Singapore | V | 5C | 5C1 |
| 3737 | 2014 | KJ806966 | Singapore | V | 5C | 5C1 |
| 3738 | 2014 | KJ806968 | Singapore | V | 5C | 5C1 |
| 3739 | 2014 | KR024708 | China | V | 5C | 5C1 |
| 3740 | 2014 | KT175082 | Malaysia | V | 5C | 5C1 |
| 3741 | 2014 | KT175089 | Malaysia | V | 5C | 5C1 |
| 3742 | 2014 | KT175108 | Singapore | V | 5C | 5C1 |
| 3743 | 2014 | KT175109 | Singapore | V | 5C | 5C1 |
| 3744 | 2014 | KT239343 | Pakistan | V | 5C | 5C1 |
| 3745 | 2014 | KT453242 | China | V | 5C | 5C1 |
| 3746 | 2014 | KT453243 | China | V | 5C | 5C1 |
| 3747 | 2014 | KT453244 | China | V | 5C | 5C1 |
| 3748 | 2014 | KT825065 | Singapore | V | 5C | 5C1 |
| 3749 | 2014 | KU570103 | China | V | 5C | 5C1 |
| 3750 | 2014 | KU570104 | China | V | 5C | 5C1 |
| 3751 | 2014 | KU570105 | China | V | 5C | 5C1 |
| 3752 | 2014 | KU570106 | China | V | 5C | 5C1 |
| 3753 | 2014 | KU570107 | China | V | 5C | 5C1 |
| 3754 | 2014 | KU570108 | China | V | 5C | 5C1 |
| 3755 | 2014 | KU570109 | China | V | 5C | 5C1 |
| 3756 | 2014 | KU570110 | China | V | 5C | 5C1 |
| 3757 | 2014 | KU570111 | China | V | 5C | 5C1 |
| 3758 | 2014 | KU570112 | China | V | 5C | 5C1 |
| 3759 | 2014 | KU570113 | China | V | 5C | 5C1 |
| 3760 | 2014 | KU570114 | China | V | 5C | 5C1 |
| 3761 | 2014 | KU666943 | Malaysia | V | 5C | 5C1 |
| 3762 | 2014 | LC038145 | Malaysia | V | 5C | 5C1 |
| 3763 | 2014 | LC038147 | Indonesia | V | 5C | 5C1 |
| 3764 | 2014 | KP191494 | China | V | 5C | 5C1 |
| 3765 | 2014 | KP191495 | China | V | 5C | 5C1 |
| 3766 | 2014 | KP191496 | China | V | 5C | 5C1 |
| 3767 | 2014 | KP191497 | China | V | 5C | 5C1 |
| 3768 | 2014 | KP191498 | China | V | 5C | 5C1 |
| 3769 | 2014 | KP191502 | China | V | 5C | 5C1 |
| 3770 | 2014 | KP191505 | China | V | 5C | 5C1 |
| 3771 | 2014 | KP191506 | China | V | 5C | 5C1 |
| 3772 | 2014 | KP191507 | China | V | 5C | 5C1 |
| 3773 | 2014 | KP191509 | China | V | 5C | 5C1 |
| 3774 | 2014 | KP191510 | China | V | 5C | 5C1 |
| 3775 | 2014 | KP191513 | China | V | 5C | 5C1 |
| 3776 | 2014 | KP191514 | China | V | 5C | 5C1 |
| 3777 | 2014 | KP191515 | China | V | 5C | 5C1 |
| 3778 | 2014 | KP191517 | China | V | 5C | 5C1 |
| 3779 | 2014 | KP191520 | China | V | 5C | 5C1 |
| 3780 | 2014 | KP686070 | China | V | 5C | 5C1 |
| 3781 | 2014 | KP723473 | China | V | 5C | 5C1 |
| 3782 | 2014 | KP723475 | China | V | 5C | 5C1 |
| 3783 | 2014 | KP723477 | China | V | 5C | 5C1 |
| 3784 | 2014 | KR024705 | China | V | 5C | 5C1 |
| 3785 | 2014 | KR024706 | China | V | 5C | 5C1 |
| 3786 | 2014 | KR024707 | China | V | 5C | 5C1 |
| 3787 | 2014 | KT037104 | China | V | 5C | 5C1 |
| 3788 | 2014 | KT037110 | China | V | 5C | 5C1 |
| 3789 | 2014 | KT037111 | China | V | 5C | 5C1 |
| 3790 | 2014 | KT037113 | China | V | 5C | 5C1 |
| 3791 | 2014 | KT037114 | China | V | 5C | 5C1 |
| 3792 | 2014 | KT037115 | China | V | 5C | 5C1 |
| 3793 | 2014 | KT175100 | China | V | 5C | 5C1 |
| 3794 | 2014 | KT187559 | China | V | 5C | 5C1 |
| 3795 | 2014 | KT187560 | China | V | 5C | 5C1 |
| 3796 | 2014 | KT187561 | China | V | 5C | 5C1 |
| 3797 | 2014 | KT187562 | China | V | 5C | 5C1 |
| 3798 | 2014 | KT187563 | China | V | 5C | 5C1 |
| 3799 | 2014 | KT232195 | China | V | 5C | 5C1 |
| 3800 | 2014 | KT232196 | China | V | 5C | 5C1 |
| 3801 | 2014 | KT232197 | China | V | 5C | 5C1 |
| 3802 | 2014 | KT232198 | China | V | 5C | 5C1 |
| 3803 | 2014 | KT232199 | China | V | 5C | 5C1 |
| 3804 | 2014 | KT232200 | China | V | 5C | 5C1 |
| 3805 | 2014 | KT232201 | China | V | 5C | 5C1 |
| 3806 | 2014 | KT232202 | China | V | 5C | 5C1 |
| 3807 | 2014 | KT232203 | China | V | 5C | 5C1 |
| 3808 | 2014 | KT382296 | China | V | 5C | 5C1 |
| 3809 | 2014 | KT382299 | China | V | 5C | 5C1 |
| 3810 | 2014 | KT382300 | China | V | 5C | 5C1 |
| 3811 | 2014 | KT382301 | China | V | 5C | 5C1 |
| 3812 | 2014 | KT382302 | China | V | 5C | 5C1 |
| 3813 | 2014 | KT382303 | China | V | 5C | 5C1 |
| 3814 | 2014 | KT382305 | China | V | 5C | 5C1 |
| 3815 | 2014 | KT428605 | China | V | 5C | 5C1 |
| 3816 | 2014 | KT428606 | China | V | 5C | 5C1 |
| 3817 | 2014 | KT428607 | China | V | 5C | 5C1 |
| 3818 | 2014 | KT428608 | China | V | 5C | 5C1 |
| 3819 | 2014 | KT428610 | China | V | 5C | 5C1 |
| 3820 | 2014 | KT428611 | China | V | 5C | 5C1 |
| 3821 | 2014 | KT428612 | China | V | 5C | 5C1 |
| 3822 | 2014 | KT428613 | China | V | 5C | 5C1 |
| 3823 | 2014 | KT428614 | China | V | 5C | 5C1 |
| 3824 | 2014 | KT428615 | China | V | 5C | 5C1 |
| 3825 | 2014 | KT453230 | China | V | 5C | 5C1 |
| 3826 | 2014 | KT453231 | China | V | 5C | 5C1 |
| 3827 | 2014 | KT453232 | China | V | 5C | 5C1 |
| 3828 | 2014 | KT453233 | China | V | 5C | 5C1 |
| 3829 | 2014 | KT453234 | China | V | 5C | 5C1 |
| 3830 | 2014 | KT453235 | China | V | 5C | 5C1 |
| 3831 | 2014 | KT453236 | China | V | 5C | 5C1 |
| 3832 | 2014 | KT453237 | China | V | 5C | 5C1 |
| 3833 | 2014 | KT453238 | China | V | 5C | 5C1 |
| 3834 | 2014 | KT453239 | China | V | 5C | 5C1 |
| 3835 | 2014 | KT453240 | China | V | 5C | 5C1 |
| 3836 | 2014 | KT453241 | China | V | 5C | 5C1 |
| 3837 | 2014 | KT819304 | China | V | 5C | 5C1 |
| 3838 | 2014 | KT827374 | China | V | 5C | 5C1 |
| 3839 | 2014 | KT827375 | China | V | 5C | 5C1 |
| 3840 | 2014 | KT827377 | China | V | 5C | 5C1 |
| 3841 | 2014 | KU570118 | China | V | 5C | 5C1 |
| 3842 | 2014 | KU672336 | China | V | 5C | 5C1 |
| 3843 | 2014 | KU672337 | China | V | 5C | 5C1 |
| 3844 | 2014 | KU672338 | China | V | 5C | 5C1 |
| 3845 | 2014 | KU672339 | China | V | 5C | 5C1 |
| 3846 | 2014 | KU672340 | China | V | 5C | 5C1 |
| 3847 | 2014 | KU672341 | China | V | 5C | 5C1 |
| 3848 | 2014 | KX225483 | China | V | 5C | 5C1 |
| 3849 | 2014 | KX225484 | China | V | 5C | 5C1 |
| 3850 | 2014 | KX459386 | China | V | 5C | 5C1 |
| 3851 | 2014 | KX459387 | China | V | 5C | 5C1 |
| 3852 | 2014 | KX459388 | China | V | 5C | 5C1 |
| 3853 | 2014 | KX459389 | China | V | 5C | 5C1 |
| 3854 | 2014 | KX459390 | China | V | 5C | 5C1 |
| 3855 | 2014 | KX459391 | China | V | 5C | 5C1 |
| 3856 | 2014 | KX459392 | China | V | 5C | 5C1 |
| 3857 | 2014 | KX620451 | China | V | 5C | 5C1 |
| 3858 | 2014 | KX620452 | China | V | 5C | 5C1 |
| 3859 | 2014 | KX620453 | China | V | 5C | 5C1 |
| 3860 | 2014 | KX621249 | China | V | 5C | 5C1 |
| 3861 | 2015 | KX721476 | India | V | 5C | 5C1 |
| 3862 | 2015 | KX721477 | India | V | 5C | 5C1 |
| 3863 | 2015 | KX721478 | India | V | 5C | 5C1 |
| 3864 | 2015 | KX721479 | India | V | 5C | 5C1 |
| 3865 | 2015 | KX721480 | India | V | 5C | 5C1 |
| 3866 | 2015 | KX721481 | India | V | 5C | 5C1 |
| 3867 | 2015 | KX721482 | India | V | 5C | 5C1 |
| 3868 | 2015 | KT825068 | India | V | 5C | 5C1 |
| 3869 | 2015 | KY021897 | India | V | 5C | 5C1 |
| 3870 | 2015 | KY021898 | India | V | 5C | 5C1 |
| 3871 | 2015 | KY021899 | India | V | 5C | 5C1 |
| 3872 | 2015 | KY978440 | India | V | 5C | 5C1 |
| 3873 | 2015 | KT825042 | Thailand | V | 5C | 5C1 |
| 3874 | 2015 | KT825067 | Australia | V | 5C | 5C1 |
| 3875 | 2015 | KY921903 | Singapore | V | 5C | 5C1 |
| 3876 | 2015 | KT827378 | China | V | 5C | 5C1 |
| 3877 | 2016 | KY495795 | Maldives | V | 5C | 5C1 |
| 3878 | 2016 | GZ-18488 | China | V | 5C | 5C1 |
| 3879 | 2016 | KX372686 | China | V | 5C | 5C1 |
| 3880 | 1971 | AY713473 | Myanmar | V | 5D | 5D1 |
| 3881 | 1976 | AF425615 | Myanmar | V | 5D | 5D1 |
| 3882 | 1976 | AY722801 | Myanmar | V | 5D | 5D1 |
| 3883 | 1996 | AY589692 | Myanmar | V | 5D | 5D2 |
| 3884 | 1996 | AY722802 | Myanmar | V | 5D | 5D2 |
| 3885 | 1998 | AY600860 | Myanmar | V | 5D | 5D2 |
| 3886 | 1998 | AY722803 | Myanmar | V | 5D | 5D2 |
| 3887 | 1968 | AF425625 | Nigeria | V | 5E | 5E1 |
| 3888 | 1985 | AF425620 | Cote_dIvoire | V | 5F | 5F1 |
| 3889 | 1999 | AF298807 | Cote_dIvoire | V | 5G | 5G1 |
| 3890 | 1970 | JF297578 | India | V | 5H | 5H1 |
| 3891 | 2014 | KT239350 | Pakistan | V | 5H | 5H1 |
| 3892 | 1971 | JF297579 | India | V | 5H | 5H2 |
| 3893 | 1971 | JQ922546 | India | V | 5H | 5H2 |
| 3894 | 2014 | KT239349 | Pakistan | V | 5H | 5H2 |
| 3895 | 2013 | KF864667 | China | V | 5I | 5I1 |
| 3896 | 2013 | KM277610 | Angola | V | 5I | 5I1 |
| 3897 | 2013 | KM277611 | Angola | V | 5I | 5I1 |
| 3898 | 2013 | KM277612 | Angola | V | 5I | 5I1 |
| 3899 | 2013 | KM277613 | Angola | V | 5I | 5I1 |
| 3900 | 2013 | KM277614 | Angola | V | 5I | 5I1 |
| 3901 | 2013 | KU570096 | China | V | 5I | 5I1 |
| 3902 | 2013 | KU570097 | China | V | 5I | 5I1 |
| 3903 | 2013 | KU570098 | China | V | 5I | 5I1 |
| 3904 | 1977 | D00501 | Jamaica | V | 5J | 5J1 |
| 3905 | 1983 | D00504 | Mexico | V | 5J | 5J2 |
| 3906 | 1985 | D00505 | Aruba | V | 5J | 5J2 |
| 3907 | 1962 | JF297572 | India | V | 5K | 5K1 |
| 3908 | 1962 | JF297573 | India | V | 5K | 5K2 |
| 3909 | 1963 | JF297574 | India | V | 5K | 5K3 |
| 3910 | 1963 | JF297575 | India | V | 5K | 5K4 |
| 3911 | 1963 | JF297576 | India | V | 5K | 5K5 |
| 3912 | 2014 | KT239352 | Pakistan | V | 5K | 5K5 |
| 3913 | 1963 | JF297577 | India | V | 5K | 5K6 |
| 3914 | 1963 | JQ922544 | India | V | 5K | 5K6 |
| 3915 | 2014 | KT239351 | Pakistan | V | 5K | 5K6 |
| 3916 | 2004 | DQ285554 | Reunion | V | 5K | 5K7 |
| 3917 | 2004 | DQ285559 | Reunion | V | 5K | 5K7 |
| 3918 | 2005 | EU081258 | Singapore | V | 5K | 5K8 |
| 3919 | 2006 | EU448413 | India | V | 5K | 5K9 |
| 3920 | 2007 | JN903578 | India | V | 5K | 5K10 |
| 3921 | 2014 | KT239345 | Pakistan | V | 5K | 5K11 |
| 3922 | 1977 | AF425621 | Jamaica | V | 5K | 5K12 |
| 3923 | 1977 | AF425618 | Grenada | V | 5K | 5K12 |
| 3924 | 1977 | JN379475 | Bahamas | V | 5K | 5K12 |
| 3925 | 1977 | JN379476 | Grenada | V | 5K | 5K12 |
| 3926 | 1977 | JN379477 | Grenada | V | 5K | 5K12 |
| 3927 | 1977 | JN379478 | Grenada | V | 5K | 5K12 |
| 3928 | 1977 | JN379479 | Grenada | V | 5K | 5K12 |
| 3929 | 1977 | JN379480 | Grenada | V | 5K | 5K12 |
| 3930 | 1978 | JN379481 | Grenada | V | 5K | 5K12 |
| 3931 | 1978 | JN379482 | Grenada | V | 5K | 5K12 |
| 3932 | 1981 | JN379484 | Grenada | V | 5K | 5K12 |
| 3933 | 1981 | JN379483 | Trinidad_and_Tobago | V | 5K | 5K12 |
| 3934 | 1981 | JN379485 | Suriname | V | 5K | 5K12 |
| 3935 | 1982 | AF425613 | Brazil | V | 5K | 5K12 |
| 3936 | 1985 | AF425616 | Colombia | V | 5K | 5K12 |
| 3937 | 1986 | AF425639 | Trinidad_and_Tobago | V | 5K | 5K12 |
| 3938 | 1996 | AF425617 | Colombia | V | 5K | 5K12 |
| 3939 | 2002 | HM450094 | Brazil | V | 5K | 5K12 |
| 3940 | 2004 | JN379473 | Aruba | V | 5K | 5K12 |
| 3941 | 1982 | DQ341188 | Mexico | V | 5K | 5K13 |
| 3942 | 1983 | AF425624 | Mexico | V | 5K | 5K13 |
| 3943 | 1984 | DQ341189 | Mexico | V | 5K | 5K13 |
| 3944 | 1986 | DQ341191 | Mexico | V | 5K | 5K14 |
| 3945 | 1990 | AF425623 | Mexico | V | 5K | 5K15 |
| 3946 | 1984 | DQ341190 | Mexico | V | 5K | 5K16 |
| 3947 | 1978 | AF425631 | Trinidad_and_Tobago | V | 5K | 5K17 |
| 3948 | 1988 | AF425610 | Angola | V | 5K | 5K18 |
| 3949 | 1993 | JN819417 | El_Salvador | V | 5K | 5K19 |
| 3950 | 1993 | AY153755 | Costa_Rica | V | 5K | 5K20 |
| 3951 | 1996 | FJ410188 | Puerto_Rico | V | 5K | 5K20 |
| 3952 | 1996 | FJ478457 | Puerto_Rico | V | 5K | 5K20 |
| 3953 | 1994 | DQ341192 | Mexico | V | 5K | 5K21 |
| 3954 | 1995 | DQ341193 | Mexico | V | 5K | 5K21 |
| 3955 | 1995 | DQ341194 | Mexico | V | 5K | 5K21 |
| 3956 | 1985 | AF425609 | Aruba | V | 5K | 5K22 |
| 3957 | 1986 | FJ562106 | Puerto_Rico | V | 5K | 5K22 |
| 3958 | 1992 | FJ410186 | Puerto_Rico | V | 5K | 5K22 |
| 3959 | 1992 | FJ547087 | Puerto_Rico | V | 5K | 5K22 |
| 3960 | 1995 | FJ547086 | Puerto_Rico | V | 5K | 5K22 |
| 3961 | 1989 | AF226687 | French_Guiana | V | 5K | 5K23 |
| 3962 | 1989 | AY630408 | French_Polynesia | V | 5K | 5K23 |
| 3963 | 1993 | FJ562105 | Puerto_Rico | V | 5K | 5K23 |
| 3964 | 1995 | FJ205874 | USA | V | 5K | 5K23 |
| 3965 | 1985 | GQ868601 | Virgin_Islands | V | 5L | 5L1 |
| 3966 | 2000 | FJ850070 | Brazil | V | 5L | 5L1 |
| 3967 | 2000 | FJ850071 | Brazil | V | 5L | 5L1 |
| 3968 | 2001 | HM450088 | Brazil | V | 5L | 5L1 |
| 3969 | 2001 | HM450089 | Brazil | V | 5L | 5L1 |
| 3970 | 2001 | KF672789 | Brazil | V | 5L | 5L1 |
| 3971 | 2002 | FJ850075 | Brazil | V | 5L | 5L1 |
| 3972 | 2002 | HM450092 | Brazil | V | 5L | 5L1 |
| 3973 | 2002 | HM450093 | Brazil | V | 5L | 5L1 |
| 3974 | 2002 | HM450095 | Brazil | V | 5L | 5L1 |
| 3975 | 2002 | HM450105 | Brazil | V | 5L | 5L1 |
| 3976 | 2003 | FJ850077 | Brazil | V | 5L | 5L1 |
| 3977 | 2003 | HM450096 | Brazil | V | 5L | 5L1 |
| 3978 | 2004 | FJ850081 | Brazil | V | 5L | 5L1 |
| 3979 | 2004 | HM450097 | Brazil | V | 5L | 5L1 |
| 3980 | 2005 | FJ850084 | Brazil | V | 5L | 5L1 |
| 3981 | 2005 | HM450099 | Brazil | V | 5L | 5L1 |
| 3982 | 2007 | FJ850090 | Brazil | V | 5L | 5L1 |
| 3983 | 2013 | KP188545 | Brazil | V | 5L | 5L2 |
| 3984 | 2013 | KP903774 | Brazil | V | 5L | 5L2 |
| 3985 | 2013 | KP903781 | Brazil | V | 5L | 5L2 |
| 3986 | 2014 | KP188568 | Brazil | V | 5L | 5L2 |
| 3987 | 2014 | KU509311 | Brazil | V | 5L | 5L2 |
| 3988 | 2015 | KT825041 | NA | V | 5L | 5L2 |
| 3989 | 2016 | KX768339 | Argentina | V | 5L | 5L2 |
| 3990 | 2016 | KX768340 | Argentina | V | 5L | 5L2 |
| 3991 | 2016 | KX768341 | Argentina | V | 5L | 5L2 |
| 3992 | 2016 | KX768342 | Argentina | V | 5L | 5L2 |
| 3993 | 2016 | KX768343 | Argentina | V | 5L | 5L2 |
| 3994 | 2016 | KX768344 | Argentina | V | 5L | 5L2 |
| 3995 | 2016 | KX768345 | Argentina | V | 5L | 5L2 |
| 3996 | 2016 | KX768346 | Argentina | V | 5L | 5L2 |
| 3997 | 2016 | KX768347 | Argentina | V | 5L | 5L2 |
| 3998 | 2016 | KX768348 | Argentina | V | 5L | 5L2 |
| 3999 | 2016 | KX768349 | Argentina | V | 5L | 5L2 |
| 4000 | 2016 | KX768350 | Argentina | V | 5L | 5L2 |
| 4001 | 2016 | KX768351 | Argentina | V | 5L | 5L2 |
| 4002 | 2016 | KX768352 | Argentina | V | 5L | 5L2 |
| 4003 | 2016 | KX768353 | Argentina | V | 5L | 5L2 |
| 4004 | 2016 | KX768354 | Argentina | V | 5L | 5L2 |
| 4005 | 2016 | KX768355 | Argentina | V | 5L | 5L2 |
| 4006 | 2016 | KX768356 | Argentina | V | 5L | 5L2 |
| 4007 | 2016 | KX768357 | Argentina | V | 5L | 5L2 |
| 4008 | 2016 | KX768358 | Argentina | V | 5L | 5L2 |
| 4009 | 2016 | KX768359 | Argentina | V | 5L | 5L2 |
| 4010 | 2016 | KX768360 | Argentina | V | 5L | 5L2 |
| 4011 | 2016 | KX768361 | Argentina | V | 5L | 5L2 |
| 4012 | 2016 | KX768362 | Argentina | V | 5L | 5L2 |
| 4013 | 2016 | KY283848 | Argentina | V | 5L | 5L2 |
| 4014 | 2016 | KY283850 | Argentina | V | 5L | 5L2 |
| 4015 | 2016 | KY283851 | Argentina | V | 5L | 5L2 |
| 4016 | 2016 | KY283852 | Argentina | V | 5L | 5L2 |
| 4017 | 2006 | FJ850087 | Brazil | V | 5L | 5L3 |
| 4018 | 2006 | HM450100 | Brazil | V | 5L | 5L3 |
| 4019 | 2007 | HM450077 | Brazil | V | 5L | 5L3 |
| 4020 | 2007 | HM450078 | Brazil | V | 5L | 5L3 |
| 4021 | 2007 | HM450101 | Brazil | V | 5L | 5L3 |
| 4022 | 2008 | GU131863 | Brazil | V | 5L | 5L3 |
| 4023 | 2009 | HM043709 | Brazil | V | 5L | 5L3 |
| 4024 | 2009 | HM043710 | Brazil | V | 5L | 5L3 |
| 4025 | 2009 | HQ026761 | Brazil | V | 5L | 5L3 |
| 4026 | 2010 | HQ026762 | Brazil | V | 5L | 5L3 |
| 4027 | 2010 | HQ696613 | Brazil | V | 5L | 5L3 |
| 4028 | 2010 | HQ696614 | Brazil | V | 5L | 5L3 |
| 4029 | 2010 | JN982362 | Brazil | V | 5L | 5L3 |
| 4030 | 2010 | JX669461 | Brazil | V | 5L | 5L3 |
| 4031 | 2010 | JX669464 | Brazil | V | 5L | 5L3 |
| 4032 | 2010 | JX669465 | Brazil | V | 5L | 5L3 |
| 4033 | 2010 | KF672759 | Brazil | V | 5L | 5L3 |
| 4034 | 2010 | KF672785 | Brazil | V | 5L | 5L3 |
| 4035 | 2010 | KF719187 | Brazil | V | 5L | 5L3 |
| 4036 | 2010 | KP188539 | Brazil | V | 5L | 5L3 |
| 4037 | 2010 | KC692511 | Argentina | V | 5L | 5L3 |
| 4038 | 2010 | KC692513 | Argentina | V | 5L | 5L3 |
| 4039 | 2011 | KF672767 | Brazil | V | 5L | 5L3 |
| 4040 | 2011 | KF672784 | Brazil | V | 5L | 5L3 |
| 4041 | 2012 | KP188543 | Brazil | V | 5L | 5L3 |
| 4042 | 2012 | KP188544 | Brazil | V | 5L | 5L3 |
| 4043 | 2012 | KY818240 | Brazil | V | 5L | 5L3 |
| 4044 | 2012 | KY818241 | Brazil | V | 5L | 5L3 |
| 4045 | 2012 | KY818237 | Brazil | V | 5L | 5L3 |
| 4046 | 2013 | KP188546 | Brazil | V | 5L | 5L3 |
| 4047 | 2013 | KP188547 | Brazil | V | 5L | 5L3 |
| 4048 | 2013 | KP188548 | Brazil | V | 5L | 5L3 |
| 4049 | 2013 | KP188567 | Brazil | V | 5L | 5L3 |
| 4050 | 2013 | KP858105 | Brazil | V | 5L | 5L3 |
| 4051 | 2013 | KP858106 | Brazil | V | 5L | 5L3 |
| 4052 | 2013 | KP858108 | Brazil | V | 5L | 5L3 |
| 4053 | 2013 | KP858109 | Brazil | V | 5L | 5L3 |
| 4054 | 2013 | KP858110 | Brazil | V | 5L | 5L3 |
| 4055 | 2013 | KP858111 | Brazil | V | 5L | 5L3 |
| 4056 | 2013 | KP858112 | Brazil | V | 5L | 5L3 |
| 4057 | 2013 | KP858113 | Brazil | V | 5L | 5L3 |
| 4058 | 2013 | KP858115 | Brazil | V | 5L | 5L3 |
| 4059 | 2013 | KP858116 | Brazil | V | 5L | 5L3 |
| 4060 | 2013 | KP858117 | Brazil | V | 5L | 5L3 |
| 4061 | 2013 | KP858118 | Brazil | V | 5L | 5L3 |
| 4062 | 2013 | KP903770 | Brazil | V | 5L | 5L3 |
| 4063 | 2013 | KP903775 | Brazil | V | 5L | 5L3 |
| 4064 | 2013 | KP903776 | Brazil | V | 5L | 5L3 |
| 4065 | 2013 | KP903777 | Brazil | V | 5L | 5L3 |
| 4066 | 2013 | KP903778 | Brazil | V | 5L | 5L3 |
| 4067 | 2013 | KP903779 | Brazil | V | 5L | 5L3 |
| 4068 | 2013 | KY818238 | Brazil | V | 5L | 5L3 |
| 4069 | 2013 | KY818242 | Brazil | V | 5L | 5L3 |
| 4070 | 2013 | KY818289 | Brazil | V | 5L | 5L3 |
| 4071 | 2013 | KP858114 | Brazil | V | 5L | 5L3 |
| 4072 | 2013 | KP858119 | Brazil | V | 5L | 5L3 |
| 4073 | 2013 | KY818236 | Brazil | V | 5L | 5L3 |
| 4074 | 2013 | KY818239 | Brazil | V | 5L | 5L3 |
| 4075 | 2016 | KX768338 | Argentina | V | 5L | 5L3 |
| 4076 | 2016 | KX768363 | Argentina | V | 5L | 5L3 |
| 4077 | 2016 | KX768364 | Argentina | V | 5L | 5L3 |
| 4078 | 2016 | KX768365 | Argentina | V | 5L | 5L3 |
| 4079 | 2016 | KX768366 | Argentina | V | 5L | 5L3 |
| 4080 | 2016 | KX768367 | Argentina | V | 5L | 5L3 |
| 4081 | 2016 | KX768368 | Argentina | V | 5L | 5L3 |
| 4082 | 2016 | KX768369 | Argentina | V | 5L | 5L3 |
| 4083 | 2016 | KX768370 | Argentina | V | 5L | 5L3 |
| 4084 | 2016 | KX768371 | Argentina | V | 5L | 5L3 |
| 4085 | 2016 | KX768372 | Argentina | V | 5L | 5L3 |
| 4086 | 2016 | KX768373 | Argentina | V | 5L | 5L3 |
| 4087 | 2016 | KX768374 | Argentina | V | 5L | 5L3 |
| 4088 | 2016 | KX768375 | Argentina | V | 5L | 5L3 |
| 4089 | 2016 | KX768376 | Argentina | V | 5L | 5L3 |
| 4090 | 2016 | KY283849 | Argentina | V | 5L | 5L3 |
| 4091 | 1991 | AF425626 | Peru | V | 5M | 5M1 |
| 4092 | 1993 | FJ410184 | Puerto_Rico | V | 5M | 5M2 |
| 4093 | 1995 | FJ205875 | USA | V | 5M | 5M3 |
| 4094 | 1995 | JN379486 | Barbados | V | 5M | 5M4 |
| 4095 | 2003 | JN379472 | Barbados | V | 5M | 5M5 |
| 4096 | 1987 | FJ410190 | Puerto_Rico | V | 5M | 5M6 |
| 4097 | 1993 | FJ410183 | Puerto_Rico | V | 5M | 5M6 |
| 4098 | 1993 | FJ410185 | Puerto_Rico | V | 5M | 5M6 |
| 4099 | 1995 | FJ410181 | Puerto_Rico | V | 5M | 5M6 |
| 4100 | 1995 | KF955439 | Puerto_Rico | V | 5M | 5M6 |
| 4101 | 1996 | KF921911 | Puerto_Rico | V | 5M | 5M6 |
| 4102 | 1998 | EU482567 | Puerto_Rico | V | 5M | 5M6 |
| 4103 | 1998 | KF955438 | Puerto_Rico | V | 5M | 5M6 |
| 4104 | 1987 | FJ478458 | Puerto_Rico | V | 5M | 5M7 |
| 4105 | 1992 | FJ410187 | Puerto_Rico | V | 5M | 5M7 |
| 4106 | 1994 | FJ410175 | Puerto_Rico | V | 5M | 5M7 |
| 4107 | 1994 | FJ410179 | Puerto_Rico | V | 5M | 5M7 |
| 4108 | 1995 | FJ390374 | Puerto_Rico | V | 5M | 5M7 |
| 4109 | 1995 | FJ410174 | Puerto_Rico | V | 5M | 5M7 |
| 4110 | 1995 | FJ410180 | Puerto_Rico | V | 5M | 5M7 |
| 4111 | 1996 | FJ410182 | Puerto_Rico | V | 5M | 5M7 |
| 4112 | 1996 | FJ410189 | Puerto_Rico | V | 5M | 5M7 |
| 4113 | 1998 | EU482592 | Puerto_Rico | V | 5M | 5M7 |
| 4114 | 1998 | FJ205872 | USA | V | 5M | 5M7 |
| 4115 | 1998 | FJ205873 | USA | V | 5M | 5M7 |
| 4116 | 1998 | FJ390378 | Puerto_Rico | V | 5M | 5M7 |
| 4117 | 1998 | FJ390379 | Puerto_Rico | V | 5M | 5M7 |
| 4118 | 1998 | FJ390380 | Puerto_Rico | V | 5M | 5M7 |
| 4119 | 1998 | FJ410173 | Puerto_Rico | V | 5M | 5M7 |
| 4120 | 1998 | KC812277 | Puerto_Rico | V | 5M | 5M7 |
| 4121 | 1998 | KF955437 | Puerto_Rico | V | 5M | 5M7 |
| 4122 | 1999 | AB111065 | Paraguay | V | 5M | 5M7 |
| 4123 | 1999 | AY277652 | Argentina | V | 5M | 5M7 |
| 4124 | 1999 | AY277653 | Argentina | V | 5M | 5M7 |
| 4125 | 1999 | AY277664 | Argentina | V | 5M | 5M7 |
| 4126 | 2000 | AF514878 | Paraguay | V | 5M | 5M7 |
| 4127 | 2000 | AF514889 | Argentina | V | 5M | 5M7 |
| 4128 | 2000 | AY206457 | Argentina | V | 5M | 5M7 |
| 4129 | 2000 | AY277654 | Argentina | V | 5M | 5M7 |
| 4130 | 2000 | AY277655 | Argentina | V | 5M | 5M7 |
| 4131 | 2000 | AY277656 | Argentina | V | 5M | 5M7 |
| 4132 | 2000 | AY277659 | Argentina | V | 5M | 5M7 |
| 4133 | 2000 | AY277660 | Argentina | V | 5M | 5M7 |
| 4134 | 2000 | AY277661 | Argentina | V | 5M | 5M7 |
| 4135 | 2000 | AY277662 | Argentina | V | 5M | 5M7 |
| 4136 | 2000 | AY277663 | Argentina | V | 5M | 5M7 |
| 4137 | 2000 | AY277666 | Argentina | V | 5M | 5M7 |
| 4138 | 1986 | HQ026760 | Brazil | V | 5N | 5N1 |
| 4139 | 1986 | HQ603916 | Brazil | V | 5N | 5N1 |
| 4140 | 1986 | JN122280 | Brazil | V | 5N | 5N1 |
| 4141 | 1988 | KF672761 | Brazil | V | 5N | 5N1 |
| 4142 | 1989 | KF672762 | Brazil | V | 5N | 5N1 |
| 4143 | 1990 | AF226685 | Brazil | V | 5N | 5N1 |
| 4144 | 1998 | FJ639741 | Venezuela | V | 5N | 5N2 |
| 4145 | 1999 | JN379487 | Barbados | V | 5N | 5N2 |
| 4146 | 2000 | GU131833 | Venezuela | V | 5N | 5N2 |
| 4147 | 2001 | JN379470 | Barbados | V | 5N | 5N2 |
| 4148 | 2001 | JN379471 | Barbados | V | 5N | 5N2 |
| 4149 | 1994 | AF425637 | Venezuela | V | 5N | 5N3 |
| 4150 | 1995 | AF425633 | Venezuela | V | 5N | 5N3 |
| 4151 | 1995 | AF425635 | Venezuela | V | 5N | 5N3 |
| 4152 | 1998 | GU056033 | Venezuela | V | 5N | 5N3 |
| 4153 | 2004 | KF955412 | Venezuela | V | 5N | 5N3 |
| 4154 | 2004 | GU131835 | Venezuela | V | 5N | 5N4 |
| 4155 | 1998 | GU056032 | Venezuela | V | 5N | 5N5 |
| 4156 | 2004 | GU131836 | Venezuela | V | 5N | 5N6 |
| 4157 | 1991 | KF672791 | Brazil | V | 5N | 5N7 |
| 4158 | 1994 | HM450079 | Brazil | V | 5N | 5N7 |
| 4159 | 1996 | HM450080 | Brazil | V | 5N | 5N7 |
| 4160 | 1996 | JX669467 | Brazil | V | 5N | 5N7 |
| 4161 | 1997 | AF311956 | Brazil | V | 5N | 5N7 |
| 4162 | 1997 | AF311957 | Brazil | V | 5N | 5N7 |
| 4163 | 1997 | AF311958 | Brazil | V | 5N | 5N7 |
| 4164 | 1997 | AF425614 | Brazil | V | 5N | 5N7 |
| 4165 | 1997 | HM450081 | Brazil | V | 5N | 5N7 |
| 4166 | 1997 | HM450082 | Brazil | V | 5N | 5N7 |
| 4167 | 1997 | JX669468 | Brazil | V | 5N | 5N7 |
| 4168 | 1997 | JX669469 | Brazil | V | 5N | 5N7 |
| 4169 | 1997 | KF672769 | Brazil | V | 5N | 5N7 |
| 4170 | 1997 | KF672770 | Brazil | V | 5N | 5N7 |
| 4171 | 1997 | KF672771 | Brazil | V | 5N | 5N7 |
| 4172 | 1997 | KF672772 | Brazil | V | 5N | 5N7 |
| 4173 | 1997 | KF672773 | Brazil | V | 5N | 5N7 |
| 4174 | 1998 | KF672774 | Brazil | V | 5N | 5N7 |
| 4175 | 1998 | KF672775 | Brazil | V | 5N | 5N7 |
| 4176 | 1998 | KF672776 | Brazil | V | 5N | 5N7 |
| 4177 | 1998 | KF672777 | Brazil | V | 5N | 5N7 |
| 4178 | 1998 | KF672778 | Brazil | V | 5N | 5N7 |
| 4179 | 1998 | KF672779 | Brazil | V | 5N | 5N7 |
| 4180 | 1999 | HM450083 | Brazil | V | 5N | 5N7 |
| 4181 | 1999 | KF672780 | Brazil | V | 5N | 5N7 |
| 4182 | 1999 | KF672781 | Brazil | V | 5N | 5N7 |
| 4183 | 1999 | KF672792 | Brazil | V | 5N | 5N7 |
| 4184 | 2000 | HM450084 | Brazil | V | 5N | 5N7 |
| 4185 | 2000 | HM450085 | Brazil | V | 5N | 5N7 |
| 4186 | 2000 | JX669472 | Brazil | V | 5N | 5N7 |
| 4187 | 2000 | KF672787 | Brazil | V | 5N | 5N7 |
| 4188 | 2001 | AB519681 | Brazil | V | 5N | 5N7 |
| 4189 | 2001 | AF513110 | Brazil | V | 5N | 5N7 |
| 4190 | 2001 | HQ603917 | Brazil | V | 5N | 5N7 |
| 4191 | 2001 | JX669473 | Brazil | V | 5N | 5N7 |
| 4192 | 2001 | JX669474 | Brazil | V | 5N | 5N7 |
| 4193 | 2001 | KF672764 | Brazil | V | 5N | 5N7 |
| 4194 | 2001 | KF672788 | Brazil | V | 5N | 5N7 |
| 4195 | 2002 | JX669475 | Brazil | V | 5N | 5N7 |
| 4196 | 2002 | KF672783 | Brazil | V | 5N | 5N7 |
| 4197 | 1998 | JX669470 | Brazil | V | 5N | 5N8 |
| 4198 | 1999 | JX669471 | Brazil | V | 5N | 5N8 |
| 4199 | 2000 | KF672763 | Brazil | V | 5N | 5N8 |
| 4200 | 2000 | KF672782 | Brazil | V | 5N | 5N8 |
| 4201 | 2000 | HM450086 | Brazil | V | 5N | 5N8 |
| 4202 | 2000 | HM450087 | Brazil | V | 5N | 5N8 |
| 4203 | 2001 | FJ850073 | Brazil | V | 5N | 5N8 |
| 4204 | 2001 | HM450098 | Brazil | V | 5N | 5N8 |
| 4205 | 2000 | AF514876 | Argentina | V | 5N | 5N9 |
| 4206 | 2000 | AF514883 | Paraguay | V | 5N | 5N9 |
| 4207 | 2000 | AF514885 | Argentina | V | 5N | 5N9 |
| 4208 | 2000 | AY277657 | Argentina | V | 5N | 5N9 |
| 4209 | 2000 | AY277658 | Argentina | V | 5N | 5N9 |
| 4210 | 2000 | AY277665 | Argentina | V | 5N | 5N9 |
| 4211 | 2002 | HM450090 | Brazil | V | 5N | 5N9 |
| 4212 | 2002 | HM450091 | Brazil | V | 5N | 5N9 |
| 4213 | 1997 | FJ639735 | Venezuela | V | 5N | 5N10 |
| 4214 | 2006 | HQ332182 | Venezuela | V | 5N | 5N10 |
| 4215 | 2006 | JN819415 | Venezuela | V | 5N | 5N10 |
| 4216 | 2007 | HQ332183 | Venezuela | V | 5N | 5N10 |
| 4217 | 2012 | JX891659 | El_Salvador | V | 5N | 5N10 |
| 4218 | 2012 | JX891660 | El_Salvador | V | 5N | 5N10 |
| 4219 | 2012 | JX891661 | El_Salvador | V | 5N | 5N10 |
| 4220 | 2013 | KY461745 | Costa_Rica | V | 5N | 5N10 |
| 4221 | 2013 | KY461753 | Costa_Rica | V | 5N | 5N10 |
| 4222 | 2013 | KY461754 | Costa_Rica | V | 5N | 5N10 |
| 4223 | 2008 | GQ199859 | Nicaragua | V | 5N | 5N11 |
| 4224 | 2008 | KF955410 | Nicaragua | V | 5N | 5N11 |
| 4225 | 2011 | KF973460 | Nicaragua | V | 5N | 5N11 |
| 4226 | 2012 | KF973454 | Nicaragua | V | 5N | 5N11 |
| 4227 | 2012 | KF973455 | Nicaragua | V | 5N | 5N11 |
| 4228 | 2012 | KF973456 | Nicaragua | V | 5N | 5N11 |
| 4229 | 2012 | KF973458 | Nicaragua | V | 5N | 5N11 |
| 4230 | 2012 | KF973472 | Nicaragua | V | 5N | 5N11 |
| 4231 | 2012 | KF973474 | Nicaragua | V | 5N | 5N11 |
| 4232 | 2009 | JF937635 | Nicaragua | V | 5N | 5N12 |
| 4233 | 2012 | KF973463 | Nicaragua | V | 5N | 5N12 |
| 4234 | 2012 | KF973466 | Nicaragua | V | 5N | 5N12 |
| 4235 | 2012 | KF973467 | Nicaragua | V | 5N | 5N12 |
| 4236 | 2012 | KF973473 | Nicaragua | V | 5N | 5N12 |
| 4237 | 2012 | KF973475 | Nicaragua | V | 5N | 5N12 |
| 4238 | 2012 | KM279411 | Mexico | V | 5N | 5N12 |
| 4239 | 2012 | KM279412 | Mexico | V | 5N | 5N12 |
| 4240 | 2012 | KM279415 | Mexico | V | 5N | 5N12 |
| 4241 | 2012 | KM279416 | Mexico | V | 5N | 5N12 |
| 4242 | 2012 | KM279417 | Mexico | V | 5N | 5N12 |
| 4243 | 2012 | KM279418 | Mexico | V | 5N | 5N12 |
| 4244 | 2012 | KM279419 | Mexico | V | 5N | 5N12 |
| 4245 | 2013 | KY882515 | Mexico | V | 5N | 5N12 |
| 4246 | 2014 | KM458188 | USA | V | 5N | 5N12 |
| 4247 | 2011 | KJ189348 | Mexico | V | 5N | 5N13 |
| 4248 | 2011 | KJ189349 | Mexico | V | 5N | 5N13 |
| 4249 | 2012 | KM279413 | Mexico | V | 5N | 5N13 |
| 4250 | 2013 | KY818080 | Colombia | V | 5N | 5N13 |
| 4251 | 2013 | KY818209 | Mexico | V | 5N | 5N13 |
| 4252 | 2013 | KY818276 | Mexico | V | 5N | 5N13 |
| 4253 | 2013 | KY818277 | Mexico | V | 5N | 5N13 |
| 4254 | 2013 | KY882517 | Mexico | V | 5N | 5N13 |
| 4255 | 2014 | KM458186 | USA | V | 5N | 5N14 |
| 4256 | 2014 | KM458189 | USA | V | 5N | 5N15 |
| 4257 | 1995 | AF425632 | Venezuela | V | 5N | 5N16 |
| 4258 | 2004 | EU596501 | Nicaragua | V | 5O | 5O1 |
| 4259 | 2004 | FJ898437 | Nicaragua | V | 5O | 5O1 |
| 4260 | 2004 | GQ199867 | Nicaragua | V | 5O | 5O1 |
| 4261 | 2004 | GQ199872 | Nicaragua | V | 5O | 5O1 |
| 4262 | 2004 | GQ199873 | Nicaragua | V | 5O | 5O1 |
| 4263 | 2004 | GQ199875 | Nicaragua | V | 5O | 5O1 |
| 4264 | 2005 | FJ410290 | Nicaragua | V | 5O | 5O1 |
| 4265 | 2005 | EU482616 | Nicaragua | V | 5O | 5O1 |
| 4266 | 2005 | EU482617 | Nicaragua | V | 5O | 5O1 |
| 4267 | 2005 | EU482618 | Nicaragua | V | 5O | 5O1 |
| 4268 | 2005 | EU482619 | Nicaragua | V | 5O | 5O1 |
| 4269 | 2005 | EU596502 | Nicaragua | V | 5O | 5O1 |
| 4270 | 2005 | EU596503 | Nicaragua | V | 5O | 5O1 |
| 4271 | 2005 | EU596504 | Nicaragua | V | 5O | 5O1 |
| 4272 | 2005 | FJ024423 | Nicaragua | V | 5O | 5O1 |
| 4273 | 2005 | FJ024478 | Nicaragua | V | 5O | 5O1 |
| 4274 | 2005 | FJ024480 | Nicaragua | V | 5O | 5O1 |
| 4275 | 2005 | FJ024481 | Nicaragua | V | 5O | 5O1 |
| 4276 | 2005 | FJ024482 | Nicaragua | V | 5O | 5O1 |
| 4277 | 2005 | FJ024483 | Nicaragua | V | 5O | 5O1 |
| 4278 | 2005 | FJ024484 | Nicaragua | V | 5O | 5O1 |
| 4279 | 2005 | FJ182002 | Nicaragua | V | 5O | 5O1 |
| 4280 | 2005 | FJ432721 | Nicaragua | V | 5O | 5O1 |
| 4281 | 2005 | FJ547089 | Nicaragua | V | 5O | 5O1 |
| 4282 | 2005 | FJ850114 | Nicaragua | V | 5O | 5O1 |
| 4283 | 2005 | FJ873814 | Nicaragua | V | 5O | 5O1 |
| 4284 | 2005 | JN379474 | Belize | V | 5O | 5O1 |
| 4285 | 2005 | KF955403 | Nicaragua | V | 5O | 5O1 |
| 4286 | 2005 | KF955409 | Nicaragua | V | 5O | 5O1 |
| 4287 | 2005 | KU728184 | Belize | V | 5O | 5O1 |
| 4288 | 2006 | EU448414 | El_Salvador | V | 5O | 5O1 |
| 4289 | 2006 | GQ868499 | Mexico | V | 5O | 5O1 |
| 4290 | 2006 | FJ024479 | Nicaragua | V | 5O | 5O1 |
| 4291 | 2006 | FJ547068 | Nicaragua | V | 5O | 5O1 |
| 4292 | 2006 | FJ810419 | Nicaragua | V | 5O | 5O1 |
| 4293 | 2006 | GQ868498 | Mexico | V | 5O | 5O1 |
| 4294 | 2006 | GU131957 | Mexico | V | 5O | 5O1 |
| 4295 | 2006 | GU131958 | Mexico | V | 5O | 5O1 |
| 4296 | 2006 | HM171557 | Mexico | V | 5O | 5O1 |
| 4297 | 2006 | HM171558 | Mexico | V | 5O | 5O1 |
| 4298 | 2006 | HM171560 | Mexico | V | 5O | 5O1 |
| 4299 | 2007 | FJ898433 | Nicaragua | V | 5O | 5O1 |
| 4300 | 2007 | GQ868510 | Mexico | V | 5O | 5O1 |
| 4301 | 2007 | GU131970 | Mexico | V | 5O | 5O1 |
| 4302 | 2007 | KJ189327 | Mexico | V | 5O | 5O1 |
| 4303 | 2007 | KJ189328 | Mexico | V | 5O | 5O1 |
| 4304 | 2007 | GQ868501 | Mexico | V | 5O | 5O1 |
| 4305 | 2007 | GQ868503 | Mexico | V | 5O | 5O1 |
| 4306 | 2007 | GQ868509 | Mexico | V | 5O | 5O1 |
| 4307 | 2007 | GQ868517 | Mexico | V | 5O | 5O1 |
| 4308 | 2007 | GQ868524 | Mexico | V | 5O | 5O1 |
| 4309 | 2007 | GQ868527 | Mexico | V | 5O | 5O1 |
| 4310 | 2007 | GU131960 | Mexico | V | 5O | 5O1 |
| 4311 | 2007 | GU131961 | Mexico | V | 5O | 5O1 |
| 4312 | 2007 | GU131964 | Mexico | V | 5O | 5O1 |
| 4313 | 2007 | GU131966 | Mexico | V | 5O | 5O1 |
| 4314 | 2007 | GU131968 | Mexico | V | 5O | 5O1 |
| 4315 | 2007 | GU131976 | Mexico | V | 5O | 5O1 |
| 4316 | 2007 | HM171561 | Mexico | V | 5O | 5O1 |
| 4317 | 2007 | HM171564 | Mexico | V | 5O | 5O1 |
| 4318 | 2007 | HM171565 | Mexico | V | 5O | 5O1 |
| 4319 | 2007 | KF955419 | Mexico | V | 5O | 5O1 |
| 4320 | 2007 | KF955422 | Mexico | V | 5O | 5O1 |
| 4321 | 2007 | KF955428 | Mexico | V | 5O | 5O1 |
| 4322 | 2007 | KF955442 | Mexico | V | 5O | 5O1 |
| 4323 | 2007 | KF955443 | Mexico | V | 5O | 5O1 |
| 4324 | 2007 | KJ189318 | Mexico | V | 5O | 5O1 |
| 4325 | 2007 | KJ189319 | Mexico | V | 5O | 5O1 |
| 4326 | 2007 | KJ189320 | Mexico | V | 5O | 5O1 |
| 4327 | 2007 | KJ189321 | Mexico | V | 5O | 5O1 |
| 4328 | 2007 | KY461737 | Costa_Rica | V | 5O | 5O1 |
| 4329 | 2007 | KY461738 | Costa_Rica | V | 5O | 5O1 |
| 4330 | 2008 | JF967804 | Honduras | V | 5O | 5O1 |
| 4331 | 2008 | GQ868535 | Mexico | V | 5O | 5O1 |
| 4332 | 2008 | GQ868536 | Mexico | V | 5O | 5O1 |
| 4333 | 2008 | GQ868539 | Mexico | V | 5O | 5O1 |
| 4334 | 2008 | KF955432 | Mexico | V | 5O | 5O1 |
| 4335 | 2008 | KF955433 | Mexico | V | 5O | 5O1 |
| 4336 | 2008 | KJ189313 | Mexico | V | 5O | 5O1 |
| 4337 | 2008 | KJ189331 | Mexico | V | 5O | 5O1 |
| 4338 | 2008 | KJ189332 | Mexico | V | 5O | 5O1 |
| 4339 | 2008 | KJ189333 | Mexico | V | 5O | 5O1 |
| 4340 | 2008 | KJ189337 | Mexico | V | 5O | 5O1 |
| 4341 | 2008 | KJ189338 | Mexico | V | 5O | 5O1 |
| 4342 | 2008 | KJ189339 | Mexico | V | 5O | 5O1 |
| 4343 | 2009 | JQ425062 | USA | V | 5O | 5O1 |
| 4344 | 2009 | KJ189341 | Mexico | V | 5O | 5O1 |
| 4345 | 2009 | KJ189342 | Mexico | V | 5O | 5O1 |
| 4346 | 2009 | JF937644 | Nicaragua | V | 5O | 5O1 |
| 4347 | 2009 | JF937645 | Nicaragua | V | 5O | 5O1 |
| 4348 | 2009 | JQ287666 | Nicaragua | V | 5O | 5O1 |
| 4349 | 2009 | KJ189345 | Mexico | V | 5O | 5O1 |
| 4350 | 2010 | KU728185 | Belize | V | 5O | 5O1 |
| 4351 | 2013 | KY461750 | Costa_Rica | V | 5O | 5O1 |
| 4352 | 2013 | KY818139 | Honduras | V | 5O | 5O1 |
| 4353 | 2014 | KM458187 | USA | V | 5O | 5O1 |
| 4354 | 2014 | KM458190 | USA | V | 5O | 5O1 |
| 4355 | 2014 | KY818140 | Honduras | V | 5O | 5O1 |
| 4356 | 2006 | FJ562104 | Nicaragua | V | 5O | 5O2 |
| 4357 | 2006 | JN819403 | Nicaragua | V | 5O | 5O2 |
| 4358 | 2007 | KY461736 | Costa_Rica | V | 5O | 5O2 |
| 4359 | 2007 | KY461739 | Costa_Rica | V | 5O | 5O2 |
| 4360 | 2008 | FJ547088 | Nicaragua | V | 5O | 5O2 |
| 4361 | 2008 | GQ199857 | Nicaragua | V | 5O | 5O2 |
| 4362 | 2008 | GQ199858 | Nicaragua | V | 5O | 5O2 |
| 4363 | 2009 | JQ425061 | USA | V | 5O | 5O2 |
| 4364 | 2009 | JQ425063 | USA | V | 5O | 5O2 |
| 4365 | 2009 | JQ425068 | USA | V | 5O | 5O2 |
| 4366 | 2010 | JF519855 | USA | V | 5O | 5O2 |
| 4367 | 2010 | JQ045564 | USA | V | 5O | 5O2 |
| 4368 | 2010 | JQ425069 | USA | V | 5O | 5O2 |
| 4369 | 2010 | JQ425070 | USA | V | 5O | 5O2 |
| 4370 | 2010 | JQ425071 | USA | V | 5O | 5O2 |
| 4371 | 2010 | JQ425072 | USA | V | 5O | 5O2 |
| 4372 | 2010 | JQ675358 | USA | V | 5O | 5O2 |
| 4373 | 2011 | KF973453 | Nicaragua | V | 5O | 5O2 |
| 4374 | 2011 | KF973459 | Nicaragua | V | 5O | 5O2 |
| 4375 | 2011 | KF973462 | Nicaragua | V | 5O | 5O2 |
| 4376 | 2012 | KF973457 | Nicaragua | V | 5O | 5O2 |
| 4377 | 2012 | KF973461 | Nicaragua | V | 5O | 5O2 |
| 4378 | 2012 | KF973464 | Nicaragua | V | 5O | 5O2 |
| 4379 | 2012 | KF973465 | Nicaragua | V | 5O | 5O2 |
| 4380 | 2012 | KF973468 | Nicaragua | V | 5O | 5O2 |
| 4381 | 2012 | KF973469 | Nicaragua | V | 5O | 5O2 |
| 4382 | 2012 | KF973470 | Nicaragua | V | 5O | 5O2 |
| 4383 | 2012 | KF973471 | Nicaragua | V | 5O | 5O2 |
| 4384 | 2012 | KY461755 | Costa_Rica | V | 5O | 5O2 |
| 4385 | 2013 | KJ415092 | Angola | V | 5O | 5O2 |
| 4386 | 2013 | KY461741 | Costa_Rica | V | 5O | 5O2 |
| 4387 | 2013 | KY461740 | Costa_Rica | V | 5O | 5O2 |
| 4388 | 2013 | KY461742 | Costa_Rica | V | 5O | 5O2 |
| 4389 | 2013 | KY461743 | Costa_Rica | V | 5O | 5O2 |
| 4390 | 2013 | KY461744 | Costa_Rica | V | 5O | 5O2 |
| 4391 | 2013 | KY461746 | Costa_Rica | V | 5O | 5O2 |
| 4392 | 2013 | KY461747 | Costa_Rica | V | 5O | 5O2 |
| 4393 | 2013 | KY461748 | Costa_Rica | V | 5O | 5O2 |
| 4394 | 2013 | KY461751 | Costa_Rica | V | 5O | 5O2 |
| 4395 | 2013 | KY461752 | Costa_Rica | V | 5O | 5O2 |
| 4396 | 2013 | KY882503 | Honduras | V | 5O | 5O2 |
| 4397 | 2014 | KU509312 | Nicaragua | V | 5O | 5O2 |
| 4398 | 2014 | KY461749 | Costa_Rica | V | 5O | 5O2 |
| 4399 | 2016 | KY829115 | Saint_Barthelemy | V | 5O | 5O2 |
| 4400 | 2005 | EU482615 | Nicaragua | V | 5O | 5O3 |
| 4401 | 2005 | FJ024485 | Nicaragua | V | 5O | 5O3 |
| 4402 | 2005 | FJ432720 | Nicaragua | V | 5O | 5O3 |
| 4403 | 2005 | FJ850113 | Nicaragua | V | 5O | 5O3 |
| 4404 | 2005 | JN819402 | Nicaragua | V | 5O | 5O3 |
| 4405 | 2005 | KU728183 | Belize | V | 5O | 5O3 |
| 4406 | 2006 | GU131956 | Mexico | V | 5O | 5O3 |
| 4407 | 2006 | HM171559 | Mexico | V | 5O | 5O3 |
| 4408 | 2006 | HM171562 | Mexico | V | 5O | 5O3 |
| 4409 | 2006 | HM171566 | Mexico | V | 5O | 5O3 |
| 4410 | 2006 | HM171567 | Mexico | V | 5O | 5O3 |
| 4411 | 2006 | KF955415 | Mexico | V | 5O | 5O3 |
| 4412 | 2006 | KF955416 | Mexico | V | 5O | 5O3 |
| 4413 | 2006 | KF955417 | Mexico | V | 5O | 5O3 |
| 4414 | 2006 | HM171568 | Mexico | V | 5O | 5O3 |
| 4415 | 2006 | HM171570 | Mexico | V | 5O | 5O3 |
| 4416 | 2007 | GQ868500 | Mexico | V | 5O | 5O3 |
| 4417 | 2007 | GQ868502 | Mexico | V | 5O | 5O3 |
| 4418 | 2007 | GQ868505 | Mexico | V | 5O | 5O3 |
| 4419 | 2007 | GQ868507 | Mexico | V | 5O | 5O3 |
| 4420 | 2007 | GQ868508 | Mexico | V | 5O | 5O3 |
| 4421 | 2007 | GQ868513 | Mexico | V | 5O | 5O3 |
| 4422 | 2007 | GQ868514 | Mexico | V | 5O | 5O3 |
| 4423 | 2007 | GQ868518 | Mexico | V | 5O | 5O3 |
| 4424 | 2007 | GQ868519 | Mexico | V | 5O | 5O3 |
| 4425 | 2007 | GQ868520 | Mexico | V | 5O | 5O3 |
| 4426 | 2007 | GQ868522 | Mexico | V | 5O | 5O3 |
| 4427 | 2007 | GQ868528 | Mexico | V | 5O | 5O3 |
| 4428 | 2007 | GU131963 | Mexico | V | 5O | 5O3 |
| 4429 | 2007 | GU131969 | Mexico | V | 5O | 5O3 |
| 4430 | 2007 | GU131977 | Mexico | V | 5O | 5O3 |
| 4431 | 2007 | GU131979 | Mexico | V | 5O | 5O3 |
| 4432 | 2007 | GU131981 | Mexico | V | 5O | 5O3 |
| 4433 | 2007 | HM171563 | Mexico | V | 5O | 5O3 |
| 4434 | 2007 | HQ166036 | Mexico | V | 5O | 5O3 |
| 4435 | 2007 | KF955427 | Mexico | V | 5O | 5O3 |
| 4436 | 2007 | KJ189317 | Mexico | V | 5O | 5O3 |
| 4437 | 2007 | KJ189325 | Mexico | V | 5O | 5O3 |
| 4438 | 2007 | KJ189326 | Mexico | V | 5O | 5O3 |
| 4439 | 2007 | KJ189329 | Mexico | V | 5O | 5O3 |
| 4440 | 2007 | GQ868504 | Mexico | V | 5O | 5O3 |
| 4441 | 2007 | GQ868506 | Mexico | V | 5O | 5O3 |
| 4442 | 2007 | GQ868511 | Mexico | V | 5O | 5O3 |
| 4443 | 2007 | GQ868512 | Mexico | V | 5O | 5O3 |
| 4444 | 2007 | GQ868521 | Mexico | V | 5O | 5O3 |
| 4445 | 2007 | GQ868523 | Mexico | V | 5O | 5O3 |
| 4446 | 2007 | GQ868525 | Mexico | V | 5O | 5O3 |
| 4447 | 2007 | GQ868526 | Mexico | V | 5O | 5O3 |
| 4448 | 2007 | GU131962 | Mexico | V | 5O | 5O3 |
| 4449 | 2007 | GU131967 | Mexico | V | 5O | 5O3 |
| 4450 | 2007 | GU131971 | Mexico | V | 5O | 5O3 |
| 4451 | 2007 | GU131972 | Mexico | V | 5O | 5O3 |
| 4452 | 2007 | GU131973 | Mexico | V | 5O | 5O3 |
| 4453 | 2007 | GU131978 | Mexico | V | 5O | 5O3 |
| 4454 | 2007 | GU131980 | Mexico | V | 5O | 5O3 |
| 4455 | 2007 | HM171569 | Mexico | V | 5O | 5O3 |
| 4456 | 2007 | HQ166035 | Mexico | V | 5O | 5O3 |
| 4457 | 2007 | KF955420 | Mexico | V | 5O | 5O3 |
| 4458 | 2007 | KF955421 | Mexico | V | 5O | 5O3 |
| 4459 | 2007 | KF955423 | Mexico | V | 5O | 5O3 |
| 4460 | 2007 | KJ189322 | Mexico | V | 5O | 5O3 |
| 4461 | 2007 | KJ189324 | Mexico | V | 5O | 5O3 |
| 4462 | 2007 | KJ189330 | Mexico | V | 5O | 5O3 |
| 4463 | 2008 | GQ868529 | Mexico | V | 5O | 5O3 |
| 4464 | 2008 | GQ868532 | Mexico | V | 5O | 5O3 |
| 4465 | 2008 | GQ868533 | Mexico | V | 5O | 5O3 |
| 4466 | 2008 | GQ868538 | Mexico | V | 5O | 5O3 |
| 4467 | 2008 | GU131984 | Mexico | V | 5O | 5O3 |
| 4468 | 2008 | HQ166037 | Mexico | V | 5O | 5O3 |
| 4469 | 2008 | KJ189312 | Mexico | V | 5O | 5O3 |
| 4470 | 2008 | KJ189314 | Mexico | V | 5O | 5O3 |
| 4471 | 2008 | GQ868531 | Mexico | V | 5O | 5O3 |
| 4472 | 2008 | GQ868534 | Mexico | V | 5O | 5O3 |
| 4473 | 2008 | GU131982 | Mexico | V | 5O | 5O3 |
| 4474 | 2008 | GU131983 | Mexico | V | 5O | 5O3 |
| 4475 | 2008 | KJ189315 | Mexico | V | 5O | 5O3 |
| 4476 | 2008 | KJ189334 | Mexico | V | 5O | 5O3 |
| 4477 | 2008 | KJ189335 | Mexico | V | 5O | 5O3 |
| 4478 | 2008 | KJ189336 | Mexico | V | 5O | 5O3 |
| 4479 | 2008 | KJ189340 | Mexico | V | 5O | 5O3 |
| 4480 | 2009 | KJ189316 | Mexico | V | 5O | 5O3 |
| 4481 | 2009 | KJ189346 | Mexico | V | 5O | 5O3 |
| 4482 | 2009 | KJ189343 | Mexico | V | 5O | 5O3 |
| 4483 | 2009 | KJ189344 | Mexico | V | 5O | 5O3 |
| 4484 | 2010 | JQ065903 | Mexico | V | 5O | 5O3 |
| 4485 | 2010 | JQ065906 | Mexico | V | 5O | 5O3 |
| 4486 | 2010 | JQ065907 | Mexico | V | 5O | 5O3 |
| 4487 | 2010 | JQ065917 | Mexico | V | 5O | 5O3 |
| 4488 | 2010 | JQ065918 | Mexico | V | 5O | 5O3 |
| 4489 | 2010 | JQ065921 | Mexico | V | 5O | 5O3 |
| 4490 | 2010 | JQ065938 | Mexico | V | 5O | 5O3 |
| 4491 | 2010 | JQ065942 | Mexico | V | 5O | 5O3 |
| 4492 | 2010 | JQ065945 | Mexico | V | 5O | 5O3 |
| 4493 | 2010 | JQ065950 | Mexico | V | 5O | 5O3 |
| 4494 | 2010 | JQ065957 | Mexico | V | 5O | 5O3 |
| 4495 | 2012 | KY818279 | Mexico | V | 5O | 5O3 |
| 4496 | 2012 | KY818286 | Mexico | V | 5O | 5O3 |
| 4497 | 2007 | GU131965 | Mexico | V | 5O | 5O4 |
| 4498 | 2007 | KJ189323 | Mexico | V | 5O | 5O4 |
| 4499 | 2008 | GQ868530 | Mexico | V | 5O | 5O4 |
| 4500 | 2008 | GQ868537 | Mexico | V | 5O | 5O4 |
| 4501 | 2009 | JQ425064 | USA | V | 5O | 5O4 |
| 4502 | 2009 | JQ425065 | USA | V | 5O | 5O4 |
| 4503 | 2009 | JQ425066 | USA | V | 5O | 5O4 |
| 4504 | 2009 | KJ189347 | Mexico | V | 5O | 5O4 |
| 4505 | 2010 | JQ065899 | Mexico | V | 5O | 5O4 |
| 4506 | 2010 | JQ065900 | Mexico | V | 5O | 5O4 |
| 4507 | 2010 | JQ065901 | Mexico | V | 5O | 5O4 |
| 4508 | 2010 | JQ065902 | Mexico | V | 5O | 5O4 |
| 4509 | 2010 | JQ065904 | Mexico | V | 5O | 5O4 |
| 4510 | 2010 | JQ065905 | Mexico | V | 5O | 5O4 |
| 4511 | 2010 | JQ065908 | Mexico | V | 5O | 5O4 |
| 4512 | 2010 | JQ065909 | Mexico | V | 5O | 5O4 |
| 4513 | 2010 | JQ065910 | Mexico | V | 5O | 5O4 |
| 4514 | 2010 | JQ065911 | Mexico | V | 5O | 5O4 |
| 4515 | 2010 | JQ065912 | Mexico | V | 5O | 5O4 |
| 4516 | 2010 | JQ065913 | Mexico | V | 5O | 5O4 |
| 4517 | 2010 | JQ065914 | Mexico | V | 5O | 5O4 |
| 4518 | 2010 | JQ065915 | Mexico | V | 5O | 5O4 |
| 4519 | 2010 | JQ065916 | Mexico | V | 5O | 5O4 |
| 4520 | 2010 | JQ065919 | Mexico | V | 5O | 5O4 |
| 4521 | 2010 | JQ065920 | Mexico | V | 5O | 5O4 |
| 4522 | 2010 | JQ065922 | Mexico | V | 5O | 5O4 |
| 4523 | 2010 | JQ065923 | Mexico | V | 5O | 5O4 |
| 4524 | 2010 | JQ065924 | Mexico | V | 5O | 5O4 |
| 4525 | 2010 | JQ065925 | Mexico | V | 5O | 5O4 |
| 4526 | 2010 | JQ065926 | Mexico | V | 5O | 5O4 |
| 4527 | 2010 | JQ065927 | Mexico | V | 5O | 5O4 |
| 4528 | 2010 | JQ065928 | Mexico | V | 5O | 5O4 |
| 4529 | 2010 | JQ065929 | Mexico | V | 5O | 5O4 |
| 4530 | 2010 | JQ065930 | Mexico | V | 5O | 5O4 |
| 4531 | 2010 | JQ065931 | Mexico | V | 5O | 5O4 |
| 4532 | 2010 | JQ065932 | Mexico | V | 5O | 5O4 |
| 4533 | 2010 | JQ065933 | Mexico | V | 5O | 5O4 |
| 4534 | 2010 | JQ065934 | Mexico | V | 5O | 5O4 |
| 4535 | 2010 | JQ065935 | Mexico | V | 5O | 5O4 |
| 4536 | 2010 | JQ065937 | Mexico | V | 5O | 5O4 |
| 4537 | 2010 | JQ065939 | Mexico | V | 5O | 5O4 |
| 4538 | 2010 | JQ065941 | Mexico | V | 5O | 5O4 |
| 4539 | 2010 | JQ065943 | Mexico | V | 5O | 5O4 |
| 4540 | 2010 | JQ065946 | Mexico | V | 5O | 5O4 |
| 4541 | 2010 | JQ065947 | Mexico | V | 5O | 5O4 |
| 4542 | 2010 | JQ065948 | Mexico | V | 5O | 5O4 |
| 4543 | 2010 | JQ065949 | Mexico | V | 5O | 5O4 |
| 4544 | 2010 | JQ065951 | Mexico | V | 5O | 5O4 |
| 4545 | 2010 | JQ065953 | Mexico | V | 5O | 5O4 |
| 4546 | 2010 | JQ065954 | Mexico | V | 5O | 5O4 |
| 4547 | 2010 | JQ065956 | Mexico | V | 5O | 5O4 |
| 4548 | 2010 | JQ920428 | Mexico | V | 5O | 5O4 |
| 4549 | 2010 | JQ920429 | Mexico | V | 5O | 5O4 |
| 4550 | 2010 | JQ920430 | Mexico | V | 5O | 5O4 |
| 4551 | 2010 | JQ920431 | Mexico | V | 5O | 5O4 |
| 4552 | 2010 | JQ920432 | Mexico | V | 5O | 5O4 |
| 4553 | 2011 | KJ189307 | Mexico | V | 5O | 5O4 |
| 4554 | 2011 | KJ189369 | Mexico | V | 5O | 5O4 |
| 4555 | 2011 | KM279414 | Mexico | V | 5O | 5O4 |
| 4556 | 2011 | KJ189306 | Mexico | V | 5O | 5O4 |
| 4557 | 2012 | KJ189368 | Mexico | V | 5O | 5O4 |
| 4558 | 2012 | KM279420 | Mexico | V | 5O | 5O4 |
| 4559 | 2012 | KY818267 | Mexico | V | 5O | 5O4 |
| 4560 | 2012 | KY818268 | Mexico | V | 5O | 5O4 |
| 4561 | 2012 | KY818272 | Mexico | V | 5O | 5O4 |
| 4562 | 2012 | KY818275 | Mexico | V | 5O | 5O4 |
| 4563 | 2012 | KY818260 | Mexico | V | 5O | 5O4 |
| 4564 | 2012 | KY818261 | Mexico | V | 5O | 5O4 |
| 4565 | 2012 | KY818262 | Mexico | V | 5O | 5O4 |
| 4566 | 2012 | KY818263 | Mexico | V | 5O | 5O4 |
| 4567 | 2012 | KY818264 | Mexico | V | 5O | 5O4 |
| 4568 | 2012 | KY818265 | Mexico | V | 5O | 5O4 |
| 4569 | 2012 | KY818269 | Mexico | V | 5O | 5O4 |
| 4570 | 2012 | KY818271 | Mexico | V | 5O | 5O4 |
| 4571 | 2012 | KY818273 | Mexico | V | 5O | 5O4 |
| 4572 | 2012 | KY818274 | Mexico | V | 5O | 5O4 |
| 4573 | 2012 | KY818278 | Mexico | V | 5O | 5O4 |
| 4574 | 2012 | KY818280 | Mexico | V | 5O | 5O4 |
| 4575 | 2012 | KY818281 | Mexico | V | 5O | 5O4 |
| 4576 | 2012 | KY818282 | Mexico | V | 5O | 5O4 |
| 4577 | 2012 | KY818283 | Mexico | V | 5O | 5O4 |
| 4578 | 2012 | KY818284 | Mexico | V | 5O | 5O4 |
| 4579 | 2012 | KY818285 | Mexico | V | 5O | 5O4 |
| 4580 | 2012 | KY818287 | Mexico | V | 5O | 5O4 |
| 4581 | 2012 | KY882518 | Mexico | V | 5O | 5O4 |
| 4582 | 2013 | KY818266 | Mexico | V | 5O | 5O4 |
| 4583 | 1994 | AF425636 | Venezuela | V | 5P | 5P1 |
| 4584 | 1995 | AF425638 | Venezuela | V | 5P | 5P1 |
| 4585 | 1997 | AF425634 | Venezuela | V | 5P | 5P1 |
| 4586 | 1998 | FJ639740 | Venezuela | V | 5P | 5P1 |
| 4587 | 1998 | GQ868559 | Colombia | V | 5P | 5P1 |
| 4588 | 1998 | GQ868560 | Colombia | V | 5P | 5P1 |
| 4589 | 1998 | JQ581648 | Colombia | V | 5P | 5P1 |
| 4590 | 1998 | JQ581649 | Colombia | V | 5P | 5P1 |
| 4591 | 1998 | KJ189302 | Colombia | V | 5P | 5P1 |
| 4592 | 1999 | FJ639743 | Venezuela | V | 5P | 5P1 |
| 4593 | 1999 | GQ868561 | Colombia | V | 5P | 5P1 |
| 4594 | 1999 | JQ581650 | Colombia | V | 5P | 5P1 |
| 4595 | 2001 | GU131834 | Venezuela | V | 5P | 5P1 |
| 4596 | 2001 | GU131948 | Colombia | V | 5P | 5P1 |
| 4597 | 2001 | JQ581651 | Colombia | V | 5P | 5P1 |
| 4598 | 2002 | JQ581652 | Colombia | V | 5P | 5P1 |
| 4599 | 2005 | GU131837 | Venezuela | V | 5P | 5P1 |
| 4600 | 2008 | FJ850104 | Venezuela | V | 5P | 5P1 |
| 4601 | 2009 | JQ425067 | USA | V | 5P | 5P1 |
| 4602 | 2008 | GQ868570 | Colombia | V | 5P | 5P2 |
| 4603 | 2013 | KY818067 | Colombia | V | 5P | 5P2 |
| 4604 | 2014 | KY474303 | Ecuador | V | 5P | 5P2 |
| 4605 | 2014 | KY474304 | Ecuador | V | 5P | 5P2 |
| 4606 | 2014 | KY474307 | Ecuador | V | 5P | 5P2 |
| 4607 | 2014 | MF797878 | Ecuador | V | 5P | 5P2 |
| 4608 | 2008 | JQ581602 | Colombia | V | 5P | 5P3 |
| 4609 | 2008 | JQ581603 | Colombia | V | 5P | 5P3 |
| 4610 | 2008 | JQ581604 | Colombia | V | 5P | 5P3 |
| 4611 | 2012 | KY818097 | Colombia | V | 5P | 5P3 |
| 4612 | 2013 | KY818060 | Colombia | V | 5P | 5P3 |
| 4613 | 2013 | KY818095 | Colombia | V | 5P | 5P3 |
| 4614 | 2013 | KY818096 | Colombia | V | 5P | 5P3 |
| 4615 | 2013 | KY818098 | Colombia | V | 5P | 5P3 |
| 4616 | 2013 | KY818100 | Colombia | V | 5P | 5P3 |
| 4617 | 2013 | KY818101 | Colombia | V | 5P | 5P3 |
| 4618 | 2015 | KX901656 | Colombia | V | 5P | 5P3 |
| 4619 | 2012 | KY818270 | Mexico | V | 5P | 5P4 |
| 4620 | 2013 | KY882519 | Colombia | V | 5P | 5P5 |
| 4621 | 2012 | KY818144 | Colombia | V | 5P | 5P6 |
| 4622 | 2013 | KY818071 | Colombia | V | 5P | 5P6 |
| 4623 | 2013 | KY818142 | Colombia | V | 5P | 5P6 |
| 4624 | 2013 | KY818143 | Colombia | V | 5P | 5P6 |
| 4625 | 2013 | KY818141 | Colombia | V | 5P | 5P6 |
| 4626 | 2013 | KY818213 | Colombia | V | 5P | 5P6 |
| 4627 | 2013 | KY818222 | Colombia | V | 5P | 5P6 |
| 4628 | 2013 | KY882521 | Colombia | V | 5P | 5P6 |
| 4629 | 2012 | KY818082 | Colombia | V | 5P | 5P7 |
| 4630 | 2012 | KY818085 | Colombia | V | 5P | 5P7 |
| 4631 | 2012 | KY818221 | Colombia | V | 5P | 5P7 |
| 4632 | 2012 | KY818229 | Colombia | V | 5P | 5P7 |
| 4633 | 2013 | KY818076 | Colombia | V | 5P | 5P7 |
| 4634 | 2013 | KY818077 | Colombia | V | 5P | 5P7 |
| 4635 | 2013 | KY818078 | Colombia | V | 5P | 5P7 |
| 4636 | 2013 | KY818079 | Colombia | V | 5P | 5P7 |
| 4637 | 2013 | KY818081 | Colombia | V | 5P | 5P7 |
| 4638 | 2013 | KY818084 | Colombia | V | 5P | 5P7 |
| 4639 | 2013 | KY818086 | Colombia | V | 5P | 5P7 |
| 4640 | 2013 | KY818087 | Colombia | V | 5P | 5P7 |
| 4641 | 2013 | KY818088 | Colombia | V | 5P | 5P7 |
| 4642 | 2013 | KY818063 | Colombia | V | 5P | 5P7 |
| 4643 | 2013 | KY818064 | Colombia | V | 5P | 5P7 |
| 4644 | 2013 | KY818072 | Colombia | V | 5P | 5P7 |
| 4645 | 2013 | KY818073 | Colombia | V | 5P | 5P7 |
| 4646 | 2013 | KY818074 | Colombia | V | 5P | 5P7 |
| 4647 | 2013 | KY818075 | Colombia | V | 5P | 5P7 |
| 4648 | 2013 | KY818083 | Colombia | V | 5P | 5P7 |
| 4649 | 2013 | KY818089 | Colombia | V | 5P | 5P7 |
| 4650 | 2013 | KY818211 | Colombia | V | 5P | 5P7 |
| 4651 | 2013 | KY818214 | Colombia | V | 5P | 5P7 |
| 4652 | 2013 | KY818215 | Colombia | V | 5P | 5P7 |
| 4653 | 2013 | KY818216 | Colombia | V | 5P | 5P7 |
| 4654 | 2013 | KY818217 | Colombia | V | 5P | 5P7 |
| 4655 | 2013 | KY818219 | Colombia | V | 5P | 5P7 |
| 4656 | 2013 | KY818223 | Colombia | V | 5P | 5P7 |
| 4657 | 2013 | KY818224 | Colombia | V | 5P | 5P7 |
| 4658 | 2013 | KY818228 | Colombia | V | 5P | 5P7 |
| 4659 | 2013 | KY882523 | Colombia | V | 5P | 5P7 |
| 4660 | 2014 | KX901655 | Colombia | V | 5P | 5P7 |
| 4661 | 1998 | KJ189303 | Colombia | V | 5P | 5P8 |
| 4662 | 2004 | JQ581632 | Colombia | V | 5P | 5P8 |
| 4663 | 2005 | KJ189304 | Colombia | V | 5P | 5P8 |
| 4664 | 2006 | JQ581621 | Colombia | V | 5P | 5P8 |
| 4665 | 2006 | GQ868563 | Colombia | V | 5P | 5P8 |
| 4666 | 2006 | GQ868564 | Colombia | V | 5P | 5P8 |
| 4667 | 2006 | GQ868565 | Colombia | V | 5P | 5P8 |
| 4668 | 2006 | GU131949 | Colombia | V | 5P | 5P8 |
| 4669 | 2006 | JQ581635 | Colombia | V | 5P | 5P8 |
| 4670 | 2006 | JQ581638 | Colombia | V | 5P | 5P8 |
| 4671 | 2006 | JQ581639 | Colombia | V | 5P | 5P8 |
| 4672 | 2006 | JQ581640 | Colombia | V | 5P | 5P8 |
| 4673 | 2006 | JQ581641 | Colombia | V | 5P | 5P8 |
| 4674 | 2006 | JQ581642 | Colombia | V | 5P | 5P8 |
| 4675 | 2006 | JQ581643 | Colombia | V | 5P | 5P8 |
| 4676 | 2006 | JQ581644 | Colombia | V | 5P | 5P8 |
| 4677 | 2006 | JQ581645 | Colombia | V | 5P | 5P8 |
| 4678 | 2006 | JQ581646 | Colombia | V | 5P | 5P8 |
| 4679 | 2006 | JQ581647 | Colombia | V | 5P | 5P8 |
| 4680 | 2007 | GQ868567 | Colombia | V | 5P | 5P8 |
| 4681 | 2007 | GQ868568 | Colombia | V | 5P | 5P8 |
| 4682 | 2007 | JQ581617 | Colombia | V | 5P | 5P8 |
| 4683 | 2007 | JQ581618 | Colombia | V | 5P | 5P8 |
| 4684 | 2007 | JQ581622 | Colombia | V | 5P | 5P8 |
| 4685 | 2007 | JQ581626 | Colombia | V | 5P | 5P8 |
| 4686 | 2007 | JQ581627 | Colombia | V | 5P | 5P8 |
| 4687 | 2007 | JQ581629 | Colombia | V | 5P | 5P8 |
| 4688 | 2007 | GQ868566 | Colombia | V | 5P | 5P8 |
| 4689 | 2007 | GQ868569 | Colombia | V | 5P | 5P8 |
| 4690 | 2007 | JQ581633 | Colombia | V | 5P | 5P8 |
| 4691 | 2007 | JQ581637 | Colombia | V | 5P | 5P8 |
| 4692 | 2008 | JQ581619 | Colombia | V | 5P | 5P8 |
| 4693 | 2008 | JQ581620 | Colombia | V | 5P | 5P8 |
| 4694 | 2008 | JQ581623 | Colombia | V | 5P | 5P8 |
| 4695 | 2008 | JQ581624 | Colombia | V | 5P | 5P8 |
| 4696 | 2008 | JQ581625 | Colombia | V | 5P | 5P8 |
| 4697 | 2008 | JQ581628 | Colombia | V | 5P | 5P8 |
| 4698 | 2008 | JQ581630 | Colombia | V | 5P | 5P8 |
| 4699 | 2008 | JQ581631 | Colombia | V | 5P | 5P8 |
| 4700 | 2008 | JQ581634 | Colombia | V | 5P | 5P8 |
| 4701 | 2008 | JQ581636 | Colombia | V | 5P | 5P8 |
| 4702 | 2009 | KX901653 | Colombia | V | 5P | 5P8 |
| 4703 | 2010 | KX901654 | Colombia | V | 5P | 5P8 |
| 4704 | 2004 | FJ639794 | Venezuela | V | 5P | 5P9 |
| 4705 | 2004 | FJ639796 | Venezuela | V | 5P | 5P9 |
| 4706 | 2004 | FJ639797 | Venezuela | V | 5P | 5P9 |
| 4707 | 2004 | FJ639802 | Venezuela | V | 5P | 5P9 |
| 4708 | 2004 | FJ744701 | Venezuela | V | 5P | 5P9 |
| 4709 | 2005 | FJ639808 | Venezuela | V | 5P | 5P9 |
| 4710 | 2005 | FJ639812 | Venezuela | V | 5P | 5P9 |
| 4711 | 2005 | FJ639813 | Venezuela | V | 5P | 5P9 |
| 4712 | 2005 | GQ868562 | Colombia | V | 5P | 5P9 |
| 4713 | 2005 | JN819411 | Venezuela | V | 5P | 5P9 |
| 4714 | 2005 | JN819412 | Venezuela | V | 5P | 5P9 |
| 4715 | 2005 | JQ581613 | Colombia | V | 5P | 5P9 |
| 4716 | 2005 | KF955407 | Venezuela | V | 5P | 5P9 |
| 4717 | 2006 | FJ639818 | Venezuela | V | 5P | 5P9 |
| 4718 | 2006 | FJ639820 | Venezuela | V | 5P | 5P9 |
| 4719 | 2006 | FJ639823 | Venezuela | V | 5P | 5P9 |
| 4720 | 2006 | FJ639824 | Venezuela | V | 5P | 5P9 |
| 4721 | 2006 | HQ332177 | Venezuela | V | 5P | 5P9 |
| 4722 | 2006 | HQ332178 | Venezuela | V | 5P | 5P9 |
| 4723 | 2006 | HQ332180 | Venezuela | V | 5P | 5P9 |
| 4724 | 2006 | JN819405 | Venezuela | V | 5P | 5P9 |
| 4725 | 2006 | JN819413 | Venezuela | V | 5P | 5P9 |
| 4726 | 2006 | JQ581606 | Colombia | V | 5P | 5P9 |
| 4727 | 2006 | JQ581607 | Colombia | V | 5P | 5P9 |
| 4728 | 2006 | JQ581608 | Colombia | V | 5P | 5P9 |
| 4729 | 2006 | JQ581609 | Colombia | V | 5P | 5P9 |
| 4730 | 2006 | JQ581610 | Colombia | V | 5P | 5P9 |
| 4731 | 2006 | JQ581611 | Colombia | V | 5P | 5P9 |
| 4732 | 2006 | JQ581612 | Colombia | V | 5P | 5P9 |
| 4733 | 2006 | JQ581614 | Colombia | V | 5P | 5P9 |
| 4734 | 2006 | JQ581615 | Colombia | V | 5P | 5P9 |
| 4735 | 2006 | JQ581616 | Colombia | V | 5P | 5P9 |
| 4736 | 2006 | KF955413 | Venezuela | V | 5P | 5P9 |
| 4737 | 2007 | EU482609 | Venezuela | V | 5P | 5P9 |
| 4738 | 2007 | FJ639806 | Venezuela | V | 5P | 5P9 |
| 4739 | 2007 | FJ850099 | Venezuela | V | 5P | 5P9 |
| 4740 | 2007 | FJ850100 | Venezuela | V | 5P | 5P9 |
| 4741 | 2007 | FJ850101 | Venezuela | V | 5P | 5P9 |
| 4742 | 2007 | FJ873809 | Venezuela | V | 5P | 5P9 |
| 4743 | 2007 | FJ882579 | Venezuela | V | 5P | 5P9 |
| 4744 | 2007 | GU131842 | Venezuela | V | 5P | 5P9 |
| 4745 | 2007 | HM450102 | Brazil | V | 5P | 5P9 |
| 4746 | 2007 | HM450103 | Brazil | V | 5P | 5P9 |
| 4747 | 2007 | HQ332179 | Venezuela | V | 5P | 5P9 |
| 4748 | 2007 | JN819414 | Venezuela | V | 5P | 5P9 |
| 4749 | 2008 | FJ850093 | Brazil | V | 5P | 5P9 |
| 4750 | 2008 | HM450104 | Brazil | V | 5P | 5P9 |
| 4751 | 2008 | JN415506 | Guyana | V | 5P | 5P9 |
| 4752 | 2008 | JQ581605 | Colombia | V | 5P | 5P9 |
| 4753 | 2008 | KF444789 | Brazil | V | 5P | 5P9 |
| 4754 | 2009 | KF444780 | Brazil | V | 5P | 5P9 |
| 4755 | 2009 | KF444781 | Brazil | V | 5P | 5P9 |
| 4756 | 2009 | KF444782 | Brazil | V | 5P | 5P9 |
| 4757 | 2009 | KF444783 | Brazil | V | 5P | 5P9 |
| 4758 | 2009 | KF444784 | Brazil | V | 5P | 5P9 |
| 4759 | 2009 | KF444785 | Brazil | V | 5P | 5P9 |
| 4760 | 2009 | KF444790 | Brazil | V | 5P | 5P9 |
| 4761 | 2009 | KF444791 | Brazil | V | 5P | 5P9 |
| 4762 | 2010 | KC692517 | Argentina | V | 5P | 5P9 |
| 4763 | 2010 | JN713897 | Brazil | V | 5P | 5P9 |
| 4764 | 2010 | KF444786 | Brazil | V | 5P | 5P9 |
| 4765 | 2010 | KF444787 | Brazil | V | 5P | 5P9 |
| 4766 | 2010 | KF444788 | Brazil | V | 5P | 5P9 |
| 4767 | 2010 | KF444792 | Brazil | V | 5P | 5P9 |
| 4768 | 2011 | KY818070 | Colombia | V | 5P | 5P9 |
| 4769 | 2011 | KU509254 | Venezuela | V | 5P | 5P9 |
| 4770 | 2012 | KY818210 | Colombia | V | 5P | 5P9 |
| 4771 | 2012 | KY818212 | Colombia | V | 5P | 5P9 |
| 4772 | 2012 | KY818218 | Colombia | V | 5P | 5P9 |
| 4773 | 2012 | KY818220 | Colombia | V | 5P | 5P9 |
| 4774 | 2012 | KY818225 | Colombia | V | 5P | 5P9 |
| 4775 | 2012 | KY818226 | Colombia | V | 5P | 5P9 |
| 4776 | 2013 | KY818069 | Colombia | V | 5P | 5P9 |
| 4777 | 2013 | KY818094 | Colombia | V | 5P | 5P9 |
| 4778 | 2013 | KY818099 | Colombia | V | 5P | 5P9 |
| 4779 | 2013 | KY818227 | Colombia | V | 5P | 5P9 |
| 4780 | 2013 | KY818061 | Colombia | V | 5P | 5P9 |
| 4781 | 2013 | KY818062 | Colombia | V | 5P | 5P9 |
| 4782 | 2013 | KY818065 | Colombia | V | 5P | 5P9 |
| 4783 | 2013 | KY818068 | Colombia | V | 5P | 5P9 |
| 4784 | 2013 | KT825054 | Barbados | V | 5P | 5P9 |
| 4785 | 2014 | KY474305 | Ecuador | V | 5P | 5P9 |
| 4786 | 2014 | KY474306 | Ecuador | V | 5P | 5P9 |
| 4787 | 2016 | KX768377 | Argentina | V | 5P | 5P9 |
| 4788 | 2013 | KY818066 | Colombia | V | 5P | 5P10 |
| 4789 | 1997 | GU056029 | Venezuela | V | 5Q | 5Q1 |
| 4790 | 1997 | GU056030 | Venezuela | V | 5Q | 5Q1 |
| 4791 | 1998 | GU056031 | Venezuela | V | 5Q | 5Q1 |
| 4792 | 2000 | GU131832 | Venezuela | V | 5Q | 5Q1 |
| 4793 | 2000 | KF955411 | Venezuela | V | 5Q | 5Q1 |
| 4794 | 2004 | JN819425 | Venezuela | V | 5Q | 5Q1 |
| 4795 | 2005 | FJ639814 | Venezuela | V | 5Q | 5Q1 |
| 4796 | 2005 | FJ810415 | Venezuela | V | 5Q | 5Q1 |
| 4797 | 2005 | JN819410 | Venezuela | V | 5Q | 5Q1 |
| 4798 | 2005 | FJ639811 | Venezuela | V | 5Q | 5Q1 |
| 4799 | 2006 | EU482591 | Puerto_Rico | V | 5Q | 5Q1 |
| 4800 | 2006 | FJ639815 | Venezuela | V | 5Q | 5Q1 |
| 4801 | 2006 | GU131838 | Venezuela | V | 5Q | 5Q1 |
| 4802 | 2006 | GU131839 | Venezuela | V | 5Q | 5Q1 |
| 4803 | 2006 | HQ332181 | Venezuela | V | 5Q | 5Q1 |
| 4804 | 2006 | FJ639819 | Venezuela | V | 5Q | 5Q1 |
| 4805 | 2006 | FJ639821 | Venezuela | V | 5Q | 5Q1 |
| 4806 | 2006 | KF955414 | Venezuela | V | 5Q | 5Q1 |
| 4807 | 2007 | EU482610 | Venezuela | V | 5Q | 5Q1 |
| 4808 | 2007 | EU482611 | Venezuela | V | 5Q | 5Q1 |
| 4809 | 2007 | FJ850102 | Venezuela | V | 5Q | 5Q1 |
| 4810 | 2007 | FJ873810 | Venezuela | V | 5Q | 5Q1 |
| 4811 | 2007 | GQ199877 | Venezuela | V | 5Q | 5Q1 |
| 4812 | 2007 | GU131840 | Venezuela | V | 5Q | 5Q1 |
| 4813 | 2007 | GU131841 | Venezuela | V | 5Q | 5Q1 |
| 4814 | 2007 | KF955441 | Venezuela | V | 5Q | 5Q1 |
| 4815 | 2008 | FJ850103 | Venezuela | V | 5Q | 5Q1 |
| 4816 | 2008 | JN022597 | Martinique | V | 5Q | 5Q1 |
| 4817 | 2008 | JN022598 | Martinique | V | 5Q | 5Q1 |
| 4818 | 2008 | JN022599 | Martinique | V | 5Q | 5Q1 |
| 4819 | 2008 | KU509251 | Venezuela | V | 5Q | 5Q1 |
| 4820 | 2010 | JF969280 | Haiti | V | 5Q | 5Q1 |
| 4821 | 2010 | JF969281 | Haiti | V | 5Q | 5Q1 |
| 4822 | 2010 | JF969282 | Haiti | V | 5Q | 5Q1 |
| 4823 | 2010 | JF969283 | Haiti | V | 5Q | 5Q1 |
| 4824 | 2010 | JF969284 | Haiti | V | 5Q | 5Q1 |
| 4825 | 2010 | JQ045561 | USA | V | 5Q | 5Q1 |
| 4826 | 2010 | JQ045563 | USA | V | 5Q | 5Q1 |
| 4827 | 2010 | JX402207 | Puerto_Rico | V | 5Q | 5Q1 |
| 4828 | 2010 | JX402208 | Puerto_Rico | V | 5Q | 5Q1 |
| 4829 | 2010 | JX402209 | Puerto_Rico | V | 5Q | 5Q1 |
| 4830 | 2010 | JX402210 | Puerto_Rico | V | 5Q | 5Q1 |
| 4831 | 2010 | JX402211 | Puerto_Rico | V | 5Q | 5Q1 |
| 4832 | 2010 | JX402212 | Puerto_Rico | V | 5Q | 5Q1 |
| 4833 | 2010 | JX402213 | Puerto_Rico | V | 5Q | 5Q1 |
| 4834 | 2010 | KU509264 | Haiti | V | 5Q | 5Q1 |
| 4835 | 2010 | KU728186 | Dominica | V | 5Q | 5Q1 |
| 4836 | 2010 | KU728187 | Dominica | V | 5Q | 5Q1 |
| 4837 | 2010 | KU728188 | Dominica | V | 5Q | 5Q1 |
| 4838 | 2010 | KC692516 | Argentina | V | 5Q | 5Q1 |
| 4839 | 2010 | KU509252 | Venezuela | V | 5Q | 5Q1 |
| 4840 | 2011 | KT175076 | China | V | 5Q | 5Q1 |
| 4841 | 2012 | KF444915 | Puerto_Rico | V | 5Q | 5Q1 |
| 4842 | 2012 | KJ189351 | Puerto_Rico | V | 5Q | 5Q1 |
| 4843 | 2012 | KJ189358 | Puerto_Rico | V | 5Q | 5Q1 |
| 4844 | 2012 | KJ189359 | Puerto_Rico | V | 5Q | 5Q1 |
| 4845 | 2012 | KU509249 | Jamaica | V | 5Q | 5Q1 |
| 4846 | 2012 | KY818254 | Puerto_Rico | V | 5Q | 5Q1 |
| 4847 | 2013 | KF444921 | Puerto_Rico | V | 5Q | 5Q1 |
| 4848 | 2013 | KJ415284 | USA | V | 5Q | 5Q1 |
| 4849 | 2013 | KU509295 | Puerto_Rico | V | 5Q | 5Q1 |
| 4850 | 2013 | KY818258 | Puerto_Rico | V | 5Q | 5Q1 |
| 4851 | 2013 | KY882522 | Puerto_Rico | V | 5Q | 5Q1 |
| 4852 | 2010 | JQ045562 | USA | V | 5Q | 5Q2 |
| 4853 | 2010 | KJ189360 | Puerto_Rico | V | 5Q | 5Q2 |
| 4854 | 2010 | KJ189361 | Puerto_Rico | V | 5Q | 5Q2 |
| 4855 | 2010 | KJ189363 | Puerto_Rico | V | 5Q | 5Q2 |
| 4856 | 2010 | KJ189365 | Puerto_Rico | V | 5Q | 5Q2 |
| 4857 | 2010 | KJ189367 | Puerto_Rico | V | 5Q | 5Q2 |
| 4858 | 2010 | KJ189362 | Puerto_Rico | V | 5Q | 5Q2 |
| 4859 | 2010 | KJ189364 | Puerto_Rico | V | 5Q | 5Q2 |
| 4860 | 2010 | KJ189366 | Puerto_Rico | V | 5Q | 5Q2 |
| 4861 | 2012 | KF444910 | Puerto_Rico | V | 5Q | 5Q2 |
| 4862 | 2012 | KF444911 | Puerto_Rico | V | 5Q | 5Q2 |
| 4863 | 2012 | KF444919 | Puerto_Rico | V | 5Q | 5Q2 |
| 4864 | 2012 | KF444922 | Puerto_Rico | V | 5Q | 5Q2 |
| 4865 | 2012 | KJ189352 | Puerto_Rico | V | 5Q | 5Q2 |
| 4866 | 2012 | KJ189355 | Puerto_Rico | V | 5Q | 5Q2 |
| 4867 | 2012 | KJ189356 | Puerto_Rico | V | 5Q | 5Q2 |
| 4868 | 2012 | KJ189357 | Puerto_Rico | V | 5Q | 5Q2 |
| 4869 | 2012 | KY818259 | Puerto_Rico | V | 5Q | 5Q2 |
| 4870 | 2012 | KF444908 | Puerto_Rico | V | 5Q | 5Q2 |
| 4871 | 2012 | KF444909 | Puerto_Rico | V | 5Q | 5Q2 |
| 4872 | 2012 | KF444912 | Puerto_Rico | V | 5Q | 5Q2 |
| 4873 | 2012 | KF444913 | Puerto_Rico | V | 5Q | 5Q2 |
| 4874 | 2012 | KF444914 | Puerto_Rico | V | 5Q | 5Q2 |
| 4875 | 2012 | KF444917 | Puerto_Rico | V | 5Q | 5Q2 |
| 4876 | 2012 | KF809750 | Puerto_Rico | V | 5Q | 5Q2 |
| 4877 | 2012 | KJ189350 | Puerto_Rico | V | 5Q | 5Q2 |
| 4878 | 2012 | KJ189353 | Puerto_Rico | V | 5Q | 5Q2 |
| 4879 | 2012 | KJ189354 | Puerto_Rico | V | 5Q | 5Q2 |
| 4880 | 2012 | KY818257 | Puerto_Rico | V | 5Q | 5Q2 |
| 4881 | 2012 | KF809751 | Puerto_Rico | V | 5Q | 5Q2 |
| 4882 | 2012 | KY882524 | Puerto_Rico | V | 5Q | 5Q2 |
| 4883 | 2013 | KJ676957 | USA | V | 5Q | 5Q2 |
| 4884 | 2013 | KY818244 | Puerto_Rico | V | 5Q | 5Q2 |
| 4885 | 2013 | KY818247 | Puerto_Rico | V | 5Q | 5Q2 |
| 4886 | 2013 | KY818256 | Puerto_Rico | V | 5Q | 5Q2 |
| 4887 | 2013 | KY882514 | Puerto_Rico | V | 5Q | 5Q2 |
| 4888 | 2013 | KF444916 | Puerto_Rico | V | 5Q | 5Q2 |
| 4889 | 2013 | KF444918 | Puerto_Rico | V | 5Q | 5Q2 |
| 4890 | 2013 | KF444920 | Puerto_Rico | V | 5Q | 5Q2 |
| 4891 | 2013 | KF809752 | Puerto_Rico | V | 5Q | 5Q2 |
| 4892 | 2013 | KY818255 | Puerto_Rico | V | 5Q | 5Q2 |
| 4893 | 2013 | KF809753 | Puerto_Rico | V | 5Q | 5Q2 |
| 4894 | 2013 | KY818243 | Puerto_Rico | V | 5Q | 5Q2 |
| 4895 | 2013 | KY818245 | Puerto_Rico | V | 5Q | 5Q2 |
| 4896 | 2013 | KY818246 | Puerto_Rico | V | 5Q | 5Q2 |
| 4897 | 2014 | KT279761 | Haiti | V | 5Q | 5Q2 |
| 4898 | 2009 | KC692495 | Argentina | V | 5R | 5R1 |
| 4899 | 2009 | KC692496 | Argentina | V | 5R | 5R1 |
| 4900 | 2009 | KC692497 | Argentina | V | 5R | 5R1 |
| 4901 | 2009 | KC692498 | Argentina | V | 5R | 5R1 |
| 4902 | 2009 | KC692499 | Argentina | V | 5R | 5R1 |
| 4903 | 2009 | KC692500 | Argentina | V | 5R | 5R1 |
| 4904 | 2009 | KC692501 | Argentina | V | 5R | 5R1 |
| 4905 | 2009 | KC692502 | Argentina | V | 5R | 5R1 |
| 4906 | 2009 | KC692503 | Argentina | V | 5R | 5R1 |
| 4907 | 2009 | KC692504 | Argentina | V | 5R | 5R1 |
| 4908 | 2009 | KC692505 | Argentina | V | 5R | 5R1 |
| 4909 | 2009 | KC692506 | Argentina | V | 5R | 5R1 |
| 4910 | 2009 | KC692507 | Argentina | V | 5R | 5R1 |
| 4911 | 2009 | KC692508 | Argentina | V | 5R | 5R1 |
| 4912 | 2009 | KC692509 | Argentina | V | 5R | 5R1 |
| 4913 | 2009 | KC692510 | Argentina | V | 5R | 5R1 |
| 4914 | 2010 | HQ696612 | Brazil | V | 5R | 5R1 |
| 4915 | 2010 | JQ015184 | Brazil | V | 5R | 5R1 |
| 4916 | 2010 | JQ015185 | Brazil | V | 5R | 5R1 |
| 4917 | 2010 | JX669462 | Brazil | V | 5R | 5R1 |
| 4918 | 2010 | JX669463 | Brazil | V | 5R | 5R1 |
| 4919 | 2010 | JX669466 | Brazil | V | 5R | 5R1 |
| 4920 | 2010 | KC692512 | Argentina | V | 5R | 5R1 |
| 4921 | 2010 | KC692514 | Argentina | V | 5R | 5R1 |
| 4922 | 2010 | KC692515 | Argentina | V | 5R | 5R1 |
| 4923 | 2010 | KF672768 | Brazil | V | 5R | 5R1 |
| 4924 | 2010 | KF672786 | Brazil | V | 5R | 5R1 |
| 4925 | 2011 | KF419418 | Paraguay | V | 5R | 5R1 |
| 4926 | 2011 | KF419419 | Paraguay | V | 5R | 5R1 |
| 4927 | 2011 | KF419420 | Paraguay | V | 5R | 5R1 |
| 4928 | 2011 | KF419421 | Paraguay | V | 5R | 5R1 |
| 4929 | 2011 | KF419422 | Paraguay | V | 5R | 5R1 |
| 4930 | 2011 | KF419423 | Paraguay | V | 5R | 5R1 |
| 4931 | 2011 | KF419424 | Paraguay | V | 5R | 5R1 |
| 4932 | 2011 | KF419425 | Paraguay | V | 5R | 5R1 |
| 4933 | 2011 | KF419426 | Paraguay | V | 5R | 5R1 |
| 4934 | 2011 | KF419427 | Paraguay | V | 5R | 5R1 |
| 4935 | 2011 | KF419428 | Paraguay | V | 5R | 5R1 |
| 4936 | 2011 | KF419429 | Paraguay | V | 5R | 5R1 |
| 4937 | 2011 | KF419430 | Paraguay | V | 5R | 5R1 |
| 4938 | 2011 | KF419431 | Paraguay | V | 5R | 5R1 |
| 4939 | 2011 | KF419432 | Paraguay | V | 5R | 5R1 |
| 4940 | 2011 | KP188540 | Brazil | V | 5R | 5R1 |
| 4941 | 2011 | KP188541 | Brazil | V | 5R | 5R1 |
| 4942 | 2011 | KP188542 | Brazil | V | 5R | 5R1 |
| 4943 | 2011 | JN122281 | Brazil | V | 5R | 5R1 |
| 4944 | 2011 | KF672760 | Brazil | V | 5R | 5R1 |
| 4945 | 2011 | KF672765 | Brazil | V | 5R | 5R1 |
| 4946 | 2011 | KF672766 | Brazil | V | 5R | 5R1 |
| 4947 | 2011 | KF672790 | Brazil | V | 5R | 5R1 |
| 4948 | 2012 | KJ651912 | Brazil | V | 5R | 5R1 |
| 4949 | 2012 | KY818230 | Brazil | V | 5R | 5R1 |
| 4950 | 2012 | KY818231 | Brazil | V | 5R | 5R1 |
| 4951 | 2012 | KY818232 | Brazil | V | 5R | 5R1 |
| 4952 | 2012 | KY818233 | Brazil | V | 5R | 5R1 |
| 4953 | 2012 | KY818288 | Brazil | V | 5R | 5R1 |
| 4954 | 2013 | KP858107 | Brazil | V | 5R | 5R1 |
| 4955 | 2013 | KP903771 | Brazil | V | 5R | 5R1 |
| 4956 | 2013 | KP903772 | Brazil | V | 5R | 5R1 |
| 4957 | 2013 | KP903773 | Brazil | V | 5R | 5R1 |
| 4958 | 2013 | KY818234 | Brazil | V | 5R | 5R1 |
| 4959 | 2013 | KY818235 | Brazil | V | 5R | 5R1 |
| 4960 | 2016 | KX372687 | China | V | 5R | 5R1 |
| 4961 | 2016 | KX768380 | Argentina | V | 5R | 5R1 |
| 4962 | 2016 | KX768381 | Argentina | V | 5R | 5R1 |
| 4963 | 2016 | KX768382 | Argentina | V | 5R | 5R1 |
| 4964 | 2016 | KX768383 | Argentina | V | 5R | 5R1 |
| 4965 | 2016 | KX768384 | Argentina | V | 5R | 5R1 |
| 4966 | 2016 | KX768385 | Argentina | V | 5R | 5R1 |
| 4967 | 2016 | KY283853 | Argentina | V | 5R | 5R1 |
| 4968 | 2016 | KX768378 | Argentina | V | 5R | 5R1 |
| 4969 | 2016 | KX768379 | Argentina | V | 5R | 5R1 |
| 4970 | 2016 | KX768386 | Argentina | V | 5R | 5R1 |
| 4971 | 2016 | KX768387 | Argentina | V | 5R | 5R1 |
| 4972 | 2016 | KX768388 | Argentina | V | 5R | 5R1 |
| 4973 | 2016 | KX768389 | Argentina | V | 5R | 5R1 |
| 4974 | 2016 | KX768390 | Argentina | V | 5R | 5R1 |
| 4975 | 2016 | KX768391 | Argentina | V | 5R | 5R1 |
| 4976 | 2016 | KX768392 | Argentina | V | 5R | 5R1 |
| 4977 | 2016 | KX768393 | Argentina | V | 5R | 5R1 |
| 4978 | 2016 | KX768394 | Argentina | V | 5R | 5R1 |
| 4979 | 2016 | KX768395 | Argentina | V | 5R | 5R1 |
| 4980 | 2016 | KX768396 | Argentina | V | 5R | 5R1 |
| 4981 | 2016 | KX768397 | Argentina | V | 5R | 5R1 |
| 4982 | 2016 | KX768398 | Argentina | V | 5R | 5R1 |
| 4983 | 2016 | KX768399 | Argentina | V | 5R | 5R1 |
| 4984 | 2016 | KX768400 | Argentina | V | 5R | 5R1 |
| 4985 | 2016 | KX768401 | Argentina | V | 5R | 5R1 |
| 4986 | 2016 | KX768402 | Argentina | V | 5R | 5R1 |
| 4987 | 2016 | KX768403 | Argentina | V | 5R | 5R1 |
| 4988 | 2016 | KX768404 | Argentina | V | 5R | 5R1 |
| 4989 | 2016 | KX768405 | Argentina | V | 5R | 5R1 |
| 4990 | 2016 | KX768406 | Argentina | V | 5R | 5R1 |
| 4991 | 2016 | KX768407 | Argentina | V | 5R | 5R1 |
| 4992 | 2016 | KX768408 | Argentina | V | 5R | 5R1 |
| 4993 | 2016 | KX768409 | Argentina | V | 5R | 5R1 |
| 4994 | 2016 | KX768410 | Argentina | V | 5R | 5R1 |
| 4995 | 2016 | KX768411 | Argentina | V | 5R | 5R1 |
| 4996 | 2016 | KX768412 | Argentina | V | 5R | 5R1 |
| 4997 | 2016 | KX768413 | Argentina | V | 5R | 5R1 |
| 4998 | 2016 | KX768414 | Argentina | V | 5R | 5R1 |
| 4999 | 2016 | KX768415 | Argentina | V | 5R | 5R1 |
| 5000 | 2016 | KX768416 | Argentina | V | 5R | 5R1 |
| 5001 | 2016 | KX768417 | Argentina | V | 5R | 5R1 |
| 5002 | 2016 | KX768418 | Argentina | V | 5R | 5R1 |
| 5003 | 2016 | KX768419 | Argentina | V | 5R | 5R1 |
