# Supplementary material for: A unified global genotyping framework of dengue virus serotype-1 for a stratified coordinated surveillance strategy of dengue epidemics
Source: Infect Dis Poverty. 2022 Oct 13;11:107. doi: 10.1186/s40249-022-01024-5 (PMC9556283; doi:10.1186/s40249-022-01024-5)
Supplement: Supplementary file 3 — Additional file 3. Features of the selected 910 E complete sequences for temporal phylogenic reconstruction covering all spatio-temporal distributions and representing all designated subgenotypes and clades in genotypes I, IV, and V from 5003 DENV-1 strains worldwide. [file 40249_2022_1024_MOESM3_ESM.docx]

**Appendix data C1. Features of the selected 910 E complete sequences for temporal phylogenic reconstruction covering all spatio-temporal distributions and representing all designated subgenotypes and clades in genotypes I, IV, and V from 5003 DENV-1 strains worldwide.**

| Isolate | Year | Genebank | Country | level 1 | level 2 | level 3 | Genotype | Subgenotype | Clade |
| --- | --- | --- | --- | --- | --- | --- | --- | --- | --- |
| 1944\|KM204119\|USA.Hawaii | 1944 | KM204119 | USA.Hawaii | 1 | 1 | 1 | I | 1A | 1A1 |
| 1979\|JN029818\|China | 1979 | JN029818 | China | 1 | 1 | 1 | I | 1A | 1A3 |
| 1980\|AY732421\|Thailand | 1980 | AY732421 | Thailand | 1 | 1 | 1 | I | 1A | 1A2 |
| 1980\|AF350498\|China | 1980 | AF350498 | China | 1 | 1 | 1 | I | 1A | 1A3 |
| 1981\|AY732383\|Thailand | 1981 | AY732383 | Thailand | 1 | 1 | 1 | I | 1A | 1A2 |
| 1982\|AY732378\|Thailand | 1982 | AY732378 | Thailand | 1 | 1 | 1 | I | 1A | 1A3 |
| 1983\|AY732390\|Thailand | 1983 | AY732390 | Thailand | 1 | 1 | 1 | I | 1A | 1A3 |
| 1985\|JN638328\|Thailand | 1985 | JN638328 | Thailand | 1 | 1 | 2 | I | 1A | 1A11 |
| 1985\|JN029817\|China | 1985 | JN029817 | China | 1 | 1 | 1 | I | 1A | 1A2 |
| 1986\|JN638336\|Thailand | 1986 | JN638336 | Thailand | 1 | 1 | 2 | I | 1A | 1A12 |
| 1986\|AY732420\|Thailand | 1986 | AY732420 | Thailand | 1 | 1 | 1 | I | 1A | 1A2 |
| 1987\|AF425628\|China | 1987 | AF425628 | China | 1 | 1 | 2 | I | 1A | 1A12 |
| 1987\|AY732425\|Thailand | 1987 | AY732425 | Thailand | 1 | 1 | 2 | I | 1A | 1A12 |
| 1987\|JN638341\|Thailand | 1987 | JN638341 | Thailand | 1 | 1 | 2 | I | 1A | 1A9 |
| 1988\|AY732437\|Thailand | 1988 | AY732437 | Thailand | 1 | 1 | 2 | I | 1A | 1A12 |
| 1989\|AY732410\|Thailand | 1989 | AY732410 | Thailand | 1 | 1 | 2 | I | 1A | 1A10 |
| 1989\|AY732433\|Thailand | 1989 | AY732433 | Thailand | 1 | 1 | 2 | I | 1A | 1A12 |
| 1989\|AY732388\|Thailand | 1989 | AY732388 | Thailand | 1 | 1 | 2 | I | 1A | 1A4 |
| 1990\|JN638342\|Thailand | 1990 | JN638342 | Thailand | 1 | 1 | 2 | I | 1A | 1A11 |
| 1990\|AY732442\|Thailand | 1990 | AY732442 | Thailand | 1 | 1 | 2 | I | 1A | 1A12 |
| 1990\|AY732448\|Thailand | 1990 | AY732448 | Thailand | 1 | 1 | 2 | I | 1A | 1A4 |
| 1991\|AY732478\|Thailand | 1991 | AY732478 | Thailand | 1 | 1 | 2 | I | 1A | 1A12 |
| 1992\|JN638338\|Thailand | 1992 | JN638338 | Thailand | 1 | 1 | 2 | I | 1A | 1A12 |
| 1992\|AY732402\|Thailand | 1992 | AY732402 | Thailand | 1 | 1 | 2 | I | 1A | 1A4 |
| 1992\|AY732395\|Thailand | 1992 | AY732395 | Thailand | 1 | 1 | 2 | I | 1A | 1A6 |
| 1993\|AY732465\|Thailand | 1993 | AY732465 | Thailand | 1 | 1 | 2 | I | 1A | 1A12 |
| 1993\|AY732399\|Thailand | 1993 | AY732399 | Thailand | 1 | 1 | 2 | I | 1A | 1A4 |
| 1994\|AY732468\|Thailand | 1994 | AY732468 | Thailand | 1 | 1 | 2 | I | 1A | 1A12 |
| 1994\|AY732456\|Thailand | 1994 | AY732456 | Thailand | 1 | 1 | 2 | I | 1A | 1A4 |
| 1995\|JN638344\|Thailand | 1995 | JN638344 | Thailand | 1 | 1 | 2 | I | 1A | 1A12 |
| 1996\|AB003090\|Laos | 1996 | AB003090 | Laos | 1 | 1 | 2 | I | 1A | 1A5 |
| 1998\|JQ317713\|China | 1998 | JQ317713 | China | 1 | 1 | 1 | I | 1A | 1A2 |
| 1998\|AF298808\|Djibouti | 1998 | AF298808 | Djibouti | 1 | 3 | 30 | I | 1A | 1A20 |
| 1998\|AY726555\|Myanmar | 1998 | AY726555 | Myanmar | 1 | 1 | 2 | I | 1A | 1A8 |
| 1999\|AY618878\|Myanmar | 1999 | AY618878 | Myanmar | 1 | 2 | 8 | I | 1A | 1A7 |
| 2000\|AY618877\|Myanmar | 2000 | AY618877 | Myanmar | 1 | 2 | 8 | I | 1A | 1A7 |
| 2001\|DQ265013\|Myanmar | 2001 | DQ265013 | Myanmar | 1 | 2 | 8 | I | 1A | 1A7 |
| 2001\|AY618210\|Myanmar | 2001 | AY618210 | Myanmar | 1 | 1 | 2 | I | 1A | 1A8 |
| 2002\|AY726552\|Myanmar | 2002 | AY726552 | Myanmar | 1 | 2 | 8 | I | 1A | 1A7 |
| 2006\|AM746212\|Saudi_Arabia | 2006 | AM746212 | Saudi_Arabia | 1 | 3 | 29 | I | 1A | 1A13 |
| 2006\|AM746213\|Saudi_Arabia | 2006 | AM746213 | Saudi_Arabia | 1 | 3 | 29 | I | 1A | 1A14 |
| 2010\|KU509258\|Eritrea | 2010 | KU509258 | Eritrea | 1 | 3 | 30 | I | 1A | 1A19 |
| 2011\|KJ649286\|Saudi_Arabia | 2011 | KJ649286 | Saudi_Arabia | 1 | 3 | 29 | I | 1A | 1A14 |
| 2011\|KC848578\|Somalia | 2011 | KC848578 | Somalia | 1 | 3 | 31 | I | 1A | 1A15 |
| 2011\|KC848579\|Somalia | 2011 | KC848579 | Somalia | 1 | 3 | 31 | I | 1A | 1A16 |
| 2011\|KC848580\|Somalia | 2011 | KC848580 | Somalia | 1 | 3 | 31 | I | 1A | 1A17 |
| 1990\|AY732441\|Thailand | 1990 | AY732441 | Thailand | 2 | 4 | 3 | I | 1B | 1B1 |
| 1991\|JQ317727\|China | 1991 | JQ317727 | China | 2 | 4 | 3 | I | 1B | 1B1 |
| 1991\|AY732413\|Thailand | 1991 | AY732413 | Thailand | 2 | 5 | 5 | I | 1B | 1B12 |
| 1992\|JN638343\|Thailand | 1992 | JN638343 | Thailand | 2 | 4 | 3 | I | 1B | 1B1 |
| 1993\|AY732461\|Thailand | 1993 | AY732461 | Thailand | 2 | 4 | 3 | I | 1B | 1B1 |
| 1993\|AY732415\|Thailand | 1993 | AY732415 | Thailand | 2 | 4 | 4 | I | 1B | 1B4 |
| 1994\|AY732393\|Thailand | 1994 | AY732393 | Thailand | 2 | 4 | 3 | I | 1B | 1B1 |
| 1994\|AB608789\|China | 1994 | AB608789 | China | 2 | 5 | 5 | I | 1B | 1B10 |
| 1994\|AY732431\|Thailand | 1994 | AY732431 | Thailand | 2 | 5 | 6 | I | 1B | 1B16 |
| 1995\|AY732404\|Thailand | 1995 | AY732404 | Thailand | 2 | 4 | 3 | I | 1B | 1B1 |
| 1995\|AY376738\|China | 1995 | AY376738 | China | 2 | 5 | 5 | I | 1B | 1B12 |
| 1995\|AY732430\|Thailand | 1995 | AY732430 | Thailand | 2 | 5 | 5 | I | 1B | 1B12 |
| 1995\|JN638339\|Thailand | 1995 | JN638339 | Thailand | 2 | 4 | 4 | I | 1B | 1B4 |
| 1996\|AY732423\|Thailand | 1996 | AY732423 | Thailand | 2 | 4 | 3 | I | 1B | 1B1 |
| 1996\|AY732422\|Thailand | 1996 | AY732422 | Thailand | 2 | 5 | 5 | I | 1B | 1B12 |
| 1996\|AY732455\|Thailand | 1996 | AY732455 | Thailand | 2 | 4 | 4 | I | 1B | 1B4 |
| 1997\|JN638330\|Thailand | 1997 | JN638330 | Thailand | 2 | 5 | 5 | I | 1B | 1B12 |
| 1997\|AY376737\|China | 1997 | AY376737 | China | 2 | 5 | 5 | I | 1B | 1B15 |
| 1997\|JQ317731\|China | 1997 | JQ317731 | China | 2 | 4 | 3 | I | 1B | 1B2 |
| 1997\|JN638340\|Thailand | 1997 | JN638340 | Thailand | 2 | 4 | 4 | I | 1B | 1B4 |
| 1997\|AY732459\|Thailand | 1997 | AY732459 | Thailand | 2 | 4 | 4 | I | 1B | 1B5 |
| 1998\|KC861971\|Vietnam | 1998 | KC861971 | Vietnam | 2 | 5 | 5 | I | 1B | 1B11 |
| 1998\|AY588273\|Myanmar | 1998 | AY588273 | Myanmar | 2 | 5 | 5 | I | 1B | 1B12 |
| 1998\|EF508198\|China | 1998 | EF508198 | China | 2 | 5 | 5 | I | 1B | 1B12 |
| 1998\|JQ317710\|China | 1998 | JQ317710 | China | 2 | 5 | 5 | I | 1B | 1B14 |
| 1998\|KY496856\|China | 1998 | KY496856 | China | 2 | 5 | 6 | I | 1B | 1B16 |
| 1998\|AF309641\|Cambodia | 1998 | AF309641 | Cambodia | 2 | 5 | 6 | I | 1B | 1B16 |
| 1998\|AY732454\|Thailand | 1998 | AY732454 | Thailand | 2 | 4 | 4 | I | 1B | 1B5 |
| 1999\|AY732400\|Thailand | 1999 | AY732400 | Thailand | 2 | 5 | 5 | I | 1B | 1B12 |
| 1999\|EF508199\|China | 1999 | EF508199 | China | 2 | 5 | 5 | I | 1B | 1B12 |
| 1999\|KC861947\|Vietnam | 1999 | KC861947 | Vietnam | 2 | 5 | 5 | I | 1B | 1B12 |
| 1999\|AY732443\|Thailand | 1999 | AY732443 | Thailand | 2 | 4 | 3 | I | 1B | 1B3 |
| 1999\|AY732470\|Thailand | 1999 | AY732470 | Thailand | 2 | 4 | 4 | I | 1B | 1B4 |
| 2000\|AY732452\|Thailand | 2000 | AY732452 | Thailand | 2 | 5 | 5 | I | 1B | 1B12 |
| 2000\|AY732460\|Thailand | 2000 | AY732460 | Thailand | 2 | 5 | 5 | I | 1B | 1B13 |
| 2000\|AY732409\|Thailand | 2000 | AY732409 | Thailand | 2 | 5 | 6 | I | 1B | 1B16 |
| 2000\|GQ868637\|Cambodia | 2000 | GQ868637 | Cambodia | 2 | 6 | 15 | I | 1B | 1B6 |
| 2001\|AY732462\|Thailand | 2001 | AY732462 | Thailand | 2 | 5 | 5 | I | 1B | 1B12 |
| 2001\|JN415525\|Thailand | 2001 | JN415525 | Thailand | 2 | 5 | 6 | I | 1B | 1B16 |
| 2001\|AY732464\|Thailand | 2001 | AY732464 | Thailand | 2 | 4 | 4 | I | 1B | 1B4 |
| 2001\|JN819423\|Cambodia | 2001 | JN819423 | Cambodia | 2 | 6 | 15 | I | 1B | 1B6 |
| 2001\|KF955406\|Cambodia | 2001 | KF955406 | Cambodia | 2 | 6 | 15 | I | 1B | 1B7 |
| 2001\|FJ639673\|Cambodia | 2001 | FJ639673 | Cambodia | 2 | 6 | 16 | I | 1B | 1B8 |
| 2002\|AY732403\|Thailand | 2002 | AY732403 | Thailand | 2 | 5 | 6 | I | 1B | 1B16 |
| 2003\|FJ639678\|Cambodia | 2003 | FJ639678 | Cambodia | 2 | 6 | 15 | I | 1B | 1B6 |
| 2003\|JN638323\|Thailand | 2003 | JN638323 | Thailand | 2 | 6 | 15 | I | 1B | 1B6 |
| 2003\|FJ850069\|Cambodia | 2003 | FJ850069 | Cambodia | 2 | 6 | 15 | I | 1B | 1B7 |
| 2003\|JN376779\|Vietnam | 2003 | JN376779 | Vietnam | 2 | 6 | 15 | I | 1B | 1B7 |
| 2006\|FJ898424\|Vietnam | 2006 | FJ898424 | Vietnam | 2 | 6 | 16 | I | 1B | 1B8 |
| 2006\|JQ993176\|Thailand | 2006 | JQ993176 | Thailand | 2 | 7 | 49 | I | 1B | 1B9 |
| 2007\|JQ993151\|Thailand | 2007 | JQ993151 | Thailand | 2 | 5 | 6 | I | 1B | 1B16 |
| 2007\|EU677176\|Vietnam | 2007 | EU677176 | Vietnam | 2 | 6 | 16 | I | 1B | 1B8 |
| 2007\|JQ993109\|Thailand | 2007 | JQ993109 | Thailand | 2 | 7 | 48 | I | 1B | 1B9 |
| 2008\|KC172835\|Laos | 2008 | KC172835 | Laos | 2 | 5 | 6 | I | 1B | 1B16 |
| 2008\|KU509260\|Cambodia | 2008 | KU509260 | Cambodia | 2 | 7 | 48 | I | 1B | 1B9 |
| 2008\|KU509262\|Thailand | 2008 | KU509262 | Thailand | 2 | 7 | 48 | I | 1B | 1B9 |
| 2009\|KY849745\|Laos | 2009 | KY849745 | Laos | 2 | 5 | 6 | I | 1B | 1B16 |
| 2009\|KC172832\|Laos | 2009 | KC172832 | Laos | 2 | 7 | 48 | I | 1B | 1B9 |
| 2009\|KU509256\|Thailand | 2009 | KU509256 | Thailand | 2 | 7 | 48 | I | 1B | 1B9 |
| 2010\|HG316481\|Thailand | 2010 | HG316481 | Thailand | 2 | 7 | 48 | I | 1B | 1B9 |
| 2002\|KC861979\|Vietnam | 2002 | KC861979 | Vietnam | 9 | 23 | 26 | I | 1C | 1C1 |
| 2003\|EU482476\|Vietnam | 2003 | EU482476 | Vietnam | 9 | 23 | 26 | I | 1C | 1C1 |
| 2006\|FJ898425\|Vietnam | 2006 | FJ898425 | Vietnam | 9 | 24 | 38 | I | 1C | 1C1 |
| 2007\|FJ024456\|Vietnam | 2007 | FJ024456 | Vietnam | 9 | 23 | 27 | I | 1C | 1C1 |
| 2008\|GQ868636\|Cambodia | 2008 | GQ868636 | Cambodia | 9 | 23 | 27 | I | 1C | 1C1 |
| 2008\|JF967849\|Vietnam | 2008 | JF967849 | Vietnam | 9 | 24 | 38 | I | 1C | 1C1 |
| 2009\|KC861953\|Vietnam | 2009 | KC861953 | Vietnam | 9 | 24 | 37 | I | 1C | 1C1 |
| 2010\|JF967943\|Vietnam | 2010 | JF967943 | Vietnam | 9 | 24 | 37 | I | 1C | 1C1 |
| 2011\|JX093684\|Vietnam | 2011 | JX093684 | Vietnam | 9 | 24 | 37 | I | 1C | 1C1 |
| 1998\|KC861961\|Vietnam | 1998 | KC861961 | Vietnam | 3 | 8 | 7 | I | 1D | 1D4 |
| 1999\|AY732439\|Thailand | 1999 | AY732439 | Thailand | 3 | 9 | 11 | I | 1D | 1D1 |
| 2000\|AY732469\|Thailand | 2000 | AY732469 | Thailand | 3 | 9 | 11 | I | 1D | 1D1 |
| 2001\|AY732467\|Thailand | 2001 | AY732467 | Thailand | 3 | 9 | 11 | I | 1D | 1D1 |
| 2002\|EU069599\|Singapore | 2002 | EU069599 | Singapore | 3 | 9 | 11 | I | 1D | 1D1 |
| 2002\|JN376778\|Vietnam | 2002 | JN376778 | Vietnam | 3 | 9 | 11 | I | 1D | 1D1 |
| 2002\|FJ639674\|Cambodia | 2002 | FJ639674 | Cambodia | 3 | 9 | 12 | I | 1D | 1D3 |
| 2003\|GQ868618\|Cambodia | 2003 | GQ868618 | Cambodia | 3 | 9 | 11 | I | 1D | 1D1 |
| 2003\|KC861964\|Vietnam | 2003 | KC861964 | Vietnam | 3 | 9 | 11 | I | 1D | 1D1 |
| 2003\|KC861920\|Vietnam | 2003 | KC861920 | Vietnam | 3 | 9 | 12 | I | 1D | 1D3 |
| 2006\|HM181939\|Cambodia | 2006 | HM181939 | Cambodia | 3 | 9 | 11 | I | 1D | 1D1 |
| 2006\|EU660402\|Vietnam | 2006 | EU660402 | Vietnam | 3 | 10 | 47 | I | 1D | 1D2 |
| 2006\|GQ199840\|Vietnam | 2006 | GQ199840 | Vietnam | 3 | 9 | 12 | I | 1D | 1D3 |
| 2006\|KC861975\|Vietnam | 2006 | KC861975 | Vietnam | 3 | 8 | 7 | I | 1D | 1D4 |
| 2007\|FJ882559\|Vietnam | 2007 | FJ882559 | Vietnam | 3 | 9 | 11 | I | 1D | 1D1 |
| 2007\|HM181945\|Cambodia | 2007 | HM181945 | Cambodia | 3 | 9 | 11 | I | 1D | 1D1 |
| 2007\|GQ199811\|Vietnam | 2007 | GQ199811 | Vietnam | 3 | 10 | 46 | I | 1D | 1D2 |
| 2007\|HQ588128\|Vietnam | 2007 | HQ588128 | Vietnam | 3 | 9 | 12 | I | 1D | 1D3 |
| 2008\|KC182104\|Laos | 2008 | KC182104 | Laos | 3 | 9 | 11 | I | 1D | 1D1 |
| 2008\|GU131756\|Vietnam | 2008 | GU131756 | Vietnam | 3 | 10 | 46 | I | 1D | 1D2 |
| 2008\|FJ410246\|Vietnam | 2008 | FJ410246 | Vietnam | 3 | 9 | 12 | I | 1D | 1D3 |
| 2008\|JQ403516\|China | 2008 | JQ403516 | China | 3 | 8 | 7 | I | 1D | 1D4 |
| 2008\|KC861938\|Vietnam | 2008 | KC861938 | Vietnam | 3 | 8 | 7 | I | 1D | 1D4 |
| 2009\|JF967861\|Vietnam | 2009 | JF967861 | Vietnam | 3 | 10 | 46 | I | 1D | 1D2 |
| 2009\|JF967871\|Vietnam | 2009 | JF967871 | Vietnam | 3 | 9 | 12 | I | 1D | 1D3 |
| 2009\|JF967851\|Vietnam | 2009 | JF967851 | Vietnam | 3 | 8 | 7 | I | 1D | 1D4 |
| 2010\|JF967953\|Vietnam | 2010 | JF967953 | Vietnam | 3 | 9 | 12 | I | 1D | 1D3 |
| 2011\|JX093683\|Vietnam | 2011 | JX093683 | Vietnam | 3 | 10 | 46 | I | 1D | 1D2 |
| 2011\|JX093712\|Vietnam | 2011 | JX093712 | Vietnam | 3 | 9 | 12 | I | 1D | 1D3 |
| 2011\|KC136240\|China | 2011 | KC136240 | China | 3 | 9 | 12 | I | 1D | 1D3 |
| 2001\|AB111071\|Cambodia | 2001 | AB111071 | Cambodia | 6 | 14 | 17 | I | 1E | 1E1 |
| 2002\|JQ317746\|China | 2002 | JQ317746 | China | 6 | 14 | 17 | I | 1E | 1E1 |
| 2006\|EF508203\|China | 2006 | EF508203 | China | 6 | 14 | 17 | I | 1E | 1E1 |
| 2006\|FJ196843\|China | 2006 | FJ196843 | China | 6 | 14 | 17 | I | 1E | 1E1 |
| 2006\|FJ882522\|Vietnam | 2006 | FJ882522 | Vietnam | 6 | 14 | 17 | I | 1E | 1E1 |
| 2008\|JN196566\|Singapore | 2008 | JN196566 | Singapore | 6 | 14 | 17 | I | 1E | 1E1 |
| 2008\|JQ403518\|China | 2008 | JQ403518 | China | 6 | 14 | 17 | I | 1E | 1E1 |
| 2008\|JF967833\|Vietnam | 2008 | JF967833 | Vietnam | 6 | 15 | 53 | I | 1E | 1E1 |
| 2010\|JF960221\|Singapore | 2010 | JF960221 | Singapore | 6 | 15 | 53 | I | 1E | 1E1 |
| 2012\|KC854415\|New_Caledonia | 2012 | KC854415 | New_Caledonia | 6 | 15 | 55 | I | 1E | 1E1 |
| 2012\|KU570095\|China | 2012 | KU570095 | China | 6 | 15 | 55 | I | 1E | 1E1 |
| 2012\|KY818248\|Vietnam | 2012 | KY818248 | Vietnam | 6 | 15 | 55 | I | 1E | 1E1 |
| 2013\|KJ806952\|Singapore | 2013 | KJ806952 | Singapore | 6 | 15 | 54 | I | 1E | 1E1 |
| 2013\|KT825012\|French_Polynesia | 2013 | KT825012 | French_Polynesia | 6 | 15 | 55 | I | 1E | 1E1 |
| 2013\|KY971717\|Vietnam | 2013 | KY971717 | Vietnam | 6 | 15 | 54 | I | 1E | 1E1 |
| 2014\|KT825033\|Vietnam | 2014 | KT825033 | Vietnam | 6 | 15 | 55 | I | 1E | 1E1 |
| 2014\|KU509313\|Singapore | 2014 | KU509313 | Singapore | 6 | 15 | 54 | I | 1E | 1E1 |
| 2014\|KX685338\|French_Polynesia | 2014 | KX685338 | French_Polynesia | 6 | 15 | 55 | I | 1E | 1E1 |
| 2015\|KT825035\|French_Polynesia | 2015 | KT825035 | French_Polynesia | 6 | 15 | 55 | I | 1E | 1E1 |
| 2015\|KY971719\|Vietnam | 2015 | KY971719 | Vietnam | 6 | 15 | 54 | I | 1E | 1E1 |
| 2016\|KY495793\|Vietnam | 2016 | KY495793 | Vietnam | 6 | 15 | 53 | I | 1E | 1E1 |
| 2003\|FJ639679\|Cambodia | 2003 | FJ639679 | Cambodia | 10 | 25 | 28 | I | 1F | 1F1 |
| 2006\|HM181941\|Cambodia | 2006 | HM181941 | Cambodia | 10 | 25 | 28 | I | 1F | 1F1 |
| 2006\|JF269174\|Vietnam | 2006 | JF269174 | Vietnam | 10 | 25 | 28 | I | 1F | 1F1 |
| 2007\|GQ199825\|Vietnam | 2007 | GQ199825 | Vietnam | 10 | 25 | 28 | I | 1F | 1F1 |
| 2007\|KF955444\|Cambodia | 2007 | KF955444 | Cambodia | 10 | 25 | 28 | I | 1F | 1F1 |
| 2008\|JF937651\|Cambodia | 2008 | JF937651 | Cambodia | 10 | 25 | 28 | I | 1F | 1F1 |
| 2008\|KY971686\|Vietnam | 2008 | KY971686 | Vietnam | 10 | 25 | 28 | I | 1F | 1F1 |
| 2009\|GU131895\|Cambodia | 2009 | GU131895 | Cambodia | 10 | 25 | 28 | I | 1F | 1F1 |
| 2009\|KY849720\|Laos | 2009 | KY849720 | Laos | 10 | 25 | 28 | I | 1F | 1F1 |
| 2009\|KY971700\|Vietnam | 2009 | KY971700 | Vietnam | 10 | 25 | 28 | I | 1F | 1F1 |
| 2010\|JF967924\|Cambodia | 2010 | JF967924 | Cambodia | 10 | 25 | 28 | I | 1F | 1F1 |
| 2010\|KY849743\|Laos | 2010 | KY849743 | Laos | 10 | 25 | 28 | I | 1F | 1F1 |
| 2010\|KY971701\|Vietnam | 2010 | KY971701 | Vietnam | 10 | 25 | 28 | I | 1F | 1F1 |
| 2011\|JX093708\|Vietnam | 2011 | JX093708 | Vietnam | 10 | 25 | 28 | I | 1F | 1F1 |
| 2011\|KT175107\|Cambodia | 2011 | KT175107 | Cambodia | 10 | 25 | 28 | I | 1F | 1F1 |
| 2012\|KT175078\|China | 2012 | KT175078 | China | 10 | 25 | 28 | I | 1F | 1F1 |
| 2012\|KT824985\|Cambodia | 2012 | KT824985 | Cambodia | 10 | 25 | 28 | I | 1F | 1F1 |
| 2012\|KT824990\|Papua_New_Guinea | 2012 | KT824990 | Papua_New_Guinea | 10 | 25 | 28 | I | 1F | 1F1 |
| 2012\|KX380798\|Singapore | 2012 | KX380798 | Singapore | 10 | 25 | 28 | I | 1F | 1F1 |
| 2013\|KF887994\|Thailand | 2013 | KF887994 | Thailand | 10 | 25 | 28 | I | 1F | 1F1 |
| 2013\|KT825017\|Vietnam | 2013 | KT825017 | Vietnam | 10 | 25 | 28 | I | 1F | 1F1 |
| 2013\|KT825055\|Cambodia | 2013 | KT825055 | Cambodia | 10 | 25 | 28 | I | 1F | 1F1 |
| 1999\|AY620950\|Myanmar | 1999 | AY620950 | Myanmar | 4 | 11 | 9 | I | 1G | 1G7 |
| 2000\|DQ265095\|Myanmar | 2000 | DQ265095 | Myanmar | 4 | 11 | 9 | I | 1G | 1G9 |
| 2001\|DQ264947\|Myanmar | 2001 | DQ264947 | Myanmar | 4 | 12 | 21 | I | 1G | 1G1 |
| 2001\|EU117309\|Thailand | 2001 | EU117309 | Thailand | 4 | 12 | 21 | I | 1G | 1G1 |
| 2001\|DQ265005\|Myanmar | 2001 | DQ265005 | Myanmar | 4 | 11 | 10 | I | 1G | 1G10 |
| 2001\|DQ264881\|Myanmar | 2001 | DQ264881 | Myanmar | 4 | 11 | 9 | I | 1G | 1G6 |
| 2001\|DQ264917\|Myanmar | 2001 | DQ264917 | Myanmar | 4 | 11 | 9 | I | 1G | 1G7 |
| 2001\|DQ264927\|Myanmar | 2001 | DQ264927 | Myanmar | 4 | 11 | 9 | I | 1G | 1G8 |
| 2001\|AY620948\|Myanmar | 2001 | AY620948 | Myanmar | 4 | 11 | 9 | I | 1G | 1G9 |
| 2002\|AY732386\|Thailand | 2002 | AY732386 | Thailand | 4 | 12 | 21 | I | 1G | 1G1 |
| 2002\|DQ265156\|Myanmar | 2002 | DQ265156 | Myanmar | 4 | 11 | 10 | I | 1G | 1G10 |
| 2002\|DQ264981\|Myanmar | 2002 | DQ264981 | Myanmar | 4 | 11 | 9 | I | 1G | 1G9 |
| 2007\|KT175102\|Myanmar | 2007 | KT175102 | Myanmar | 4 | 12 | 22 | I | 1G | 1G3 |
| 2008\|JF967840\|Myanmar | 2008 | JF967840 | Myanmar | 4 | 12 | 21 | I | 1G | 1G1 |
| 2008\|JF967848\|Myanmar | 2008 | JF967848 | Myanmar | 4 | 12 | 22 | I | 1G | 1G3 |
| 2008\|JF967822\|Thailand | 2008 | JF967822 | Thailand | 4 | 12 | 22 | I | 1G | 1G3 |
| 2009\|KT373893\|Thailand | 2009 | KT373893 | Thailand | 4 | 12 | 23 | I | 1G | 1G5 |
| 2011\|KT175077\|China | 2011 | KT175077 | China | 4 | 12 | 22 | I | 1G | 1G4 |
| 2011\|KT175103\|Myanmar | 2011 | KT175103 | Myanmar | 4 | 12 | 23 | I | 1G | 1G5 |
| 2013\|KR051909\|Myanmar | 2013 | KR051909 | Myanmar | 4 | 12 | 22 | I | 1G | 1G4 |
| 2013\|KR051930\|Myanmar | 2013 | KR051930 | Myanmar | 4 | 12 | 23 | I | 1G | 1G5 |
| 2013\|KT175079\|China | 2013 | KT175079 | China | 4 | 12 | 23 | I | 1G | 1G5 |
| 2013\|KU509290\|Thailand | 2013 | KU509290 | Thailand | 4 | 12 | 23 | I | 1G | 1G5 |
| 2014\|KU509294\|Thailand | 2014 | KU509294 | Thailand | 4 | 12 | 23 | I | 1G | 1G5 |
| 2014\|KX620454\|China | 2014 | KX620454 | China | 4 | 12 | 23 | I | 1G | 1G5 |
| 2014\|KY038895\|China | 2014 | KY038895 | China | 4 | 12 | 22 | I | 1G | 1G2 |
| 2014\|LC038146\|Myanmar | 2014 | LC038146 | Myanmar | 4 | 12 | 23 | I | 1G | 1G5 |
| 2015\|KX056467\|China | 2015 | KX056467 | China | 4 | 12 | 23 | I | 1G | 1G5 |
| 2015\|KX357943\|Myanmar | 2015 | KX357943 | Myanmar | 4 | 12 | 23 | I | 1G | 1G5 |
| 2015\|KX357952\|Myanmar | 2015 | KX357952 | Myanmar | 4 | 12 | 22 | I | 1G | 1G4 |
| 2015\|KX357962\|Myanmar | 2015 | KX357962 | Myanmar | 4 | 12 | 22 | I | 1G | 1G3 |
| 2015\|KY234170\|Thailand | 2015 | KY234170 | Thailand | 4 | 12 | 22 | I | 1G | 1G2 |
| 2001\|FJ850068\|Thailand | 2001 | FJ850068 | Thailand | 7 | 16 | 19 | I | 1H | 1H1 |
| 2001\|JQ317752\|China | 2001 | JQ317752 | China | 7 | 16 | 19 | I | 1H | 1H1 |
| 2003\|EU448393\|Thailand | 2003 | EU448393 | Thailand | 7 | 16 | 19 | I | 1H | 1H1 |
| 2006\|JQ993193\|Thailand | 2006 | JQ993193 | Thailand | 7 | 16 | 19 | I | 1H | 1H1 |
| 2006\|EU482811\|Vietnam | 2006 | EU482811 | Vietnam | 7 | 16 | 20 | I | 1H | 1H2 |
| 2006\|FJ196844\|China | 2006 | FJ196844 | China | 7 | 17 | 39 | I | 1H | 1H4 |
| 2006\|JQ993192\|Thailand | 2006 | JQ993192 | Thailand | 7 | 17 | 39 | I | 1H | 1H4 |
| 2006\|JQ317744\|China | 2006 | JQ317744 | China | 7 | 18 | 42 | I | 1H | 1H5 |
| 2007\|EU448395\|Malaysia | 2007 | EU448395 | Malaysia | 7 | 16 | 19 | I | 1H | 1H1 |
| 2007\|GQ357689\|Singapore | 2007 | GQ357689 | Singapore | 7 | 16 | 19 | I | 1H | 1H1 |
| 2007\|JQ993148\|Thailand | 2007 | JQ993148 | Thailand | 7 | 16 | 19 | I | 1H | 1H1 |
| 2007\|FJ461328\|Vietnam | 2007 | FJ461328 | Vietnam | 7 | 16 | 20 | I | 1H | 1H2 |
| 2007\|HM181952\|Cambodia | 2007 | HM181952 | Cambodia | 7 | 16 | 20 | I | 1H | 1H2 |
| 2007\|FJ687474\|Thailand | 2007 | FJ687474 | Thailand | 7 | 19 | 56 | I | 1H | 1H3 |
| 2007\|EU280167\|China | 2007 | EU280167 | China | 7 | 17 | 39 | I | 1H | 1H4 |
| 2007\|JQ993108\|Thailand | 2007 | JQ993108 | Thailand | 7 | 17 | 39 | I | 1H | 1H4 |
| 2008\|JQ403517\|China | 2008 | JQ403517 | China | 7 | 16 | 19 | I | 1H | 1H1 |
| 2008\|KC172834\|Laos | 2008 | KC172834 | Laos | 7 | 16 | 19 | I | 1H | 1H1 |
| 2008\|KY971689\|Vietnam | 2008 | KY971689 | Vietnam | 7 | 16 | 20 | I | 1H | 1H2 |
| 2008\|KY849706\|Laos | 2008 | KY849706 | Laos | 7 | 19 | 56 | I | 1H | 1H3 |
| 2008\|JF967810\|Myanmar | 2008 | JF967810 | Myanmar | 7 | 17 | 39 | I | 1H | 1H4 |
| 2008\|JN415527\|Thailand | 2008 | JN415527 | Thailand | 7 | 17 | 39 | I | 1H | 1H4 |
| 2009\|KY849746\|Laos | 2009 | KY849746 | Laos | 7 | 19 | 56 | I | 1H | 1H3 |
| 2009\|JF967879\|Thailand | 2009 | JF967879 | Thailand | 7 | 17 | 39 | I | 1H | 1H4 |
| 2009\|JF960219\|Singapore | 2009 | JF960219 | Singapore | 7 | 18 | 42 | I | 1H | 1H5 |
| 2009\|JN054256\|Sri_Lanka | 2009 | JN054256 | Sri_Lanka | 7 | 18 | 42 | I | 1H | 1H5 |
| 2009\|KT373894\|Thailand | 2009 | KT373894 | Thailand | 7 | 18 | 42 | I | 1H | 1H5 |
| 2010\|KU570094\|China | 2010 | KU570094 | China | 7 | 19 | 56 | I | 1H | 1H3 |
| 2010\|KY849747\|Laos | 2010 | KY849747 | Laos | 7 | 19 | 56 | I | 1H | 1H3 |
| 2010\|JF960220\|Singapore | 2010 | JF960220 | Singapore | 7 | 18 | 42 | I | 1H | 1H5 |
| 2010\|JN054255\|Sri_Lanka | 2010 | JN054255 | Sri_Lanka | 7 | 18 | 42 | I | 1H | 1H5 |
| 2011\|JQ317754\|China | 2011 | JQ317754 | China | 7 | 16 | 19 | I | 1H | 1H1 |
| 2011\|KF926700\|Laos | 2011 | KF926700 | Laos | 7 | 19 | 56 | I | 1H | 1H3 |
| 2011\|KT825049\|Sri_Lanka | 2011 | KT825049 | Sri_Lanka | 7 | 18 | 42 | I | 1H | 1H5 |
| 2011\|LC148030\|Maldives | 2011 | LC148030 | Maldives | 7 | 18 | 42 | I | 1H | 1H5 |
| 2012\|KJ726662\|Sri_Lanka | 2012 | KJ726662 | Sri_Lanka | 7 | 18 | 42 | I | 1H | 1H5 |
| 2012\|KY978438\|India | 2012 | KY978438 | India | 7 | 18 | 42 | I | 1H | 1H5 |
| 2012\|KT824994\|Thailand | 2012 | KT824994 | Thailand | 7 | 18 | 43 | I | 1H | 1H5 |
| 2013\|KJ545444\|China | 2013 | KJ545444 | China | 7 | 18 | 43 | I | 1H | 1H5 |
| 2013\|KJ545482\|Thailand | 2013 | KJ545482 | Thailand | 7 | 18 | 43 | I | 1H | 1H5 |
| 2013\|KJ755855\|India | 2013 | KJ755855 | India | 7 | 18 | 42 | I | 1H | 1H5 |
| 2013\|KJ806941\|Singapore | 2013 | KJ806941 | Singapore | 7 | 17 | 41 | I | 1H | 1H4 |
| 2013\|KJ806946\|Singapore | 2013 | KJ806946 | Singapore | 7 | 18 | 43 | I | 1H | 1H5 |
| 2013\|KR051919\|Myanmar | 2013 | KR051919 | Myanmar | 7 | 18 | 43 | I | 1H | 1H5 |
| 2013\|KR051920\|Myanmar | 2013 | KR051920 | Myanmar | 7 | 17 | 41 | I | 1H | 1H4 |
| 2013\|KT825018\|Australia | 2013 | KT825018 | Australia | 7 | 18 | 43 | I | 1H | 1H5 |
| 2013\|KT825058\|Thailand | 2013 | KT825058 | Thailand | 7 | 18 | 43 | I | 1H | 1H5 |
| 2013\|KU509310\|Sri_Lanka | 2013 | KU509310 | Sri_Lanka | 7 | 18 | 42 | I | 1H | 1H5 |
| 2013\|KY038883\|China | 2013 | KY038883 | China | 7 | 17 | 40 | I | 1H | 1H4 |
| 2014\|KT175104\|Myanmar | 2014 | KT175104 | Myanmar | 7 | 17 | 41 | I | 1H | 1H4 |
| 2014\|KT825029\|Sri_Lanka | 2014 | KT825029 | Sri_Lanka | 7 | 18 | 42 | I | 1H | 1H5 |
| 2014\|KY038888\|China | 2014 | KY038888 | China | 7 | 18 | 43 | I | 1H | 1H5 |
| 2015\|KX056462\|China | 2015 | KX056462 | China | 7 | 17 | 40 | I | 1H | 1H4 |
| 2015\|KX056469\|China | 2015 | KX056469 | China | 7 | 18 | 43 | I | 1H | 1H5 |
| 2015\|KX357900\|Myanmar | 2015 | KX357900 | Myanmar | 7 | 18 | 43 | I | 1H | 1H5 |
| 2015\|KX357970\|Myanmar | 2015 | KX357970 | Myanmar | 7 | 17 | 39 | I | 1H | 1H4 |
| 2015\|KY495792\|Thailand | 2015 | KY495792 | Thailand | 7 | 17 | 41 | I | 1H | 1H4 |
| 2015\|KY978439\|India | 2015 | KY978439 | India | 7 | 18 | 42 | I | 1H | 1H5 |
| 2000\|FJ639669\|Cambodia | 2000 | FJ639669 | Cambodia | 5 | 13 | 14 | I | 1I | 1I1 |
| 2001\|FJ639672\|Cambodia | 2001 | FJ639672 | Cambodia | 5 | 13 | 14 | I | 1I | 1I1 |
| 2003\|FJ882563\|Vietnam | 2003 | FJ882563 | Vietnam | 5 | 13 | 14 | I | 1I | 1I1 |
| 2003\|GQ868619\|Cambodia | 2003 | GQ868619 | Cambodia | 5 | 13 | 14 | I | 1I | 1I1 |
| 2006\|HQ588121\|Vietnam | 2006 | HQ588121 | Vietnam | 5 | 13 | 14 | I | 1I | 1I1 |
| 2008\|JF967828\|Vietnam | 2008 | JF967828 | Vietnam | 5 | 13 | 14 | I | 1I | 1I1 |
| 2009\|KC861928\|Vietnam | 2009 | KC861928 | Vietnam | 5 | 13 | 14 | I | 1I | 1I1 |
| 2002\|AB111079\|Thailand | 2002 | AB111079 | Thailand | 8 | 20 | 24 | I | 1J | 1J6 |
| 2002\|AB178040\|Micronesia | 2002 | AB178040 | Micronesia | 8 | 20 | 24 | I | 1J | 1J6 |
| 2002\|EU069600\|Singapore | 2002 | EU069600 | Singapore | 8 | 20 | 24 | I | 1J | 1J6 |
| 2003\|EU448396\|Thailand | 2003 | EU448396 | Thailand | 8 | 20 | 24 | I | 1J | 1J6 |
| 2003\|FJ469909\|Singapore | 2003 | FJ469909 | Singapore | 8 | 20 | 24 | I | 1J | 1J6 |
| 2006\|EU448400\|Malaysia | 2006 | EU448400 | Malaysia | 8 | 21 | 33 | I | 1J | 1J4 |
| 2006\|KC861918\|Vietnam | 2006 | KC861918 | Vietnam | 8 | 21 | 33 | I | 1J | 1J4 |
| 2006\|JQ993196\|Thailand | 2006 | JQ993196 | Thailand | 8 | 20 | 24 | I | 1J | 1J6 |
| 2007\|EU448397\|Vietnam | 2007 | EU448397 | Vietnam | 8 | 21 | 32 | I | 1J | 1J1 |
| 2007\|KC182102\|Laos | 2007 | KC182102 | Laos | 8 | 21 | 32 | I | 1J | 1J1 |
| 2007\|JN638334\|Thailand | 2007 | JN638334 | Thailand | 8 | 21 | 32 | I | 1J | 1J1 |
| 2007\|KX951690\|China | 2007 | KX951690 | China | 8 | 21 | 32 | I | 1J | 1J1 |
| 2007\|JQ993201\|Thailand | 2007 | JQ993201 | Thailand | 8 | 21 | 32 | I | 1J | 1J2 |
| 2007\|HM469968\|Thailand | 2007 | HM469968 | Thailand | 8 | 21 | 32 | I | 1J | 1J3 |
| 2007\|KT827368\|China | 2007 | KT827368 | China | 8 | 21 | 33 | I | 1J | 1J4 |
| 2007\|EU448398\|Thailand | 2007 | EU448398 | Thailand | 8 | 20 | 24 | I | 1J | 1J6 |
| 2008\|JF967819\|Thailand | 2008 | JF967819 | Thailand | 8 | 21 | 32 | I | 1J | 1J1 |
| 2008\|KC182105\|Laos | 2008 | KC182105 | Laos | 8 | 21 | 32 | I | 1J | 1J1 |
| 2008\|KY849749\|Laos | 2008 | KY849749 | Laos | 8 | 21 | 32 | I | 1J | 1J2 |
| 2008\|JF967801\|Thailand | 2008 | JF967801 | Thailand | 8 | 21 | 32 | I | 1J | 1J2 |
| 2008\|JF967803\|Thailand | 2008 | JF967803 | Thailand | 8 | 21 | 32 | I | 1J | 1J3 |
| 2008\|GQ357685\|Singapore | 2008 | GQ357685 | Singapore | 8 | 21 | 33 | I | 1J | 1J4 |
| 2008\|JF967798\|Indonesia | 2008 | JF967798 | Indonesia | 8 | 21 | 33 | I | 1J | 1J4 |
| 2008\|JQ317737\|China | 2008 | JQ317737 | China | 8 | 21 | 34 | I | 1J | 1J5 |
| 2008\|KC172833\|Laos | 2008 | KC172833 | Laos | 8 | 21 | 34 | I | 1J | 1J5 |
| 2008\|JN022601\|Singapore | 2008 | JN022601 | Singapore | 8 | 20 | 24 | I | 1J | 1J6 |
| 2008\|KC861935\|Vietnam | 2008 | KC861935 | Vietnam | 8 | 20 | 24 | I | 1J | 1J6 |
| 2009\|JF967875\|Indonesia | 2009 | JF967875 | Indonesia | 8 | 21 | 32 | I | 1J | 1J1 |
| 2009\|KT373896\|Thailand | 2009 | KT373896 | Thailand | 8 | 21 | 32 | I | 1J | 1J1 |
| 2009\|KT373899\|Thailand | 2009 | KT373899 | Thailand | 8 | 21 | 32 | I | 1J | 1J2 |
| 2009\|KY849727\|Laos | 2009 | KY849727 | Laos | 8 | 21 | 32 | I | 1J | 1J2 |
| 2009\|JQ403519\|China | 2009 | JQ403519 | China | 8 | 21 | 32 | I | 1J | 1J3 |
| 2009\|KT373897\|Thailand | 2009 | KT373897 | Thailand | 8 | 21 | 32 | I | 1J | 1J3 |
| 2009\|JF960214\|Singapore | 2009 | JF960214 | Singapore | 8 | 21 | 33 | I | 1J | 1J4 |
| 2009\|JF967855\|Indonesia | 2009 | JF967855 | Indonesia | 8 | 21 | 33 | I | 1J | 1J4 |
| 2009\|JN638335\|Thailand | 2009 | JN638335 | Thailand | 8 | 21 | 34 | I | 1J | 1J5 |
| 2009\|KY849748\|Laos | 2009 | KY849748 | Laos | 8 | 21 | 34 | I | 1J | 1J5 |
| 2009\|JF960212\|Singapore | 2009 | JF960212 | Singapore | 8 | 20 | 25 | I | 1J | 1J6 |
| 2010\|AB624554\|Indonesia | 2010 | AB624554 | Indonesia | 8 | 21 | 32 | I | 1J | 1J1 |
| 2010\|JF967923\|Thailand | 2010 | JF967923 | Thailand | 8 | 21 | 32 | I | 1J | 1J1 |
| 2010\|JN415528\|Thailand | 2010 | JN415528 | Thailand | 8 | 21 | 32 | I | 1J | 1J2 |
| 2010\|JF967919\|Cambodia | 2010 | JF967919 | Cambodia | 8 | 21 | 32 | I | 1J | 1J2 |
| 2010\|JF960222\|Singapore | 2010 | JF960222 | Singapore | 8 | 21 | 33 | I | 1J | 1J4 |
| 2010\|JF967896\|Indonesia | 2010 | JF967896 | Indonesia | 8 | 21 | 33 | I | 1J | 1J4 |
| 2010\|JN380807\|Singapore | 2010 | JN380807 | Singapore | 8 | 21 | 33 | I | 1J | 1J4 |
| 2010\|KC182091\|Laos | 2010 | KC182091 | Laos | 8 | 21 | 34 | I | 1J | 1J5 |
| 2010\|JF960224\|Singapore | 2010 | JF960224 | Singapore | 8 | 20 | 25 | I | 1J | 1J6 |
| 2010\|KT204460\|Indonesia | 2010 | KT204460 | Indonesia | 8 | 20 | 25 | I | 1J | 1J6 |
| 2010\|JN415489\|Indonesia | 2010 | JN415489 | Indonesia | 8 | 22 | 64 | I | 1J | 1J7 |
| 2011\|KT824981\|Thailand | 2011 | KT824981 | Thailand | 8 | 21 | 32 | I | 1J | 1J3 |
| 2011\|KY849704\|Laos | 2011 | KY849704 | Laos | 8 | 21 | 34 | I | 1J | 1J5 |
| 2011\|KT825072\|Indonesia | 2011 | KT825072 | Indonesia | 8 | 20 | 25 | I | 1J | 1J6 |
| 2011\|KM216688\|Indonesia | 2011 | KM216688 | Indonesia | 8 | 22 | 64 | I | 1J | 1J7 |
| 2012\|KJ806934\|Singapore | 2012 | KJ806934 | Singapore | 8 | 21 | 32 | I | 1J | 1J3 |
| 2012\|KY818158\|Thailand | 2012 | KY818158 | Thailand | 8 | 21 | 32 | I | 1J | 1J3 |
| 2012\|KF052647\|Indonesia | 2012 | KF052647 | Indonesia | 8 | 21 | 33 | I | 1J | 1J4 |
| 2012\|KY818146\|Thailand | 2012 | KY818146 | Thailand | 8 | 21 | 34 | I | 1J | 1J5 |
| 2012\|KT204449\|Indonesia | 2012 | KT204449 | Indonesia | 8 | 20 | 25 | I | 1J | 1J6 |
| 2012\|KT824984\|Indonesia | 2012 | KT824984 | Indonesia | 8 | 22 | 64 | I | 1J | 1J7 |
| 2012\|KT824997\|Australia | 2012 | KT824997 | Australia | 8 | 22 | 64 | I | 1J | 1J7 |
| 2013\|AB915376\|Indonesia | 2013 | AB915376 | Indonesia | 8 | 20 | 25 | I | 1J | 1J6 |
| 2013\|KJ438293\|China | 2013 | KJ438293 | China | 8 | 21 | 32 | I | 1J | 1J2 |
| 2013\|KJ806947\|Singapore | 2013 | KJ806947 | Singapore | 8 | 21 | 33 | I | 1J | 1J4 |
| 2013\|KT825013\|Thailand | 2013 | KT825013 | Thailand | 8 | 21 | 32 | I | 1J | 1J3 |
| 2013\|KT825015\|Australia | 2013 | KT825015 | Australia | 8 | 22 | 64 | I | 1J | 1J7 |
| 2013\|KT825053\|Indonesia | 2013 | KT825053 | Indonesia | 8 | 22 | 64 | I | 1J | 1J7 |
| 2013\|KY882504\|Thailand | 2013 | KY882504 | Thailand | 8 | 21 | 34 | I | 1J | 1J5 |
| 2014\|KP191493\|China | 2014 | KP191493 | China | 8 | 22 | 64 | I | 1J | 1J7 |
| 2014\|KT825031\|Thailand | 2014 | KT825031 | Thailand | 8 | 22 | 64 | I | 1J | 1J7 |
| 2014\|KU529696\|Indonesia | 2014 | KU529696 | Indonesia | 8 | 21 | 33 | I | 1J | 1J4 |
| 2014\|KX452065\|Malaysia | 2014 | KX452065 | Malaysia | 8 | 22 | 64 | I | 1J | 1J7 |
| 2014\|LC012534\|Singapore | 2014 | LC012534 | Singapore | 8 | 22 | 64 | I | 1J | 1J7 |
| 2014\|LC012535\|Japan | 2014 | LC012535 | Japan | 8 | 21 | 32 | I | 1J | 1J3 |
| 2014\|LC016760\|Japan | 2014 | LC016760 | Japan | 8 | 22 | 64 | I | 1J | 1J7 |
| 2014\|LC038150\|Indonesia | 2014 | LC038150 | Indonesia | 8 | 22 | 64 | I | 1J | 1J7 |
| 2015\|KT306907\|China | 2015 | KT306907 | China | 8 | 22 | 64 | I | 1J | 1J7 |
| 2015\|KT825071\|Australia | 2015 | KT825071 | Australia | 8 | 22 | 64 | I | 1J | 1J7 |
| 2015\|KU509289\|Thailand | 2015 | KU509289 | Thailand | 8 | 22 | 64 | I | 1J | 1J7 |
| 2015\|KU529727\|Indonesia | 2015 | KU529727 | Indonesia | 8 | 20 | 24 | I | 1J | 1J6 |
| 2015\|KY006132\|Indonesia | 2015 | KY006132 | Indonesia | 8 | 22 | 64 | I | 1J | 1J7 |
| 2006\|EF508206\|China | 2006 | EF508206 | China | 12 | 30 | 44 | I | 1K | 1K1 |
| 2008\|KU509257\|Thailand | 2008 | KU509257 | Thailand | 12 | 30 | 44 | I | 1K | 1K1 |
| 2008\|LC148025\|Cambodia | 2008 | LC148025 | Cambodia | 12 | 30 | 44 | I | 1K | 1K1 |
| 2009\|HQ149731\|China | 2009 | HQ149731 | China | 12 | 30 | 44 | I | 1K | 1K1 |
| 2009\|KT373898\|Thailand | 2009 | KT373898 | Thailand | 12 | 30 | 44 | I | 1K | 1K1 |
| 2012\|KY818160\|Thailand | 2012 | KY818160 | Thailand | 12 | 30 | 45 | I | 1K | 1K1 |
| 2013\|KJ806945\|Singapore | 2013 | KJ806945 | Singapore | 12 | 31 | 65 | I | 1K | 1K1 |
| 2013\|KT825002\|Australia | 2013 | KT825002 | Australia | 12 | 31 | 65 | I | 1K | 1K1 |
| 2013\|KX621251\|China | 2013 | KX621251 | China | 12 | 31 | 65 | I | 1K | 1K1 |
| 2014\|KT825064\|Australia | 2014 | KT825064 | Australia | 12 | 31 | 65 | I | 1K | 1K1 |
| 2014\|KT827372\|China | 2014 | KT827372 | China | 12 | 31 | 65 | I | 1K | 1K1 |
| 2015\|KT827379\|China | 2015 | KT827379 | China | 12 | 31 | 65 | I | 1K | 1K1 |
| 2008\|JF967796\|Malaysia | 2008 | JF967796 | Malaysia | 13 | 33 | 61 | I | 1L | 1L1 |
| 2008\|GQ357687\|Singapore | 2008 | GQ357687 | Singapore | 13 | 32 | 59 | I | 1L | 1L2 |
| 2008\|JF967821\|Malaysia | 2008 | JF967821 | Malaysia | 13 | 32 | 59 | I | 1L | 1L2 |
| 2009\|JF967876\|Malaysia | 2009 | JF967876 | Malaysia | 13 | 33 | 61 | I | 1L | 1L1 |
| 2009\|JF960218\|Singapore | 2009 | JF960218 | Singapore | 13 | 32 | 59 | I | 1L | 1L2 |
| 2009\|JF967887\|Malaysia | 2009 | JF967887 | Malaysia | 13 | 32 | 59 | I | 1L | 1L2 |
| 2010\|JF967911\|Malaysia | 2010 | JF967911 | Malaysia | 13 | 33 | 61 | I | 1L | 1L1 |
| 2010\|JQ317733\|China | 2010 | JQ317733 | China | 13 | 33 | 61 | I | 1L | 1L1 |
| 2010\|KM216674\|Indonesia | 2010 | KM216674 | Indonesia | 13 | 33 | 62 | I | 1L | 1L1 |
| 2010\|JF960223\|Singapore | 2010 | JF960223 | Singapore | 13 | 32 | 59 | I | 1L | 1L2 |
| 2010\|JF967897\|Malaysia | 2010 | JF967897 | Malaysia | 13 | 32 | 59 | I | 1L | 1L2 |
| 2012\|KY818153\|Malaysia | 2012 | KY818153 | Malaysia | 13 | 33 | 61 | I | 1L | 1L1 |
| 2012\|AB915381\|Indonesia | 2012 | AB915381 | Indonesia | 13 | 32 | 59 | I | 1L | 1L2 |
| 2012\|KT204450\|Indonesia | 2012 | KT204450 | Indonesia | 13 | 32 | 59 | I | 1L | 1L2 |
| 2013\|KJ806949\|Singapore | 2013 | KJ806949 | Singapore | 13 | 33 | 61 | I | 1L | 1L1 |
| 2013\|KJ806951\|Singapore | 2013 | KJ806951 | Singapore | 13 | 34 | 66 | I | 1L | 1L3 |
| 2013\|KT825052\|Indonesia | 2013 | KT825052 | Indonesia | 13 | 34 | 66 | I | 1L | 1L3 |
| 2013\|KU509291\|Thailand | 2013 | KU509291 | Thailand | 13 | 33 | 61 | I | 1L | 1L1 |
| 2013\|KY818145\|Indonesia | 2013 | KY818145 | Indonesia | 13 | 32 | 59 | I | 1L | 1L2 |
| 2014\|KT825061\|Indonesia | 2014 | KT825061 | Indonesia | 13 | 32 | 59 | I | 1L | 1L2 |
| 2014\|KU365900\|China | 2014 | KU365900 | China | 13 | 34 | 66 | I | 1L | 1L3 |
| 2014\|KU509292\|Vietnam | 2014 | KU509292 | Vietnam | 13 | 33 | 61 | I | 1L | 1L1 |
| 2014\|KU666939\|Malaysia | 2014 | KU666939 | Malaysia | 13 | 32 | 60 | I | 1L | 1L2 |
| 2014\|KX224258\|Singapore | 2014 | KX224258 | Singapore | 13 | 33 | 61 | I | 1L | 1L1 |
| 2014\|KX224261\|Singapore | 2014 | KX224261 | Singapore | 13 | 32 | 60 | I | 1L | 1L2 |
| 2014\|KX225492\|China | 2014 | KX225492 | China | 13 | 32 | 60 | I | 1L | 1L2 |
| 2014\|LC038144\|Indonesia | 2014 | LC038144 | Indonesia | 13 | 34 | 66 | I | 1L | 1L3 |
| 2014\|LC062957\|Japan | 2014 | LC062957 | Japan | 13 | 34 | 66 | I | 1L | 1L3 |
| 2015\|KT825039\|Myanmar | 2015 | KT825039 | Myanmar | 13 | 32 | 60 | I | 1L | 1L2 |
| 2015\|KT825069\|Malaysia | 2015 | KT825069 | Malaysia | 13 | 32 | 60 | I | 1L | 1L2 |
| 2015\|KU529701\|Indonesia | 2015 | KU529701 | Indonesia | 13 | 32 | 59 | I | 1L | 1L2 |
| 2015\|KU529702\|Indonesia | 2015 | KU529702 | Indonesia | 13 | 33 | 62 | I | 1L | 1L1 |
| 2015\|KY006130\|Indonesia | 2015 | KY006130 | Indonesia | 13 | 34 | 66 | I | 1L | 1L3 |
| 2016\|KX621253\|China | 2016 | KX621253 | China | 13 | 32 | 60 | I | 1L | 1L2 |
| 2007\|KC762654\|Indonesia | 2007 | KC762654 | Indonesia | 11 | 26 | 35 | I | 1M | 1M1 |
| 2007\|EU448401\|Indonesia | 2007 | EU448401 | Indonesia | 11 | 27 | 50 | I | 1M | 1M2 |
| 2008\|KC762626\|Indonesia | 2008 | KC762626 | Indonesia | 11 | 26 | 36 | I | 1M | 1M1 |
| 2008\|FJ687477\|Indonesia | 2008 | FJ687477 | Indonesia | 11 | 27 | 50 | I | 1M | 1M2 |
| 2008\|JN415531\|Australia | 2008 | JN415531 | Australia | 11 | 28 | 57 | I | 1M | 1M8 |
| 2008\|JF967813\|Singapore | 2008 | JF967813 | Singapore | 11 | 28 | 58 | I | 1M | 1M8 |
| 2008\|JF967832\|Indonesia | 2008 | JF967832 | Indonesia | 11 | 28 | 58 | I | 1M | 1M8 |
| 2009\|JN415519\|Papua_New_Guinea | 2009 | JN415519 | Papua_New_Guinea | 11 | 26 | 35 | I | 1M | 1M1 |
| 2009\|HQ149730\|China | 2009 | HQ149730 | China | 11 | 27 | 51 | I | 1M | 1M3 |
| 2009\|JF967857\|Indonesia | 2009 | JF967857 | Indonesia | 11 | 27 | 51 | I | 1M | 1M3 |
| 2009\|JN380805\|Singapore | 2009 | JN380805 | Singapore | 11 | 28 | 57 | I | 1M | 1M8 |
| 2010\|KC762639\|Indonesia | 2010 | KC762639 | Indonesia | 11 | 26 | 35 | I | 1M | 1M1 |
| 2010\|KM216681\|Indonesia | 2010 | KM216681 | Indonesia | 11 | 27 | 50 | I | 1M | 1M2 |
| 2010\|JF960234\|Singapore | 2010 | JF960234 | Singapore | 11 | 27 | 51 | I | 1M | 1M3 |
| 2010\|JF967931\|Indonesia | 2010 | JF967931 | Indonesia | 11 | 27 | 51 | I | 1M | 1M3 |
| 2010\|JF967900\|Indonesia | 2010 | JF967900 | Indonesia | 11 | 27 | 51 | I | 1M | 1M4 |
| 2010\|JF960233\|Singapore | 2010 | JF960233 | Singapore | 11 | 27 | 52 | I | 1M | 1M7 |
| 2010\|KC762642\|Indonesia | 2010 | KC762642 | Indonesia | 11 | 28 | 58 | I | 1M | 1M8 |
| 2011\|KT824977\|Papua_New_Guinea | 2011 | KT824977 | Papua_New_Guinea | 11 | 26 | 35 | I | 1M | 1M1 |
| 2011\|KT824970\|Indonesia | 2011 | KT824970 | Indonesia | 11 | 27 | 50 | I | 1M | 1M2 |
| 2012\|KC589008\|Indonesia | 2012 | KC589008 | Indonesia | 11 | 27 | 50 | I | 1M | 1M2 |
| 2012\|KT204446\|Indonesia | 2012 | KT204446 | Indonesia | 11 | 27 | 52 | I | 1M | 1M7 |
| 2012\|KC589009\|Indonesia | 2012 | KC589009 | Indonesia | 11 | 28 | 58 | I | 1M | 1M8 |
| 2013\|AB915378\|Indonesia | 2013 | AB915378 | Indonesia | 11 | 27 | 52 | I | 1M | 1M7 |
| 2013\|KY818092\|Indonesia | 2013 | KY818092 | Indonesia | 11 | 27 | 51 | I | 1M | 1M3 |
| 2013\|KY818138\|Indonesia | 2013 | KY818138 | Indonesia | 11 | 27 | 51 | I | 1M | 1M6 |
| 2014\|KT175091\|Indonesia | 2014 | KT175091 | Indonesia | 11 | 28 | 58 | I | 1M | 1M8 |
| 2014\|KT175095\|Indonesia | 2014 | KT175095 | Indonesia | 11 | 26 | 36 | I | 1M | 1M1 |
| 2014\|KU529691\|Indonesia | 2014 | KU529691 | Indonesia | 11 | 29 | 67 | I | 1M | 1M9 |
| 2015\|KU529700\|Indonesia | 2015 | KU529700 | Indonesia | 11 | 29 | 67 | I | 1M | 1M9 |
| 2015\|KU529712\|Indonesia | 2015 | KU529712 | Indonesia | 11 | 27 | 51 | I | 1M | 1M5 |
| 2015\|KU529738\|Indonesia | 2015 | KU529738 | Indonesia | 11 | 28 | 58 | I | 1M | 1M8 |
| 2015\|KY006133\|Indonesia | 2015 | KY006133 | Indonesia | 11 | 26 | 36 | I | 1M | 1M1 |
| 1973\|U88535\|Nauru_Island | 1973 | U88535 | Nauru_Island | 1 | 1 | 1 | IV | 4A | 4A2 |
| 1974\|AF425627\|Philippines | 1974 | AF425627 | Philippines | 1 | 1 | 1 | IV | 4A | 4A1 |
| 1984\|D00503\|Philippines | 1984 | D00503 | Philippines | 1 | 1 | 1 | IV | 4A | 4A2 |
| 1991\|FJ196845\|China | 1991 | FJ196845 | China | 1 | 1 | 1 | IV | 4A | 4A3 |
| 1995\|AY422784\|Philippines | 1995 | AY422784 | Philippines | 1 | 1 | 1 | IV | 4A | 4A4 |
| 1999\|AY422785\|Philippines | 1999 | AY422785 | Philippines | 1 | 1 | 1 | IV | 4A | 4A9 |
| 2000\|JN415515\|Palau | 2000 | JN415515 | Palau | 1 | 1 | 1 | IV | 4A | 4A5 |
| 2001\|AB111068\|Samoa | 2001 | AB111068 | Samoa | 1 | 1 | 2 | IV | 4A | 4A10 |
| 2001\|DQ091261\|USA.Hawaii | 2001 | DQ091261 | USA.Hawaii | 1 | 1 | 2 | IV | 4A | 4A10 |
| 2001\|JQ655095\|Samoa | 2001 | JQ655095 | Samoa | 1 | 1 | 2 | IV | 4A | 4A10 |
| 2001\|AY422777\|Philippines | 2001 | AY422777 | Philippines | 1 | 1 | 1 | IV | 4A | 4A8 |
| 2002\|AY422783\|Philippines | 2002 | AY422783 | Philippines | 1 | 1 | 2 | IV | 4A | 4A10 |
| 2002\|AB111074\|Philippines | 2002 | AB111074 | Philippines | 1 | 1 | 2 | IV | 4A | 4A11 |
| 2002\|AY422778\|Philippines | 2002 | AY422778 | Philippines | 1 | 1 | 1 | IV | 4A | 4A6 |
| 2002\|AY422781\|Philippines | 2002 | AY422781 | Philippines | 1 | 1 | 1 | IV | 4A | 4A7 |
| 2003\|EU448406\|Philippines | 2003 | EU448406 | Philippines | 1 | 1 | 2 | IV | 4A | 4A10 |
| 2006\|KP406803\|Philippines | 2006 | KP406803 | Philippines | 1 | 1 | 2 | IV | 4A | 4A13 |
| 2007\|EU448405\|Philippines | 2007 | EU448405 | Philippines | 1 | 2 | 32 | IV | 4A | 4A14 |
| 2008\|KJ946238\|Philippines | 2008 | KJ946238 | Philippines | 1 | 1 | 2 | IV | 4A | 4A12 |
| 2008\|KJ946237\|Philippines | 2008 | KJ946237 | Philippines | 1 | 2 | 32 | IV | 4A | 4A14 |
| 2010\|JF967936\|Philippines | 2010 | JF967936 | Philippines | 1 | 2 | 32 | IV | 4A | 4A14 |
| 2011\|KT825047\|Philippines | 2011 | KT825047 | Philippines | 1 | 2 | 32 | IV | 4A | 4A14 |
| 2012\|KY818204\|Philippines | 2012 | KY818204 | Philippines | 1 | 2 | 33 | IV | 4A | 4A14 |
| 2013\|KU509316\|Thailand | 2013 | KU509316 | Thailand | 1 | 2 | 32 | IV | 4A | 4A14 |
| 2013\|KU570101\|China | 2013 | KU570101 | China | 1 | 2 | 33 | IV | 4A | 4A14 |
| 2013\|KY818173\|Philippines | 2013 | KY818173 | Philippines | 1 | 2 | 33 | IV | 4A | 4A14 |
| 2014\|KT175098\|Philippines | 2014 | KT175098 | Philippines | 1 | 2 | 32 | IV | 4A | 4A14 |
| 2016\|KY495796\|Philippines | 2016 | KY495796 | Philippines | 1 | 2 | 33 | IV | 4A | 4A14 |
| 2003\|EU448404\|Indonesia | 2003 | EU448404 | Indonesia | 7 | 21 | 22 | IV | 4B | 4B1 |
| 2006\|EU448402\|Indonesia | 2006 | EU448402 | Indonesia | 7 | 22 | 26 | IV | 4B | 4B3 |
| 2006\|EU448403\|Vietnam | 2006 | EU448403 | Vietnam | 7 | 22 | 26 | IV | 4B | 4B3 |
| 2008\|KC762640\|Indonesia | 2008 | KC762640 | Indonesia | 7 | 22 | 27 | IV | 4B | 4B3 |
| 2009\|JF967865\|Indonesia | 2009 | JF967865 | Indonesia | 7 | 21 | 23 | IV | 4B | 4B1 |
| 2010\|KT204461\|Indonesia | 2010 | KT204461 | Indonesia | 7 | 23 | 44 | IV | 4B | 4B3 |
| 2011\|KT824973\|Indonesia | 2011 | KT824973 | Indonesia | 7 | 22 | 27 | IV | 4B | 4B3 |
| 2011\|KT824982\|Australia | 2011 | KT824982 | Australia | 7 | 22 | 27 | IV | 4B | 4B3 |
| 2012\|AB915368\|Indonesia | 2012 | AB915368 | Indonesia | 7 | 21 | 24 | IV | 4B | 4B2 |
| 2012\|KT204447\|Indonesia | 2012 | KT204447 | Indonesia | 7 | 23 | 44 | IV | 4B | 4B3 |
| 2013\|AB915384\|Indonesia | 2013 | AB915384 | Indonesia | 7 | 23 | 44 | IV | 4B | 4B3 |
| 2007\|FJ687476\|Philippines | 2007 | FJ687476 | Philippines | 8 | 24 | 34 | IV | 4C | 4C3 |
| 2010\|KT827367\|China | 2010 | KT827367 | China | 8 | 24 | 34 | IV | 4C | 4C1 |
| 2010\|JF967937\|Philippines | 2010 | JF967937 | Philippines | 8 | 24 | 34 | IV | 4C | 4C3 |
| 2011\|KY818116\|Philippines | 2011 | KY818116 | Philippines | 8 | 24 | 34 | IV | 4C | 4C1 |
| 2011\|KY818161\|Philippines | 2011 | KY818161 | Philippines | 8 | 24 | 34 | IV | 4C | 4C2 |
| 2012\|KJ933413\|China | 2012 | KJ933413 | China | 8 | 24 | 34 | IV | 4C | 4C1 |
| 2012\|KY882506\|Philippines | 2012 | KY882506 | Philippines | 8 | 24 | 34 | IV | 4C | 4C1 |
| 2012\|KY882507\|Philippines | 2012 | KY882507 | Philippines | 8 | 24 | 34 | IV | 4C | 4C2 |
| 2012\|KY882502\|Philippines | 2012 | KY882502 | Philippines | 8 | 24 | 34 | IV | 4C | 4C3 |
| 2013\|KJ415096\|Angola | 2013 | KJ415096 | Angola | 8 | 24 | 34 | IV | 4C | 4C3 |
| 2013\|KJ806939\|Singapore | 2013 | KJ806939 | Singapore | 8 | 24 | 34 | IV | 4C | 4C1 |
| 2013\|KY818202\|Philippines | 2013 | KY818202 | Philippines | 8 | 24 | 34 | IV | 4C | 4C1 |
| 2014\|KT175081\|China | 2014 | KT175081 | China | 8 | 24 | 34 | IV | 4C | 4C3 |
| 2014\|KT175096\|Philippines | 2014 | KT175096 | Philippines | 8 | 24 | 34 | IV | 4C | 4C1 |
| 2014\|KT175099\|Philippines | 2014 | KT175099 | Philippines | 8 | 24 | 34 | IV | 4C | 4C2 |
| 2015\|KT825043\|Australia | 2015 | KT825043 | Australia | 8 | 24 | 34 | IV | 4C | 4C3 |
| 2015\|KU310948\|China | 2015 | KU310948 | China | 8 | 24 | 34 | IV | 4C | 4C3 |
| 2016\|KY496854\|China | 2016 | KY496854 | China | 8 | 24 | 34 | IV | 4C | 4C1 |
| 1983\|AF425611\|Australia | 1983 | AF425611 | Australia | 2 | 3 | 3 | IV | 4D | 4D1 |
| 2000\|JN415499\|East_Timor | 2000 | JN415499 | East_Timor | 2 | 4 | 14 | IV | 4D | 4D3 |
| 2001\|KY275186\|East_Timor | 2001 | KY275186 | East_Timor | 2 | 4 | 14 | IV | 4D | 4D3 |
| 2002\|KY275188\|East_Timor | 2002 | KY275188 | East_Timor | 2 | 4 | 14 | IV | 4D | 4D3 |
| 2007\|KC762651\|Indonesia | 2007 | KC762651 | Indonesia | 2 | 5 | 37 | IV | 4D | 4D2 |
| 2008\|JN415500\|East_Timor | 2008 | JN415500 | East_Timor | 2 | 4 | 14 | IV | 4D | 4D3 |
| 2009\|KT825045\|Australia | 2009 | KT825045 | Australia | 2 | 4 | 15 | IV | 4D | 4D3 |
| 2010\|JN415502\|East_Timor | 2010 | JN415502 | East_Timor | 2 | 4 | 15 | IV | 4D | 4D3 |
| 2010\|JN415510\|Indonesia | 2010 | JN415510 | Indonesia | 2 | 4 | 15 | IV | 4D | 4D3 |
| 1993\|DQ211348\|China | 1993 | DQ211348 | China | 4 | 10 | 5 | IV | 4E | 4E4 |
| 1998\|AB189121\|Indonesia | 1998 | AB189121 | Indonesia | 4 | 11 | 12 | IV | 4E | 4E1 |
| 2002\|JF459993\|Myanmar | 2002 | JF459993 | Myanmar | 4 | 11 | 13 | IV | 4E | 4E2 |
| 2002\|DQ855296\|China | 2002 | DQ855296 | China | 4 | 10 | 5 | IV | 4E | 4E4 |
| 2002\|AB232666\|Indonesia | 2002 | AB232666 | Indonesia | 4 | 10 | 6 | IV | 4E | 4E5 |
| 2003\|FJ196842\|China | 2003 | FJ196842 | China | 4 | 10 | 5 | IV | 4E | 4E4 |
| 2003\|JN415488\|Indonesia | 2003 | JN415488 | Indonesia | 4 | 10 | 5 | IV | 4E | 4E4 |
| 2003\|JN415514\|Australia | 2003 | JN415514 | Australia | 4 | 10 | 5 | IV | 4E | 4E4 |
| 2007\|EU448409\|Indonesia | 2007 | EU448409 | Indonesia | 4 | 10 | 5 | IV | 4E | 4E4 |
| 2007\|KT827366\|China | 2007 | KT827366 | China | 4 | 10 | 5 | IV | 4E | 4E4 |
| 2008\|JF967793\|Indonesia | 2008 | JF967793 | Indonesia | 4 | 10 | 7 | IV | 4E | 4E7 |
| 2008\|KC762630\|Indonesia | 2008 | KC762630 | Indonesia | 4 | 12 | 38 | IV | 4E | 4E8 |
| 2009\|JF967867\|Indonesia | 2009 | JF967867 | Indonesia | 4 | 11 | 12 | IV | 4E | 4E1 |
| 2009\|JF967854\|Indonesia | 2009 | JF967854 | Indonesia | 4 | 10 | 7 | IV | 4E | 4E7 |
| 2009\|AB597980\|Indonesia | 2009 | AB597980 | Indonesia | 4 | 12 | 39 | IV | 4E | 4E8 |
| 2010\|KU509261\|Indonesia | 2010 | KU509261 | Indonesia | 4 | 11 | 12 | IV | 4E | 4E1 |
| 2010\|JF967918\|Indonesia | 2010 | JF967918 | Indonesia | 4 | 11 | 13 | IV | 4E | 4E3 |
| 2010\|KY275190\|East_Timor | 2010 | KY275190 | East_Timor | 4 | 10 | 6 | IV | 4E | 4E6 |
| 2010\|HQ871946\|Australia | 2010 | HQ871946 | Australia | 4 | 10 | 7 | IV | 4E | 4E7 |
| 2010\|KM216668\|Indonesia | 2010 | KM216668 | Indonesia | 4 | 10 | 7 | IV | 4E | 4E7 |
| 2010\|JF967902\|Indonesia | 2010 | JF967902 | Indonesia | 4 | 12 | 38 | IV | 4E | 4E8 |
| 2011\|JN544411\|Singapore | 2011 | JN544411 | Singapore | 4 | 11 | 12 | IV | 4E | 4E1 |
| 2011\|KT824971\|Indonesia | 2011 | KT824971 | Indonesia | 4 | 11 | 12 | IV | 4E | 4E1 |
| 2011\|KT824976\|Australia | 2011 | KT824976 | Australia | 4 | 11 | 12 | IV | 4E | 4E1 |
| 2011\|JN544410\|Singapore | 2011 | JN544410 | Singapore | 4 | 10 | 6 | IV | 4E | 4E6 |
| 2011\|AB915374\|Indonesia | 2011 | AB915374 | Indonesia | 4 | 12 | 39 | IV | 4E | 4E8 |
| 2012\|AB915373\|Indonesia | 2012 | AB915373 | Indonesia | 4 | 12 | 39 | IV | 4E | 4E8 |
| 2013\|KY818135\|Indonesia | 2013 | KY818135 | Indonesia | 4 | 10 | 6 | IV | 4E | 4E6 |
| 1988\|AB600922\|Indonesia | 1988 | AB600922 | Indonesia | 3 | 6 | 4 | IV | 4F | 4F1 |
| 1988\|AB600923\|Indonesia | 1988 | AB600923 | Indonesia | 3 | 6 | 4 | IV | 4F | 4F2 |
| 1995\|DQ855297\|China | 1995 | DQ855297 | China | 3 | 7 | 8 | IV | 4F | 4F4 |
| 2003\|AB195673\|Seychelles | 2003 | AB195673 | Seychelles | 3 | 8 | 19 | IV | 4F | 4F3 |
| 2006\|EU448412\|Madagascar | 2006 | EU448412 | Madagascar | 3 | 8 | 19 | IV | 4F | 4F3 |
| 2009\|KX646375\|Indonesia | 2009 | KX646375 | Indonesia | 3 | 8 | 21 | IV | 4F | 4F3 |
| 2010\|JN415513\|Malaysia | 2010 | JN415513 | Malaysia | 3 | 9 | 42 | IV | 4F | 4F5 |
| 2010\|KM216682\|Indonesia | 2010 | KM216682 | Indonesia | 3 | 9 | 42 | IV | 4F | 4F5 |
| 2011\|LC148028\|Indonesia | 2011 | LC148028 | Indonesia | 3 | 9 | 42 | IV | 4F | 4F5 |
| 2002\|JN415503\|Fiji | 2002 | JN415503 | Fiji | 6 | 18 | 16 | IV | 4G | 4G5 |
| 2002\|JQ655094\|Solomon_Islands | 2002 | JQ655094 | Solomon_Islands | 6 | 18 | 16 | IV | 4G | 4G5 |
| 2003\|JN415495\|Australia | 2003 | JN415495 | Australia | 6 | 18 | 16 | IV | 4G | 4G5 |
| 2003\|JN415518\|Papua_New_Guinea | 2003 | JN415518 | Papua_New_Guinea | 6 | 18 | 17 | IV | 4G | 4G5 |
| 2003\|JX891658\|Marshall_Islands | 2003 | JX891658 | Marshall_Islands | 6 | 18 | 18 | IV | 4G | 4G5 |
| 2011\|KT824978\|Papua_New_Guinea | 2011 | KT824978 | Papua_New_Guinea | 6 | 19 | 45 | IV | 4G | 4G1 |
| 2011\|KT825048\|Papua_New_Guinea | 2011 | KT825048 | Papua_New_Guinea | 6 | 19 | 46 | IV | 4G | 4G2 |
| 2012\|KT824991\|Papua_New_Guinea | 2012 | KT824991 | Papua_New_Guinea | 6 | 20 | 48 | IV | 4G | 4G4 |
| 2013\|KT825009\|Papua_New_Guinea | 2013 | KT825009 | Papua_New_Guinea | 6 | 20 | 49 | IV | 4G | 4G4 |
| 2013\|KT825010\|Papua_New_Guinea | 2013 | KT825010 | Papua_New_Guinea | 6 | 19 | 46 | IV | 4G | 4G2 |
| 2015\|KT825070\|Papua_New_Guinea | 2015 | KT825070 | Papua_New_Guinea | 6 | 19 | 47 | IV | 4G | 4G3 |
| 2015\|KY495800\|Papua_New_Guinea | 2015 | KY495800 | Papua_New_Guinea | 6 | 20 | 48 | IV | 4G | 4G4 |
| 2016\|KY495801\|Papua_New_Guinea | 2016 | KY495801 | Papua_New_Guinea | 6 | 19 | 46 | IV | 4G | 4G2 |
| 1998\|AB189120\|Indonesia | 1998 | AB189120 | Indonesia | 5 | 13 | 11 | IV | 4H | 4H1 |
| 2001\|DQ672563\|USA.Hawaii | 2001 | DQ672563 | USA.Hawaii | 5 | 13 | 11 | IV | 4H | 4H1 |
| 2001\|FJ898448\|French_Polynesia | 2001 | FJ898448 | French_Polynesia | 5 | 13 | 11 | IV | 4H | 4H1 |
| 2001\|JQ655049\|New_Caledonia | 2001 | JQ655049 | New_Caledonia | 5 | 13 | 11 | IV | 4H | 4H1 |
| 2002\|EU863650\|Easter_Island | 2002 | EU863650 | Easter_Island | 5 | 13 | 11 | IV | 4H | 4H1 |
| 2002\|JN415497\|Cook_Islands | 2002 | JN415497 | Cook_Islands | 5 | 13 | 11 | IV | 4H | 4H1 |
| 2002\|JQ915077\|New_Caledonia | 2002 | JQ915077 | New_Caledonia | 5 | 13 | 11 | IV | 4H | 4H1 |
| 2003\|JQ655061\|New_Caledonia | 2003 | JQ655061 | New_Caledonia | 5 | 13 | 11 | IV | 4H | 4H1 |
| 2003\|JQ655097\|Wallis_and_Futuna | 2003 | JQ655097 | Wallis_and_Futuna | 5 | 13 | 11 | IV | 4H | 4H1 |
| 2006\|JN415498\|Cook_Islands | 2006 | JN415498 | Cook_Islands | 5 | 15 | 30 | IV | 4H | 4H1 |
| 2006\|JN415505\|Fiji | 2006 | JN415505 | Fiji | 5 | 15 | 30 | IV | 4H | 4H1 |
| 2007\|JQ655067\|New_Caledonia | 2007 | JQ655067 | New_Caledonia | 5 | 15 | 30 | IV | 4H | 4H1 |
| 2007\|KT824964\|Cook_Islands | 2007 | KT824964 | Cook_Islands | 5 | 15 | 30 | IV | 4H | 4H1 |
| 2007\|JQ654974\|French_Polynesia | 2007 | JQ654974 | French_Polynesia | 5 | 15 | 31 | IV | 4H | 4H1 |
| 2008\|JF967797\|Tonga | 2008 | JF967797 | Tonga | 5 | 15 | 30 | IV | 4H | 4H1 |
| 2008\|JQ655074\|New_Caledonia | 2008 | JQ655074 | New_Caledonia | 5 | 15 | 30 | IV | 4H | 4H1 |
| 2008\|JQ655033\|French_Polynesia | 2008 | JQ655033 | French_Polynesia | 5 | 16 | 36 | IV | 4H | 4H1 |
| 2009\|JQ915076\|French_Polynesia | 2009 | JQ915076 | French_Polynesia | 5 | 16 | 36 | IV | 4H | 4H1 |
| 2009\|JQ655077\|New_Caledonia | 2009 | JQ655077 | New_Caledonia | 5 | 17 | 40 | IV | 4H | 4H1 |
| 2010\|JQ655096\|Vanuatu | 2010 | JQ655096 | Vanuatu | 5 | 17 | 40 | IV | 4H | 4H1 |
| 2010\|JQ915080\|New_Caledonia | 2010 | JQ915080 | New_Caledonia | 5 | 17 | 40 | IV | 4H | 4H1 |
| 2011\|KT825046\|Fiji | 2011 | KT825046 | Fiji | 5 | 17 | 41 | IV | 4H | 4H1 |
| 2012\|KC316018\|New_Caledonia | 2012 | KC316018 | New_Caledonia | 5 | 17 | 41 | IV | 4H | 4H1 |
| 2012\|KY495797\|Fiji | 2012 | KY495797 | Fiji | 5 | 17 | 41 | IV | 4H | 4H1 |
| 2012\|KY495798\|Kiribati | 2012 | KY495798 | Kiribati | 5 | 17 | 41 | IV | 4H | 4H1 |
| 2012\|KY495799\|Niue | 2012 | KY495799 | Niue | 5 | 17 | 41 | IV | 4H | 4H1 |
| 2013\|KT825019\|Fiji | 2013 | KT825019 | Fiji | 5 | 17 | 41 | IV | 4H | 4H1 |
| 2014\|KM279390\|Fiji | 2014 | KM279390 | Fiji | 5 | 17 | 41 | IV | 4H | 4H1 |
| 1956\|KF289073\|India | 1956 | KF289073 | India | 1 | 1 | 1 | V | 5A | 5A1 |
| 2011\|JN544409\|Singapore | 2011 | JN544409 | Singapore | 1 | 1 | 3 | V | 5A | 5A3 |
| 2014\|KT239347\|Pakistan | 2014 | KT239347 | Pakistan | 1 | 1 | 2 | V | 5A | 5A2 |
| 1962\|JF297571\|India | 1962 | JF297571 | India | 1 | 2 | 4 | V | 5B | 5B4 |
| 1980\|AY732411\|Thailand | 1980 | AY732411 | Thailand | 1 | 2 | 4 | V | 5B | 5B4 |
| 1982\|JF297580\|India | 1982 | JF297580 | India | 1 | 2 | 5 | V | 5B | 5B3 |
| 1983\|AY732379\|Thailand | 1983 | AY732379 | Thailand | 1 | 2 | 4 | V | 5B | 5B4 |
| 1993\|AY762084\|Singapore | 1993 | AY762084 | Singapore | 1 | 2 | 6 | V | 5B | 5B1 |
| 1993\|DQ285562\|Comoros | 1993 | DQ285562 | Comoros | 1 | 2 | 7 | V | 5B | 5B2 |
| 1994\|AM746218\|Saudi_Arabia | 1994 | AM746218 | Saudi_Arabia | 1 | 2 | 7 | V | 5B | 5B2 |
| 2006\|FJ687475\|India | 2006 | FJ687475 | India | 1 | 3 | 32 | V | 5B | 5B5 |
| 2007\|GQ357691\|Singapore | 2007 | GQ357691 | Singapore | 1 | 3 | 32 | V | 5B | 5B5 |
| 2010\|JF967935\|Malaysia | 2010 | JF967935 | Malaysia | 1 | 3 | 33 | V | 5B | 5B5 |
| 2010\|JF754980\|Nepal | 2010 | JF754980 | Nepal | 1 | 4 | 62 | V | 5B | 5B7 |
| 2013\|KJ806950\|Singapore | 2013 | KJ806950 | Singapore | 1 | 3 | 33 | V | 5B | 5B5 |
| 2014\|KT239348\|Pakistan | 2014 | KT239348 | Pakistan | 1 | 2 | 5 | V | 5B | 5B3 |
| 2014\|KU551905\|India | 2014 | KU551905 | India | 1 | 3 | 34 | V | 5B | 5B6 |
| 2015\|KY021900\|India | 2015 | KY021900 | India | 1 | 3 | 33 | V | 5B | 5B5 |
| 2008\|GQ357692\|Singapore | 2008 | GQ357692 | Singapore | 10 | 27 | 39 | V | 5C | 5C1 |
| 2008\|JN415507\|India | 2008 | JN415507 | India | 10 | 28 | 59 | V | 5C | 5C1 |
| 2009\|JF960211\|Singapore | 2009 | JF960211 | Singapore | 10 | 27 | 40 | V | 5C | 5C1 |
| 2009\|HQ149733\|China | 2009 | HQ149733 | China | 10 | 28 | 59 | V | 5C | 5C1 |
| 2009\|JN036371\|Bangladesh | 2009 | JN036371 | Bangladesh | 10 | 28 | 59 | V | 5C | 5C1 |
| 2009\|JQ917404\|India | 2009 | JQ917404 | India | 10 | 28 | 59 | V | 5C | 5C1 |
| 2010\|JN029809\|China | 2010 | JN029809 | China | 10 | 27 | 40 | V | 5C | 5C1 |
| 2010\|KT824969\|India | 2010 | KT824969 | India | 10 | 27 | 40 | V | 5C | 5C1 |
| 2011\|KU509255\|India | 2011 | KU509255 | India | 10 | 27 | 40 | V | 5C | 5C1 |
| 2011\|JN544400\|Singapore | 2011 | JN544400 | Singapore | 10 | 27 | 41 | V | 5C | 5C1 |
| 2012\|KY581728\|India | 2012 | KY581728 | India | 10 | 27 | 40 | V | 5C | 5C1 |
| 2012\|KU570099\|China | 2012 | KU570099 | China | 10 | 28 | 59 | V | 5C | 5C1 |
| 2012\|KX380799\|Singapore | 2012 | KX380799 | Singapore | 10 | 28 | 59 | V | 5C | 5C1 |
| 2013\|KM403635\|Singapore | 2013 | KM403635 | Singapore | 10 | 28 | 60 | V | 5C | 5C1 |
| 2013\|KP849862\|Bhutan | 2013 | KP849862 | Bhutan | 10 | 28 | 59 | V | 5C | 5C1 |
| 2013\|KX225487\|China | 2013 | KX225487 | China | 10 | 28 | 60 | V | 5C | 5C1 |
| 2013\|KY581730\|India | 2013 | KY581730 | India | 10 | 27 | 40 | V | 5C | 5C1 |
| 2014\|KT175110\|India | 2014 | KT175110 | India | 10 | 27 | 40 | V | 5C | 5C1 |
| 2014\|KT239344\|Pakistan | 2014 | KT239344 | Pakistan | 10 | 27 | 40 | V | 5C | 5C1 |
| 2014\|KT825026\|Maldives | 2014 | KT825026 | Maldives | 10 | 27 | 40 | V | 5C | 5C1 |
| 2014\|KT825063\|Australia | 2014 | KT825063 | Australia | 10 | 27 | 40 | V | 5C | 5C1 |
| 2014\|KT825065\|Singapore | 2014 | KT825065 | Singapore | 10 | 28 | 59 | V | 5C | 5C1 |
| 2014\|KX621249\|China | 2014 | KX621249 | China | 10 | 28 | 60 | V | 5C | 5C1 |
| 2014\|LC038145\|Malaysia | 2014 | LC038145 | Malaysia | 10 | 28 | 59 | V | 5C | 5C1 |
| 2014\|LC038147\|Indonesia | 2014 | LC038147 | Indonesia | 10 | 28 | 59 | V | 5C | 5C1 |
| 2015\|KT825042\|Thailand | 2015 | KT825042 | Thailand | 10 | 28 | 59 | V | 5C | 5C1 |
| 2015\|KT825067\|Australia | 2015 | KT825067 | Australia | 10 | 28 | 59 | V | 5C | 5C1 |
| 2015\|KT827378\|China | 2015 | KT827378 | China | 10 | 28 | 60 | V | 5C | 5C1 |
| 2015\|KX721476\|India | 2015 | KX721476 | India | 10 | 27 | 42 | V | 5C | 5C1 |
| 2015\|KY921903\|Singapore | 2015 | KY921903 | Singapore | 10 | 28 | 59 | V | 5C | 5C1 |
| 2016\|KX372686\|China | 2016 | KX372686 | China | 10 | 28 | 59 | V | 5C | 5C1 |
| 2016\|KY495795\|Maldives | 2016 | KY495795 | Maldives | 10 | 27 | 40 | V | 5C | 5C1 |
| 1971\|AY713473\|Myanmar | 1971 | AY713473 | Myanmar | 3 | 8 | 14 | V | 5D | 5D1 |
| 1976\|AF425615\|Myanmar | 1976 | AF425615 | Myanmar | 3 | 8 | 14 | V | 5D | 5D1 |
| 1996\|AY589692\|Myanmar | 1996 | AY589692 | Myanmar | 3 | 8 | 15 | V | 5D | 5D2 |
| 1998\|AY600860\|Myanmar | 1998 | AY600860 | Myanmar | 3 | 8 | 15 | V | 5D | 5D2 |
| 1968\|AF425625\|Nigeria | 1968 | AF425625 | Nigeria | 3 | 7 | 11 | V | 5E | 5E1 |
| 1985\|AF425620\|Cote_dIvoire | 1985 | AF425620 | Cote_dIvoire | 3 | 7 | 11 | V | 5F | 5F1 |
| 1999\|AF298807\|Cote_dIvoire | 1999 | AF298807 | Cote_dIvoire | 3 | 7 | 13 | V | 5G | 5G1 |
| 1970\|JF297578\|India | 1970 | JF297578 | India | 3 | 7 | 12 | V | 5H | 5H1 |
| 1971\|JF297579\|India | 1971 | JF297579 | India | 3 | 7 | 12 | V | 5H | 5H2 |
| 2014\|KT239349\|Pakistan | 2014 | KT239349 | Pakistan | 3 | 7 | 12 | V | 5H | 5H2 |
| 2014\|KT239350\|Pakistan | 2014 | KT239350 | Pakistan | 3 | 7 | 12 | V | 5H | 5H1 |
| 2013\|KF864667\|China | 2013 | KF864667 | China | 3 | 10 | 68 | V | 5I | 5I1 |
| 2013\|KM277610\|Angola | 2013 | KM277610 | Angola | 3 | 10 | 69 | V | 5I | 5I1 |
| 2013\|KU570098\|China | 2013 | KU570098 | China | 3 | 10 | 69 | V | 5I | 5I1 |
| 1977\|D00501\|Jamaica | 1977 | D00501 | Jamaica | 3 | 9 | 18 | V | 5J | 5J1 |
| 1983\|D00504\|Mexico | 1983 | D00504 | Mexico | 3 | 9 | 18 | V | 5J | 5J2 |
| 1985\|D00505\|Aruba | 1985 | D00505 | Aruba | 3 | 9 | 18 | V | 5J | 5J2 |
| 1962\|JF297572\|India | 1962 | JF297572 | India | 2 | 5 | 8 | V | 5K | 5K1 |
| 1962\|JF297573\|India | 1962 | JF297573 | India | 2 | 5 | 8 | V | 5K | 5K2 |
| 1963\|JF297574\|India | 1963 | JF297574 | India | 2 | 5 | 8 | V | 5K | 5K3 |
| 1963\|JF297575\|India | 1963 | JF297575 | India | 2 | 5 | 8 | V | 5K | 5K4 |
| 1963\|JF297576\|India | 1963 | JF297576 | India | 2 | 5 | 8 | V | 5K | 5K5 |
| 1963\|JF297577\|India | 1963 | JF297577 | India | 2 | 5 | 8 | V | 5K | 5K6 |
| 1977\|AF425621\|Jamaica | 1977 | AF425621 | Jamaica | 2 | 6 | 16 | V | 5K | 5K12 |
| 1977\|AF425618\|Grenada | 1977 | AF425618 | Grenada | 2 | 6 | 16 | V | 5K | 5K12 |
| 1977\|JN379475\|Bahamas | 1977 | JN379475 | Bahamas | 2 | 6 | 16 | V | 5K | 5K12 |
| 1978\|JN379481\|Grenada | 1978 | JN379481 | Grenada | 2 | 6 | 16 | V | 5K | 5K12 |
| 1978\|JN379482\|Grenada | 1978 | JN379482 | Grenada | 2 | 6 | 16 | V | 5K | 5K12 |
| 1978\|AF425631\|Trinidad_and_Tobago | 1978 | AF425631 | Trinidad_and_Tobago | 2 | 6 | 16 | V | 5K | 5K17 |
| 1981\|JN379484\|Grenada | 1981 | JN379484 | Grenada | 2 | 6 | 16 | V | 5K | 5K12 |
| 1981\|JN379483\|Trinidad_and_Tobago | 1981 | JN379483 | Trinidad_and_Tobago | 2 | 6 | 16 | V | 5K | 5K12 |
| 1981\|JN379485\|Suriname | 1981 | JN379485 | Suriname | 2 | 6 | 16 | V | 5K | 5K12 |
| 1982\|AF425613\|Brazil | 1982 | AF425613 | Brazil | 2 | 6 | 16 | V | 5K | 5K12 |
| 1982\|DQ341188\|Mexico | 1982 | DQ341188 | Mexico | 2 | 6 | 16 | V | 5K | 5K13 |
| 1983\|AF425624\|Mexico | 1983 | AF425624 | Mexico | 2 | 6 | 16 | V | 5K | 5K13 |
| 1984\|DQ341189\|Mexico | 1984 | DQ341189 | Mexico | 2 | 6 | 16 | V | 5K | 5K13 |
| 1984\|DQ341190\|Mexico | 1984 | DQ341190 | Mexico | 2 | 6 | 16 | V | 5K | 5K16 |
| 1985\|AF425616\|Colombia | 1985 | AF425616 | Colombia | 2 | 6 | 16 | V | 5K | 5K12 |
| 1985\|AF425609\|Aruba | 1985 | AF425609 | Aruba | 2 | 6 | 17 | V | 5K | 5K22 |
| 1986\|AF425639\|Trinidad_and_Tobago | 1986 | AF425639 | Trinidad_and_Tobago | 2 | 6 | 16 | V | 5K | 5K12 |
| 1986\|DQ341191\|Mexico | 1986 | DQ341191 | Mexico | 2 | 6 | 16 | V | 5K | 5K14 |
| 1986\|FJ562106\|Puerto_Rico | 1986 | FJ562106 | Puerto_Rico | 2 | 6 | 17 | V | 5K | 5K22 |
| 1988\|AF425610\|Angola | 1988 | AF425610 | Angola | 2 | 6 | 16 | V | 5K | 5K18 |
| 1989\|AF226687\|French_Guiana | 1989 | AF226687 | French_Guiana | 2 | 6 | 17 | V | 5K | 5K23 |
| 1990\|AF425623\|Mexico | 1990 | AF425623 | Mexico | 2 | 6 | 16 | V | 5K | 5K15 |
| 1992\|FJ410186\|Puerto_Rico | 1992 | FJ410186 | Puerto_Rico | 2 | 6 | 17 | V | 5K | 5K22 |
| 1993\|JN819417\|El_Salvador | 1993 | JN819417 | El_Salvador | 2 | 6 | 16 | V | 5K | 5K19 |
| 1993\|AY153755\|Costa_Rica | 1993 | AY153755 | Costa_Rica | 2 | 6 | 16 | V | 5K | 5K20 |
| 1993\|FJ562105\|Puerto_Rico | 1993 | FJ562105 | Puerto_Rico | 2 | 6 | 17 | V | 5K | 5K23 |
| 1994\|DQ341192\|Mexico | 1994 | DQ341192 | Mexico | 2 | 6 | 16 | V | 5K | 5K21 |
| 1995\|DQ341194\|Mexico | 1995 | DQ341194 | Mexico | 2 | 6 | 16 | V | 5K | 5K21 |
| 1995\|FJ547086\|Puerto_Rico | 1995 | FJ547086 | Puerto_Rico | 2 | 6 | 17 | V | 5K | 5K22 |
| 1995\|FJ205874\|USA | 1995 | FJ205874 | USA | 2 | 6 | 17 | V | 5K | 5K23 |
| 1996\|AF425617\|Colombia | 1996 | AF425617 | Colombia | 2 | 6 | 16 | V | 5K | 5K12 |
| 1996\|FJ410188\|Puerto_Rico | 1996 | FJ410188 | Puerto_Rico | 2 | 6 | 16 | V | 5K | 5K20 |
| 2002\|HM450094\|Brazil | 2002 | HM450094 | Brazil | 2 | 6 | 16 | V | 5K | 5K12 |
| 2006\|EU448413\|India | 2006 | EU448413 | India | 2 | 5 | 10 | V | 5K | 5K9 |
| 2007\|JN903578\|India | 2007 | JN903578 | India | 2 | 5 | 10 | V | 5K | 5K10 |
| 2014\|KT239345\|Pakistan | 2014 | KT239345 | Pakistan | 2 | 5 | 10 | V | 5K | 5K11 |
| 2014\|KT239351\|Pakistan | 2014 | KT239351 | Pakistan | 2 | 5 | 8 | V | 5K | 5K6 |
| 2014\|KT239352\|Pakistan | 2014 | KT239352 | Pakistan | 2 | 5 | 8 | V | 5K | 5K5 |
| 1985\|GQ868601\|Virgin_Islands | 1985 | GQ868601 | Virgin_Islands | 4 | 11 | 19 | V | 5L | 5L1 |
| 2000\|FJ850070\|Brazil | 2000 | FJ850070 | Brazil | 4 | 11 | 19 | V | 5L | 5L1 |
| 2001\|HM450088\|Brazil | 2001 | HM450088 | Brazil | 4 | 11 | 19 | V | 5L | 5L1 |
| 2002\|HM450105\|Brazil | 2002 | HM450105 | Brazil | 4 | 11 | 19 | V | 5L | 5L1 |
| 2003\|HM450096\|Brazil | 2003 | HM450096 | Brazil | 4 | 11 | 19 | V | 5L | 5L1 |
| 2006\|FJ850087\|Brazil | 2006 | FJ850087 | Brazil | 4 | 12 | 49 | V | 5L | 5L3 |
| 2007\|FJ850090\|Brazil | 2007 | FJ850090 | Brazil | 4 | 11 | 19 | V | 5L | 5L1 |
| 2007\|HM450101\|Brazil | 2007 | HM450101 | Brazil | 4 | 12 | 49 | V | 5L | 5L3 |
| 2008\|GU131863\|Brazil | 2008 | GU131863 | Brazil | 4 | 12 | 49 | V | 5L | 5L3 |
| 2009\|HQ026761\|Brazil | 2009 | HQ026761 | Brazil | 4 | 12 | 49 | V | 5L | 5L3 |
| 2010\|HQ026762\|Brazil | 2010 | HQ026762 | Brazil | 4 | 12 | 49 | V | 5L | 5L3 |
| 2010\|KC692513\|Argentina | 2010 | KC692513 | Argentina | 4 | 12 | 50 | V | 5L | 5L3 |
| 2011\|KF672784\|Brazil | 2011 | KF672784 | Brazil | 4 | 12 | 49 | V | 5L | 5L3 |
| 2012\|KY818237\|Brazil | 2012 | KY818237 | Brazil | 4 | 12 | 50 | V | 5L | 5L3 |
| 2013\|KP188545\|Brazil | 2013 | KP188545 | Brazil | 4 | 13 | 70 | V | 5L | 5L2 |
| 2013\|KY818239\|Brazil | 2013 | KY818239 | Brazil | 4 | 12 | 50 | V | 5L | 5L3 |
| 2014\|KU509311\|Brazil | 2014 | KU509311 | Brazil | 4 | 13 | 70 | V | 5L | 5L2 |
| 2016\|KX768338\|Argentina | 2016 | KX768338 | Argentina | 4 | 12 | 49 | V | 5L | 5L3 |
| 2016\|KX768339\|Argentina | 2016 | KX768339 | Argentina | 4 | 13 | 70 | V | 5L | 5L2 |
| 1987\|FJ410190\|Puerto_Rico | 1987 | FJ410190 | Puerto_Rico | 6 | 16 | 22 | V | 5M | 5M6 |
| 1987\|FJ478458\|Puerto_Rico | 1987 | FJ478458 | Puerto_Rico | 6 | 17 | 24 | V | 5M | 5M7 |
| 1991\|AF425626\|Peru | 1991 | AF425626 | Peru | 6 | 16 | 23 | V | 5M | 5M1 |
| 1992\|FJ410187\|Puerto_Rico | 1992 | FJ410187 | Puerto_Rico | 6 | 17 | 24 | V | 5M | 5M7 |
| 1993\|FJ410184\|Puerto_Rico | 1993 | FJ410184 | Puerto_Rico | 6 | 16 | 23 | V | 5M | 5M2 |
| 1993\|FJ410183\|Puerto_Rico | 1993 | FJ410183 | Puerto_Rico | 6 | 16 | 22 | V | 5M | 5M6 |
| 1994\|FJ410175\|Puerto_Rico | 1994 | FJ410175 | Puerto_Rico | 6 | 17 | 24 | V | 5M | 5M7 |
| 1995\|FJ205875\|USA | 1995 | FJ205875 | USA | 6 | 16 | 23 | V | 5M | 5M3 |
| 1995\|JN379486\|Barbados | 1995 | JN379486 | Barbados | 6 | 16 | 23 | V | 5M | 5M4 |
| 1995\|FJ410181\|Puerto_Rico | 1995 | FJ410181 | Puerto_Rico | 6 | 16 | 22 | V | 5M | 5M6 |
| 1995\|FJ410180\|Puerto_Rico | 1995 | FJ410180 | Puerto_Rico | 6 | 17 | 24 | V | 5M | 5M7 |
| 1996\|KF921911\|Puerto_Rico | 1996 | KF921911 | Puerto_Rico | 6 | 16 | 22 | V | 5M | 5M6 |
| 1996\|FJ410182\|Puerto_Rico | 1996 | FJ410182 | Puerto_Rico | 6 | 17 | 24 | V | 5M | 5M7 |
| 1998\|KF955438\|Puerto_Rico | 1998 | KF955438 | Puerto_Rico | 6 | 16 | 22 | V | 5M | 5M6 |
| 1998\|EU482592\|Puerto_Rico | 1998 | EU482592 | Puerto_Rico | 6 | 17 | 24 | V | 5M | 5M7 |
| 1998\|FJ205872\|USA | 1998 | FJ205872 | USA | 6 | 17 | 24 | V | 5M | 5M7 |
| 1998\|KF955437\|Puerto_Rico | 1998 | KF955437 | Puerto_Rico | 6 | 17 | 24 | V | 5M | 5M7 |
| 1999\|AB111065\|Paraguay | 1999 | AB111065 | Paraguay | 6 | 17 | 25 | V | 5M | 5M7 |
| 1999\|AY277652\|Argentina | 1999 | AY277652 | Argentina | 6 | 17 | 25 | V | 5M | 5M7 |
| 2000\|AY277666\|Argentina | 2000 | AY277666 | Argentina | 6 | 17 | 25 | V | 5M | 5M7 |
| 2003\|JN379472\|Barbados | 2003 | JN379472 | Barbados | 6 | 16 | 23 | V | 5M | 5M5 |
| 1986\|HQ026760\|Brazil | 1986 | HQ026760 | Brazil | 5 | 14 | 20 | V | 5N | 5N1 |
| 1988\|KF672761\|Brazil | 1988 | KF672761 | Brazil | 5 | 14 | 20 | V | 5N | 5N1 |
| 1989\|KF672762\|Brazil | 1989 | KF672762 | Brazil | 5 | 14 | 20 | V | 5N | 5N1 |
| 1990\|AF226685\|Brazil | 1990 | AF226685 | Brazil | 5 | 14 | 20 | V | 5N | 5N1 |
| 1991\|KF672791\|Brazil | 1991 | KF672791 | Brazil | 5 | 15 | 26 | V | 5N | 5N7 |
| 1994\|AF425637\|Venezuela | 1994 | AF425637 | Venezuela | 5 | 14 | 20 | V | 5N | 5N3 |
| 1994\|HM450079\|Brazil | 1994 | HM450079 | Brazil | 5 | 15 | 26 | V | 5N | 5N7 |
| 1995\|AF425632\|Venezuela | 1995 | AF425632 | Venezuela | 5 | 14 | 20 | V | 5N | 5N16 |
| 1995\|AF425635\|Venezuela | 1995 | AF425635 | Venezuela | 5 | 14 | 20 | V | 5N | 5N3 |
| 1996\|HM450080\|Brazil | 1996 | HM450080 | Brazil | 5 | 15 | 26 | V | 5N | 5N7 |
| 1997\|FJ639735\|Venezuela | 1997 | FJ639735 | Venezuela | 5 | 14 | 21 | V | 5N | 5N10 |
| 1997\|AF311956\|Brazil | 1997 | AF311956 | Brazil | 5 | 15 | 26 | V | 5N | 5N7 |
| 1998\|FJ639741\|Venezuela | 1998 | FJ639741 | Venezuela | 5 | 14 | 20 | V | 5N | 5N2 |
| 1998\|GU056033\|Venezuela | 1998 | GU056033 | Venezuela | 5 | 14 | 20 | V | 5N | 5N3 |
| 1998\|GU056032\|Venezuela | 1998 | GU056032 | Venezuela | 5 | 14 | 20 | V | 5N | 5N5 |
| 1998\|KF672779\|Brazil | 1998 | KF672779 | Brazil | 5 | 15 | 26 | V | 5N | 5N7 |
| 1998\|JX669470\|Brazil | 1998 | JX669470 | Brazil | 5 | 15 | 26 | V | 5N | 5N8 |
| 1999\|JN379487\|Barbados | 1999 | JN379487 | Barbados | 5 | 14 | 20 | V | 5N | 5N2 |
| 1999\|HM450083\|Brazil | 1999 | HM450083 | Brazil | 5 | 15 | 26 | V | 5N | 5N7 |
| 1999\|JX669471\|Brazil | 1999 | JX669471 | Brazil | 5 | 15 | 26 | V | 5N | 5N8 |
| 2000\|GU131833\|Venezuela | 2000 | GU131833 | Venezuela | 5 | 14 | 20 | V | 5N | 5N2 |
| 2000\|HM450084\|Brazil | 2000 | HM450084 | Brazil | 5 | 15 | 26 | V | 5N | 5N7 |
| 2000\|KF672787\|Brazil | 2000 | KF672787 | Brazil | 5 | 15 | 26 | V | 5N | 5N7 |
| 2000\|KF672763\|Brazil | 2000 | KF672763 | Brazil | 5 | 15 | 26 | V | 5N | 5N8 |
| 2000\|AF514883\|Paraguay | 2000 | AF514883 | Paraguay | 5 | 15 | 26 | V | 5N | 5N9 |
| 2000\|AY277665\|Argentina | 2000 | AY277665 | Argentina | 5 | 15 | 26 | V | 5N | 5N9 |
| 2001\|JN379471\|Barbados | 2001 | JN379471 | Barbados | 5 | 14 | 20 | V | 5N | 5N2 |
| 2001\|AB519681\|Brazil | 2001 | AB519681 | Brazil | 5 | 15 | 26 | V | 5N | 5N7 |
| 2001\|HM450098\|Brazil | 2001 | HM450098 | Brazil | 5 | 15 | 26 | V | 5N | 5N8 |
| 2002\|JX669475\|Brazil | 2002 | JX669475 | Brazil | 5 | 15 | 26 | V | 5N | 5N7 |
| 2002\|HM450091\|Brazil | 2002 | HM450091 | Brazil | 5 | 15 | 26 | V | 5N | 5N9 |
| 2006\|JN819415\|Venezuela | 2006 | JN819415 | Venezuela | 5 | 14 | 21 | V | 5N | 5N10 |
| 2007\|HQ332183\|Venezuela | 2007 | HQ332183 | Venezuela | 5 | 14 | 21 | V | 5N | 5N10 |
| 2008\|KF955410\|Nicaragua | 2008 | KF955410 | Nicaragua | 5 | 14 | 21 | V | 5N | 5N11 |
| 2009\|JF937635\|Nicaragua | 2009 | JF937635 | Nicaragua | 5 | 14 | 21 | V | 5N | 5N12 |
| 2011\|KF973460\|Nicaragua | 2011 | KF973460 | Nicaragua | 5 | 14 | 21 | V | 5N | 5N11 |
| 2011\|KJ189348\|Mexico | 2011 | KJ189348 | Mexico | 5 | 14 | 21 | V | 5N | 5N13 |
| 2012\|JX891659\|El_Salvador | 2012 | JX891659 | El_Salvador | 5 | 14 | 21 | V | 5N | 5N10 |
| 2012\|KF973474\|Nicaragua | 2012 | KF973474 | Nicaragua | 5 | 14 | 21 | V | 5N | 5N11 |
| 2012\|KF973463\|Nicaragua | 2012 | KF973463 | Nicaragua | 5 | 14 | 21 | V | 5N | 5N12 |
| 2012\|KM279419\|Mexico | 2012 | KM279419 | Mexico | 5 | 14 | 21 | V | 5N | 5N12 |
| 2012\|KM279413\|Mexico | 2012 | KM279413 | Mexico | 5 | 14 | 21 | V | 5N | 5N13 |
| 2013\|KY461754\|Costa_Rica | 2013 | KY461754 | Costa_Rica | 5 | 14 | 21 | V | 5N | 5N10 |
| 2013\|KY882515\|Mexico | 2013 | KY882515 | Mexico | 5 | 14 | 21 | V | 5N | 5N12 |
| 2013\|KY882517\|Mexico | 2013 | KY882517 | Mexico | 5 | 14 | 21 | V | 5N | 5N13 |
| 2014\|KM458186\|USA | 2014 | KM458186 | USA | 5 | 14 | 20 | V | 5N | 5N14 |
| 2014\|KM458188\|USA | 2014 | KM458188 | USA | 5 | 14 | 21 | V | 5N | 5N12 |
| 2014\|KM458189\|USA | 2014 | KM458189 | USA | 5 | 14 | 20 | V | 5N | 5N15 |
| 2006\|EU448414\|El_Salvador | 2006 | EU448414 | El_Salvador | 9 | 23 | 36 | V | 5O | 5O1 |
| 2006\|FJ547068\|Nicaragua | 2006 | FJ547068 | Nicaragua | 9 | 23 | 35 | V | 5O | 5O1 |
| 2006\|GU131958\|Mexico | 2006 | GU131958 | Mexico | 9 | 23 | 35 | V | 5O | 5O1 |
| 2006\|FJ562104\|Nicaragua | 2006 | FJ562104 | Nicaragua | 9 | 25 | 46 | V | 5O | 5O2 |
| 2006\|KF955416\|Mexico | 2006 | KF955416 | Mexico | 9 | 24 | 43 | V | 5O | 5O3 |
| 2007\|FJ898433\|Nicaragua | 2007 | FJ898433 | Nicaragua | 9 | 23 | 36 | V | 5O | 5O1 |
| 2007\|KJ189318\|Mexico | 2007 | KJ189318 | Mexico | 9 | 23 | 35 | V | 5O | 5O1 |
| 2007\|KY461738\|Costa_Rica | 2007 | KY461738 | Costa_Rica | 9 | 23 | 35 | V | 5O | 5O1 |
| 2007\|KY461736\|Costa_Rica | 2007 | KY461736 | Costa_Rica | 9 | 25 | 46 | V | 5O | 5O2 |
| 2007\|KJ189330\|Mexico | 2007 | KJ189330 | Mexico | 9 | 24 | 44 | V | 5O | 5O3 |
| 2007\|GU131965\|Mexico | 2007 | GU131965 | Mexico | 9 | 26 | 51 | V | 5O | 5O4 |
| 2008\|JF967804\|Honduras | 2008 | JF967804 | Honduras | 9 | 23 | 36 | V | 5O | 5O1 |
| 2008\|KJ189339\|Mexico | 2008 | KJ189339 | Mexico | 9 | 23 | 35 | V | 5O | 5O1 |
| 2008\|FJ547088\|Nicaragua | 2008 | FJ547088 | Nicaragua | 9 | 25 | 46 | V | 5O | 5O2 |
| 2008\|KJ189315\|Mexico | 2008 | KJ189315 | Mexico | 9 | 24 | 44 | V | 5O | 5O3 |
| 2008\|GQ868537\|Mexico | 2008 | GQ868537 | Mexico | 9 | 26 | 52 | V | 5O | 5O4 |
| 2009\|JQ425062\|USA | 2009 | JQ425062 | USA | 9 | 23 | 36 | V | 5O | 5O1 |
| 2009\|JQ287666\|Nicaragua | 2009 | JQ287666 | Nicaragua | 9 | 23 | 35 | V | 5O | 5O1 |
| 2009\|KJ189345\|Mexico | 2009 | KJ189345 | Mexico | 9 | 23 | 35 | V | 5O | 5O1 |
| 2009\|JQ425068\|USA | 2009 | JQ425068 | USA | 9 | 25 | 47 | V | 5O | 5O2 |
| 2009\|KJ189316\|Mexico | 2009 | KJ189316 | Mexico | 9 | 24 | 43 | V | 5O | 5O3 |
| 2009\|JQ425066\|USA | 2009 | JQ425066 | USA | 9 | 26 | 51 | V | 5O | 5O4 |
| 2009\|KJ189347\|Mexico | 2009 | KJ189347 | Mexico | 9 | 26 | 52 | V | 5O | 5O4 |
| 2010\|KU728185\|Belize | 2010 | KU728185 | Belize | 9 | 23 | 35 | V | 5O | 5O1 |
| 2010\|JQ675358\|USA | 2010 | JQ675358 | USA | 9 | 25 | 47 | V | 5O | 5O2 |
| 2010\|JQ065903\|Mexico | 2010 | JQ065903 | Mexico | 9 | 24 | 45 | V | 5O | 5O3 |
| 2010\|JQ920432\|Mexico | 2010 | JQ920432 | Mexico | 9 | 26 | 52 | V | 5O | 5O4 |
| 2011\|KF973462\|Nicaragua | 2011 | KF973462 | Nicaragua | 9 | 25 | 46 | V | 5O | 5O2 |
| 2011\|KJ189307\|Mexico | 2011 | KJ189307 | Mexico | 9 | 26 | 51 | V | 5O | 5O4 |
| 2012\|KF973471\|Nicaragua | 2012 | KF973471 | Nicaragua | 9 | 25 | 46 | V | 5O | 5O2 |
| 2012\|KY461755\|Costa_Rica | 2012 | KY461755 | Costa_Rica | 9 | 25 | 48 | V | 5O | 5O2 |
| 2012\|KY818279\|Mexico | 2012 | KY818279 | Mexico | 9 | 24 | 44 | V | 5O | 5O3 |
| 2012\|KY882518\|Mexico | 2012 | KY882518 | Mexico | 9 | 26 | 54 | V | 5O | 5O4 |
| 2013\|KJ415092\|Angola | 2013 | KJ415092 | Angola | 9 | 25 | 47 | V | 5O | 5O2 |
| 2013\|KY461750\|Costa_Rica | 2013 | KY461750 | Costa_Rica | 9 | 23 | 36 | V | 5O | 5O1 |
| 2013\|KY461752\|Costa_Rica | 2013 | KY461752 | Costa_Rica | 9 | 25 | 48 | V | 5O | 5O2 |
| 2013\|KY818139\|Honduras | 2013 | KY818139 | Honduras | 9 | 23 | 36 | V | 5O | 5O1 |
| 2013\|KY818266\|Mexico | 2013 | KY818266 | Mexico | 9 | 26 | 52 | V | 5O | 5O4 |
| 2013\|KY882503\|Honduras | 2013 | KY882503 | Honduras | 9 | 25 | 48 | V | 5O | 5O2 |
| 2014\|KM458190\|USA | 2014 | KM458190 | USA | 9 | 23 | 36 | V | 5O | 5O1 |
| 2014\|KU509312\|Nicaragua | 2014 | KU509312 | Nicaragua | 9 | 25 | 48 | V | 5O | 5O2 |
| 2014\|KY461749\|Costa_Rica | 2014 | KY461749 | Costa_Rica | 9 | 25 | 48 | V | 5O | 5O2 |
| 2014\|KY818140\|Honduras | 2014 | KY818140 | Honduras | 9 | 23 | 36 | V | 5O | 5O1 |
| 2016\|KY829115\|Saint_Barthelemy | 2016 | KY829115 | Saint_Barthelemy | 9 | 25 | 48 | V | 5O | 5O2 |
| 1994\|AF425636\|Venezuela | 1994 | AF425636 | Venezuela | 7 | 18 | 27 | V | 5P | 5P1 |
| 1995\|AF425638\|Venezuela | 1995 | AF425638 | Venezuela | 7 | 18 | 27 | V | 5P | 5P1 |
| 1997\|AF425634\|Venezuela | 1997 | AF425634 | Venezuela | 7 | 18 | 27 | V | 5P | 5P1 |
| 1998\|FJ639740\|Venezuela | 1998 | FJ639740 | Venezuela | 7 | 18 | 27 | V | 5P | 5P1 |
| 1998\|KJ189302\|Colombia | 1998 | KJ189302 | Colombia | 7 | 18 | 27 | V | 5P | 5P1 |
| 1998\|KJ189303\|Colombia | 1998 | KJ189303 | Colombia | 7 | 18 | 28 | V | 5P | 5P8 |
| 1999\|FJ639743\|Venezuela | 1999 | FJ639743 | Venezuela | 7 | 18 | 27 | V | 5P | 5P1 |
| 2001\|GU131834\|Venezuela | 2001 | GU131834 | Venezuela | 7 | 18 | 27 | V | 5P | 5P1 |
| 2001\|GU131948\|Colombia | 2001 | GU131948 | Colombia | 7 | 18 | 27 | V | 5P | 5P1 |
| 2002\|JQ581652\|Colombia | 2002 | JQ581652 | Colombia | 7 | 18 | 27 | V | 5P | 5P1 |
| 2006\|JQ581642\|Colombia | 2006 | JQ581642 | Colombia | 7 | 18 | 29 | V | 5P | 5P8 |
| 2006\|JQ581616\|Colombia | 2006 | JQ581616 | Colombia | 7 | 19 | 37 | V | 5P | 5P9 |
| 2006\|KF955413\|Venezuela | 2006 | KF955413 | Venezuela | 7 | 19 | 37 | V | 5P | 5P9 |
| 2007\|JQ581633\|Colombia | 2007 | JQ581633 | Colombia | 7 | 18 | 29 | V | 5P | 5P8 |
| 2007\|HM450103\|Brazil | 2007 | HM450103 | Brazil | 7 | 19 | 37 | V | 5P | 5P9 |
| 2007\|HQ332179\|Venezuela | 2007 | HQ332179 | Venezuela | 7 | 19 | 37 | V | 5P | 5P9 |
| 2008\|FJ850104\|Venezuela | 2008 | FJ850104 | Venezuela | 7 | 18 | 27 | V | 5P | 5P1 |
| 2008\|GQ868570\|Colombia | 2008 | GQ868570 | Colombia | 7 | 20 | 55 | V | 5P | 5P2 |
| 2008\|JQ581604\|Colombia | 2008 | JQ581604 | Colombia | 7 | 20 | 55 | V | 5P | 5P3 |
| 2008\|JQ581619\|Colombia | 2008 | JQ581619 | Colombia | 7 | 18 | 28 | V | 5P | 5P8 |
| 2008\|JN415506\|Guyana | 2008 | JN415506 | Guyana | 7 | 19 | 37 | V | 5P | 5P9 |
| 2008\|JQ581605\|Colombia | 2008 | JQ581605 | Colombia | 7 | 19 | 37 | V | 5P | 5P9 |
| 2008\|KF444789\|Brazil | 2008 | KF444789 | Brazil | 7 | 19 | 37 | V | 5P | 5P9 |
| 2009\|JQ425067\|USA | 2009 | JQ425067 | USA | 7 | 18 | 27 | V | 5P | 5P1 |
| 2009\|KX901653\|Colombia | 2009 | KX901653 | Colombia | 7 | 18 | 29 | V | 5P | 5P8 |
| 2009\|KF444791\|Brazil | 2009 | KF444791 | Brazil | 7 | 19 | 37 | V | 5P | 5P9 |
| 2010\|KX901654\|Colombia | 2010 | KX901654 | Colombia | 7 | 18 | 29 | V | 5P | 5P8 |
| 2010\|KC692517\|Argentina | 2010 | KC692517 | Argentina | 7 | 19 | 38 | V | 5P | 5P9 |
| 2010\|JN713897\|Brazil | 2010 | JN713897 | Brazil | 7 | 19 | 37 | V | 5P | 5P9 |
| 2011\|KY818070\|Colombia | 2011 | KY818070 | Colombia | 7 | 19 | 38 | V | 5P | 5P9 |
| 2011\|KU509254\|Venezuela | 2011 | KU509254 | Venezuela | 7 | 19 | 37 | V | 5P | 5P9 |
| 2012\|KY818097\|Colombia | 2012 | KY818097 | Colombia | 7 | 20 | 55 | V | 5P | 5P3 |
| 2012\|KY818270\|Mexico | 2012 | KY818270 | Mexico | 7 | 20 | 57 | V | 5P | 5P4 |
| 2012\|KY818144\|Colombia | 2012 | KY818144 | Colombia | 7 | 20 | 57 | V | 5P | 5P6 |
| 2012\|KY818229\|Colombia | 2012 | KY818229 | Colombia | 7 | 20 | 58 | V | 5P | 5P7 |
| 2012\|KY818210\|Colombia | 2012 | KY818210 | Colombia | 7 | 19 | 38 | V | 5P | 5P9 |
| 2013\|KY818066\|Colombia | 2013 | KY818066 | Colombia | 7 | 20 | 57 | V | 5P | 5P10 |
| 2013\|KY818069\|Colombia | 2013 | KY818069 | Colombia | 7 | 19 | 38 | V | 5P | 5P9 |
| 2013\|KY818071\|Colombia | 2013 | KY818071 | Colombia | 7 | 20 | 57 | V | 5P | 5P6 |
| 2013\|KY818101\|Colombia | 2013 | KY818101 | Colombia | 7 | 20 | 55 | V | 5P | 5P3 |
| 2013\|KY882519\|Colombia | 2013 | KY882519 | Colombia | 7 | 20 | 57 | V | 5P | 5P5 |
| 2013\|KY882523\|Colombia | 2013 | KY882523 | Colombia | 7 | 20 | 58 | V | 5P | 5P7 |
| 2014\|KX901655\|Colombia | 2014 | KX901655 | Colombia | 7 | 20 | 58 | V | 5P | 5P7 |
| 2014\|KY474306\|Ecuador | 2014 | KY474306 | Ecuador | 7 | 19 | 38 | V | 5P | 5P9 |
| 2014\|MF797878\|Ecuador | 2014 | MF797878 | Ecuador | 7 | 20 | 55 | V | 5P | 5P2 |
| 2015\|KX901656\|Colombia | 2015 | KX901656 | Colombia | 7 | 20 | 55 | V | 5P | 5P3 |
| 2016\|KX768377\|Argentina | 2016 | KX768377 | Argentina | 7 | 19 | 37 | V | 5P | 5P9 |
| 1997\|GU056029\|Venezuela | 1997 | GU056029 | Venezuela | 8 | 21 | 30 | V | 5Q | 5Q1 |
| 1998\|GU056031\|Venezuela | 1998 | GU056031 | Venezuela | 8 | 21 | 30 | V | 5Q | 5Q1 |
| 2000\|GU131832\|Venezuela | 2000 | GU131832 | Venezuela | 8 | 21 | 30 | V | 5Q | 5Q1 |
| 2006\|EU482591\|Puerto_Rico | 2006 | EU482591 | Puerto_Rico | 8 | 21 | 30 | V | 5Q | 5Q1 |
| 2007\|FJ850102\|Venezuela | 2007 | FJ850102 | Venezuela | 8 | 21 | 30 | V | 5Q | 5Q1 |
| 2008\|FJ850103\|Venezuela | 2008 | FJ850103 | Venezuela | 8 | 21 | 30 | V | 5Q | 5Q1 |
| 2008\|JN022597\|Martinique | 2008 | JN022597 | Martinique | 8 | 21 | 30 | V | 5Q | 5Q1 |
| 2010\|JX402213\|Puerto_Rico | 2010 | JX402213 | Puerto_Rico | 8 | 21 | 30 | V | 5Q | 5Q1 |
| 2010\|KU509264\|Haiti | 2010 | KU509264 | Haiti | 8 | 21 | 30 | V | 5Q | 5Q1 |
| 2010\|KU728188\|Dominica | 2010 | KU728188 | Dominica | 8 | 21 | 30 | V | 5Q | 5Q1 |
| 2010\|KC692516\|Argentina | 2010 | KC692516 | Argentina | 8 | 21 | 31 | V | 5Q | 5Q1 |
| 2010\|KU509252\|Venezuela | 2010 | KU509252 | Venezuela | 8 | 21 | 31 | V | 5Q | 5Q1 |
| 2010\|JQ045562\|USA | 2010 | JQ045562 | USA | 8 | 22 | 63 | V | 5Q | 5Q2 |
| 2010\|KJ189360\|Puerto_Rico | 2010 | KJ189360 | Puerto_Rico | 8 | 22 | 63 | V | 5Q | 5Q2 |
| 2011\|KT175076\|China | 2011 | KT175076 | China | 8 | 21 | 30 | V | 5Q | 5Q1 |
| 2012\|KJ189359\|Puerto_Rico | 2012 | KJ189359 | Puerto_Rico | 8 | 21 | 30 | V | 5Q | 5Q1 |
| 2012\|KU509249\|Jamaica | 2012 | KU509249 | Jamaica | 8 | 21 | 30 | V | 5Q | 5Q1 |
| 2012\|KY882524\|Puerto_Rico | 2012 | KY882524 | Puerto_Rico | 8 | 22 | 65 | V | 5Q | 5Q2 |
| 2013\|KJ415284\|USA | 2013 | KJ415284 | USA | 8 | 21 | 30 | V | 5Q | 5Q1 |
| 2013\|KJ676957\|USA | 2013 | KJ676957 | USA | 8 | 22 | 63 | V | 5Q | 5Q2 |
| 2013\|KY818246\|Puerto_Rico | 2013 | KY818246 | Puerto_Rico | 8 | 22 | 65 | V | 5Q | 5Q2 |
| 2013\|KY882522\|Puerto_Rico | 2013 | KY882522 | Puerto_Rico | 8 | 21 | 30 | V | 5Q | 5Q1 |
| 2014\|KT279761\|Haiti | 2014 | KT279761 | Haiti | 8 | 22 | 65 | V | 5Q | 5Q2 |
| 2009\|KC692495\|Argentina | 2009 | KC692495 | Argentina | 11 | 29 | 61 | V | 5R | 5R1 |
| 2010\|KC692515\|Argentina | 2010 | KC692515 | Argentina | 11 | 29 | 61 | V | 5R | 5R1 |
| 2010\|KF672768\|Brazil | 2010 | KF672768 | Brazil | 11 | 29 | 61 | V | 5R | 5R1 |
| 2011\|KF419432\|Paraguay | 2011 | KF419432 | Paraguay | 11 | 29 | 61 | V | 5R | 5R1 |
| 2011\|KP188540\|Brazil | 2011 | KP188540 | Brazil | 11 | 29 | 61 | V | 5R | 5R1 |
| 2012\|KJ651912\|Brazil | 2012 | KJ651912 | Brazil | 11 | 30 | 66 | V | 5R | 5R1 |
| 2013\|KP858107\|Brazil | 2013 | KP858107 | Brazil | 11 | 30 | 66 | V | 5R | 5R1 |
| 2016\|KX372687\|China | 2016 | KX372687 | China | 11 | 30 | 66 | V | 5R | 5R1 |
| 2016\|KX768380\|Argentina | 2016 | KX768380 | Argentina | 11 | 30 | 66 | V | 5R | 5R1 |
